# Mortality and cardiovascular risk of triglyceride-glucose and derived indices in cardiovascular-kidney-metabolic syndrome stages 0-3: a dose-response meta-analysis

**DOI:** 10.3389/fendo.2026.1927610

**Published:** 2026-08-17

**Authors:** Jun-Qiao An, Xue He, Qian-Ying Hao, Yan Wu, Qing-Yong He

**Affiliations:** 1 Guang’anmen Hospital, China Academy of Chinese Medical Sciences, Beijing, China; 2 School of Traditional Chinese Medicine, Beijing University of Chinese Medicine, Beijing, China

**Keywords:** insulin resistance, non-linear association, risk stratification, TyG, visceral adiposity

## Abstract

**Background:**

The associations of the triglyceride-glucose (TyG) index and its derived indices with the progression of cardiovascular-kidney-metabolic (CKM) syndrome remain poorly elucidated.

**Methods:**

We conducted a systematic review and dose-response meta-analysis of 80 observational studies encompassing populations within CKM stages 0–3, evaluating TyG, TyG-BMI, TyG-WC, TyG-WHtR, and CTI for all-cause mortality, cardiovascular disease (CVD) mortality, and CVD incidence (including stroke, coronary heart disease, heart failure and major adverse cardiovascular events). A one-stage mixed-effects approach was used, supplemented by subgroup, meta-regression, leave-one-out, and trim-and-fill analyses.

**Results:**

In both continuous (per 1-SD increment) and categorical (highest vs. lowest) analyses, TyG-derived indices generally demonstrated stronger risk associations with target outcomes than TyG alone. Notably, CTI, TyG-WC, and TyG-WHtR exhibited higher risk associations. In the highest-category analysis, TyG-WC yielded the most pronounced risk for CVD mortality (RR = 1.52, 95% CI: 1.35–1.70, 95% PI: 1.26–1.82). CTI was associated with a 57% increase in incident CVD (RR = 1.57, 95% CI: 1.33–1.85, 95% PI: 1.00–2.46). Conversely, TyG-BMI showed non-significant associations with mortality-related outcomes (P > 0.05), though it predicted a 51% risk increase for incident CVD (RR = 1.51, 95% CI: 1.36–1.67, 95% PI: 1.05–2.16). Subgroup analysis indicated that these positive associations were pronounced in CKM stages 1–3 but entirely non-significant in stage 0 and the obesity subgroup had a significant impact on the risk of mortality. Dose-response analyses revealed a significant non-linear U-shaped association of the TyG index with all-cause mortality (Pnon-linearity < 0.0001; nadir = 8.90). For CVD mortality, the overall association was significant (Poverall = 0.005; nadir = 8.91), whereas a monotonic increasing trend was observed for CVD incidence (Poverall = 0.0014). In sensitivity analyses excluding participants with cancer or consumptive diseases at baseline, as well as those in CKM stage 0, the U-shaped patterns remained stable (mortality nadir shifted to 9.1).

**Conclusions:**

TyG-derived indices, particularly TyG-WC, TyG-WHtR, and CTI, show stronger risk associations than the TyG index for cardiovascular risk assessment in CKM populations, whereas the clinical utility of TyG-BMI is relatively limited. Moderate elevations in TyG were associated with lower mortality risk within a certain range, though the non-linear association was confirmed only for all-cause mortality.

**Systematic review registration:**

https://www.crd.york.ac.uk/PROSPERO/view/CRD420251111362, identifier CRD420251111362.

## Introduction

The triglyceride-glucose (TyG) index, calculated as ln(fasting triglycerides [mg/dL] × fasting glucose [mg/dL]/2), is a simple, cost-effective surrogate marker for insulin resistance (IR) (1). Unlike the homeostatic model assessment of insulin resistance (HOMA-IR), which requires insulin assays, the TyG index can be computed using routine laboratory parameters (2). Recently, the TyG index has gained substantial attention for cardiovascular risk assessment (3–5). Accumulating evidence indicates that IR is a central mechanism driving cardiovascular disease, type 2 diabetes, and metabolic dysfunction (6). This underscores the value of the TyG index as a tool for early risk stratification.

Cardiovascular-Kidney-Metabolic (CKM) syndrome is a high-risk phenotype defined by the clustering of cardiovascular, metabolic, and renal risk factors (7). The American Heart Association (AHA) established a CKM health framework that stages the syndrome from 0 to 4. Stage 0 indicates no risk factors. Stage 1 involves excess or dysfunctional adiposity and impaired glucose tolerance (prediabetes). Stage 2 features established metabolic risk factors or moderate-to-severe chronic kidney disease. Stage 3 represents subclinical cardiovascular disease or heart failure, while Stage 4 denotes established clinical cardiovascular disease. Since Stage 4 patients require secondary prevention, this study focuses on CKM stages 0–3 to facilitate early risk identification. Across these early stages, IR serves as the central pathological feature and the unifying mechanism driving metabolic dysfunction toward cardiorenal organ damage (8).

Although various cohort studies have explored TyG-derived indices combining anthropometric or inflammatory markers, a systematic meta-analysis is required to synthesize and compare these findings in CKM stages 0-3 (5, 9, 10). Indices such as TyG-BMI, TyG-WC, and TyG-WHtR evaluate systemic metabolic abnormalities by pairing adiposity measures with IR status (10–12). Similarly, the C-reactive protein-triglyceride glucose index (CTI) integrates chronic low-grade inflammation and IR, two synergistic drivers of CKM progression (13). However, previous findings regarding these indices and major cardiovascular outcomes are inconsistent (4, 14, 15). A comprehensive comparison of their predictive values in well-staged CKM 0–3 populations is currently lackin. Furthermore, individual cohorts report conflicting results, and the exact dose-response relationships between these composite indices and clinical risks remain poorly characterized (4, 16, 17).

To address these gaps, we conducted a systematic review and dose-response meta-analysis to evaluate the curvilinear associations of TyG, TyG-BMI, TyG-WC, TyG-WHtR, and CTI with clinical risks in a CKM stage 0–3 population. Specifically, we examined all-cause mortality, cardiovascular mortality, and major adverse cardiovascular events, including incident coronary heart disease, stroke, and heart failure. By synthesizing evidence from multiple large cohorts, this study provides a comprehensive framework for risk stratification in early CKM stages.

## Methods

### Study registration and reporting

This systematic review and meta-analysis was designed and reported in accordance with the Preferred Reporting Items for Systematic Reviews and Meta-Analyses (PRISMA) guidelines. The protocol was prospectively registered in PROSPERO prior to data collection (CRD420251111362).

### Literature search

Two investigators (JQA and XH) independently searched PubMed, Embase and Web of Science from inception through the search date, using terms related to TyG index and its derivatives (TyG-BMI, TyG-WC, TyG-WHtR, and CTI) combined with terms for all-cause mortality, CVD mortality and CVD events. No language restrictions were applied. Detailed search strategies are presented in **Supplementary Table 1**. Reference lists of all eligible articles and relevant reviews were manually screened for additional studies.

### Eligibility criteria

We included prospective cohort, retrospective cohort, and cross-sectional studies that reported associations between TyG index or its derivatives and at least one of the following outcomes: all-cause mortality, cardiovascular disease (CVD) mortality, or CVD incidence (including stroke, coronary artery disease [CAD], heart failure, and major adverse cardiovascular events [MACE]).

Eligible studies were required to include adult populations corresponding to the AHA CKM syndrome stages 0 to 3: Stage 0 (individuals with no CKM risk factors); Stage 1 (individuals with excess or dysfunctional adiposity and/or impaired glucose tolerance); Stage 2 (individuals with established metabolic risk factors, such as hypertension or hypertriglyceridemia, type 2 diabetes mellitus, or moderate-to-severe chronic kidney disease); and Stage 3 (individuals with subclinical cardiovascular disease or high predicted CVD risk). Populations with established clinical CVD (Stage 4) at baseline were strictly excluded. The CKM stage classification for each included study was systematically determined based on population characteristics (**Supplementary Table 2**). Studies that enrolled populations explicitly defined as CKM syndrome stages 0–3 according to the AHA 2023 staging framework were classified as explicit CKM cohorts. Studies which populations met CKM stage criteria based on their component conditions were classified as inference CKM cohorts.

Studies were required to report relative risk (RR), hazard ratio (HR), or odds ratio (OR) with 95% confidence intervals, or to provide sufficient data to derive these estimates. We excluded studies conducted exclusively in children or adolescents, studies reporting only intermediate metabolic outcomes without clinical endpoints, and duplicate publications from the same cohort, retaining the most informative or most recent report in such cases.

**Figure 1:**
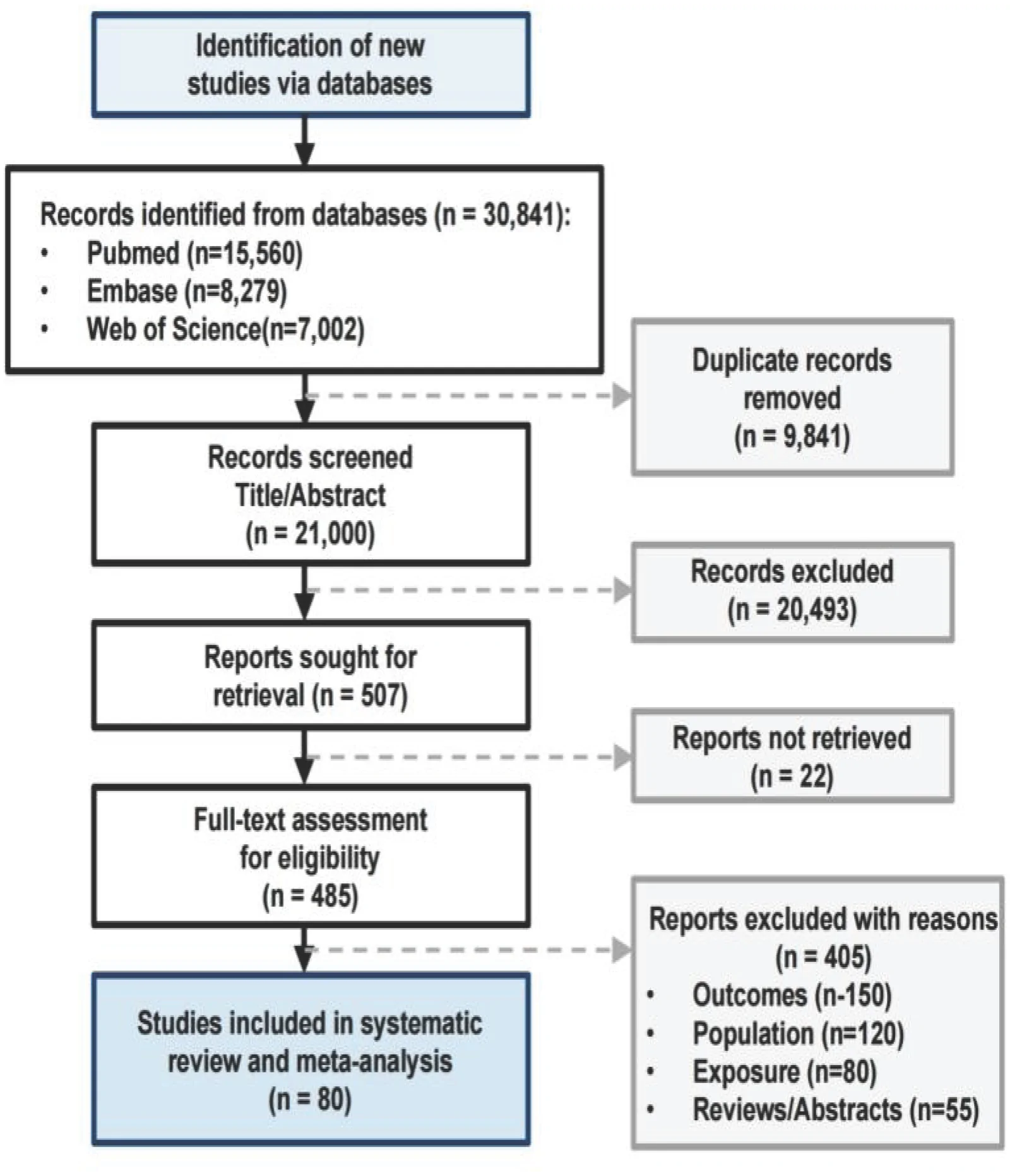
Flow chart of included cohorts.

A total of 30,841 records were initially identified from Pubmed (n = 15,560), Embase (n = 8,279), and Web of Science (n = 7,002). After removing 9,841 duplicate records, 21,000 studies were screened based on titles and abstracts, leaving 507 reports sought for retrieval. Of these, 22 reports were not retrieved, and the remaining 485 reports underwent full-text assessment for eligibility. Following the exclusion of 405 reports with specific reasons, 80 studies were finally included in the systematic review and meta-analysis (**Figure 1**).

### Data extraction

Two reviewers independently (JQA and XH) extracted the following information from each eligible study using a standardized form: first author, publication year, country, study design, cohort population and corresponding CKM stage, sample size, sex distribution, mean or median age, follow-up duration, definitions of TyG index and its derivatives, outcomes, and fully adjusted effect estimates with 95% CIs across exposure categories.

Hazard ratios were treated as relative risks. Odds ratios were converted to RRs using the method of Zhang and Yu when the outcome was common (>10%). Specifically, two distinct formats of effect estimates were extracted to facilitate comprehensive analyses: (1) continuous risk estimates per 1 standard deviation (SD) increment in the TyG index or its derivatives, and (2) categorical dose-response data across exposure quantiles with their corresponding number of cases and total participants. Discrepancies were resolved by discussion or adjudication by a third reviewer (JYD).

### Quality assessment

The methodological quality of each included study was evaluated independently by two reviewers (JQA and XY) using the Risk of Bias in Non-randomized Studies of Exposures (ROBINS-E) tool. Studies were classified as having low, moderate, or high/critical risk of bias. Disagreements were resolved by consensus.

### Statistical analysis

#### Effect size and pooling

Relative risk was used as the common effect measure throughout. Pooled RRs and 95% CIs were estimated using random-effects models with restricted maximum likelihood (REML) estimation to account for anticipated between-study heterogeneity. Heterogeneity was quantified using the I² statistic and Cochran Q test.

#### Continuous and categorical analyses

Two separate meta-analyses were conducted for each index-outcome pair. First, we pooled effect estimates per 1 standard deviation (SD) increase in TyG index or derivative to enable comparisons across exposures and studies. Second, we pooled effect estimates comparing the highest versus the lowest exposure category. For studies reporting continuous effect estimates, the per-1-SD RR was used directly or rescaled as appropriate. For studies reporting categorical data, the lowest category served as the reference.

#### Dose-response meta-analysis

The dose-response relationship between the TyG index and each primary outcome was examined using a robust one-stage mixed-effects meta-analysis approach. To appropriately account for the correlation among effect estimates derived from the same study, within-study covariance matrices were rigorously constructed for categorical data sharing a common reference group. The reference value was explicitly set to the median baseline TyG value of the unexposed group (or the overall median if unexposed data were unavailable). Exposures were modeled with restricted cubic splines (RCS); to determine the optimal curve fit, models with 3, 4, and 5 knots were compared using the Akaike Information Criterion (AIC), with knot locations dynamically placed at pre-specified percentiles of the distribution. Departure from linearity was assessed using the Wald test for the non-linear spline terms (P < 0.05 indicating significant non-linearity), and a separate Wald test was conducted to evaluate the significance of the overall association.

#### Subgroup and meta-regression analyses

Prespecified subgroup analyses were conducted by CKM syndrome stage (stage 0 vs. stages 1–3), glycemic status (no diabetes, prediabetes, diabetes), weight status (normal weight vs. overweight/obese), sex, geographic region, study design, follow-up duration, and sample size. Meta-regression was performed to identify study-level variables that explained between-study heterogeneity. Moderators evaluated included mean age, proportion of male participants, follow-up duration, and sample size.

#### Sensitivity analyses

Several sensitivity analyses were conducted. To assess the potential influence of reverse causation, we restricted the analysis to studies that explicitly excluded participants with severe consumptive diseases (e.g., active cancer, tuberculosis, or other chronic conditions characterized by severe unintended weight loss and hypercatabolism) at baseline. A second sensitivity analysis excluded studies including CKM stage 0 populations. Third, a leave-one-out sensitivity analysis was performed by sequentially omitting one study at a time and recalculating the pooled effect. The statistical robustness was determined by calculating the relative percentage change between the recalculated RR and the original pooled RR. Results were strictly evaluated: models with a maximum RR change of <5% were considered extremely robust, 5–10% as robust, and >10% as requiring further investigation to ensure no single study disproportionately drove the overall conclusions.

#### Publication bias

Publication bias was assessed for outcomes with at least ten eligible studies using Egger’s weighted regression test and visual inspection of funnel plots. When significant asymmetry was detected (P < 0.05), the non-parametric trim-and-fill method was applied. Specifically, a random-effects model with restricted maximum likelihood (REML) estimation was utilized to impute the number of potentially missing studies required to achieve funnel plot symmetry. An adjusted pooled RR, along with updated heterogeneity parameters (adjusted τ² and I²), was then recalculated and visualized via a contour-enhanced funnel plot to confirm whether potential publication bias fundamentally altered the main findings.

All statistical analyses were performed using Stata version 17.0 and R software (packages dosresmeta, metafor). Two-sided P < 0.05 was considered statistically significant.

## Results

### Baseline characteristics of included studies

This meta-analysis included a total of 80 studies, of which 21 explicitly focused on clearly-defined populations with CKM syndrome stages 0-3, and 59 focused on populations belonging to the components of CKM syndrome stages 0-3 (**Supplementary Tables 2**, **S3**). The detailed CKM stage classification for each study is presented in **Supplementary Table 2**.

We evaluated the impact of CKM classification uncertainty. The pooled effect estimates for all major indicator-outcome pairs showed consistent directions between explicit defined and inferred classified CKM 0–3 cohorts (**Supplementary Table 4**). For example, the per 1-SD increase in TyG index was associated with CVD incidence in both explicit (RR = 1.066, 95% CI: 1.026-1.107) and inferred (RR = 1.204, 95% CI: 1.132-1.280) groups. However, the CTI-CVD incidence association showed a larger point estimate in the inferred group (RR = 2.408, I²=95.8%) compared with the explicit group (RR = 1.449, I²=20.3%).

We also evaluated the uncertainties of included cross-sectional studies. Cross-sectional studies tended to show larger effect magnitudes, particularly for CVD mortality. However, after excluding cross-sectional studies, the pooled estimates remained materially unchanged across all outcomes (**Supplementary Table 5**).

According to the literature quality assessment using the ROBINS-E tool, 53 studies (66.25%) were rated as low risk of bias, 23 (28.75%) as moderate risk, and 4 (5%) as high risk (**Supplementary Table 6**).

### Continuous TyG and related indices and risk of CVD events and mortality

In the continuous variable analysis, per 1-SD increment in the TyG index, the risk of all-cause mortality increased by 16% (RR = 1.16, 95% CI: 1.08–1.25), the risk of CVD mortality increased by 20% (RR = 1.20, 95% CI: 1.09-1.33), and the risk of incident CVD increased by 19% (RR = 1.19, 95% CI: 1.15-1.23) (**Table 1**; **Supplementary Figure 1**). Among specific CVD events, the risks for stroke, coronary heart disease, and heart failure were RR = 1.07 (95% CI: 1.02-1.13), 1.15 (95% CI: 1.11-1.20), and 1.28 (95% CI: 1.00-1.64), respectively.

**Table 1:** Summary relative risks of target outcome for an increase of per 1 SD and highest vs. reference category for TyG and derived indicators.

Type	Index	Outcome	RR (95%CI)	PI (95%CI)	I², %	Type	RR (95%CI)	PI (95%CI)	I², %
per 1 SD	TyG	All-cause mortality	1.16 (1.08-1.25)	0.88-1.53	85.9	highest vs. lowest	1.16 (1.10-1.23)	0.95-1.42	82.9
		CVD mortality	1.20 (1.09-1.33)	0.86-1.69	77.3		1.22 (1.09-1.37)	0.83-1.78	55.3
		CVD incidence	1.19 (1.15-1.23)	1.01-1.39	91.5		1.34 (1.26-1.42)	1.01-1.77	93.9
		CHD incidence	1.15 (1.11-1.20)	1.03-1.29	90.5		1.39 (1.23-1.58)	0.93-2.08	96
		Stroke incidence	1.07 (1.02-1.13)	0.93-1.23	80.8		1.21 (1.11-1.32)	0.93-1.58	90.8
		Heart failure incidence	1.28 (1.00-1.64)	0.47-3.53	85		1.27 (0.96-1.69)	0.50-3.22	84.6
		MACE incidence	1.20 (0.99-1.46)	0.65-2.23	77		1.71 (1.26-2.32)	0.68-4.29	83.8
	TyG-BMI	All-cause mortality	1.04 (0.99-1.10)	0.89-1.23	95		1.16 (0.90-1.50)	0.56-2.41	84.4
		CVD mortality	1.12 (0.98-1.28)	0.77-1.64	82.3		1.45 (0.99-2.11)	0.49-4.26	79.6
		CVD incidence	1.11 (1.05-1.19)	0.89-1.40	99		1.51 (1.36-1.67)	1.05-2.16	92.2
		CHD incidence	1.11 (1.01-1.22)	0.72-1.70	99.3		1.47 (1.14-1.89)	0.09-24.67	98
		Stroke incidence	1.02 (0.99-1.04)	0.94-1.10	62.7		1.08 (1.00-1.17)	0.80-1.45	64.1
	TyG-WC	All-cause mortality	1.16 (1.09-1.23)	0.93-1.45	68.1		1.34 (1.15-1.55)	0.85-2.10	77.1
		CVD mortality	1.60 (1.08-2.38)	0.34-7.46	74.8		1.52 (1.35-1.70)	1.26-1.82	0
		CVD incidence	1.17 (1.11-1.23)	0.97-1.41	97.5		1.50 (1.37-1.64)	1.09-2.05	89.8
		CHD incidence	1.18 (1.09-1.28)	0.47-2.97	97.3		/	/	/
		Stroke incidence	1.05 (1.03-1.08)	0.90-1.23	0		1.15 (1.01-1.31)	0.67-1.96	82.9
	TyG-WHtR	All-cause mortality	1.15 (1.09-1.21)	0.99-1.33	66.3		1.33 (1.17-1.51)	0.93-1.89	71
		CVD mortality	1.34 (1.10-1.62)	0.76-2.37	78.6		1.51 (1.31-1.74)	1.14-2.00	15.8
		CVD incidence	1.16 (1.13-1.20)	1.06-1.27	81.9		1.45 (1.31-1.61)	1.01-2.10	91.1
		CHD incidence	1.21 (1.15-1.27)	0.71-2.07	95.4		1.57 (1.20-2.05)	0.08-31.02	97.9
		Stroke incidence	1.05 (1.03-1.08)	0.91-1.22	0		1.10 (1.03-1.17)	0.73-1.66	0
	CTI	All-cause mortality	1.55 (1.39-1.73)	0.76-3.15	0		/	/	/
		CVD incidence	1.21 (1.16-1.25)	1.10-1.32	53.6		1.57 (1.33-1.85)	1.00-2.46	60.8
		Stroke incidence	1.24 (1.19-1.30)	1.16-1.33	0		1.62 (1.33-1.97)	1.07-2.46	16.8

SD, standard deviation; TyG, triglyceride-glucose index; RR, relative risk.

Among TyG-derived indices, CTI, TyG-WC, and TyG-WHtR showed stronger risk associations (**Table 1**; **Supplementary Figures S2**–**S5**). Per 1-SD increment in TyG-WC, the risk of all-cause mortality increased by 16%, CVD mortality increased by 60%, and incident CVD increased by 17%; for TyG-WHtR, all-cause mortality increased by 15%, CVD mortality increased by 34%, and incident CVD increased by 16%; for CTI, all-cause mortality increased by 55%, and incident CVD increased by 21%. In contrast, TyG-BMI did not show significant associations, as its increased risks for all-cause mortality (p>0.05) and CVD mortality (p>0.05) both failed to reach statistical significance, with only the risk of incident CVD significantly increasing by 11%.

Subgroup analyses showed that in populations with CKM stage 0, the TyG index and its derived indices had no significant associations with the risk of incident CVD (TyG: RR = 0.89, 95% CI: 0.695-1.14, p=0.3568; TyG-WHtR: RR = 1.013, 95% CI: 0.798-1.285, p=0.9185), whereas in populations with CKM stages 1 to 3, these indices were significantly positively correlated with the risk of incident CVD. In the weight status subgroup, the all-cause mortality risk for obese populations was RR = 2.475 (95% CI: 1.519-4.032), whereas normal-weight populations showed no significant association (RR = 1.056, 95% CI: 0.885-1.259, p=0.5459) (**Supplementary Tables S7**–**S10** for TyG, **Supplementary Tables S11**–**S13** for TyG-BMI, **Supplementary Table 14** for TyG-WC, **Supplementary Tables S15**–**S17** for TyG-WHtR and **Supplementary Tables S18**, **S19** for CTI).

### Highest category of TyG and related indices and risk of CVD events and mortality

In the categorical variable analysis (highest vs. lowest category), the highest TyG index group had a 16% increased risk of all-cause mortality (RR = 1.16, 95% CI: 1.10–1.23), a 22% increased risk of CVD mortality (RR = 1.22, 95% CI: 1.09-1.37), and a 34% increased risk of incident CVD (RR = 1.34, 95% CI: 1.26-1.42) (**Table 1**; **Supplementary Figure 6**). Among specific CVD events, the risk for stroke was RR = 1.21 (95% CI: 1.11-1.32), for coronary heart disease was RR = 1.39 (95% CI: 1.23-1.58), and for MACE was RR = 1.71 (95% CI: 1.26-2.32).

Among the derived indices, CTI, TyG-WC, and TyG-WHtR showed stronger risk associations (**Table 1**, **Supplementary Figures S6**–**S10**). The highest TyG-WC group had a 34% increase in all-cause mortality, a 52% increase in CVD mortality, and a 50% increase in incident CVD (RR = 1.50); TyG-WHtR had a 33% increase in all-cause mortality, a 51% increase in CVD mortality, and a 45% increase in incident CVD (RR = 1.45); CTI had a 57% increase in incident CVD (RR = 1.57). In contrast, the associations of the highest TyG-BMI group with all-cause mortality and CVD mortality did not reach statistical significance, with only incident CVD increasing by 51% (RR = 1.51). The highest categories of all four derived indices were positively correlated with the risks of incident coronary heart disease and stroke.

Subgroup analyses of the highest category similarly showed that in the CKM stage subgroup, the TyG index of the stage 0 population also had no significant association with the risk of incident CVD (RR = 1.1, 95% CI: 0.585-2.067, p=0.7671). In the glucose metabolism status subgroup, the strengths of the association between the highest TyG index category and the risk of all-cause mortality were sequentially: no diabetes (RR = 1.637, 95% CI: 1.373–1.951), prediabetes (RR = 1.390, 95% CI: 1.215–1.591), and diabetes (RR = 1.101, 95% CI: 1.014–1.196). For the risk of CVD mortality, the highest TyG index group showed statistical significance in the prediabetic population (RR = 1.785, 95% CI: 1.392–2.290), but no significant association was observed in the non-diabetic population (RR = 0.995, 95% CI: 0.923–1.073, p=0.8975). Regarding the risk of CVD mortality for TyG, the obese subgroup had an RR = 1.564 (95% CI: 1.321-1.852), whereas the normal-weight subgroup showed no significant association (RR = 0.941, 95% CI: 0.794-1.116, p=0.4861) (**Supplementary Tables S20**–**S23** for TyG, **Supplementary Tables S24**, **S25** for TyG-BMI, **Supplementary Table 26** for TyG-WC and **Supplementary Table 27** for TyG-WHtR).

### Dose-response meta-analysis

**Figure 2:**
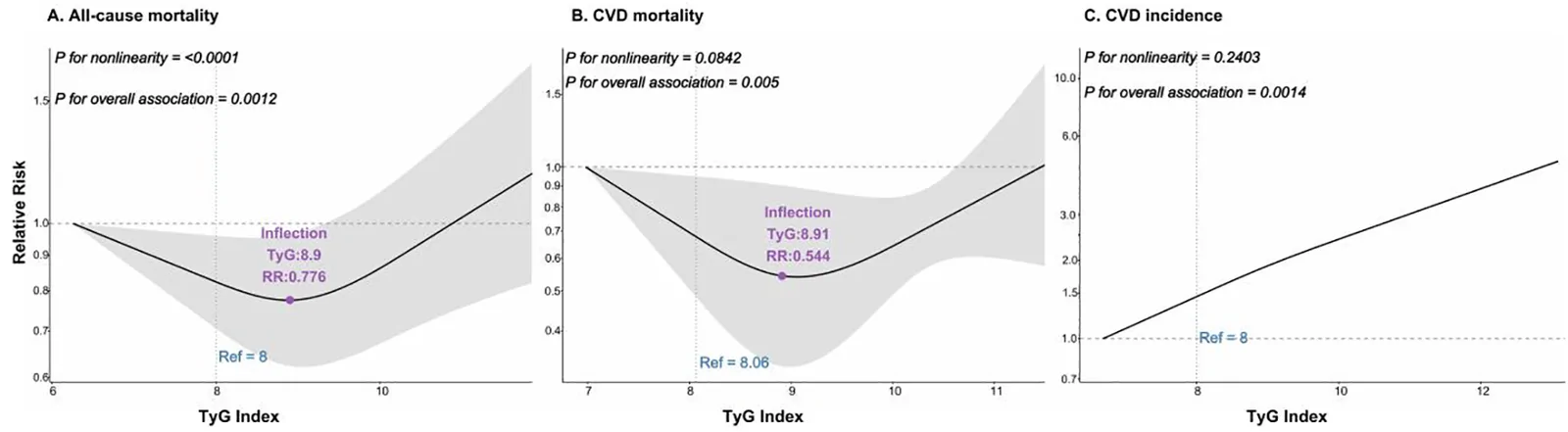
Dose-response associations of TyG index levels with all-cause mortality, CVD mortality and CVD incidence. TyG, triglyceride-glucose. CVD, cardiovascular disease.

In the dose-response meta-analysis, a significant non-linear U-shaped association was observed between the TyG index and all-cause mortality (P for non-linearity < 0.0001, P for overall association = 0.0012) (**Figure 2**). The risk of all-cause mortality reached its nadir when the TyG index was 8.90. For CVD mortality, the overall association was significant (P = 0.005), with the lowest risk at TyG = 8.91. However, the test for non-linearity did not reach statistical significance (P = 0.0842). In contrast, a monotonically increasing trend was observed between the TyG index and incident CVD (P for non-linearity = 0.2403, P for overall association = 0.0014), indicating that its risk of occurrence continuously increased with rising TyG index levels.

**Figure 3:**
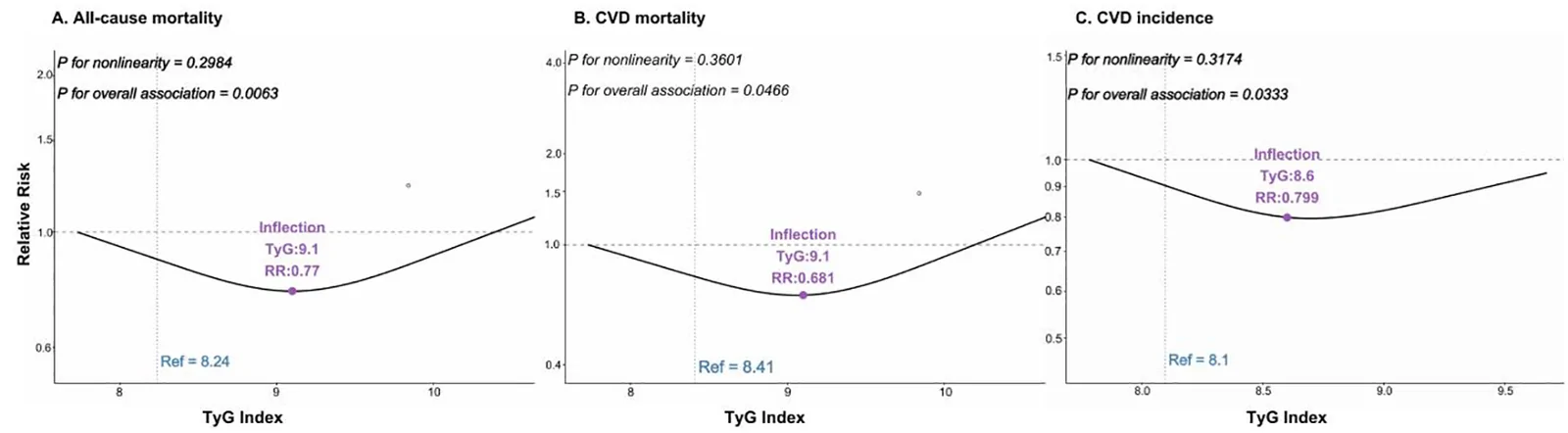
Sensitivity analysis excluding participants with baseline cancer or degenerative disease. TyG, Triglyceride-Glucose. CVD, Cardiovascular Disease.

Sensitivity analysis to evaluate potential reverse causality effects was conducted after excluding participants with cancer or consumptive diseases at baseline (**Figure 3**). The results showed that the overall associations of the TyG index with all-cause mortality (P for overall association = 0.0063) and CVD mortality (P for overall association = 0.0466) remained significant and basically maintained the U-shaped trend, with the lowest risk point shifting to a TyG index of 9.1.

**Figure 4:**
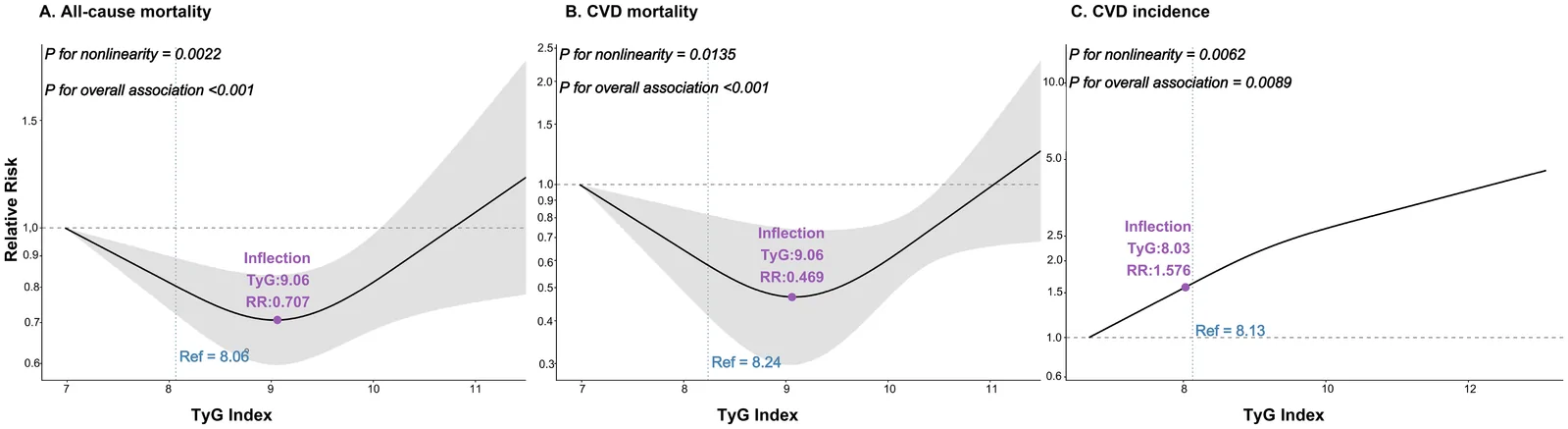
Sensitivity analysis excluding participants with CKM syndrome stage 0. TyG, Triglyceride-Glucose. CVD, Cardiovascular Disease.

In another sensitivity analysis excluding participants in CKM syndrome stage 0, the non-linear test of the dose-response association remained significant (**Figure 4**). Specifically, a significant non-linear U-shaped curve was observed between the TyG index and all-cause mortality (P for non-linearity = 0.0022, P for overall association < 0.0001), a significant non-linear U-shaped curve was observed between the TyG index and CVD mortality (P for non-linearity = 0.0135, P for overall association < 0.0001) and a monotonically increasing trend was observed between the TyG index and CVD incidence (P for non-linearity = 0.0062, P for overall association = 0.0089).

### Meta-regression, leave-one-out analysis

Meta-regression analysis was performed for the associations between major indices and outcomes (**Supplementary Tables S28**–**S41**). TyG and all-cause mortality: Age was the main source of heterogeneity (adjusted R²=35.9%), but there was no significant correlation (P>0.05). TyG and cardiovascular events: The number of cohorts (aRR=0.82, P<0.0001, R²=100%) and age (aRR=0.61, P = 0.006, R²=74.73%) were significant sources of heterogeneity. TyG-BMI and cardiovascular events (10 cohorts): Follow-up time (aRR=1.27, P = 0.004, adjusted R²=99.96%) and the proportion of males (aRR=1.27, P = 0.004) could significantly explain the heterogeneity. TyG-WHtR and cardiovascular events (11 cohorts): The proportion of males (aRR=0.89, P<0.0001, adjusted R²=100%) was a key factor. CTI and cardiovascular events: No significant source of heterogeneity was identified.

The leave-one-out sensitivity analysis showed that the results of most included studies had high robustness, but the robustness of some associations was still insufficient (**Supplementary Figures S11**–**S31**; **Supplementary Tables 42**-**62**). In most cases, removing the study with the largest percentage change consistently produced higher pooled RR estimates. In the per 1 SD analysis, for TyG-WC and CVD mortality, the pooled RR increased to 1.974 (95% CI: 1.412-2.759; I²=0.0%) after excluding Kun Liu 2025. For TyG-BMI and incident CVD, the pooled RR increased to 1.139 (95% CI: 1.129-1.149; I²=92.39%) after excluding Keyu Bian 2025. For the TyG index and incident heart failure, the pooled RR was 1.107 (95% CI: 0.959-1.279; I²=70.8%) after excluding Mao-Jun Liu 2025. In the highest vs lowest analysis, for TyG-BMI and all-cause mortality, the pooled RR increased to 1.338 (95% CI: 1.174-1.525; I²=83.8%) after excluding Kun Liu 2025. The results of the leave-one-out analyses for the remaining associations all showed RR change rates <10%.

### Publication bias

Due to the limited number of eligible studies, only TyG related groups were evaluated for publication bias. The adjusted analysis showed that, except for categorical TyG-CVD mortality group, the remaining indices still maintained statistical significance.

When evaluating per 1-SD increase in the TyG index, the Egger’s test P values for all-cause mortality, CVD incidence, and CVD mortality were 0.009, 0.008, and 0.007, respectively (**Supplementary Figure 32**). To check the reliability of our findings, we performed a trim-and-fill sensitivity analysis. For all-cause mortality, no missing studies were identified, and the pooled effect estimate remained unchanged (adjusted RR = 1.196, adjusted Z = 2.8499, adjusted P = 0.0044). For CVD mortality, the imputation of two missing studies reduced from an original RR of 1.167 to an adjusted RR of 1.1298, while preserving statistical significance (adjusted Z = 2.2611, adjusted P = 0.0238). Similarly, for CVD incidence, after imputing 10 missing studies, the original RR 1.2169 decreased to 1.1212, retained statistical significance (adjusted Z = 2.5056, adjusted P = 0.0122).

When comparing the highest with the lowest group of the TyG index, Egger’s test revealed no evidence of publication bias for all-cause mortality (P = 0.100), whereas significant publication bias was observed for CVD mortality (P = 0.001) and CVD incidence (P = 0.036). The Trim-and-Fill adjustment for CVD mortality imputed eight missing studies, leading original RR of 1.2235 down to an adjusted RR of 1.0678. The association between the highest TyG category and CVD mortality lost statistical significance (adjusted Z = 0.9857, adjusted P = 0.3243) after adjustment. For CVD incidence, imputation of seven missing studies adjusted the effect size downwards from an original RR of 1.3628 to an adjusted RR of 1.2891, which remained significant (adjusted Z = 5.9462, adjusted P < 0.001).

## Discussion

### Principal findings

This systematic review and dose-response meta-analysis of 80 observational studies evaluated the prognostic value of the triglyceride-glucose (TyG) index and its derivatives across cardiovascular-kidney-metabolic (CKM) syndrome stages 0–3. We found that TyG-derived indices, particularly TyG-WC, TyG-WHtR, and CTI, exhibited stronger associations with target outcomes than the TyG index alone. Specifically, CTI showed the strongest link to all-cause mortality, while TyG-WC was most strongly associated with cardiovascular (CV) mortality. Conversely, TyG-BMI failed to predict mortality. Subgroup analyses revealed that these associations were significant only in CKM stages 1–3, not in stage 0, suggesting a metabolic threshold for clinical risk. Obesity significantly amplified the TyG-related mortality risk. Dose-response analyses revealed a U-shaped relationship between the TyG index and all-cause mortality, while a curvilinear trend was observed for CVD mortality, which confirmed as U-shaped after excluding CKM stage 0 individuals. In contrast, the TyG index showed a monotonic, positive association with incident CV events. Sensitivity analyses, including the exclusion of patients with cancer or wasting diseases, confirmed the stability of these U-shaped relationships, including a consistent U-shaped association with heart failure. These findings highlight the clinical utility of the TyG index and its derivatives in risk stratification for the CKM population.

### Differential prognostic value of TyG-derived indices in CKM syndrome

#### Evidence from prior studies

The higher effect estimates of waist-based TyG derivatives over TyG-BMI align with a growing body of cohort evidence. Zhang et al (9) in a NHANES-based cohort of 6,383 CKM stage 0–3 participants, reported that TyG-WHtR and TyG-WC were significantly associated with all-cause and cardiovascular mortality, whereas TyG-BMI showed comparably weaker or stage-dependent associations. Liu et al. (10) similarly confirmed across a UK Biobank cohort of 282,920 CKM stage 0–3 participants that TyG-WC and TyG-WHtR yielded higher hazard ratios for cardiovascular outcomes than TyG-BMI, with significant net reclassification improvements. Studies in metabolic syndrome populations have paralleled these findings: Wang et al. (18) demonstrated that TyG-WC and TyG-WHtR were independently associated with cardiovascular mortality among metabolic syndrome participants (TyG-WC: aHR = 1.45, 95% CI 1.13-1.85; TyG-WHtR: aHR = 1.50, 95% CI 1.17-1.92). Comparable results were observed by Liang et al (19). in the metabolic dysfunction-associated steatotic liver disease population, where TyG-WHtR achieved a C-index of 0.615 for cardiovascular mortality versus only 0.561 for TyG alone. In aging cohorts, Meng et al. demonstrated that TyG and TyG-WHtR were significantly associated with all-cause and cardiovascular mortality, while TyG-BMI was not statistically significant in the phenotypic age-acceleration subgroup (20).

The CTI index, integrating CRP with TyG, has received increasing validation as a superior composite marker. Sun et al. (13) reported in a NHANES retrospective cohort that higher CTI quartiles were significantly associated with CVD mortality and all-cause mortality, showing stronger risk associations than TyG or CRP alone. A parallel dual-cohort study validating CTI in both NHANES and CHARLS populations (21) confirmed its superior prognostic utility over both TyG and isolated CRP, with the highest CTI quartile associated with substantially elevated all-cause and cardiovascular mortality risk. In the specific CKM 0–3 context, Ou et al. confirmed that CTI was positively and independently associated with future CVD risk, with a stronger predictive capacity than either TyG or CRP in stage-stratified analyses (22).

#### Biological mechanisms

The inferior performance of TyG-BMI relative to waist-based indices is mechanistically grounded in well-established limitations of BMI as a measure of adiposity. BMI reflects total body mass indexed to height but fails to discriminate between visceral and subcutaneous adipose tissue, and cannot distinguish lean muscle mass from fat mass. This distinction is clinically critical because visceral adipose tissue (VAT), rather than total or subcutaneous adiposity, is the metabolically active depot primarily responsible for dysregulated adipokine secretion, elevated free fatty acid flux to the liver and myocardium, and ectopic lipid deposition in cardiorenal organs—all pathways directly relevant to CKM progression from stage 1 (metabolic risk accumulation) through stages 2-3 (subclinical and clinically manifest organ damage) (23, 24). Waist circumference and waist-to-height ratio, by contrast, are validated proxies for VAT, with meta-analytic data consistently demonstrating that waist-based measures show stronger risk associations than BMI in predicting cardiometabolic mortality: Petursson et al. (2019) showed in a comparison of >190,000 participants that WHtR achieved hazard ratios of 1.28 for all-cause and 1.88 for cardiovascular mortality in the highest versus lowest quintile, substantially exceeding the HR from BMI alone (24–26). Furthermore, the “obesity paradox”—wherein elevated BMI appears paradoxically protective in certain cardiovascular populations—has been substantially attributed to BMI’s inability to distinguish visceral from subcutaneous fat; visceral fat accumulation, captured by WC and WHtR, does not exhibit this paradox and maintains a linear positive association with mortality across populations (27, 28).

The mechanistic advantage of TyG-WC and TyG-WHtR over TyG-BMI thus reflects an additive informational contribution: visceral adiposity expansion promotes dysregulated adipokine secretion (suppressed adiponectin, elevated leptin and resistin), increased hepatic VLDL secretion, enhanced free fatty acid delivery to cardiorenal organs, and sustained pro-inflammatory cytokine output—all of which amplify insulin resistance and accelerate atherosclerotic plaque formation and destabilization via pathways not fully captured by BMI (29–32). CTI’s superiority over TyG alone is explained by the bidirectional, self-amplifying relationship between inflammation and insulin resistance: elevated CRP promotes adipocyte release of TNF-α and IL-6 through NF-κB pathway activation, which directly inhibits phosphorylation of insulin receptor substrate-1 (IRS-1), thereby worsening insulin resistance; in turn, the hyperglycemia and lipotoxicity of advanced IR activate the NLRP3 inflammasome, stimulate advanced glycation end-product formation, and further amplify CRP synthesis (33–36).

Beyond the distinction between visceral and subcutaneous fat captured by waist-based TyG indices, body fat distribution provides additional insight. Android (upper-body) obesity is characterized by greater visceral fat accumulation, which drives more pronounced insulin resistance, systemic inflammation, and adverse cardiovascular remodeling. Gynoid (lower-body) obesity, in contrast, represents a substantially lower-risk metabolic phenotype, even in individuals with comparable BMI values (37). This distinction is clinically important because it helps explain why waist-based TyG indices outperform BMI-based ones. Recent echocardiographic evidence further supports this concept, showing that women with gynoid obesity have more preserved myocardial mechanics than those with android obesity, suggesting that fat distribution affects not only metabolic risk but also cardiac structure and function (38).

Another interesting anthropometric phenotype associated with a favorable cardiovascular profile is the concave-shaped chest wall conformation. Individuals with this trait have been reported to have a lower prevalence of ischemic heart disease and generally better long-term cardiovascular outcomes, despite showing altered cardiac geometry on imaging (39, 40). Although the underlying mechanism is not fully understood, this observation reinforces the broader point that simple anthropometric characteristics can carry meaningful prognostic information beyond traditional risk factors.

Together, these findings support the core message of our study: anthropometric traits, when integrated with metabolic biomarkers such as the TyG index, can refine cardiovascular risk assessment beyond what BMI alone can provide. Given the differential prognostic performance discovered here, clinicians managing CKM stage 1–3 patients should prioritize TyG-WC, TyG-WHtR, or CTI over TyG-BMI as the preferred composite insulin resistance markers for mortality risk stratification and treatment targeting.

### Lack of prognostic association in CKM stage 0

#### Evidence from prior studies

The absence of significant associations between all TyG-related indices and mortality or cardiovascular outcomes specifically in CKM stage 0 is consistent with findings from cohort studies analyzing CKM stage-stratified outcomes. Zhang et al. (9) reported in their NHANES cohort that TyG-WC and TyG-WHtR showed pronounced mortality associations in CKM stages 1 and 3 but not in stage 0 (p for interaction < 0.05), consistent with the present meta-analytic findings. In a parallel CKM stage-stratified analysis, studies examining TyG-BMI in the CHARLS cohort (41) demonstrated no significant correlation between TyG-BMI and CVD incidence in CKM stages 0 and 1, which the authors attributed to the absence of significant metabolic burden in these early stages. A broader CKM staging cohort study (21) further confirmed that only advanced CKM stages (2-4) were independently associated with significantly elevated all-cause and cardiovascular mortality, with stage 0 showing no significant excess risk compared to healthy populations. These converging observations across geographically distinct cohorts support a metabolic threshold model in which TyG and its derivatives begin to acquire prognostic signal only upon the emergence of frank metabolic or early organ-level dysfunction.

Supporting evidence from UK Biobank analyses confirms that insulin resistance metrics including TyG-related indices demonstrate significant stage-dependent associations, with their predictive utility increasing across the CKM staging continuum in stages 1–3 but contributing minimally to risk stratification in stage 0 (42).

#### Biological mechanisms

CKM stage 0 is defined by the absence of all recognized cardiometabolic risk factors—normal BMI, normal waist circumference, normoglycemia, normolipidemia, and no kidney disease or cardiovascular pathology (23). In this metabolically healthy state, physiological compensatory mechanisms—including preserved pancreatic beta-cell reserve, intact hepatic glucose disposal, and normal endothelial nitric oxide bioavailability—maintain euglycemia and normal lipid homeostasis even in the presence of incipient insulin resistance, effectively buffering TyG levels within a narrow physiological range and attenuating between-individual variation that drives risk discrimination (43, 44). At stage 0, both TyG values and their within-individual variability are narrowly distributed, limiting the statistical power of these indices to detect incremental risk differences. Critically, the pathophysiological cascades through which insulin resistance promotes adverse outcomes—hepatic VLDL overproduction, endothelial dysfunction via impaired eNOS activation, ectopic lipid deposition, and macrophage-driven plaque formation—are attenuated by intact counter-regulatory mechanisms in metabolically healthy individuals (45–47). As CKM stage advances from 1 (excess adiposity or prediabetes) through 2-3 (established metabolic risk factors, subclinical organ damage), compensatory mechanisms are progressively overwhelmed; TyG values rise, variance increases, and the index transitions from a population-level screening marker to a clinically discriminating risk predictor. This threshold effect parallels observations in the hypertension-mortality literature, where blood pressure intervention benefits emerge primarily in individuals with established cardiovascular risk burden rather than in low-risk normotensive populations (48, 49).

These findings support restricting routine TyG-index risk stratification to individuals at CKM stages 1 and above; measurement in truly stage 0 populations yields negligible prognostic differentiation and should not routinely guide clinical decision-making.

### Dose-response relationships and the non-linear mortality pattern

The U-shaped association between TyG and all-cause mortality, together with the curvilinear pattern observed for CVD mortality, with risk nadirs identified at TyG = 8.90 and 8.91 respectively, warrant careful clinical interpretation distinct from the monotonically increasing trend observed for incident cardiovascular events. Analogous non-linear U-shaped patterns have been extensively reported in the blood pressure–mortality literature among individuals with type 2 diabetes, where apparent risk elevation at lower blood pressure levels has been substantially attributed to reverse causation from pre-existing cardiovascular diseases and malignancy that simultaneously lower blood pressure and elevate mortality risk (50). Wang et al. discovered that J-shaped SBP–mortality associations were largely abolished after excluding participants with baseline cardiovascular disease or cancer, revealing a flattened pattern. In the present meta-analysis, excluding participants with cancer and consumptive diseases preserved the U-shaped mortality pattern with only a modest nadir shift (from TyG=8.90 to 9.1), suggesting that reverse causation from these specific conditions does not fully explain the lower-limb risk elevation. Residual confounding from unmeasured low-grade consumptive states, occult frailty, or advanced CKM-related renal or cardiac cachexia—conditions that simultaneously suppress hepatic lipogenesis and triglyceride output (thereby lowering TyG) while markedly increasing mortality risk—cannot be excluded in this aggregate-data framework (51, 52). Furthermore, it is biologically plausible that severely low TyG values may reflect a phenotype of sarcopenic obesity, inadequate nutritional substrate, or impaired lipid metabolism rather than genuinely favorable insulin sensitivity, particularly in the older, higher-stage CKM populations (53, 54).

Collectively, these findings caution against interpreting TyG minimization as a universally beneficial therapeutic target and underscore the necessity of individualized cardiometabolic risk assessment integrating TyG trajectory with the broader CKM-stage context and competing risk profile.

### Heterogeneity

Substantial between-study heterogeneity was observed across most primary analyses, consistent with prior large-scale meta-analyses of TyG and cardiovascular outcomes (4, 55). Meta-regression identified follow-up duration as the predominant contributor to heterogeneity in TyG-BMI and CVD associations (adjusted R² = 99.96%), mirroring findings from a JACC meta-analysis: blood pressure-cardiovascular meta-analyses in type 2 diabetes where longer follow-up duration similarly explained the majority of between-study variance (50). Age and sex proportion each partially explained variance in TyG-cardiovascular event heterogeneity, reflecting well-documented sex hormone–mediated and aging-related modifications of insulin resistance pathophysiology that modulate TyG’s prognostic performance across demographic strata (9). Despite high I² statistics, directional consistency was observed across the vast majority of included cohorts, supporting the validity of pooled estimates. As emphasized in meta-analytic methodological guidance, directional consistency substantially mitigates the inferential concerns arising from quantitative heterogeneity alone. Remaining unexplained heterogeneity likely reflects differences in population-level CKM stage composition across cohorts, lifestyle patterns and metabolic condition that independently influences TyG distributions, variability in outcome ascertainment methods and composite endpoint definitions, and the inclusion of a minority of cross-sectional studies introducing prevalent-case bias.

### Leave-one-out analyses

Leave-one-out sensitivity analyses identified limited robustness for several specific associations. Notably, the pooled estimate for TyG-WC and cardiovascular mortality was substantially influenced by a single study (RR change rate 59.58% after exclusion), and the TyG-BMI and incident CVD association showed a 13.63% change rate after exclusion of a dominant study. These findings indicate that the TyG-WC cardiovascular mortality estimate and TyG-BMI CVD association should be interpreted with particular caution and that replication in additional large, adequately powered cohort studies—ideally with explicit CKM stage stratification—is required before these specific effect magnitudes are incorporated into clinical decision models. The robustness of TyG-WHtR and CTI associations for primary mortality outcomes was not substantially altered by single-study exclusions, further supporting the inferential stability of these indices as prognostic markers.

### Publication bias

Significant publication bias was detected for several primary associations in both continuous and categorical analyses. While trim-and-fill corrections preserved statistical significance for most outcomes, the adjusted cardiovascular mortality estimate for the categorical TyG analysis crossed the null threshold after imputation of potential missing studies. This finding underscores the need for cautious extrapolation of categorical TyG mortality estimates and highlights the importance of prospective registration and reporting of null or negative TyG-related cohort findings. The stability of the continuous-variable estimates and dose-response shapes after publication bias correction, combined with the directional consistency across included cohorts, supports the statistical authenticity of the primary associations. Future preregistered meta-analyses incorporating gray literature and negative-result repositories would provide more robust bias-adjusted estimates.

### Strengths and limitations

This meta-analysis has several notable strengths. To our knowledge, it represents the first systematic comparison of five TyG-derived composite indices within an explicit CKM staging framework, providing a clinically structured hierarchy directly aligned with the 2023 AHA CKM Health Advisory. The transparent study-level CKM classification framework further enhances methodological rigor and enables validation of stage-specific findings. The adoption of one-stage mixed-effects dose-response methodology, supplemented by comprehensive sensitivity analyses addressing reverse causation, CKM-stage confounding, and single-study influence, substantially advances methodological rigor beyond prior pairwise meta-analyses. The reporting of prediction intervals alongside pooled estimates provides essential transparency regarding the expected range of true effects across future populations.

Several limitations merit acknowledgment. First, as an aggregate-data rather than individual-participant-data meta-analysis, adjustment for unmeasured confounders and individual-level subgroup analyses were inherently constrained, and residual confounding cannot be fully excluded. Second, leave-one-out sensitivity analyses revealed moderate to large percentage changes after sequential exclusion of individual studies. However, in most cases, the pooled RR increased after exclusion, indicating that these studies were conservative outliers whose inclusion attenuated rather than inflated the overall estimates. Nevertheless, the magnitude of these changes necessitates cautious interpretation and replication in additional cohorts. Furthermore, publication bias was detected for several primary outcomes, and trim-and-fill adjustment rendered the categorical TyG–CVD mortality association non-significant. Although most adjusted estimates retained statistical significance, this specific finding should be interpreted with particular caution. Third, considerable variation in TyG index calculation methods, endpoint definitions, follow-up durations, and covariate adjustment sets limits direct inter-cohort comparability and contributes to residual hetero. Fourth, the CKM stage classification relied on study-level population characteristics rather than individual participant data. Approximately 59 of 80 studies were classified based on their component conditions rather than explicit CKM syndrome staging. Although effect directions were consistent across explicit and inferred CKM cohorts in our analyses, residual misclassification may have occurred, particularly for studies with limited information to distinguish stage 3 from adjacent stages. Fifth, several limitations affect the comparison across TyG-derived indices. Marked differences existed across indices in terms of the number of studies included in each analysis, study population, length of follow-up, and degree of statistical control. The estimation of per 1-SD effects to facilitate comparison across indices provides only an indirect basis for evaluating relative predictive performance. Conclusions regarding the relative superiority of specific indices should therefore be interpreted cautiously and with allowance for these limitations. Finally, reliance on single baseline TyG measurements in most included studies precludes characterization of dynamic TyG trajectories, which may carry prognostic information independent of baseline values and are particularly relevant in the context of lifestyle or pharmacological interventions across CKM stages 1-2.

### Conclusion

This dose-response meta-analysis of 80 cohorts demonstrates that visceral adiposity- and inflammation-based indices (TyG-WC, TyG-WHtR, and CTI) significantly show stronger risk associations than the standard TyG index and TyG-BMI in predicting mortality and cardiovascular risks. Their prognostic utility is strictly confined to CKM stages 1-3, lacking predictive value in stage 0. Furthermore, while the TyG index monotonically predicts incident cardiovascular events, it exhibits a robust U-shaped association with all-cause mortality and a curvilinear association with cardiovascular mortality. The non-linear pattern for CVD mortality was confirmed after excluding CKM stage 0 individuals. This non-linear risk persists after accounting for potential reverse causation, cautioning against indiscriminate TyG minimization. Future studies using individual participant data and dynamic trajectories are warranted to establish precise, stage-specific therapeutic targets.

## Data Availability

The original contributions presented in the study are included in the article/**Supplementary Material**, further inquiries can be directed to the corresponding author.

## References

[1] Sánchez-García A Rodríguez-Gutiérrez R Mancillas-Adame L González-Nava V Díaz González-Colmenero A Solis RC . Diagnostic accuracy of the triglyceride and glucose index for insulin resistance: A systematic review. Int J Endocrinol. (2020) 2020:4678526. doi: 10.1155/2020/4678526 32256572 PMC7085845

[2] Son DH Lee HS Lee YJ Lee JH Han JH . Comparison of triglyceride-glucose index and HOMA-IR for predicting prevalence and incidence of metabolic syndrome. Nutr Metab Cardiovasc Dis. (2022) 32:596–604. doi: 10.1016/j.numecd.2021.11.017 35090800

[3] Ding X Wang X Wu J Zhang M Cui M . Triglyceride-glucose index and the incidence of atherosclerotic cardiovascular diseases: A meta-analysis of cohort studies. Cardiovasc Diabetol. (2021) 20:76. doi: 10.1186/s12933-021-01268-9 33812373 PMC8019501

[4] Liu X Tan Z Huang Y Zhao H Liu M Yu P . Relationship between the triglyceride-glucose index and risk of cardiovascular diseases and mortality in the general population: A systematic review and meta-analysis. Cardiovasc Diabetol. (2022) 21:124. doi: 10.1186/s12933-022-01546-0 35778731 PMC9250255

[5] Nayak SS Kuriyakose D Polisetty LD Patil AA Ameen D Bonu R . Diagnostic and prognostic value of triglyceride glucose index: A comprehensive evaluation of meta-analysis. Cardiovasc Diabetol. (2024) 23:310. doi: 10.1186/s12933-024-02392-y 39180024 PMC11344391

[6] Ormazabal V Nair S Elfeky O Aguayo C Salomon C Zuñiga FA . Association between insulin resistance and the development of cardiovascular disease. Cardiovasc Diabetol. (2018) 17:122. doi: 10.1186/s12933-018-0762-4 30170598 PMC6119242

[7] Ndumele CE Rangaswami J Chow SL Neeland IJ Tuttle KR Khan SS . Cardiovascular-kidney-metabolic health: A presidential advisory from the American Heart Association. Circulation. (2023) 148:1606–35. doi: 10.1161/cir.0000000000001184 37807924

[8] Marassi M Fadini GP . The cardio-renal-metabolic connection: A review of the evidence. Cardiovasc Diabetol. (2023) 22:195. doi: 10.1186/s12933-023-01937-x 37525273 PMC10391899

[9] Zhang P Mo D Zeng W Dai H . Association between triglyceride-glucose related indices and all-cause and cardiovascular mortality among the population with cardiovascular-kidney-metabolic syndrome stage 0-3: A cohort study. Cardiovasc Diabetol. (2025) 24:92. doi: 10.1186/s12933-025-02642-7 40022225 PMC11871745

[10] Liu K Hu J Huang Y He D Zhang J . Triglyceride-glucose-related indices and risk of cardiovascular disease and mortality in individuals with cardiovascular-kidney-metabolic (CKM) syndrome stages 0-3: A prospective cohort study of 282,920 participants in the UK Biobank. Cardiovasc Diabetol. (2025) 24:277. doi: 10.1186/s12933-025-02842-1 40640813 PMC12243434

[11] Er LK Wu S Chou HH Hsu LA Teng MS Sun YC . Triglyceride glucose-body mass index is a simple and clinically useful surrogate marker for insulin resistance in nondiabetic individuals. PLoS One. (2016) 11:e0149731. doi: 10.1371/journal.pone.0149731 26930652 PMC4773118

[12] Dang K Wang X Hu J Zhang Y Cheng L Qi X . The association between triglyceride-glucose index and its combination with obesity indicators and cardiovascular disease: NHANES 2003-2018. Cardiovasc Diabetol. (2024) 23:8. doi: 10.1186/s12933-023-02115-9 38184598 PMC10771672

[13] Sun Y Guo Y Ma S Mao Z Meng D Xuan K . Association of C-reactive protein-triglyceride glucose index with the incidence and mortality of cardiovascular disease: A retrospective cohort study. Cardiovasc Diabetol. (2025) 24:313. doi: 10.1186/s12933-025-02835-0 40750895 PMC12317521

[14] Liang S Wang C Zhang J Liu Z Bai Y Chen Z . Triglyceride-glucose index and coronary artery disease: A systematic review and meta-analysis of risk, severity, and prognosis. Cardiovasc Diabetol. (2023) 22:170. doi: 10.1186/s12933-023-01906-4 37415168 PMC10327356

[15] Luo JW Duan WH Yu YQ Song L Shi DZ . Prognostic significance of triglyceride-glucose index for adverse cardiovascular events in patients with coronary artery disease: A systematic review and meta-analysis. Front Cardiovasc Med. (2021) 8:774781. doi: 10.3389/fcvm.2021.774781 34926622 PMC8674619

[16] Liu XC He GD Lo K Huang YQ Feng YQ . The triglyceride-glucose index, an insulin resistance marker, was non-linear associated with all-cause and cardiovascular mortality in the general population. Front Cardiovasc Med. (2020) 7:628109. doi: 10.3389/fcvm.2020.628109 33521071 PMC7840600

[17] Liu Q Zhang Y Chen S Xiang H Ouyang J Liu H . Association of the triglyceride-glucose index with all-cause and cardiovascular mortality in patients with cardiometabolic syndrome: A national cohort study. Cardiovasc Diabetol. (2024) 23:80. doi: 10.1186/s12933-024-02152-y 38402393 PMC10893675

[18] Wei X Min Y Song G Ye X Liu L . Association between triglyceride-glucose related indices with the all-cause and cause-specific mortality among the population with metabolic syndrome. Cardiovasc Diabetol. (2024) 23:134. doi: 10.1186/s12933-024-02215-0 38658993 PMC11044377

[19] Min Y Wei X Wei Z Song G Zhao X Lei Y . Prognostic effect of triglyceride glucose-related parameters on all-cause and cardiovascular mortality in the United States adults with metabolic dysfunction-associated steatotic liver disease. Cardiovasc Diabetol. (2024) 23:188. doi: 10.1186/s12933-024-02287-y 38824550 PMC11144336

[20] Cheng MQ Bai ZP Song G Zhang CQ Liu X Li R . The association of triglyceride-glucose index and related indices with all-cause and cardiovascular mortality in biologically aging populations. Med (Baltimore). (2025) 104:e46036. doi: 10.1097/md.0000000000046036 41305805 PMC12643700

[21] Song G . The C-reactive protein-triglyceride glucose index (CTI) predicts mortality in cardiovascular-kidney-metabolic syndrome: A dual-cohort study with machine learning validation. Int J Surg. (2026) 112:1340–52. doi: 10.1097/js9.0000000000003560 41572549 PMC12825706

[22] Ou H Wei M Li X Xia X . C-reactive protein-triglyceride glucose index in evaluating cardiovascular disease and all-cause mortality incidence among individuals across stages 0-3 of cardiovascular-kidney-metabolic syndrome: A nationwide prospective cohort study. Cardiovasc Diabetol. (2025) 24:296. doi: 10.1186/s12933-025-02848-9 40696460 PMC12281998

[23] Ndumele CE Neeland IJ Tuttle KR Chow SL Mathew RO Khan SS . A synopsis of the evidence for the science and clinical management of cardiovascular-kidney-metabolic (CKM) syndrome: A scientific statement from the American Heart Association. Circulation. (2023) 148:1636–64. doi: 10.1161/CIR.0000000000001186 37807920

[24] Cesaro A De Michele G Fimiani F Acerbo V Scherillo G Signore G . Visceral adipose tissue and residual cardiovascular risk: A pathological link and new therapeutic options. Front Cardiovasc Med. (2023) 10:1187735. doi: 10.3389/fcvm.2023.1187735 37576108 PMC10421666

[25] Chait A den Hartigh LJ . Adipose tissue distribution, inflammation and its metabolic consequences, including diabetes and cardiovascular disease. Front Cardiovasc Med. (2020) 7:22. doi: 10.3389/fcvm.2020.00022 32158768 PMC7052117

[26] Gunnarsson S Vito O Unwin RJ . Cardiovascular-kidney-metabolic syndrome: Prevalence, risks, disease trajectories, and early-stage management. Am J Physiol Cell Physiol. (2026) 330:C1–c8. doi: 10.1152/ajpcell.00499.2025 41269265

[27] Zhao M Chen H Zhang Q Zhao T Xu C Yin B . Revisiting the obesity paradox: Visceral fat distribution outperforms BMI in predicting mortality and cardiometabolic risk. Int J Surg. (2026) 112:11932–42. doi: 10.1097/js9.0000000000004912 41632015

[28] Iliodromiti S Celis-Morales CA Lyall DM Anderson J Gray SR Mackay DF . The impact of confounding on the associations of different adiposity measures with the incidence of cardiovascular disease: A cohort study of 296 535 adults of white European descent. Eur Heart J. (2018) 39:1514–20. doi: 10.1093/eurheartj/ehy057 29718151 PMC5930252

[29] Mallick R Basak S Das RK Banerjee A Paul S Pathak S . Fatty acids and their proteins in adipose tissue inflammation. Cell Biochem Biophys. (2024) 82:35–51. doi: 10.1007/s12013-023-01185-6 37794302 PMC10867084

[30] Ghosh A Gao L Thakur A Siu PM Lai CWK . Role of free fatty acids in endothelial dysfunction. J BioMed Sci. (2017) 24:50. doi: 10.1186/s12929-017-0357-5 28750629 PMC5530532

[31] Kwaifa IK Bahari H Yong YK Noor SM . Endothelial dysfunction in obesity-induced inflammation: Molecular mechanisms and clinical implications. Biomolecules. (2020) 10:291. doi: 10.3390/biom10020291 32069832 PMC7072669

[32] Luo J Zhang J Xie Y Wu M Wang Z Ma D . TNF superfamily molecules in atherosclerosis: Mechanistic insights and therapeutic translation. Front Immunol. (2025) 16:1629577. doi: 10.3389/fimmu.2025.1629577 41268556 PMC12626877

[33] Jiao X Tian G . Role of the NLRP3 inflammasome in diabetes and its complications (review). Mol Med Rep. (2025) 32:292. doi: 10.3892/mmr.2025.13657 40849818 PMC12395217

[34] Park JE Han JS . Improving the effect of ferulic acid on inflammation and insulin resistance by regulating the JNK/ERK and NF-κB pathways in TNF-α-treated 3T3-L1 adipocytes. Nutrients. (2024) 16:294. doi: 10.3390/nu16020294 38257186 PMC10819237

[35] Mohallem R Aryal UK . Regulators of TNFα mediated insulin resistance elucidated by quantitative proteomics. Sci Rep. (2020) 10:20878. doi: 10.1038/s41598-020-77914-1 33257747 PMC7705713

[36] Kahleova H Znayenko-Miller T Motoa G Eng E Prevost A Uribarri J . Dietary advanced glycation end-products and their associations with body weight on a Mediterranean diet and low-fat vegan diet: A randomized, cross-over trial. Front Nutr. (2024) 11:1426642. doi: 10.3389/fnut.2024.1426642 39176029 PMC11340516

[37] Pinnick KE Nicholson G Manolopoulos KN McQuaid SE Valet P Frayn KN . Distinct developmental profile of lower-body adipose tissue defines resistance against obesity-associated metabolic complications. Diabetes. (2014) 63:3785–97. doi: 10.2337/db14-0385 24947352

[38] Sonaglioni A Ferrulli A Nicolosi GL Lombardo M Luzi L . The influence of anthropometrics on cardiac mechanics in healthy women with opposite obesity phenotypes (android vs gynoid). Cureus. (2024) 16:e51698. doi: 10.7759/cureus.51698 38187025 PMC10768943

[39] Freed LA Levy D Levine RA Larson MG Evans JC Fuller DL . Prevalence and clinical outcome of mitral-valve prolapse. N Engl J Med. (1999) 341:1–7. doi: 10.1056/nejm199907013410101 10387935

[40] Sonaglioni A Nicolosi GL Bruno A Lombardo M Muti P . Echocardiographic assessment of mitral valve prolapse prevalence before and after the year 1999: A systematic review. J Clin Med. (2024) 13:6160. doi: 10.3390/jcm13206160 39458110 PMC11508471

[41] Li W Shen C Kong W Zhou X Fan H Zhang Y . Association between the triglyceride glucose-body mass index and future cardiovascular disease risk in a population with cardiovascular-kidney-metabolic syndrome stage 0-3: A nationwide prospective cohort study. Cardiovasc Diabetol. (2024) 23:292. doi: 10.1186/s12933-024-02352-6 39113004 PMC11308445

[42] Zhang H Huang S Fang Y Zhang H Yang W Yu M . Insulin resistance assessed by estimated glucose disposal rate predicts cardiovascular disease in stages 0-3 of cardiovascular-kidney-metabolic syndrome: A UK biobank cohort study. Cardiovasc Diabetol. (2025) 24:360. doi: 10.1186/s12933-025-02860-z 40936079 PMC12427105

[43] Lee J Yoon KH . β-cell recovery revisited: Is prediabetes remission driven by insulin sensitivity or β-cell function? J Diabetes Investig. (2026) 17:716–8. doi: 10.1111/jdi.70292 41863035 PMC13137268

[44] Tokgöz S Deden LN Hofboer A Meijer RI Hazebroek EJ van Bon AC . Beta cell function and mass in individuals with and without remission of type 2 diabetes after Roux-en-Y gastric bypass. Diabetologia. (2026) 69:1295–300. doi: 10.1007/s00125-025-06659-1 41555052 PMC13005773

[45] Horton WB Love KM Gregory JM Liu Z Barrett EJ . Metabolic and vascular insulin resistance: Partners in the pathogenesis of cardiovascular disease in diabetes. Am J Physiol Heart Circ Physiol. (2025) 328:H1218–h1236. doi: 10.1152/ajpheart.00826.2024 40257392 PMC12172477

[46] Tan J Li X Dou N . Insulin resistance triggers atherosclerosis: Caveolin 1 cooperates with PKCzeta to block insulin signaling in vascular endothelial cells. Cardiovasc Drugs Ther. (2024) 38:885–93. doi: 10.1007/s10557-023-07477-6 37289375 PMC11438709

[47] Yu M Ge L Fu C Zhao R . Unraveling the metabolic pathways between atherosclerosis and sarcopenia. Front Endocrinol (Lausanne). (2025) 16:1762825. doi: 10.3389/fendo.2025.1762825 41726101 PMC12920195

[48] Basu S Sussman JB Rigdon J Steimle L Denton BT Hayward RA . Benefit and harm of intensive blood pressure treatment: Derivation and validation of risk models using data from the SPRINT and ACCORD trials. PLoS Med. (2017) 14:e1002410. doi: 10.1371/journal.pmed.1002410 29040268 PMC5644999

[49] Zhang Y An J . Projected impact of 2025 AHA/ACC high blood pressure guideline on medication use. Hypertension. (2025) 82:2064–6. doi: 10.1161/hypertensionaha.125.25903 41084800 PMC12614625

[50] Wang S Wang K Gu H Zhang J Cai L Xie W . Blood pressure levels and outcomes in type 2 diabetes: Dose-response meta-analysis of 5.87 million cohort participants. J Am Coll Cardiol. (2026) 87:2355–69. doi: 10.1016/j.jacc.2025.10.068 41563182

[51] Liu B Wu X Wang Y Hu X . Association between cardiac cachexia and adverse outcomes in patients with heart failure: a meta-analysis of cohort studies. Heart. (2025) 111:952–9. doi: 10.1136/heartjnl-2024-325431 40089313

[52] Price SR Wang XH . Protein-energy wasting in chronic kidney disease: mechanisms responsible for loss of muscle mass and function. Kidney Res Clin Pract. (2025) 44:726–40. doi: 10.23876/j.krcp.24.214 39980159 PMC12417578

[53] Huang Q Du X Ouyang W Wang J Liu X . Relationship between triglyceride-glucose index and all-cause mortality in older adults with sarcopenic obesity. Metabol Open. (2025) 27:100388. doi: 10.1016/j.metop.2025.100388 40893912 PMC12398777

[54] Zhao Z Cai R Tao L Sun Y Sun K . Association between triglyceride-glucose index and sarcopenic obesity in adults: a population-based study. Front Nutr. (2025) 12:1452512. doi: 10.3389/fnut.2025.1452512 40290665 PMC12021607

[55] Yu Z Liu W Li B Chen Y Li J . Triglyceride-glucose index and the prognosis of patients with heart failure: a meta-analysis. Biomol BioMed. (2025) 25:278–90. doi: 10.17305/bb.2024.10559 38972053 PMC11734812

