# Correction: Mortality and cardiovascular risk of triglyceride-glucose and derived indices in cardiovascular-kidney-metabolic syndrome stages 0-3: a dose-response meta-analysis

**DOI:** 10.3389/fendo.2026.2006995

**Published:** 2026-10-02

**Authors:** Jun-Qiao An, Xue He, Qian-Ying Hao, Yan Wu, Qing-Yong He

**Affiliations:** 1 Guang’anmen Hospital, China Academy of Chinese Medical Sciences, Beijing, China; 2 School of Traditional Chinese Medicine, Beijing University of Chinese Medicine, Beijing, China

**Keywords:** insulin resistance, non-linear association, risk stratification, TyG, visceral adiposity

The funder “Young Beijing Scholar Program (No. 075), the National Natural Science Foundation of China (No. 82575044) and the Special Program for Cultivating Outstanding Young Scientific and Technological Talents of China Academy of Chinese Medical Sciences (No. CI2023D004)” to “Qing Yong He” was erroneously omitted.

The original version of this article has been updated.

