# Supplementary material for: Mortality and cardiovascular risk of triglyceride-glucose and derived indices in cardiovascular-kidney-metabolic syndrome stages 0-3: a dose-response meta-analysis
Source: Front Endocrinol (Lausanne). 2026 Aug 17;17:1927610. doi: 10.3389/fendo.2026.1927610 (PMC13526669; doi:10.3389/fendo.2026.1927610)
Supplement: Supplementary file 1 [file DataSheet1.pdf]

Supplementary Materials

Figure S1. Forest plot of the association between TyG index (per 1-SD increase) and risk of (A) all-cause mortality, (B) CVD mortality, (C) CVD incidence, (D) CHD incidence, (E) stroke incidence, (F) heart failure incidence and (G) MACE incidence. 5
Figure S2. Forest plot of the association between TyG-BMI index (per 1-SD increase) and risk of (A) all-cause mortality, (B) CVD mortality, (C) CVD incidence, (D) CHD incidence and (E) stroke incidence. 8
Figure S3. Forest plot of the association between TyG-WC index (per 1-SD increase) and risk of (A) all-cause mortality, (B) CVD mortality, (C) CVD incidence, (D) CHD incidence, and (E) stroke incidence. 10
Figure S4. Forest plot of the association between TyG-WhtR index (per 1-SD increase) and risk of (A) all-cause mortality, (B) CVD mortality, (C) CVD incidence, (D) CHD incidence, and (E) stroke incidence. 12
Figure S5. Forest plot of the association between CTI index (per 1-SD increase) and risk of (A) all-cause mortality, (B) CVD incidence, (C) stroke incidence. 14
Figure S6. Forest plot of the association between categorized TyG index (highest vs. reference category) and risk of (A) all-cause mortality, (B) CVD mortality, (C) CVD incidence, (D) CHD incidence, (E) stroke incidence, (F) heart failure incidence and (G) MACE incidence. 15
Figure S7. Forest plot of the association between categorized TyG-BMI index (highest vs. reference category) and risk of (A) all-cause mortality, (B) CVD mortality, (C) CVD incidence, (D) CHD incidence and (E) stroke incidence. 19
Figure S8. Forest plot of the association between categorized TyG-WC index (highest vs. reference category) and risk of (A) all-cause mortality, (B) CVD mortality, (C) CVD incidence, (D) CHD incidence and (E) stroke incidence. 21
Figure S9. Forest plot of the association between categorized TyG-WhtR index (highest vs. reference category) and risk of (A) all-cause mortality, (B) CVD mortality, (C) CVD incidence, (D) CHD incidence and (E) stroke incidence. 23
Figure S10. Forest plot of the association between categorized CTI index (highest vs. reference category) and risk of (A) CVD incidence and (B)Stroke incidence. 25
Figure S11. Leave-one-out analysis for the association between (A)TyG index (per 1-SD increase), (B) categorised TyG index (highest vs. reference category) and risks of all-cause mortality. 26
Figure S12. Leave-one-out analysis for the association between TyG index (per 1-SD increase), (B) categorised TyG index (highest vs. reference category) and risks of CVD mortality. 27
Figure S13. Leave-one-out analysis for the association between TyG index (per 1-SD increase) , (B) categorised TyG index (highest vs. reference category) and risks of CVD incidence. 28
Figure S14. Leave-one-out analysis for the association between TyG index (per 1-SD increase) , (B) categorised TyG index (highest vs. reference category) and risks of CHD incidence. 30
Figure S15. Leave-one-out analysis for the association between TyG index (per 1-SD increase) , (B) categorised TyG index (highest vs. reference category) and risks of stroke incidence. 31
Figure S16. Leave-one-out analysis for the association between TyG index (per 1-SD increase) , (B) categorised TyG index (highest vs. reference category) and risks of heart failure incidence. 32
Figure S17. Leave-one-out analysis for the association between TyG index (per 1-SD increase) , (B) categorised TyG index (highest vs. reference category) and risks of MACE incidence. 33
Figure S18. Leave-one-out analysis for the association between TyG-BMI index (per 1-SD increase), (B) categorised TyG-BMI index (highest vs. reference category) and risks of all-cause mortality. 34
Figure S19. Leave-one-out analysis for the association between TyG-BMI index (per 1-SD increase), (B) categorised TyG index (highest vs. reference category) and risks of CVD mortality. 35
Figure S20. Leave-one-out analysis for the association between TyG-BMI index (per 1-SD increase), (B) categorised TyG index (highest vs. reference category) and risks of CVD incidence. 36
Figure S21. Leave-one-out analysis for the association between TyG-BMI index (per 1-SD increase), (B) categorised TyG index (highest vs. reference category) and risks of CHD incidence. 37
Figure S22. Leave-one-out analysis for the association between TyG-BMI index (per 1-SD increase), (B) categorised TyG index (highest vs. reference category) and risks of stroke incidence. 38
Figure S23. Leave-one-out analysis for the association between TyG-WC index (per 1-SD increase), (B) categorised TyG-WC index (highest vs. reference category) and risks of all-cause mortality. 39

Figure S24. Leave-one-out analysis for the association between TyG-WC index (per 1-SD increase, (B) categorised TyG-WC index (highest vs. reference category) and risks of CVD mortality. 40
Figure S25. Leave-one-out analysis for the association between TyG-WC index (per 1-SD increase), (B) categorised TyG-WC index (highest vs. reference category) and risks of CVD incidence. 41
Figure S26. Leave-one-out analysis for the association between TyG-WC index (per 1-SD increase), (B) categorised TyG-WC index (highest vs. reference category) and risks of stroke incidence. 42
Figure S27. Leave-one-out analysis for the association between TyG-WHtR index (per 1-SD increase), (B) categorised TyG-WHtR index (highest vs. reference category) and risks of all-cause mortality. 43
Figure S28. Leave-one-out analysis for the association between TyG-WHtR index (per 1-SD increase), (B) categorised TyG-WHtR index (highest vs. reference category) and risks of CVD mortality. 44
Figure S29. Leave-one-out analysis for the association between TyG-WHtR index (per 1-SD increase), (B) categorised TyG-WHtR index (highest vs. reference category) and risks of CVD incidence. 45
Figure S30. Leave-one-out analysis for the association between CTI index (per 1-SD increase), (B) categorised CTI index (highest vs. reference category) and risks of CVD incidence. 46
Figure S31. Leave-one-out analysis for the association between CTI index (per 1-SD increase), (B) categorised CTI index (highest vs. reference category) and risks of stroke incidence. 47
Figure 32. Funnel plot and trimfill funnel plot for the association between TyG index (per 1-SD increase), (B) categorised TyG index (highest vs. reference category) and all-cause mortality, CVD mortality and CVD incidence. .. 48
Table S1. Search strategy 50
Table S2. CKM Stage Classification of Included Studies. 51
Table S3. Characteristics of 80 included cohorts. 54
Table S4. Sensitivity analysis of inferred CKM and explicit CKM cohorts: summary relative risks of target outcome for TyG and derived indicators 76
Table S5. Sensitivity analysis excluding cross-sectional studies: summary relative risks of target outcome for TyG and derived indicators 77
Table S6. Risk bias of each included cohort by the ROBINS-E tool. 78
Table S7. Subgroup analysis of the association between TyG index (per 1-SD increase) and risk of all-cause mortality. TyG, Triglyceride-glucose; SD, Standard Deviation. 82
Table S8. Subgroup analysis of the association between TyG index (per 1-SD increase) and risk of CVD mortality; . 83
Table S9. Subgroup analysis of the association between TyG index (per 1-SD increase) and risk of CVD incidence; .. 84
Table S10. Subgroup analysis of the association between TyG index (per 1-SD increase) and risk of MACE incidence; 85
Table S11. Subgroup analysis of the association between TyG-BMI index (per 1-SD increase) and risk of all-cause mortality. 85
Table S12. Subgroup analysis of the association between TyG-BMI index (per 1-SD increase) and risk of CVD mortality. 85
Table S13. Subgroup analysis of the association between TyG-BMI index (per 1-SD increase) and risk of CVD incidence. 86
Table S14. Subgroup analysis of the association between TyG-WC index (per 1-SD increase) and risk of CVD incidence. 86
Table S15. Subgroup analysis of the association between TyG-WHtR index (per 1-SD increase) and risk of all-cause mortality. 87
Table S16. Subgroup analysis of the association between TyG-WHtR index (per 1-SD increase) and risk of CVD mortality. 87
Table S17. Subgroup analysis of the association between TyG-WHtR index (per 1-SD increase) and risk of CVD incidence. 88
Table S18. Subgroup analysis of the association between CTI index (per 1-SD increase) and risk of CVD incidence. 89
Table S19. Subgroup analysis of the association between CTI index (per 1-SD increase) and risk of stroke incidence. 89
Table S20. Subgroup analysis of the association between categorized TyG index (highest vs. reference category) and risk of all-cause mortality. 90

Table S21. Subgroup analysis of the association between categorized TyG index (highest vs. reference category) and risk of CVD mortality. 91
Table S22. Subgroup analysis of the association between categorized TyG index (highest vs. reference category) and risk of CVD incidence. 92
Table S23. Subgroup analysis of the association between categorized TyG index (highest vs. reference category) and risk of stroke incidence. 93
Table S24. Subgroup analysis of the association between categorized TyG-BMI index (highest vs. reference category) and risk of all-cause mortality. 93
Table S25. Subgroup analysis of the association between categorized TyG-BMI index (highest vs. reference category) and risk of CVD incidence. 94
Table S26. Subgroup analysis of the association between categorized TyG-WC index (highest vs. reference category) and risk of CVD incidence. 94
Table S27. Subgroup analysis of the association between categorized TyG-WHtR index (highest vs. reference category) and risk of all-cause mortality. 95
Table S28. Meta-regression of study-level characteristics on associations of TyG Index (per 1-SD increase) with all-cause mortality. 95
Table S29. Meta-regression of study-level characteristics on associations of TyG Index (per 1-SD increase) with CVD mortality. 95
Table S30. Meta-regression of study-level characteristics on associations of TyG Index (per 1-SD increase) with CVD incidence. 95
Table S31. Meta-regression of study-level characteristics on associations of TyG-BMI Index (per 1-SD increase) with CVD incidence. 95
Table S32. Meta-regression of study-level characteristics on associations of TyG-WC Index (per 1-SD increase) with CVD incidence. 96
Table S33. Meta-regression of study-level characteristics on associations of TyG-WHtR Index (per 1-SD increase) with CVD incidence. 96
Table S34. Meta-regression of study-level characteristics on associations of CTI Index (per 1-SD increase) with CVD incidence. 96
Table S35. Meta-regression of study-level characteristics on associations of TyG index (highest vs. reference category) with all-cause mortality. 96
Table S36. Meta-regression of study-level characteristics on associations of TyG index (highest vs. reference category) with CVD mortality. 96
Table S37. Meta-regression of study-level characteristics on associations of TyG index (highest vs. reference category) with CVD incidence. 97
Table S38. Meta-regression of study-level characteristics on associations of TyG index (highest vs. reference category) with stroke incidence. 97
Table S39. Meta-regression of study-level characteristics on associations of TyG-BMI index (highest vs. reference category) with CVD incidence. 97
Table S40. Meta-regression of study-level characteristics on associations of TyG-WC index (highest vs. reference category) with CVD incidence. 97
Table S41. Meta-regression of study-level characteristics on associations of TyG-WHtR index (highest vs. reference category) with CVD incidence. 97
Table S42. Leave-one-out analysis for the association between TyG index (per 1-SD increase), (B) categorised TyG index (highest vs. reference category) and risks of all-cause mortality. 99
Table S43. Leave-one-out analysis for the association between TyG index (per 1-SD increase), (B) categorised TyG index (highest vs. reference category) and risks of CVD mortality. 101
Table S44. Leave-one-out analysis for the association between TyG index (per 1-SD increase), (B) categorised TyG index (highest vs. reference category) and risks of CVD incidence. 102
Table S45. Leave-one-out analysis for the association between TyG index (per 1-SD increase), (B) categorised TyG

index (highest vs. reference category) and risks of CHD incidence. 104
Table S46. Leave-one-out analysis for the association between TyG index (per 1-SD increase), (B) categorised TyG index (highest vs. reference category) and risks of stroke incidence. 105
Table S47. Leave-one-out analysis for the association between TyG index (per 1-SD increase), (B) categorised TyG index (highest vs. reference category) and risks of heart failure incidence. 106
Table S48. Leave-one-out analysis for the association between TyG index (per 1-SD increase), (B) categorised TyG index (highest vs. reference category) and risks of MACE incidence. 107
Table S49. Leave-one-out analysis for the association between TyG-BMI index (per 1-SD increase), (B) categorised TyG index (highest vs. reference category) and risks of all-cause mortality. 108
Table S50. Leave-one-out analysis for the association between TyG-BMI index (per 1-SD increase), (B) categorised TyG index (highest vs. reference category) and risks of CVD mortality. 109
Table S51. Leave-one-out analysis for the association between TyG-BMI index (per 1-SD increase), (B) categorised TyG index (highest vs. reference category) and risks of CVD incidence. 110
Table S52. Leave-one-out analysis for the association between TyG-BMI index (per 1-SD increase) and risks of CHD incidence. 111
Table S53. Leave-one-out analysis for the association between TyG-BMI index (per 1-SD increase), (B) categorised TyG index (highest vs. reference category) and risks of stroke incidence. 112
Table S54. Leave-one-out analysis for the association between TyG-WC index (per 1-SD increase), (B) categorised TyG-WC index (highest vs. reference category) and risks of CVD mortality. 112
Table S55. Leave-one-out analysis for the association between TyG-WC index (per 1-SD increase), (B) categorised TyG-WC index (highest vs. reference category) and risks of CVD incidence. 113
Table S56. Leave-one-out analysis for the association between TyG-WC index (per 1-SD increase), (B) categorised TyG-WC index (highest vs. reference category) and risks of stroke incidence. 114
Table S57. Leave-one-out analysis for the association between TyG-WHtR index (per 1-SD increase), (B) categorised TyG-WHtR index (highest vs. reference category) and risks of all-cause mortality. 114
Table S58. Leave-one-out analysis for the association between TyG-WHtR index (per 1-SD increase), (B) categorised TyG-WHtR index (highest vs. reference category) and risks of CVD mortality. 115
Table S59. Leave-one-out analysis for the association between TyG-WHtR index (per 1-SD increase), (B) categorised TyG-WHtR index (highest vs. reference category) and risks of CVD incidence. 116
Table S60. Leave-one-out analysis for the association between TyG-WC index (per 1-SD increase), (B) categorised TyG-WC index (highest vs. reference category) and risks of all-cause mortality. 117
Table S61. Leave-one-out analysis for the association between CTI index (per 1-SD increase), (B) categorised CTI index (highest vs. reference category) and risks of CVD incidence. 118
Table S62. Leave-one-out analysis for the association between CTI index (per 1-SD increase), (B) categorised CTI index (highest vs. reference category) and risks of stroke incidence. 119

Figure S1. Forest plot of the association between TyG index (per 1-SD increase) and risk of (A) all-cause mortality, (B) CVD mortality, (C) CVD incidence, (D) CHD incidence, (E) stroke incidence, (F) heart failure incidence and (G) MACE incidence. TyG, Triglyceride-glucose; SD, Standard Deviation; CVD, Cardiovascular Disease; CHD, Coronary Heart Disease; MACE, Major Adverse Cardiovascular Event.

(A) All-cause mortality

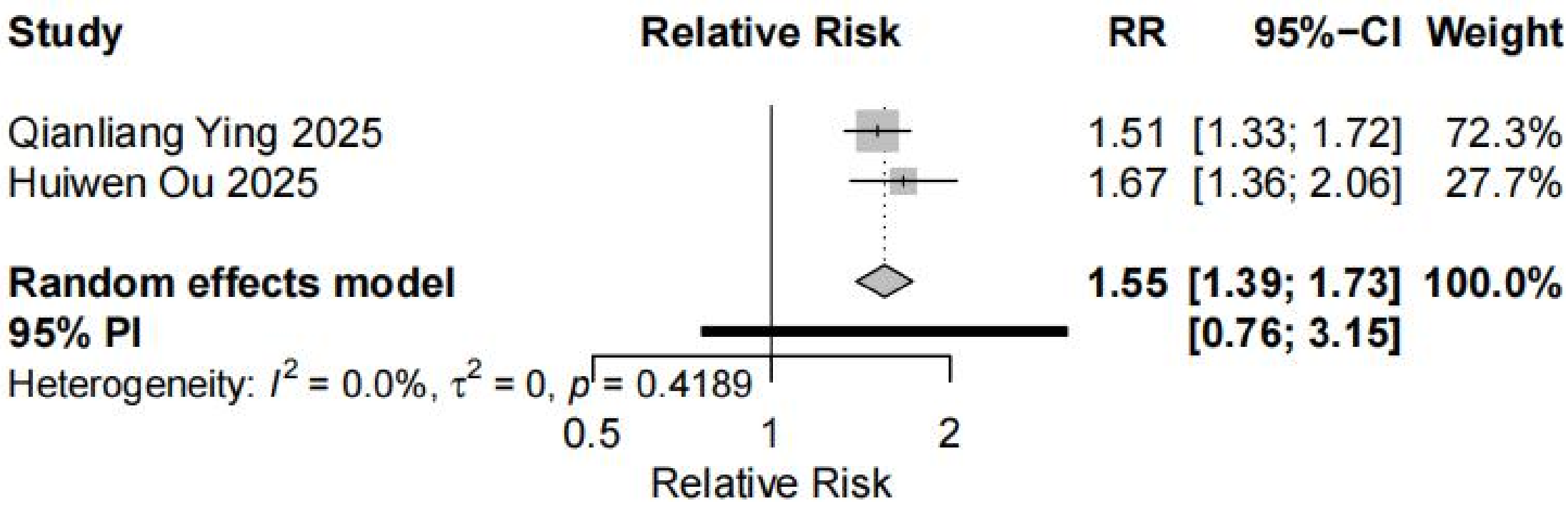

(B) CVD mortality

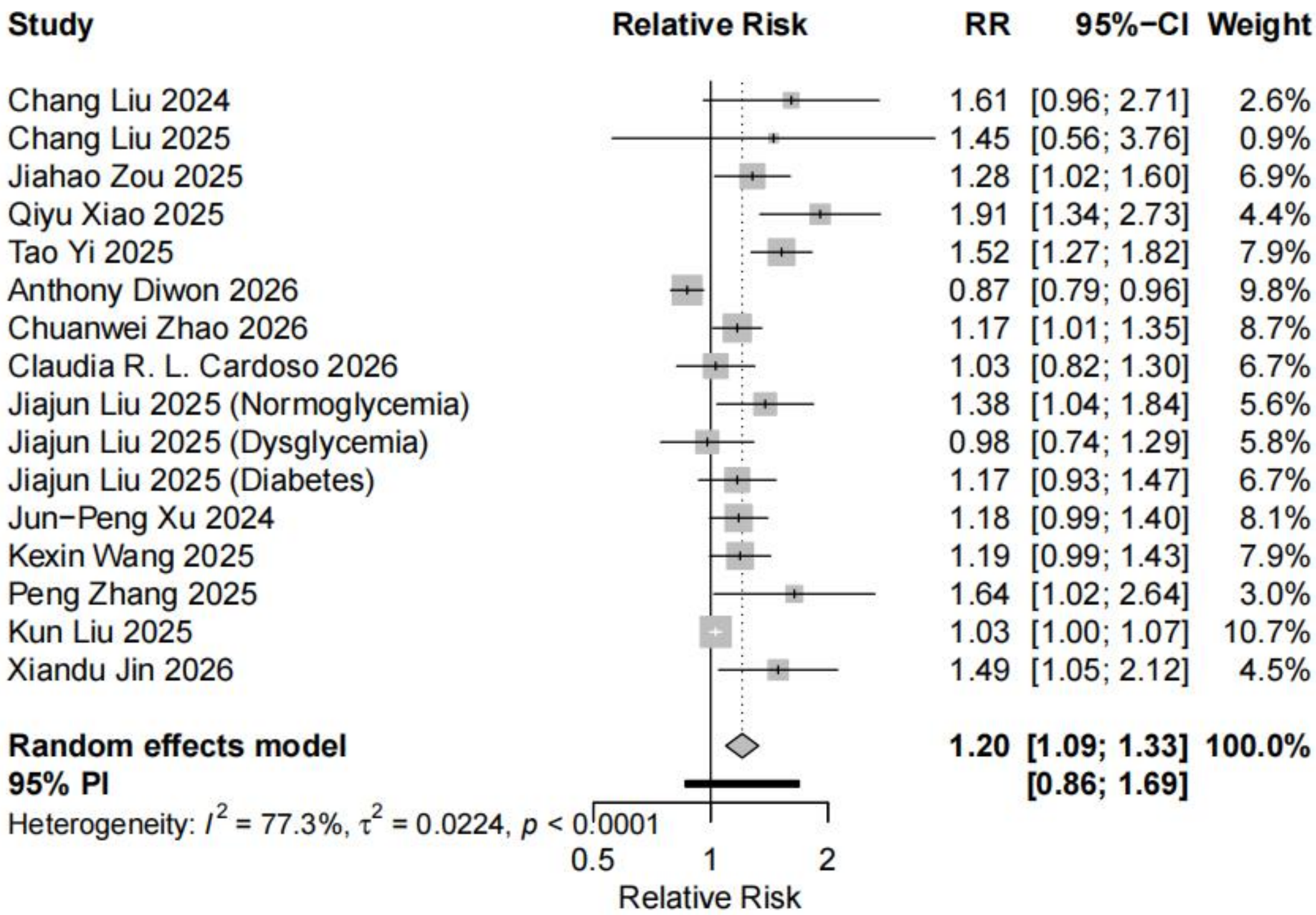

(C) CVD incidence

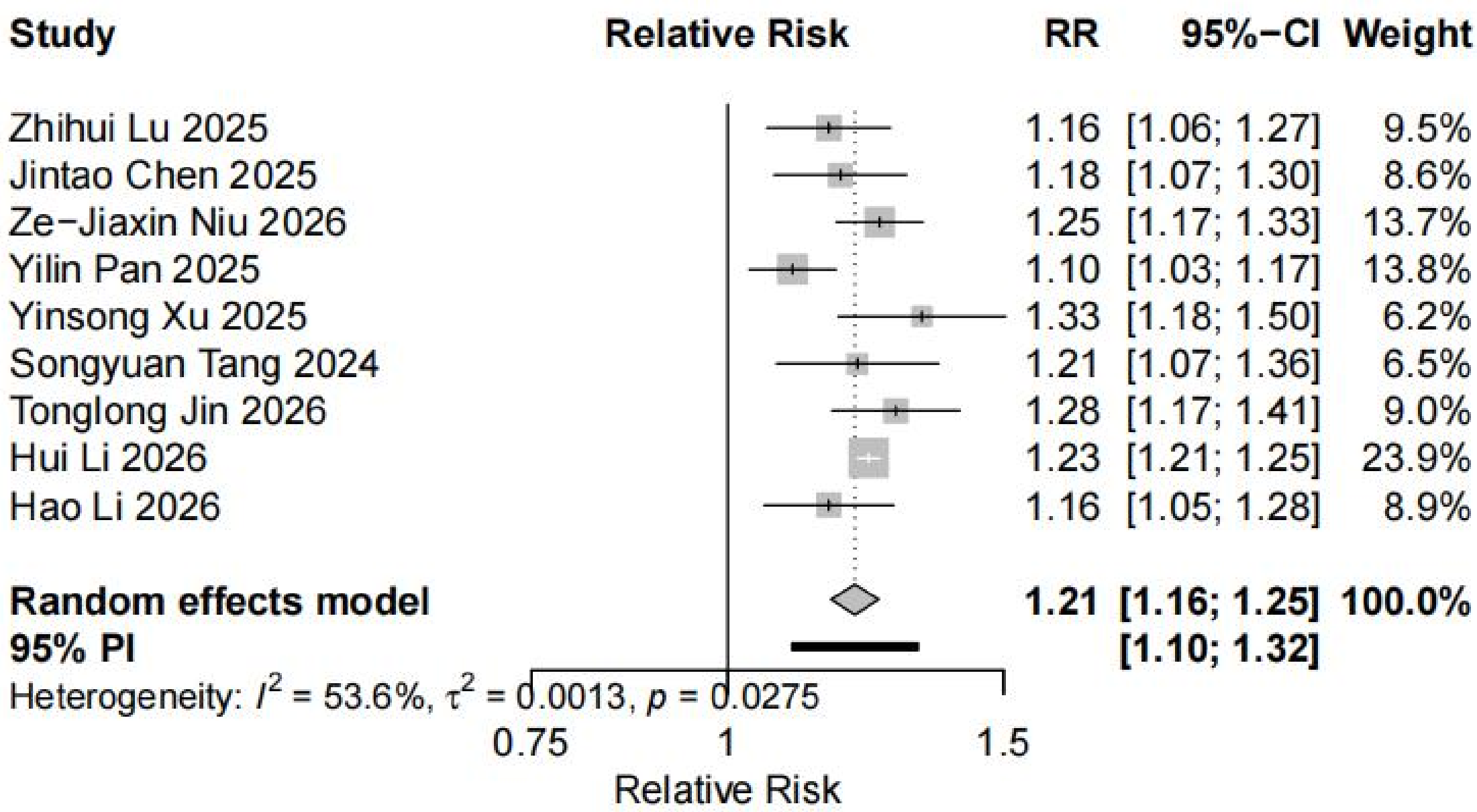

(D) CHD incidence

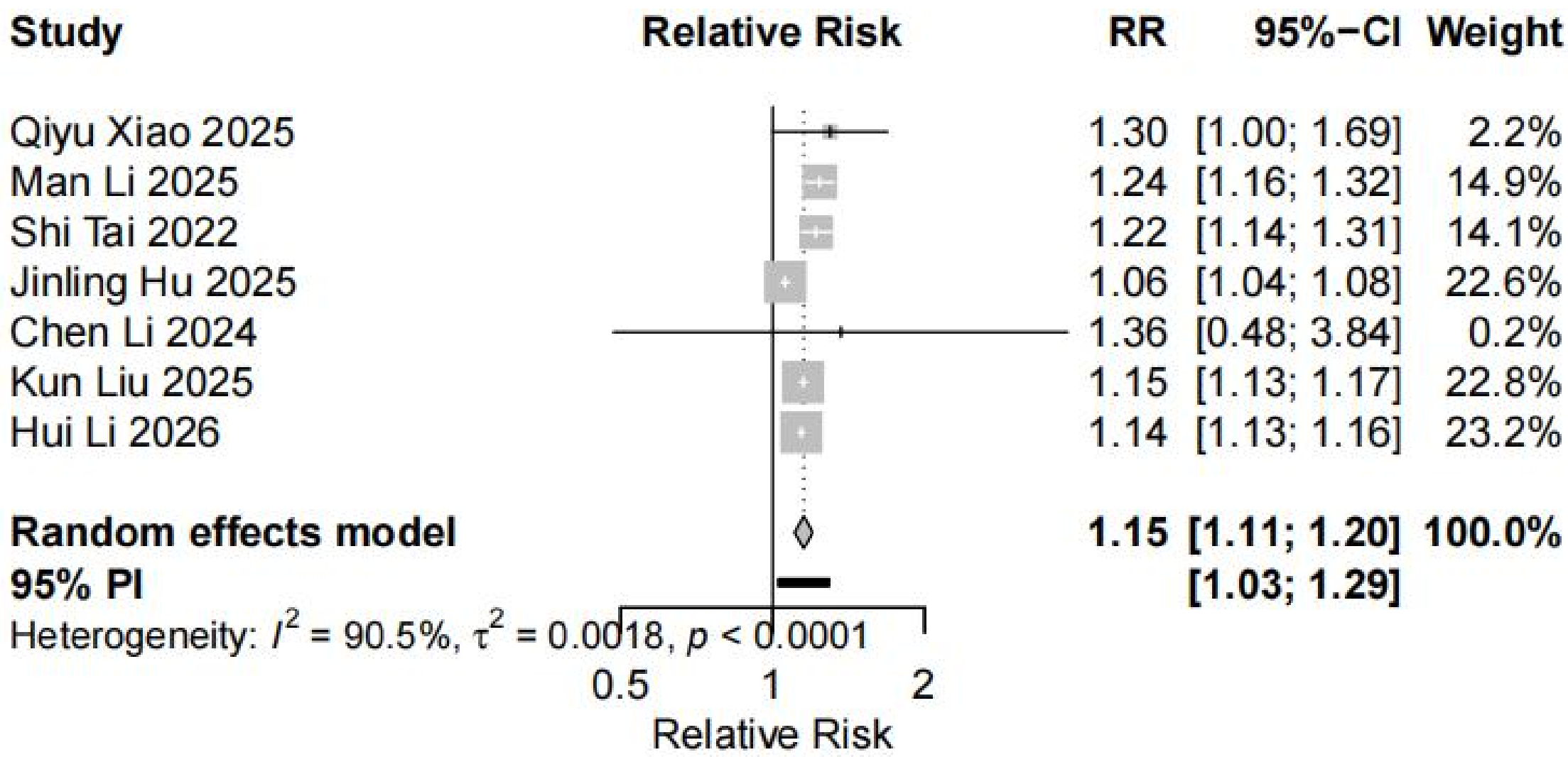

(E) Stroke incidence

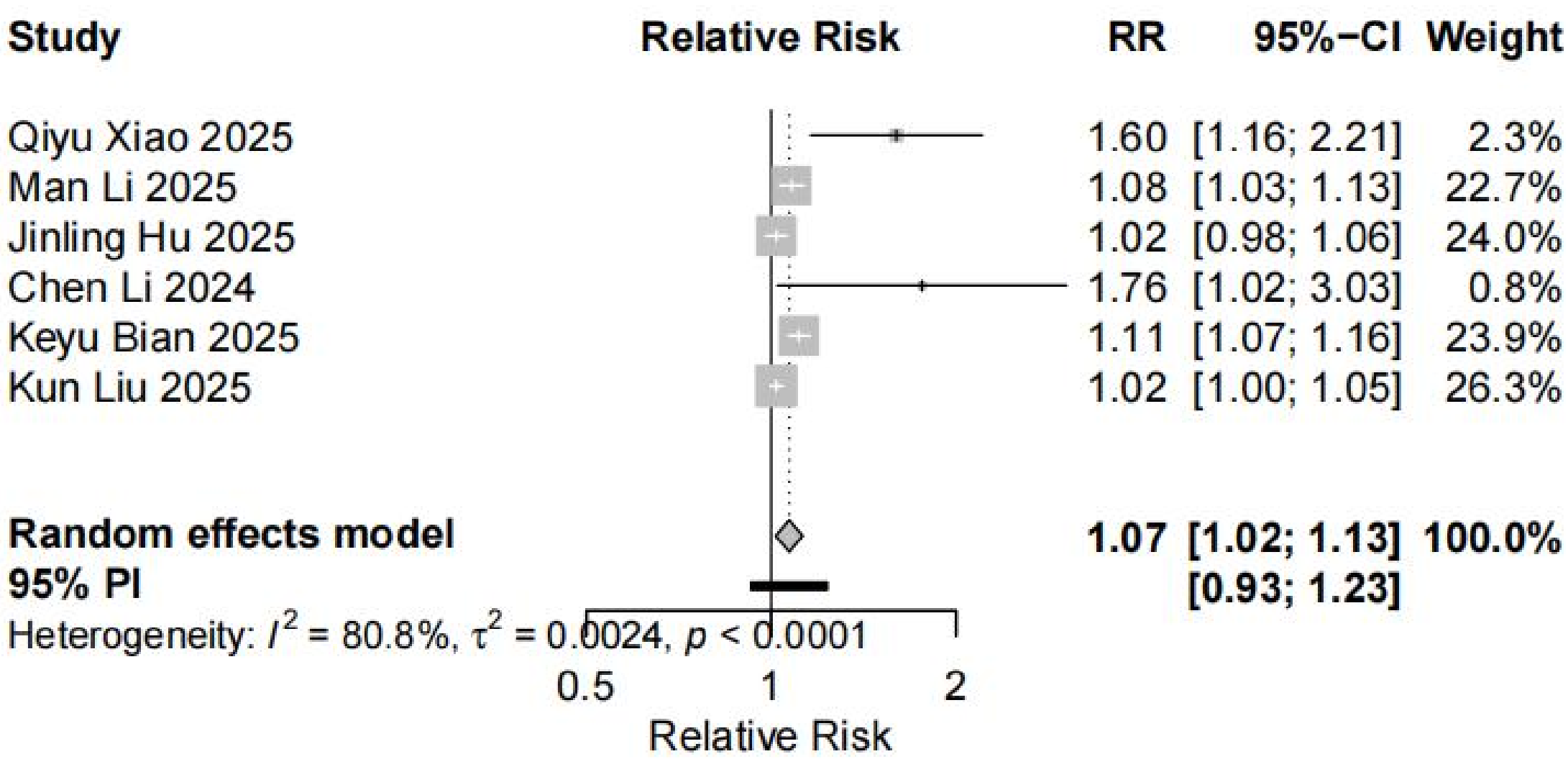

(F) Heart failure incidence

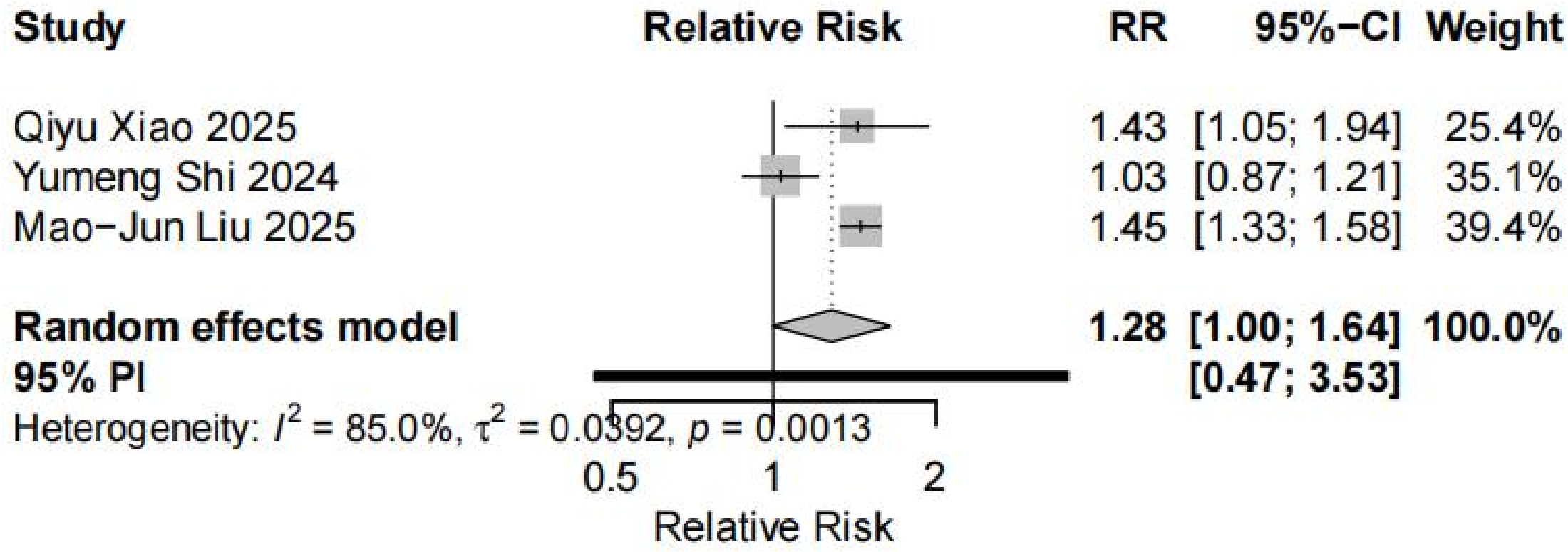

(G) MACE incidence

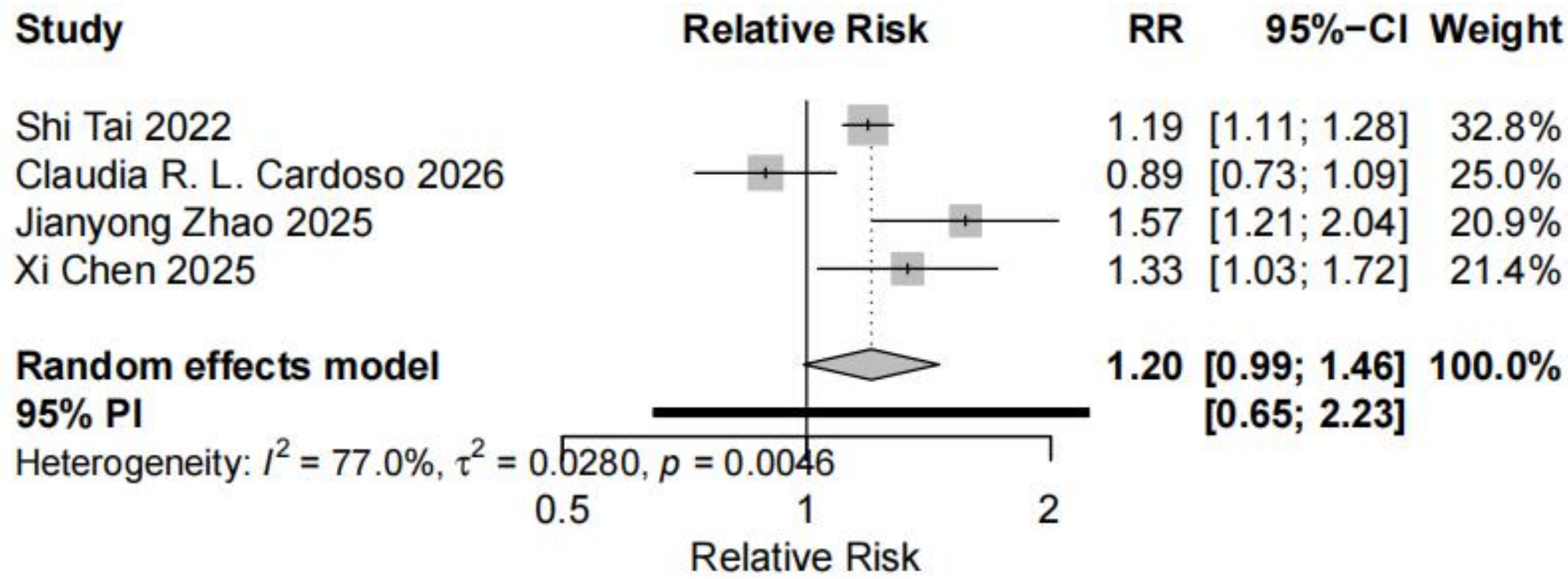

Figure S2. Forest plot of the association between TyG-BMI index (per 1-SD increase) and risk of (A) all-cause mortality, (B) CVD mortality, (C) CVD incidence, (D) CHD incidence and (E) stroke incidence. TyG-BMI, Triglyceride Glucose-Body Mass; SD, Standard Deviation; CVD, Cardiovascular Disease; CHD, Coronary Heart Disease.

(A)All-cause mortality

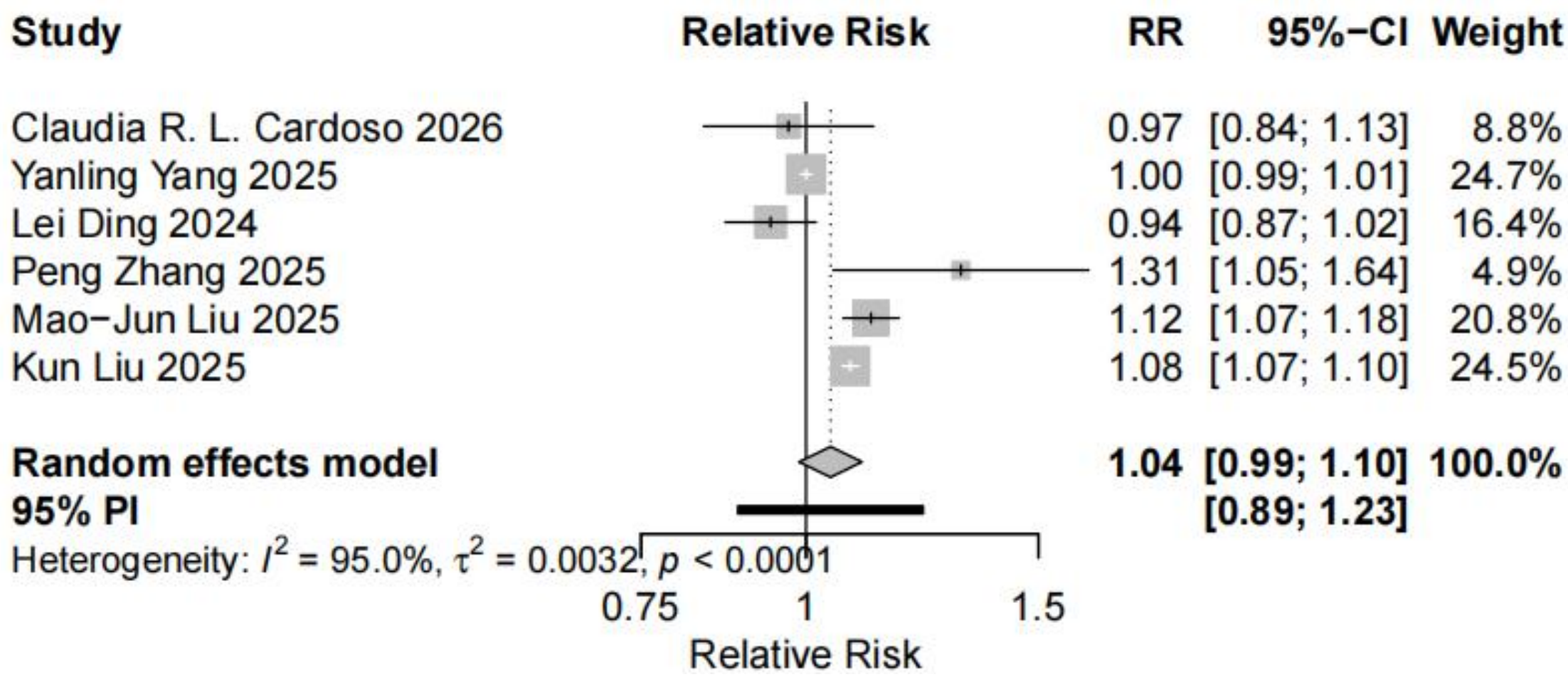

(B)CVD mortality

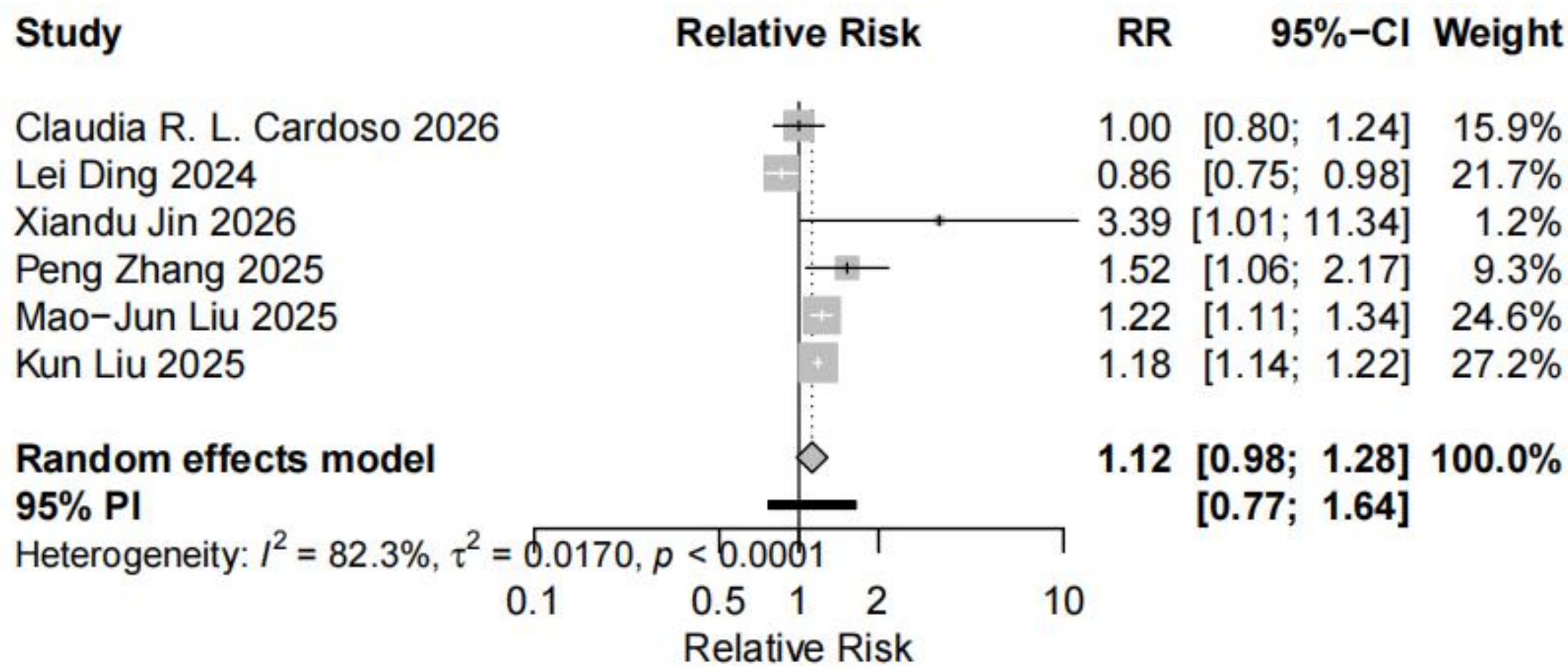

(C)CVD incidence

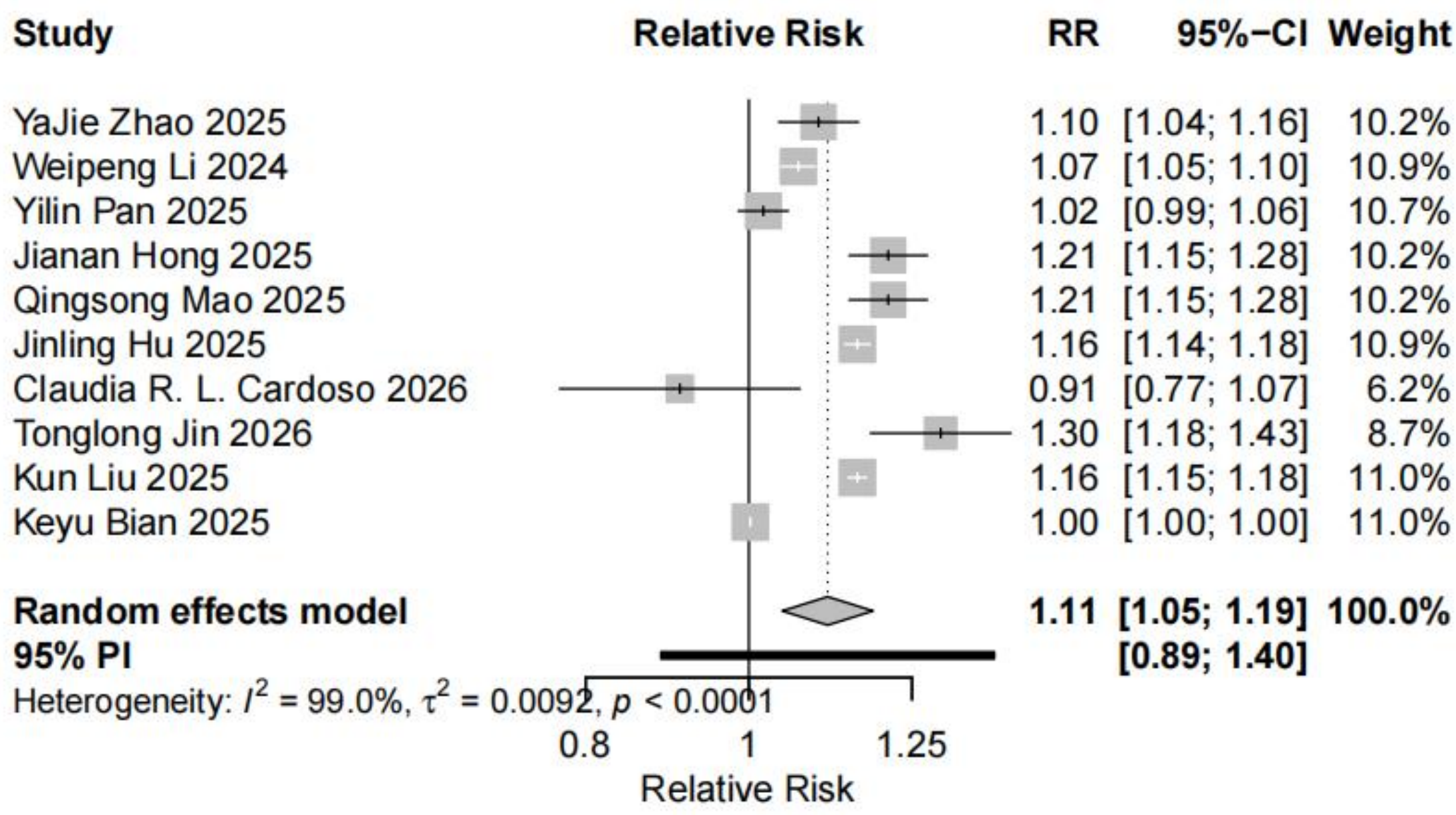

(D)CHD incidence

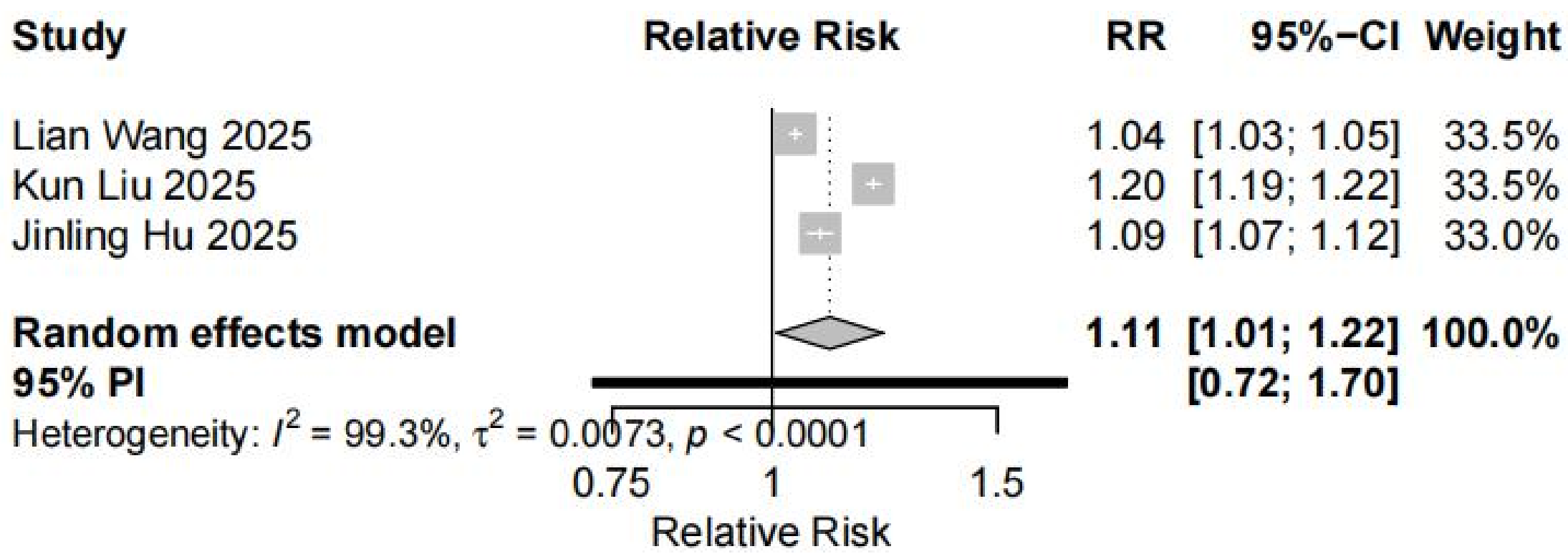

(E)Stroke incidence

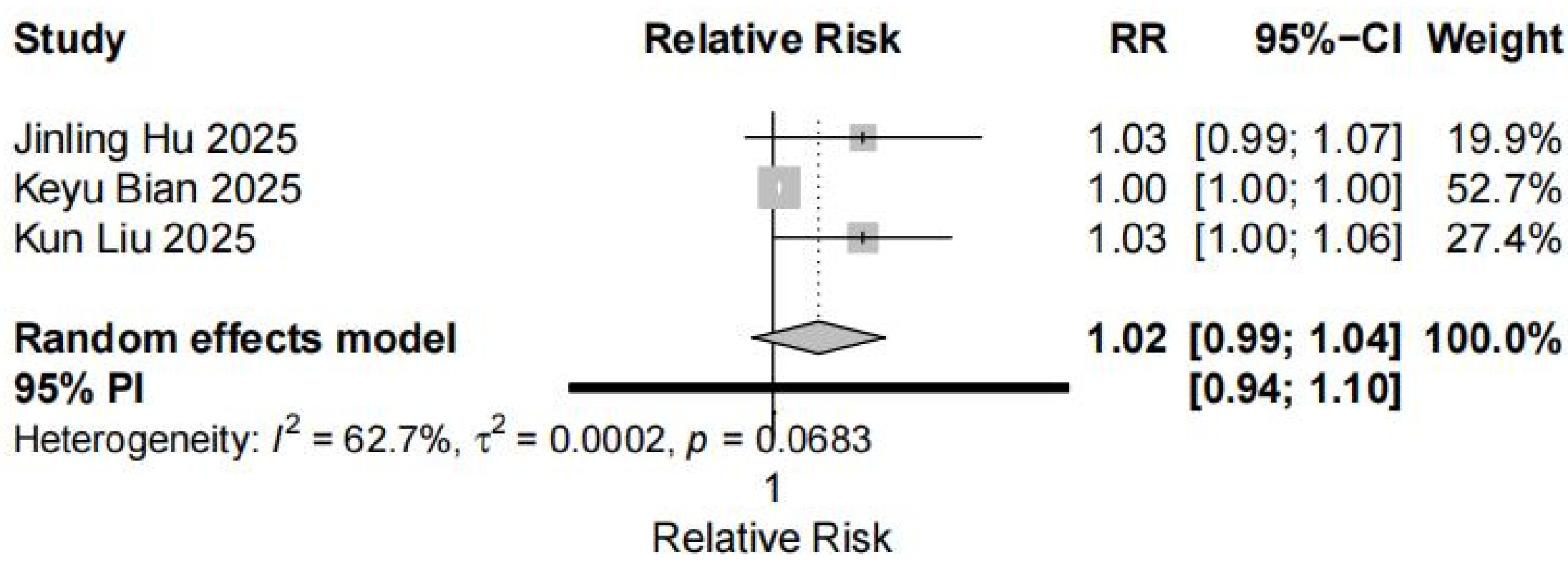

Figure S3. Forest plot of the association between TyG-WC index (per 1-SD increase) and risk of (A) all-cause mortality, (B) CVD mortality, (C) CVD incidence, (D) CHD incidence, and (E) stroke incidence. TyG-WC, Triglyceride-Glucose-Waist Circumference; SD, Standard Deviation; CVD, Cardiovascular Disease; CHD, Coronary Heart Disease.

(A) All-cause mortality

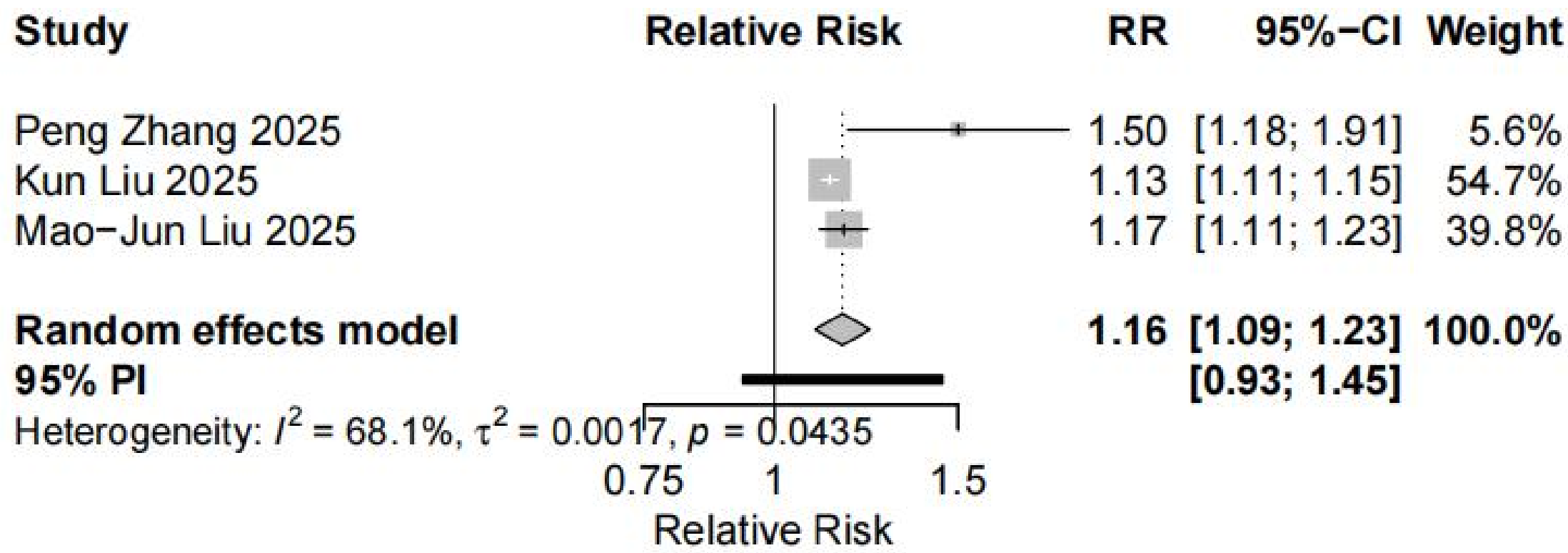

(B) CVD mortality

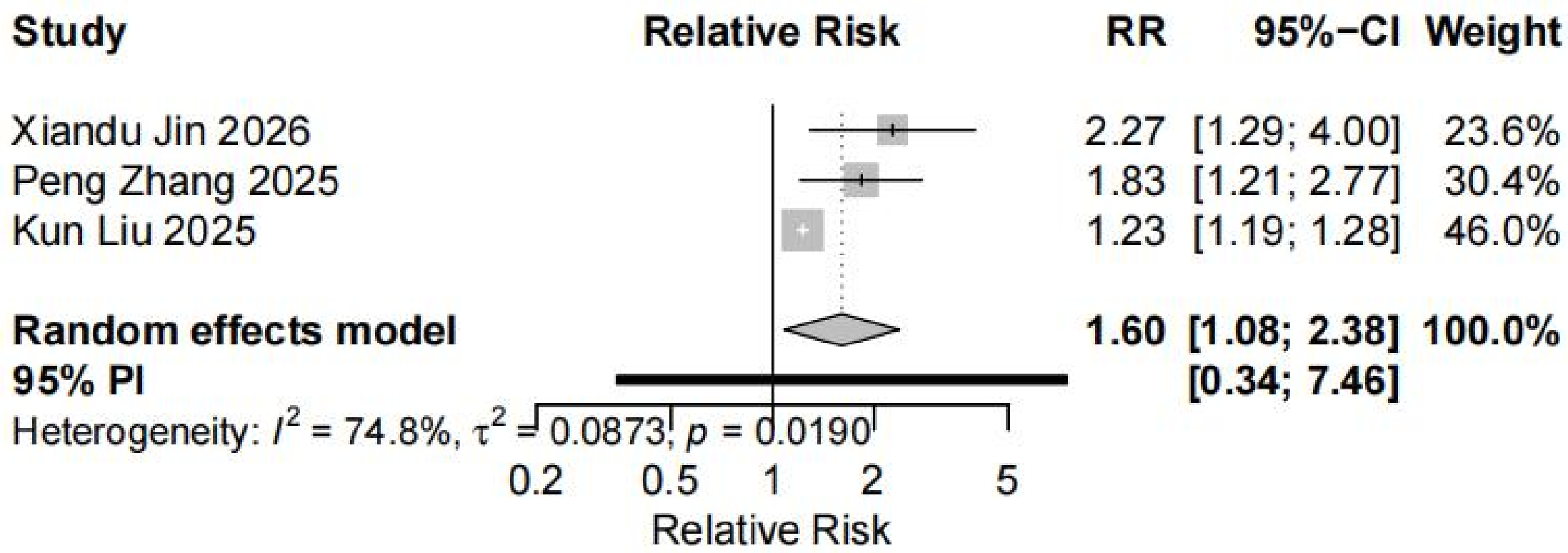

(C) CVD incidence

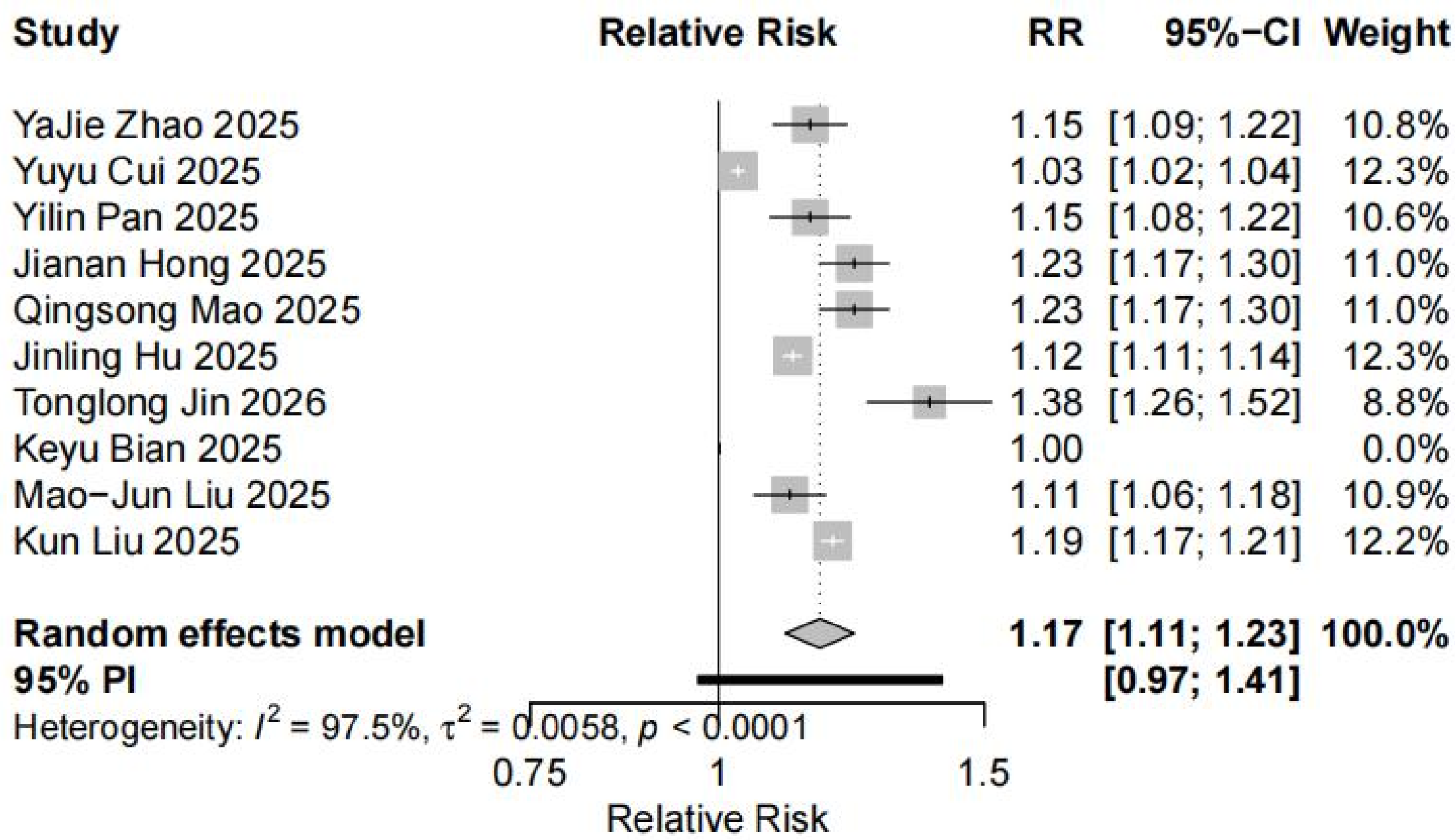

(D) CHD incidence

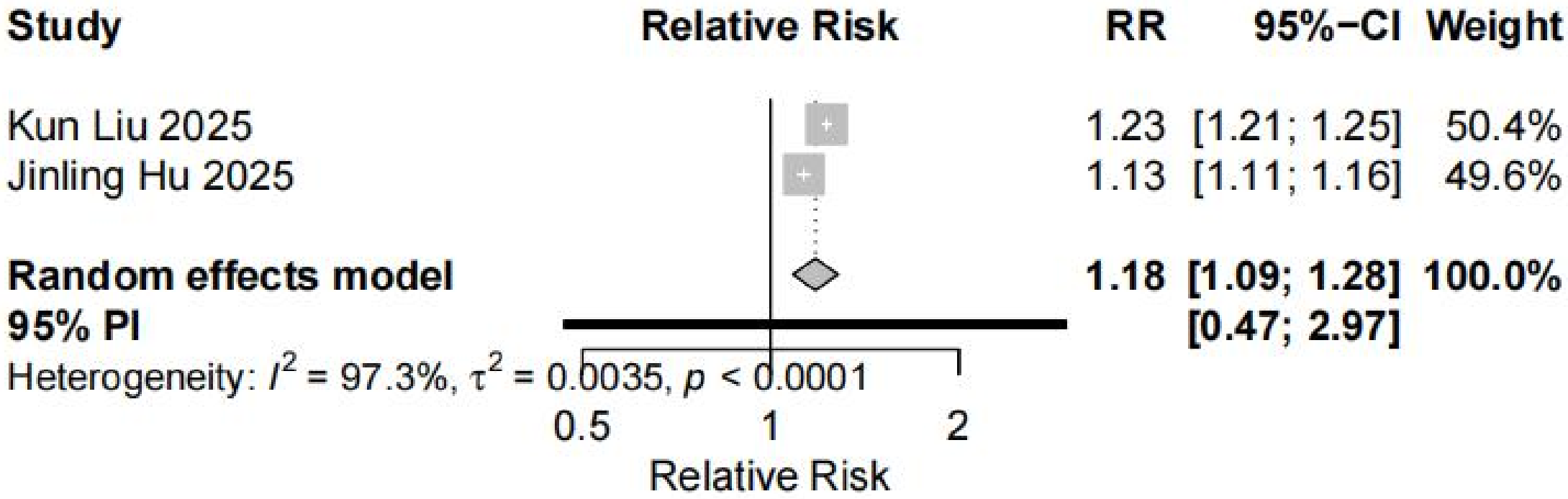

(E) Stroke incidence

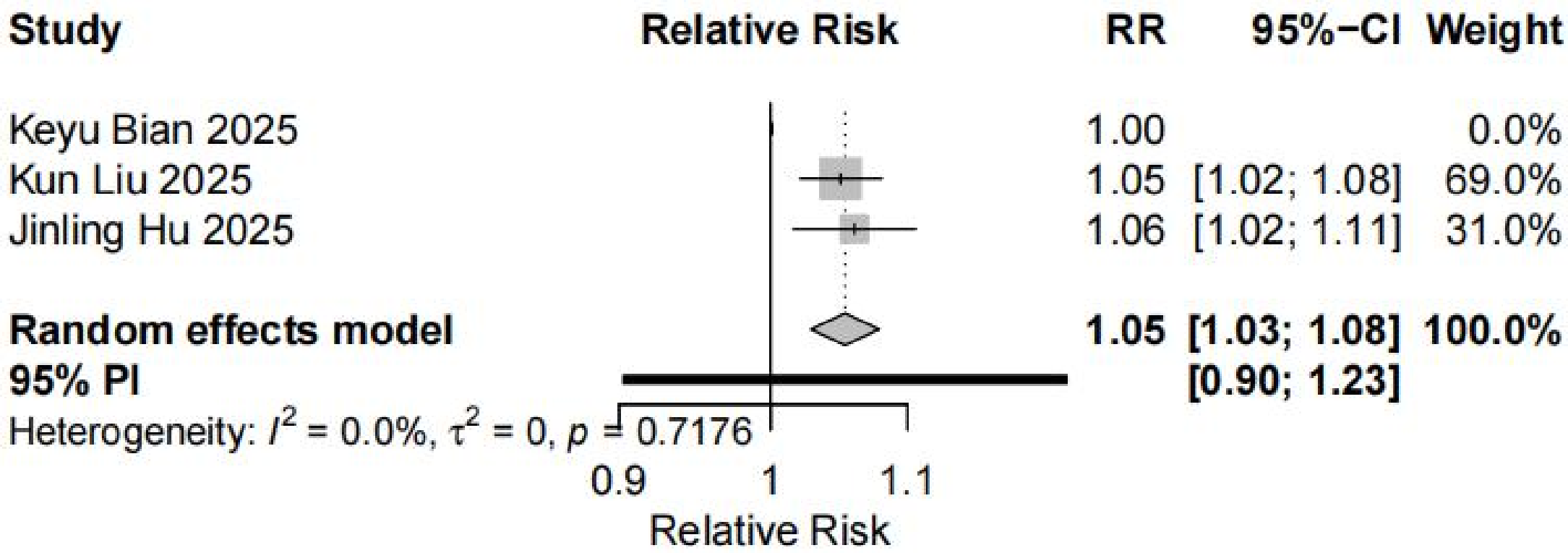

Figure S4. Forest plot of the association between TyG-WhtR index (per 1-SD increase) and risk of (A) all-cause mortality, (B) CVD mortality, (C) CVD incidence, (D) CHD incidence, and (E) stroke incidence. TyG-WHtR, Triglyceride-Glucose Waist-to-Height Ratio; SD, Standard Deviation; CVD, Cardiovascular Disease; CHD, Coronary Heart Disease.

(F) All-cause mortality

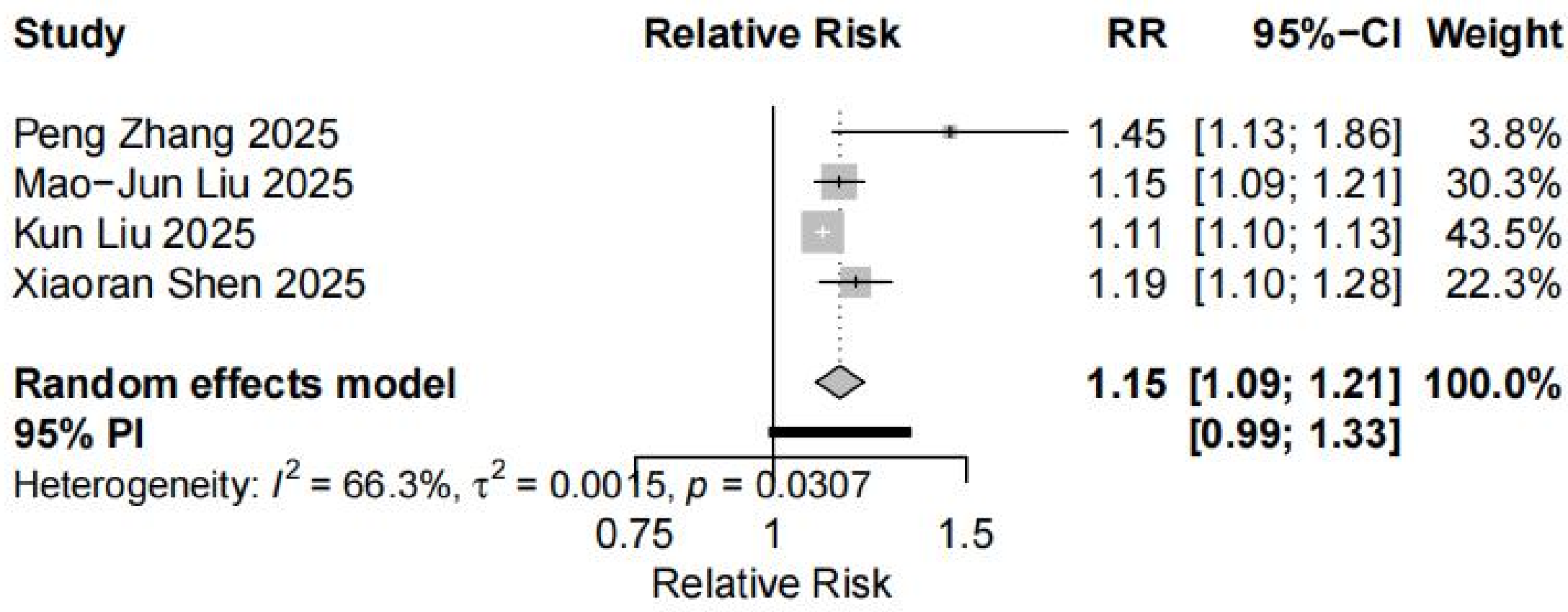

(G) CVD mortality

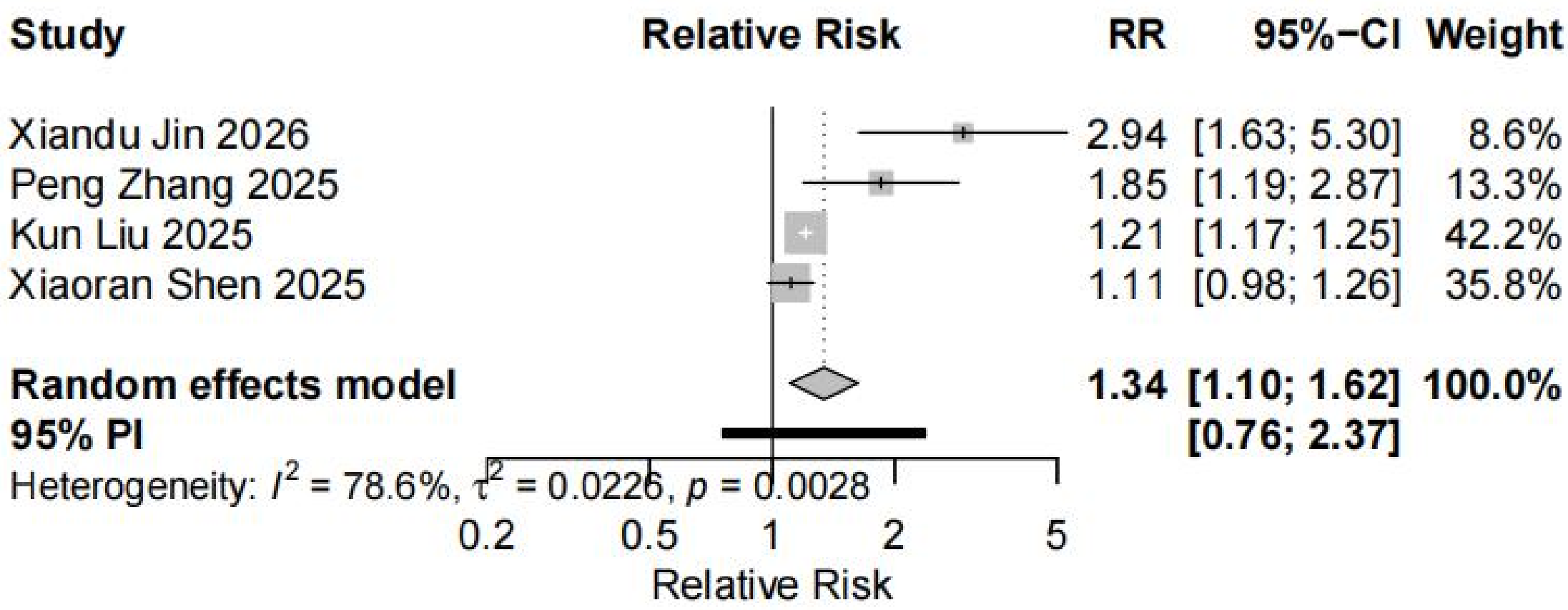

(H) CVD incidence

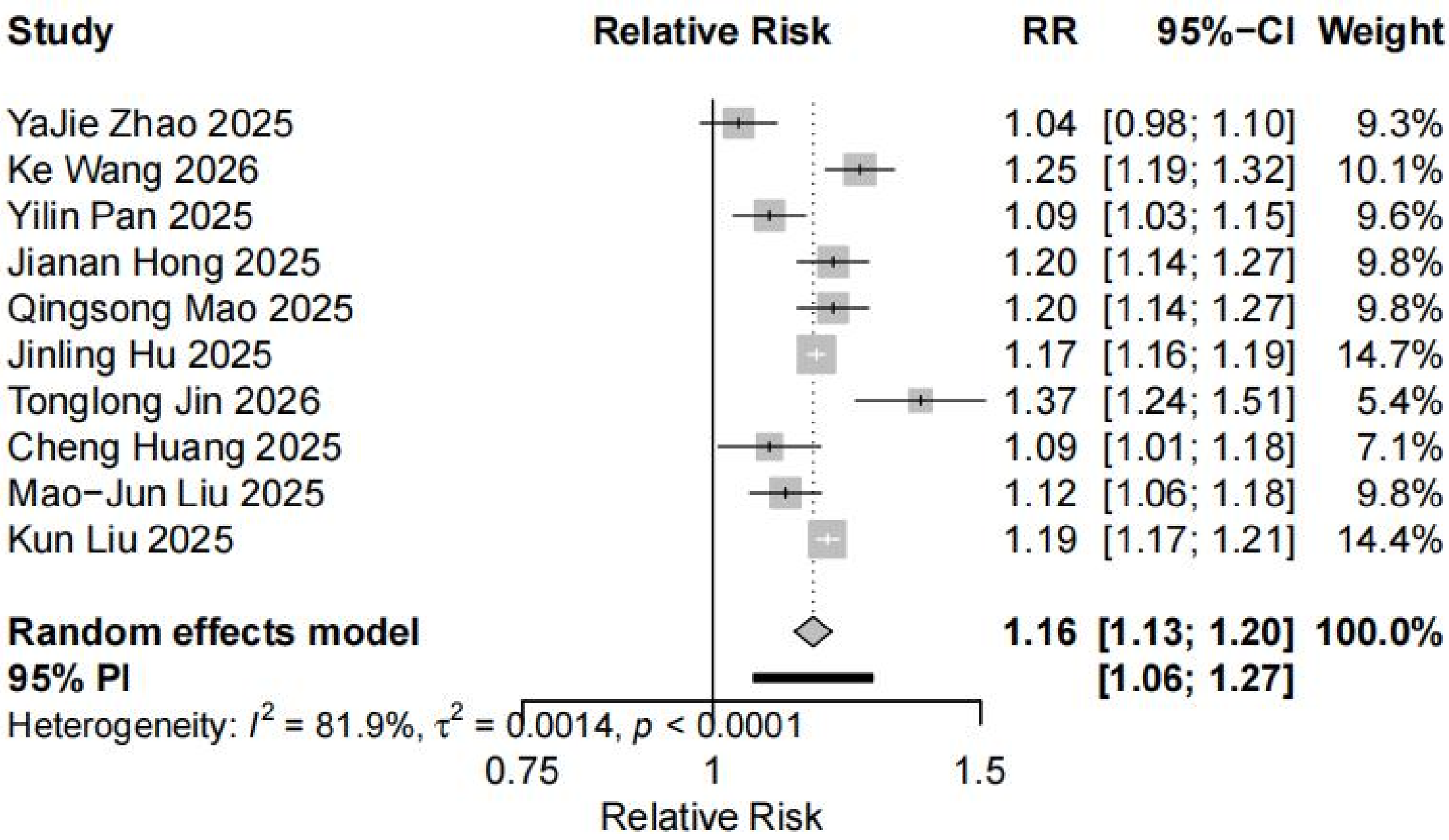

(I) CHD incidence

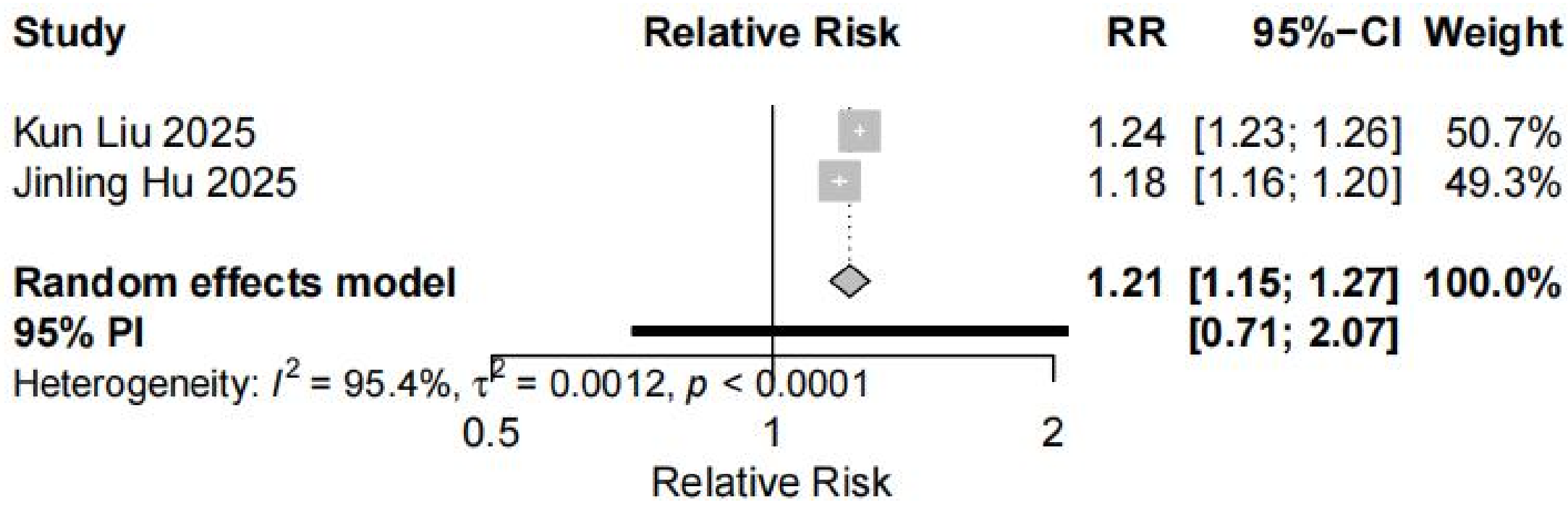

(J) Stroke incidence

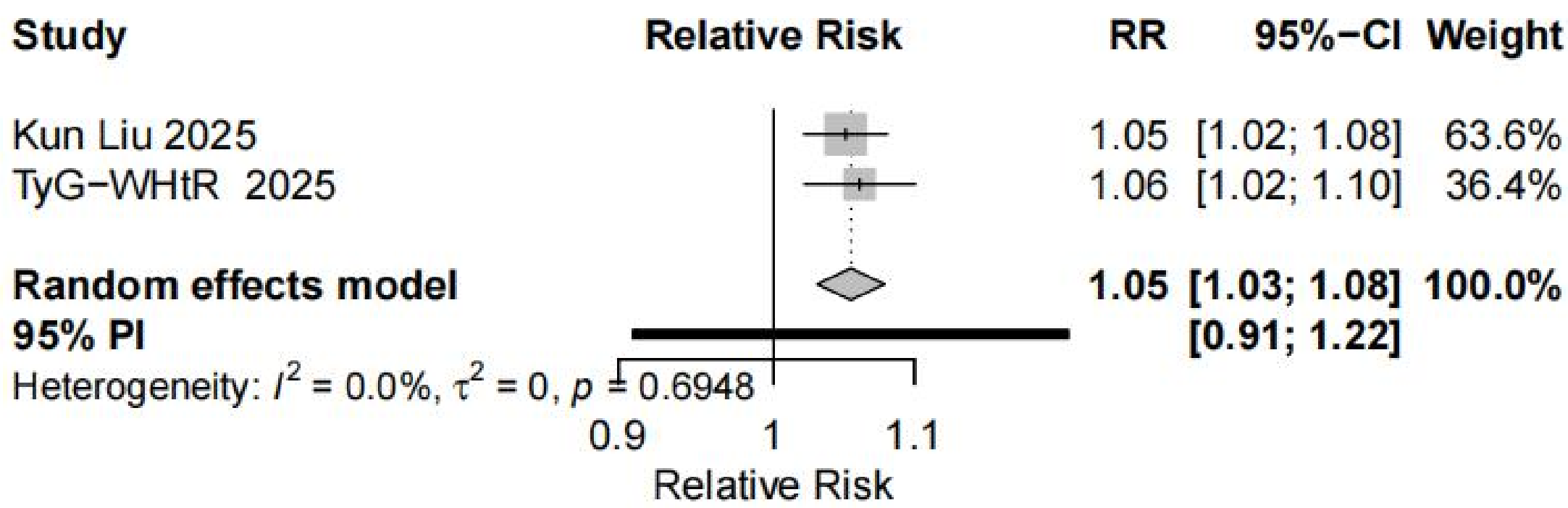

Figure S5. Forest plot of the association between CTI index (per 1-SD increase) and risk of (A) all-cause mortality, (B) CVD incidence, (C) stroke incidence. CTI, C-Reactive Protein-Triglyceride Glucose; SD, Standard Deviation; CVD, Cardiovascular Disease.

(K) All-cause mortality

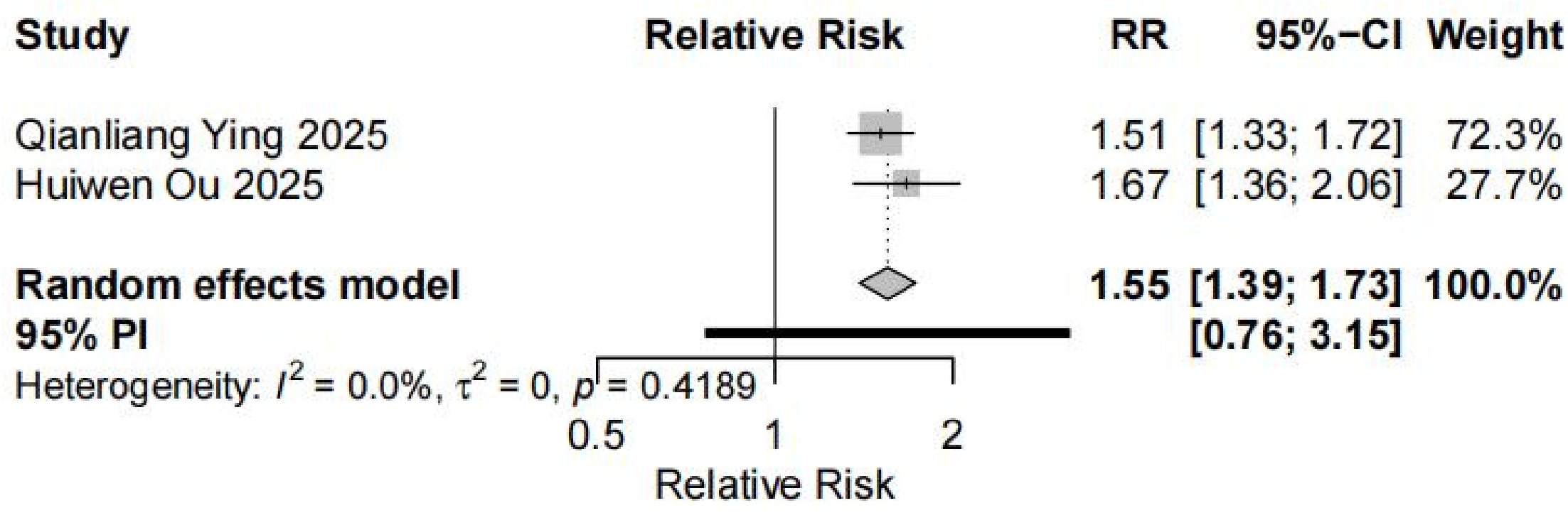

(L) CVD incidence

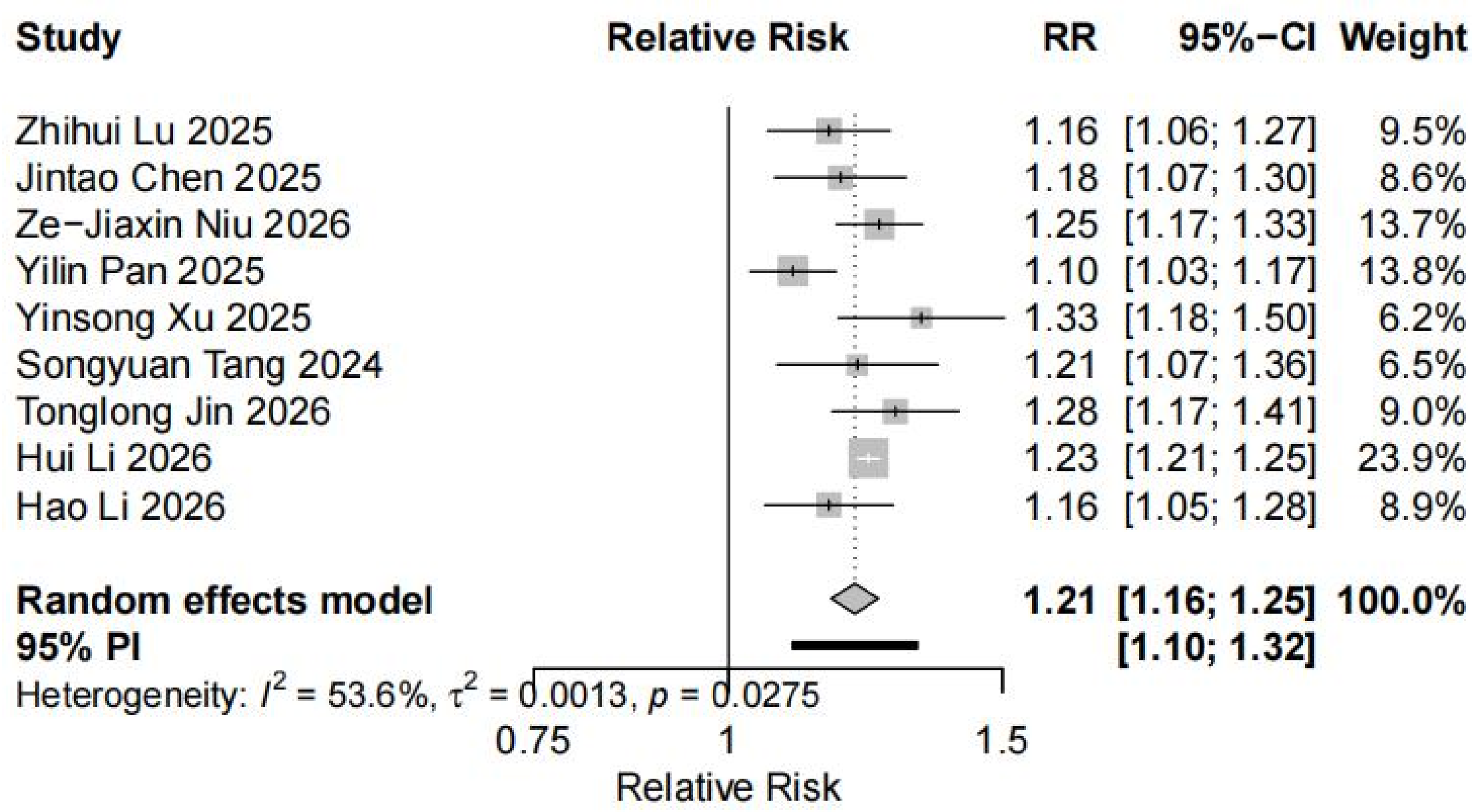

(M) Stroke incidence

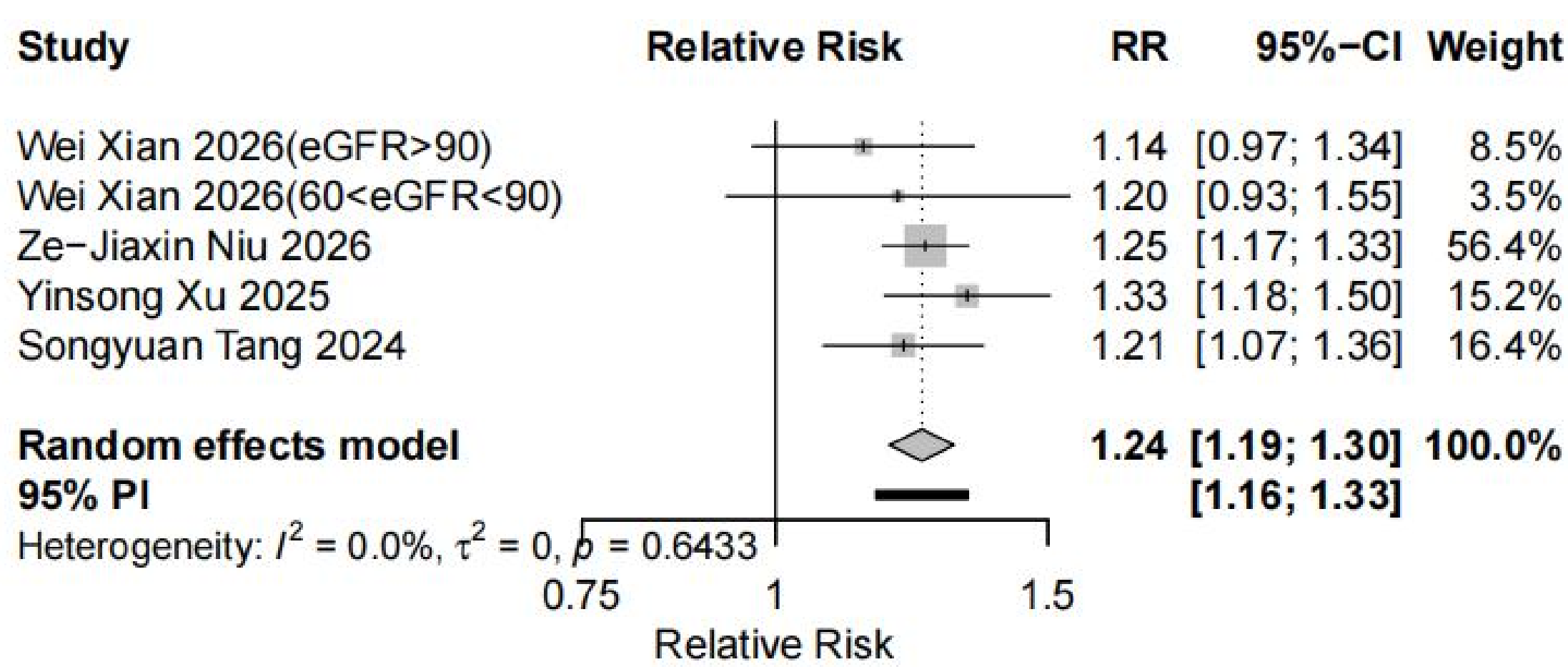

Figure S6. Forest plot of the association between categorized TyG index (highest vs. reference category) and risk of (A) all-cause mortality, (B) CVD mortality, (C) CVD incidence, (D) CHD incidence, (E) stroke incidence, (F) heart failure incidence and (G) MACE incidence. TyG, Triglyceride-glucose; SD, Standard Deviation; CVD, Cardiovascular Disease; CHD, Coronary Heart Disease; MACE, Major Adverse Cardiovascular Event.

(A)All-cause mortality

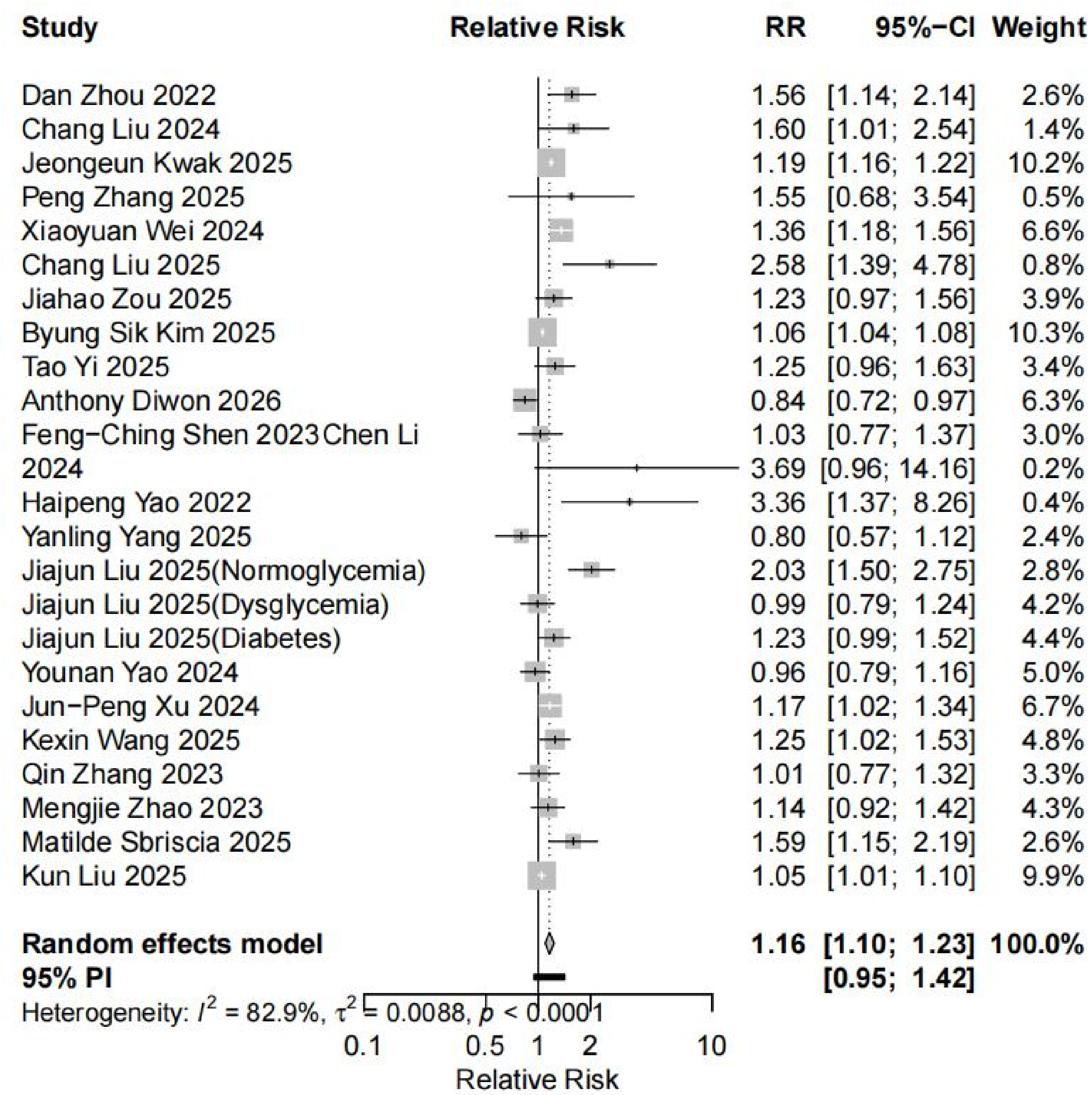

(B)CVD mortality

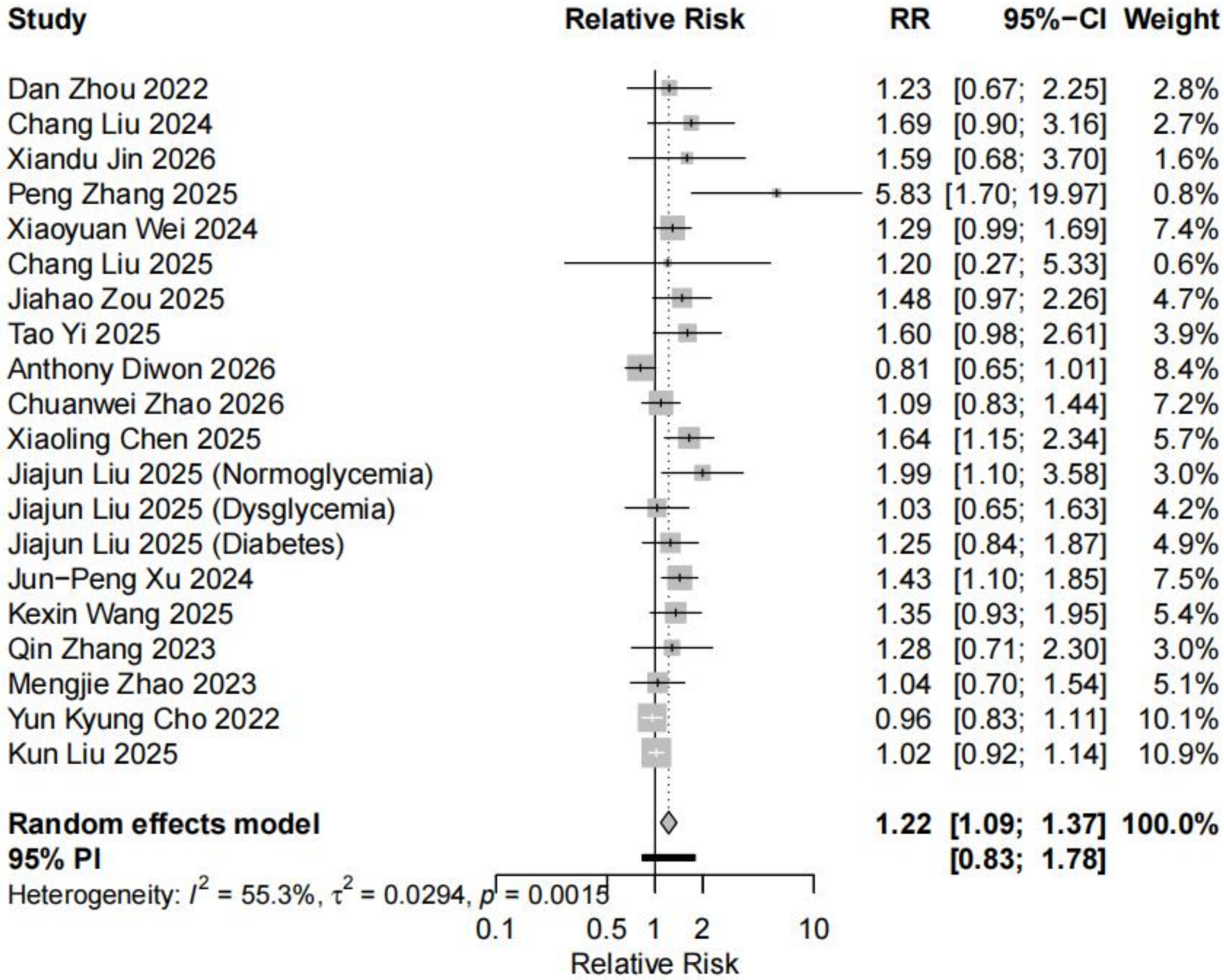

(C)CVD incidence

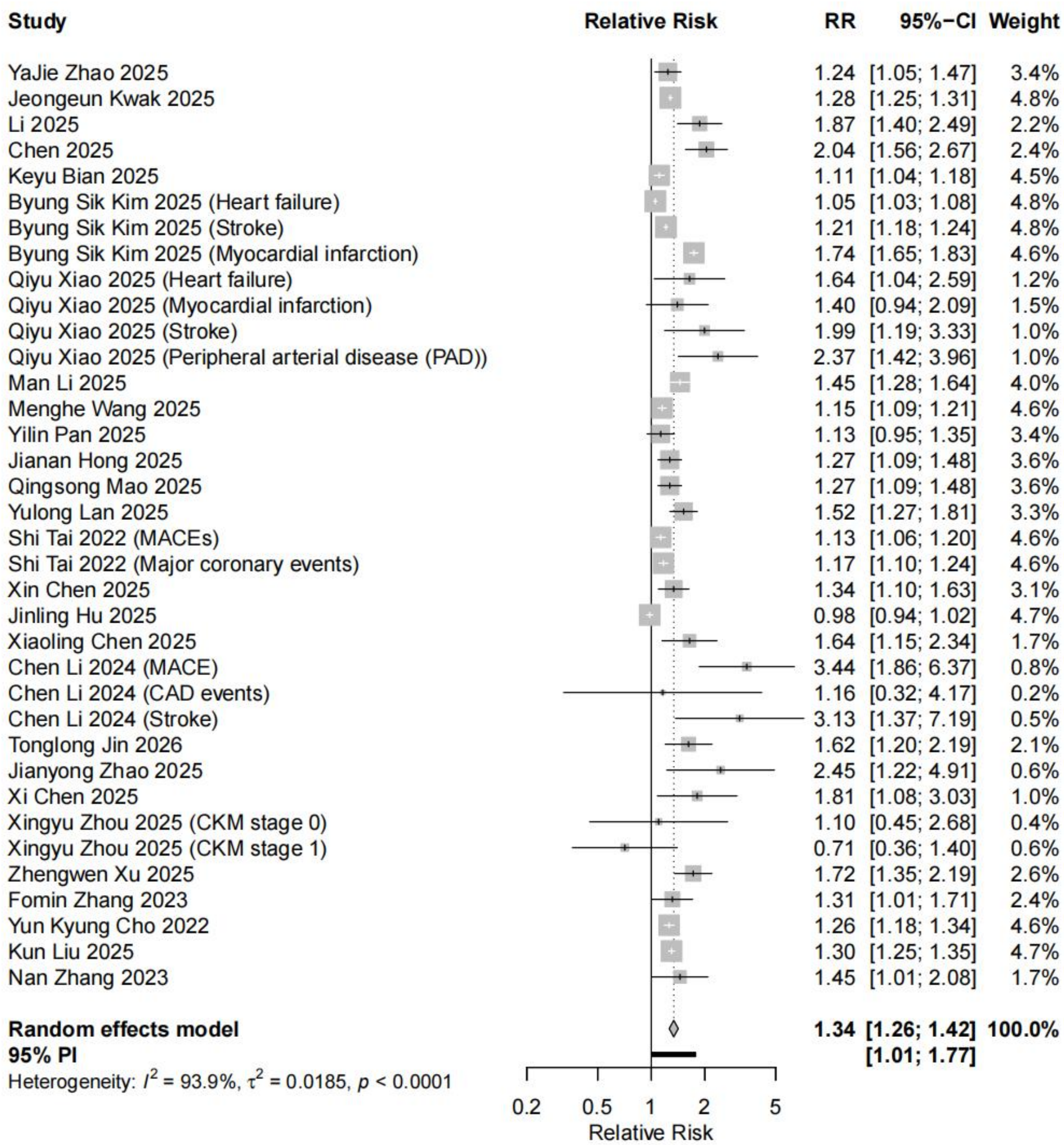

(D)CHD incidence

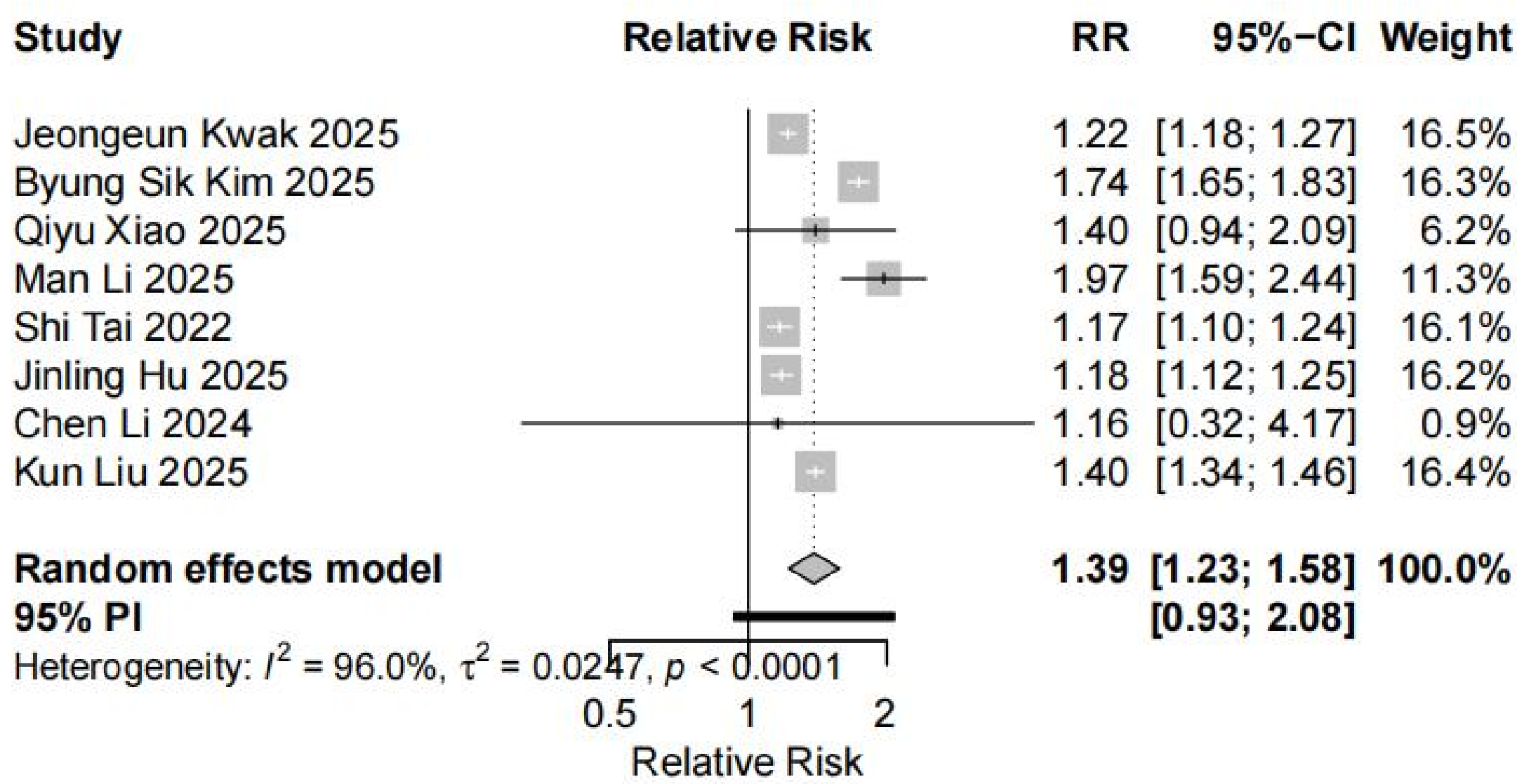

(E)Stroke incidence

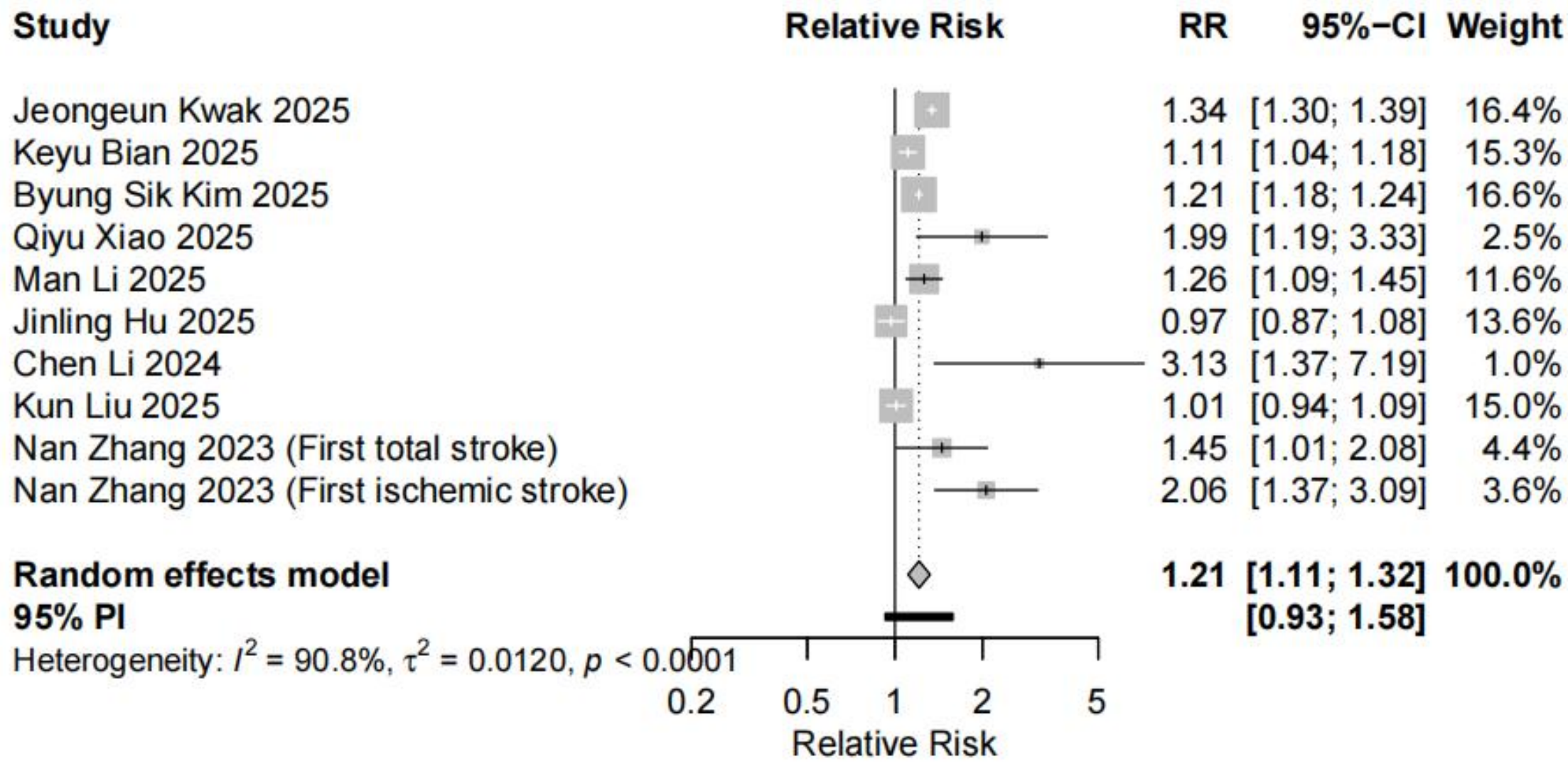

(F)Heart failure incidence

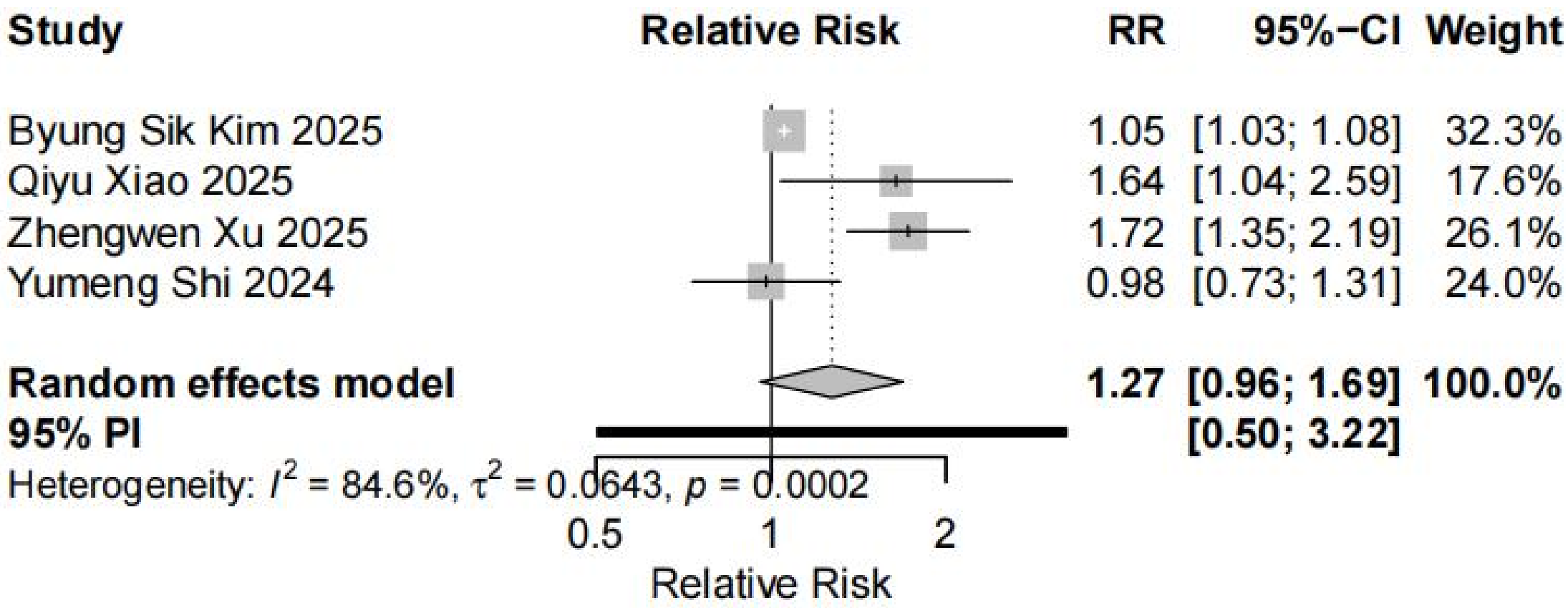

(G)MACE incidence

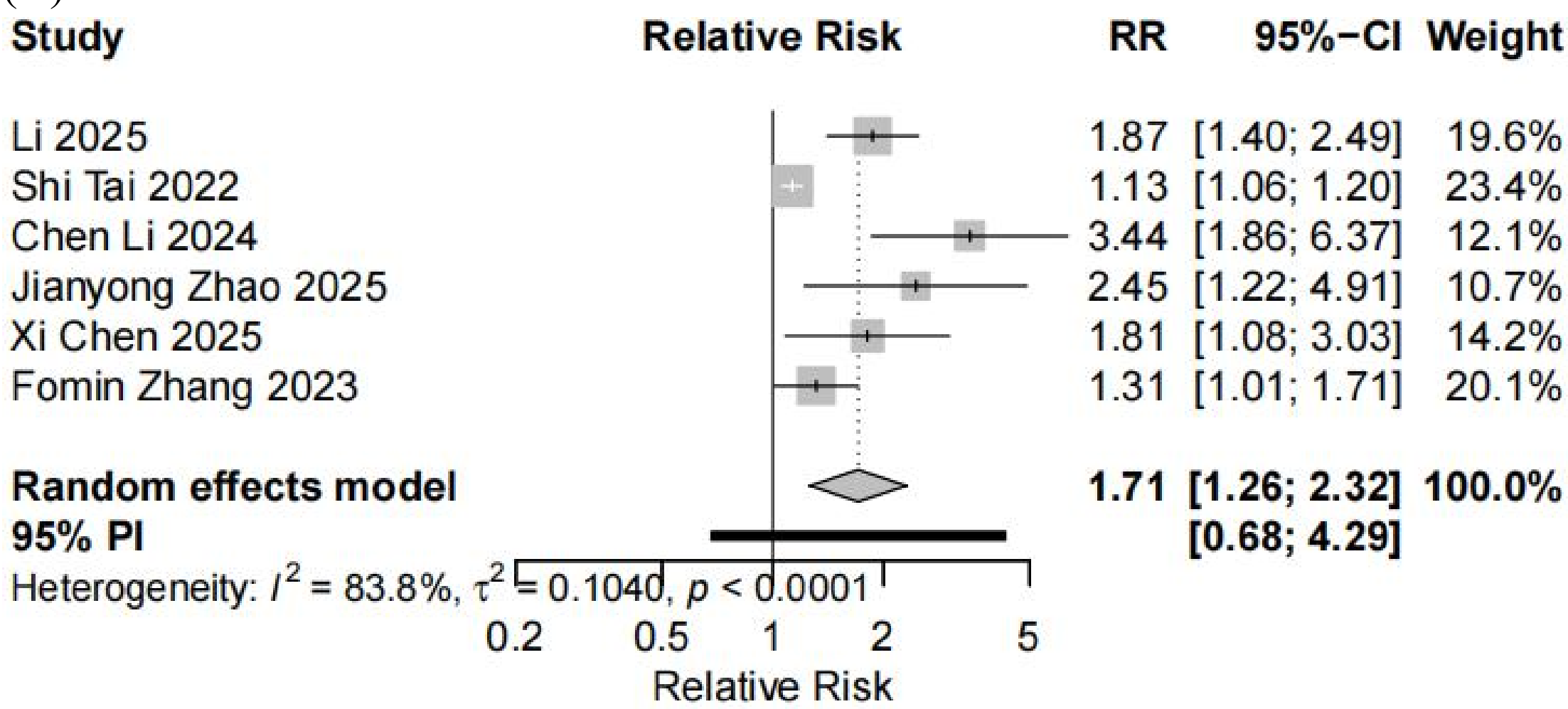

Figure S7. Forest plot of the association between categorized TyG-BMI index (highest vs. reference category) and risk of (A) all-cause mortality, (B) CVD mortality, (C) CVD incidence, (D) CHD incidence and (E) stroke incidence. TyG-BMI, Triglyceride Glucose-Body Mass; SD, Standard Deviation; CVD, Cardiovascular Disease; CHD, Coronary Heart Disease.

(A)All-cause mortality

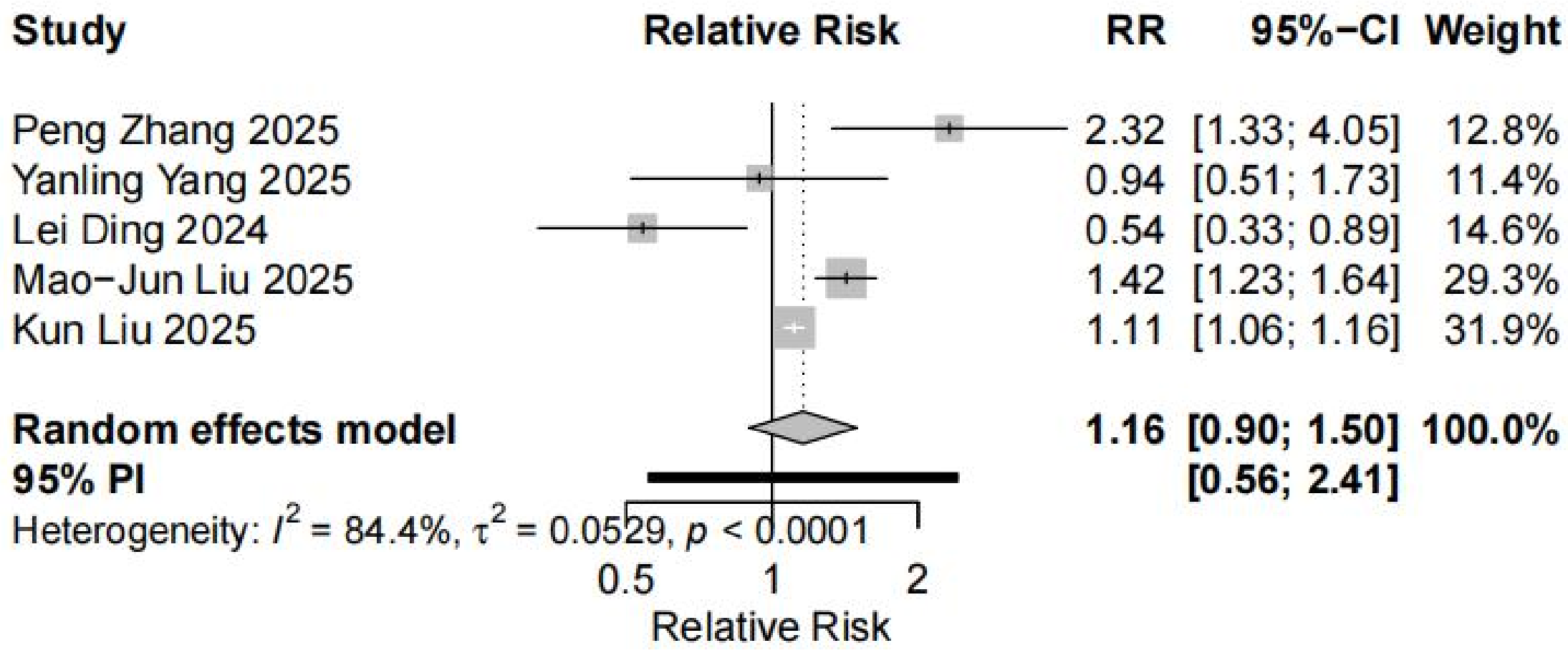

(B)CVD mortality

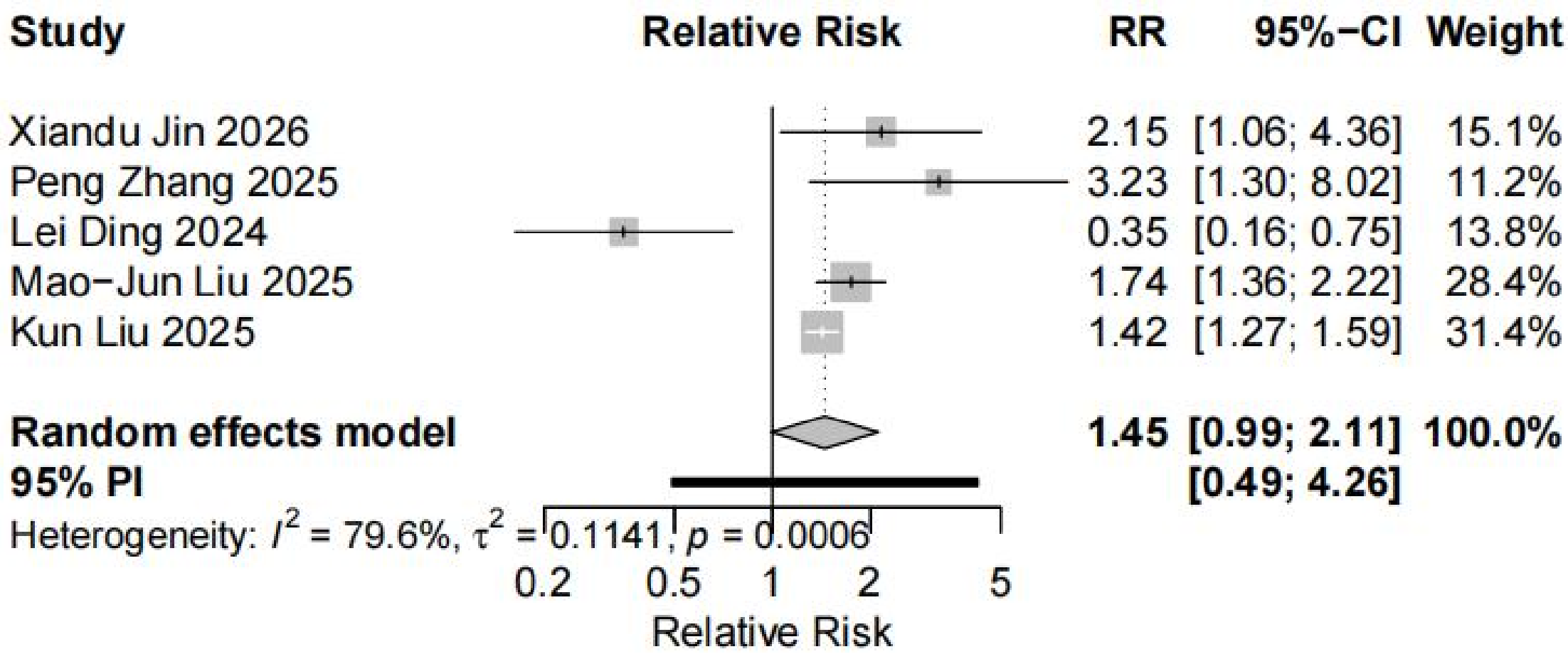

(C)CVD incidence

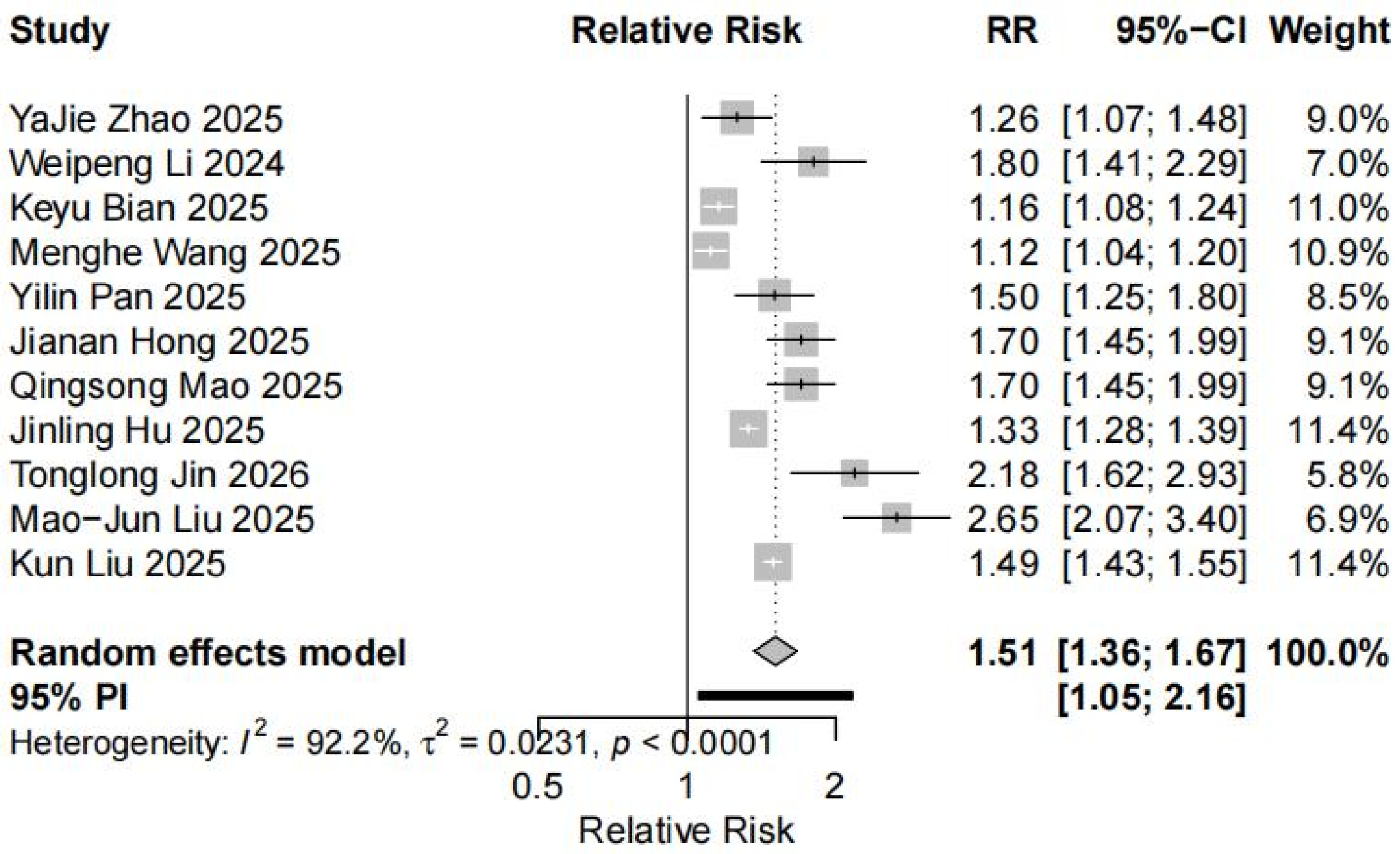

(D)CHD incidence

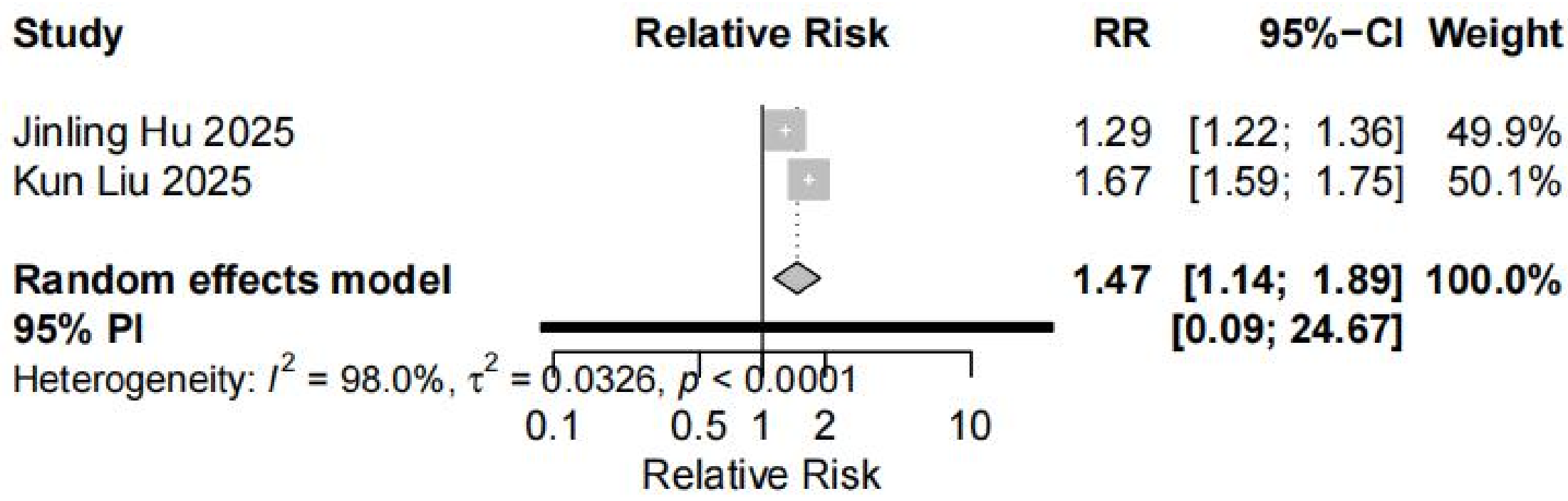

(E)Stroke incidence

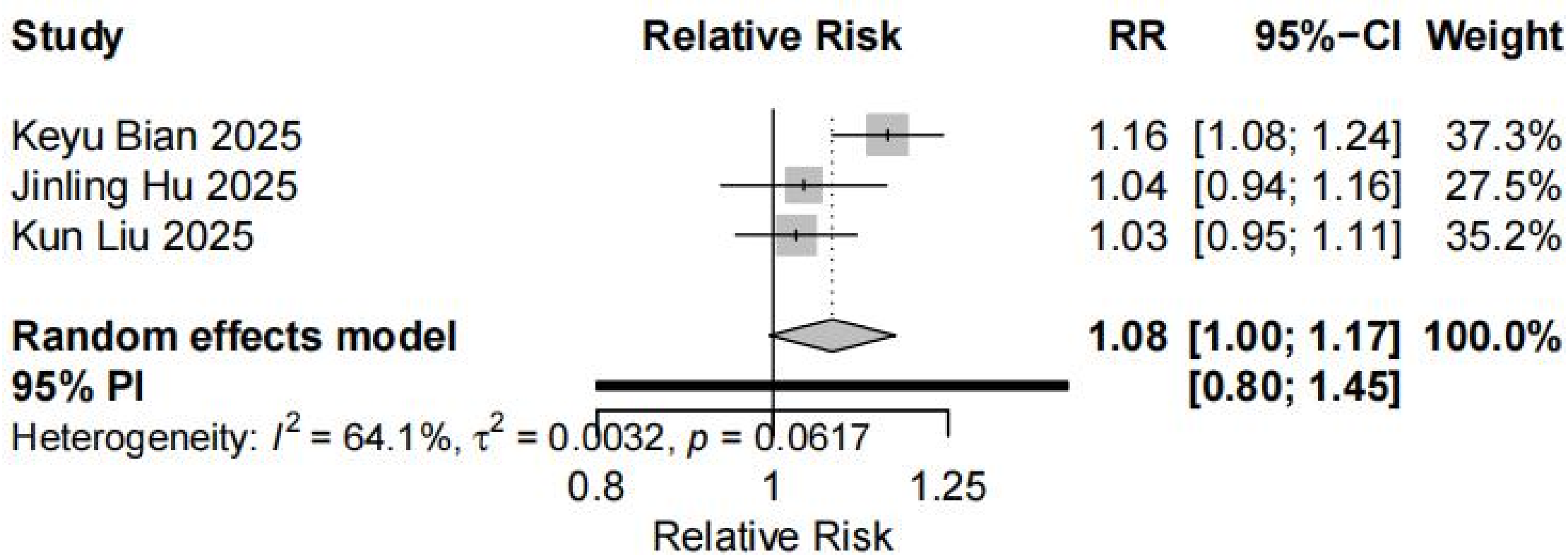

Figure S8. Forest plot of the association between categorized TyG-WC index (highest vs. reference category) and risk of (A) all-cause mortality, (B) CVD mortality, (C) CVD incidence, (D) CHD incidence and (E) stroke incidence. TyG-WC, Triglyceride-Glucose-Waist Circumference; SD, Standard Deviation; CVD, Cardiovascular Disease; CHD, Coronary Heart Disease.

(A)All-cause mortality

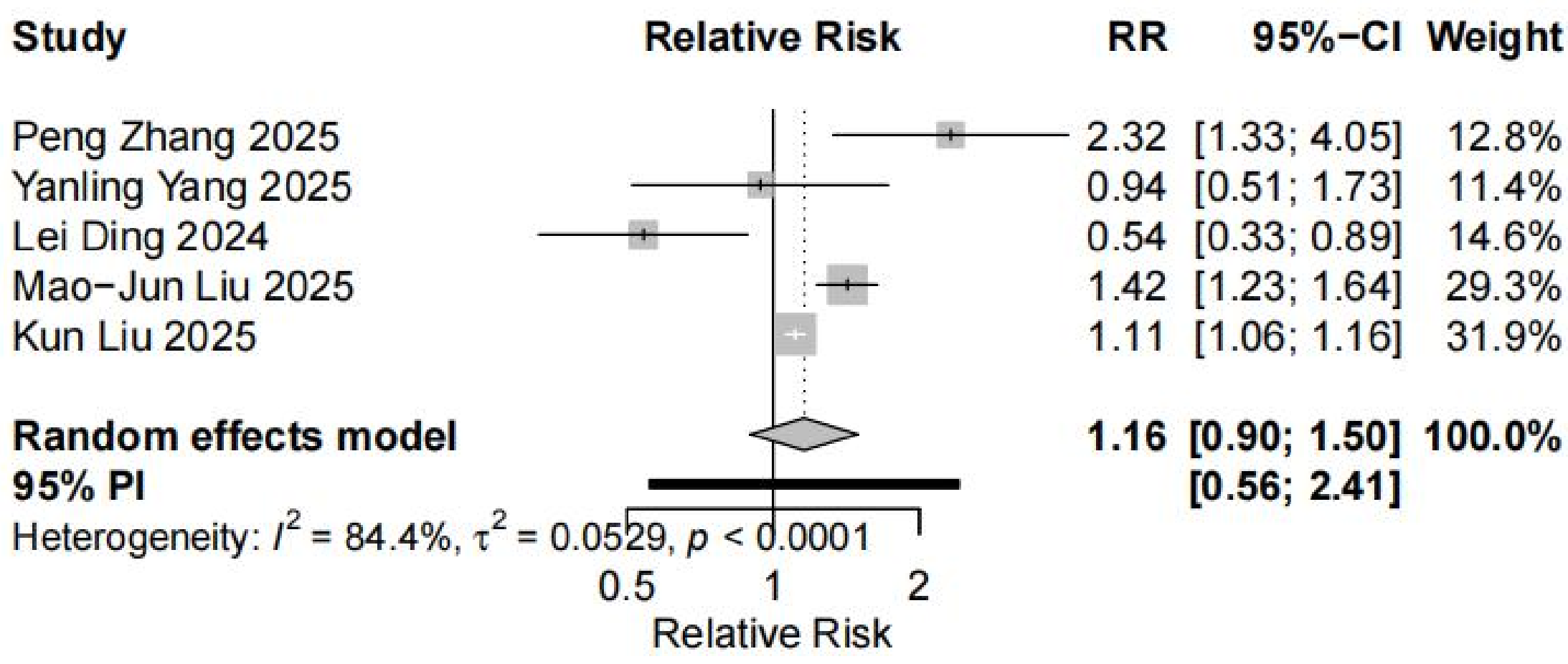

(B)CVD mortality

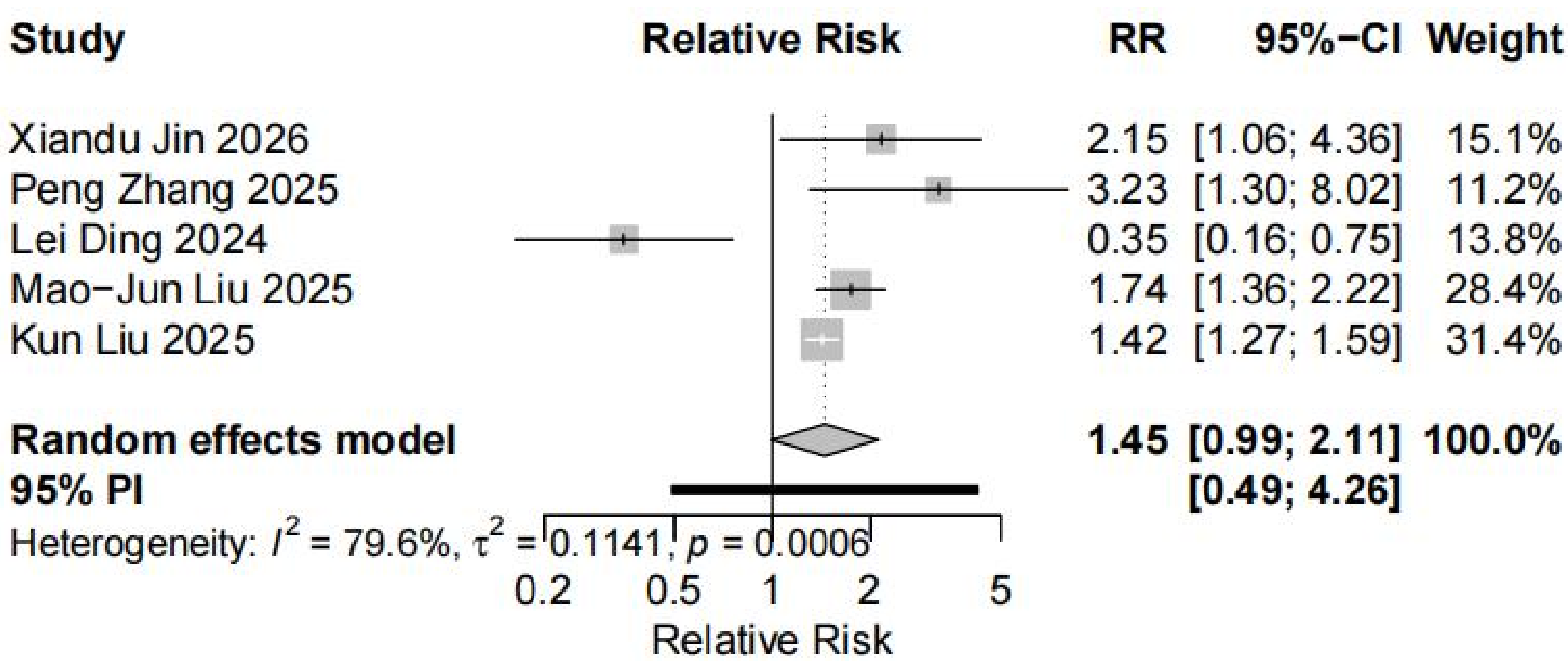

(C)CVD incidence

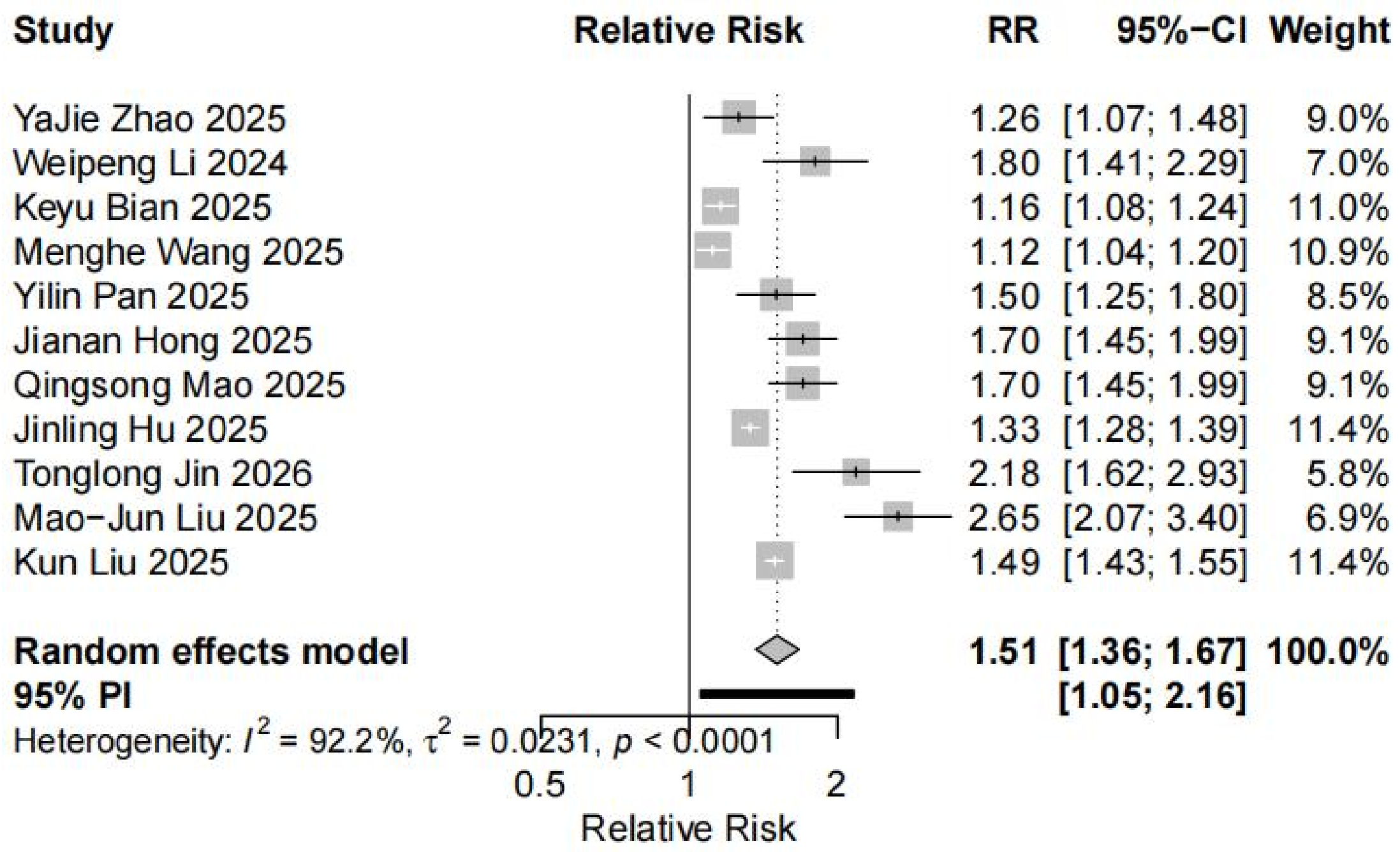

(D)CHD incidence

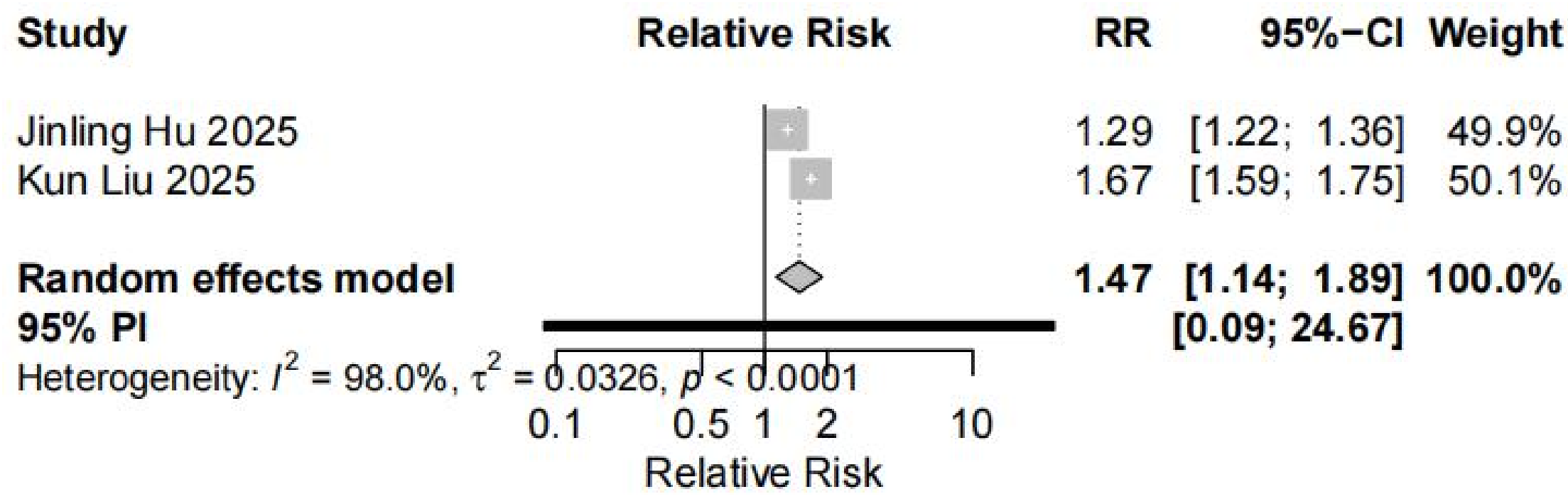

(E)Stroke incidence

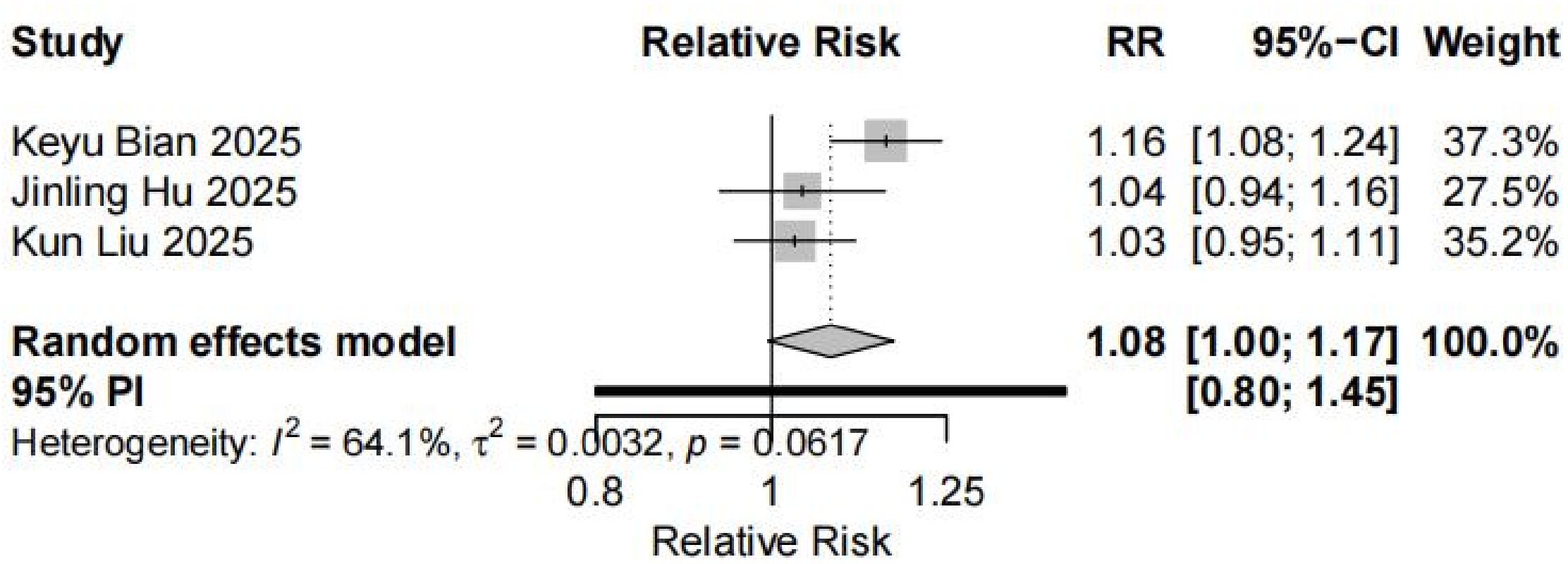

Figure S9. Forest plot of the association between categorized TyG-WhtR index (highest vs. reference category) and risk of (A) all-cause mortality, (B) CVD mortality, (C) CVD incidence, (D) CHD incidence and (E) stroke incidence. TyG-WHtR, Triglyceride-Glucose Waist-to-Height Ratio; SD, Standard Deviation; CVD, Cardiovascular Disease; CHD, Coronary Heart Disease.

(A)All-cause mortality

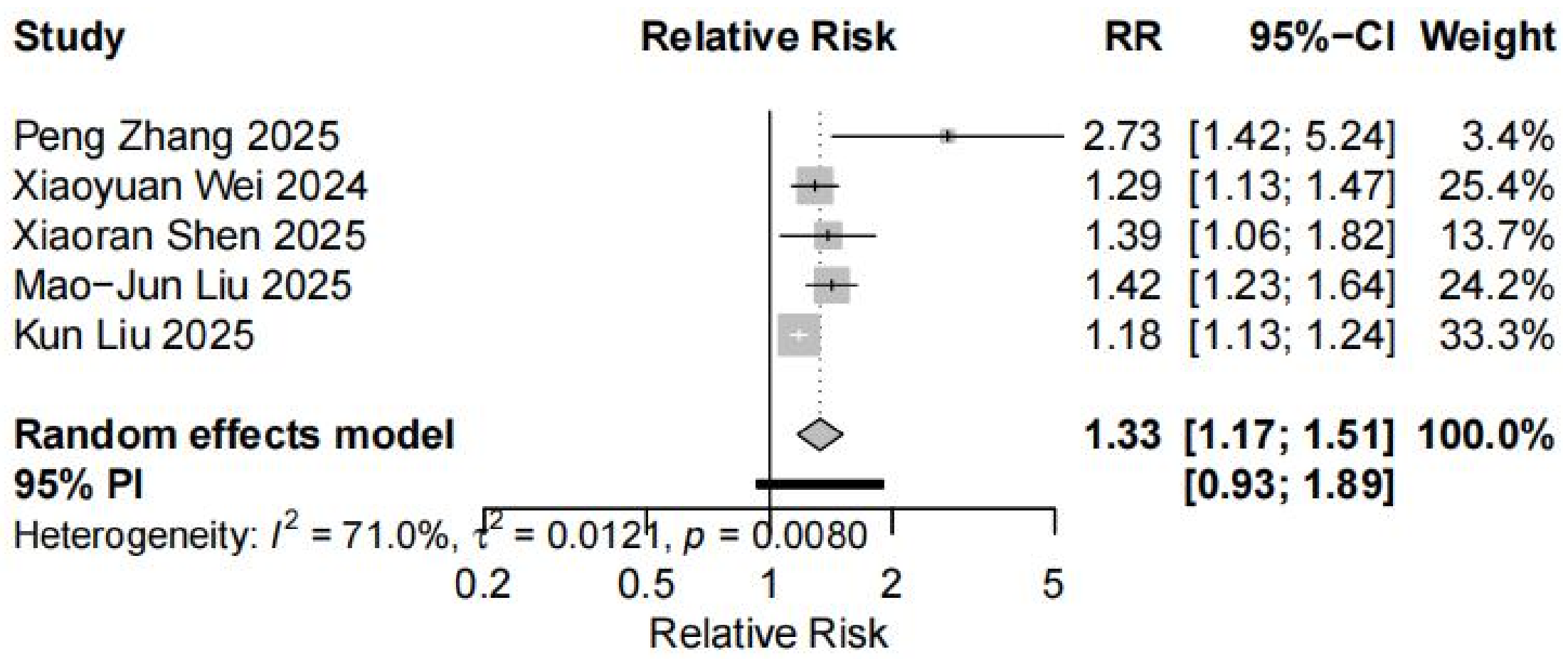

(B)CVD mortality

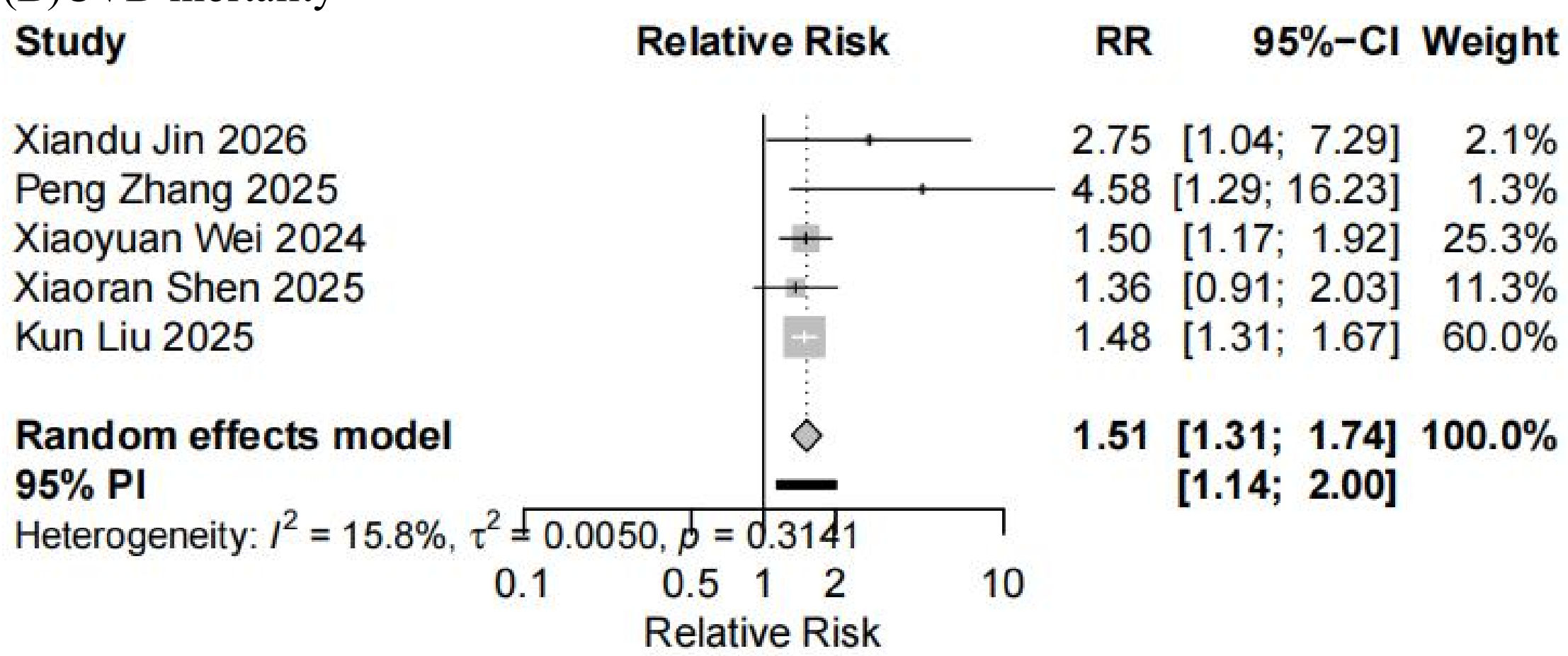

(C)CVD incidence

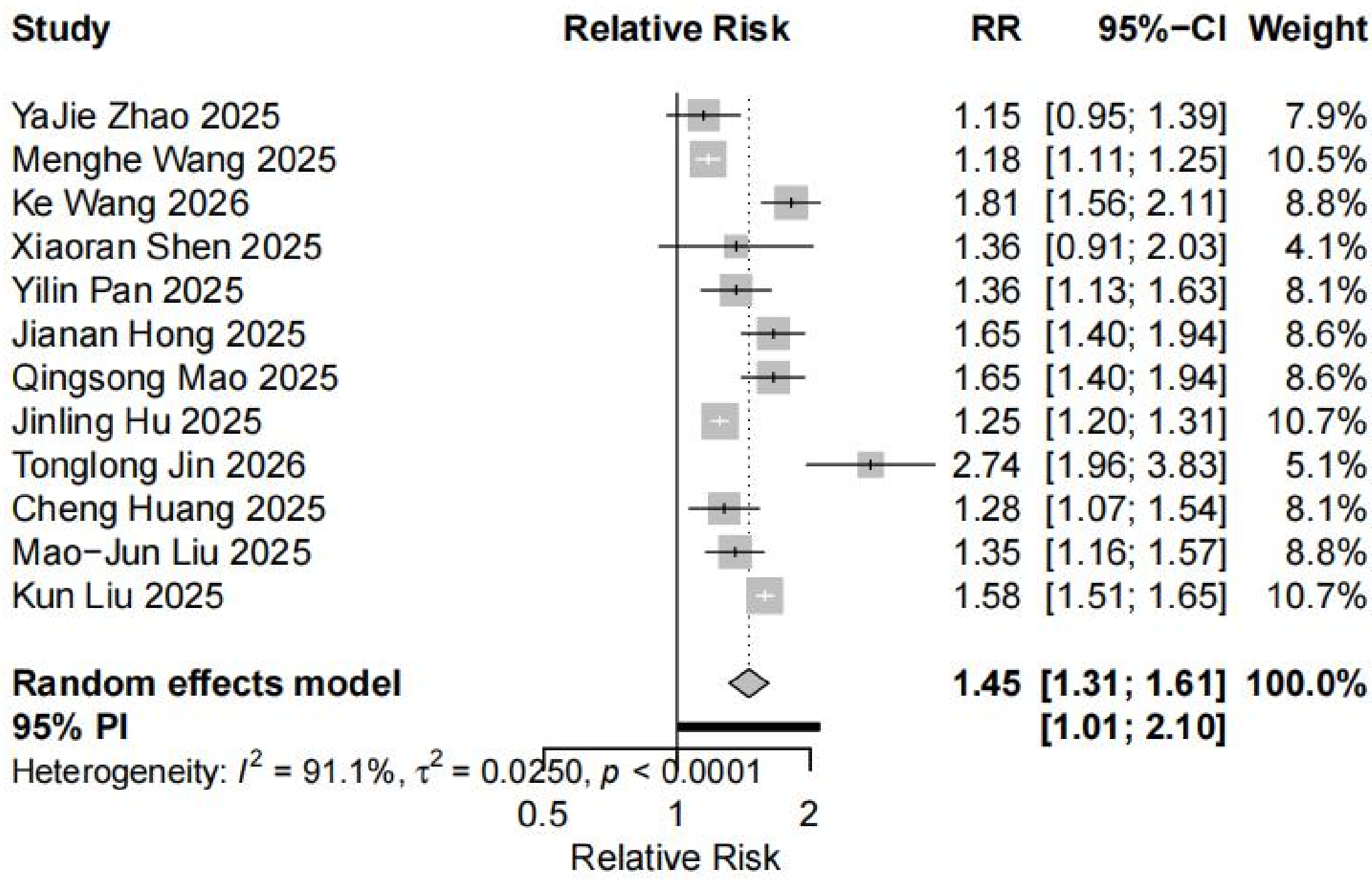

(D)CHD incidence

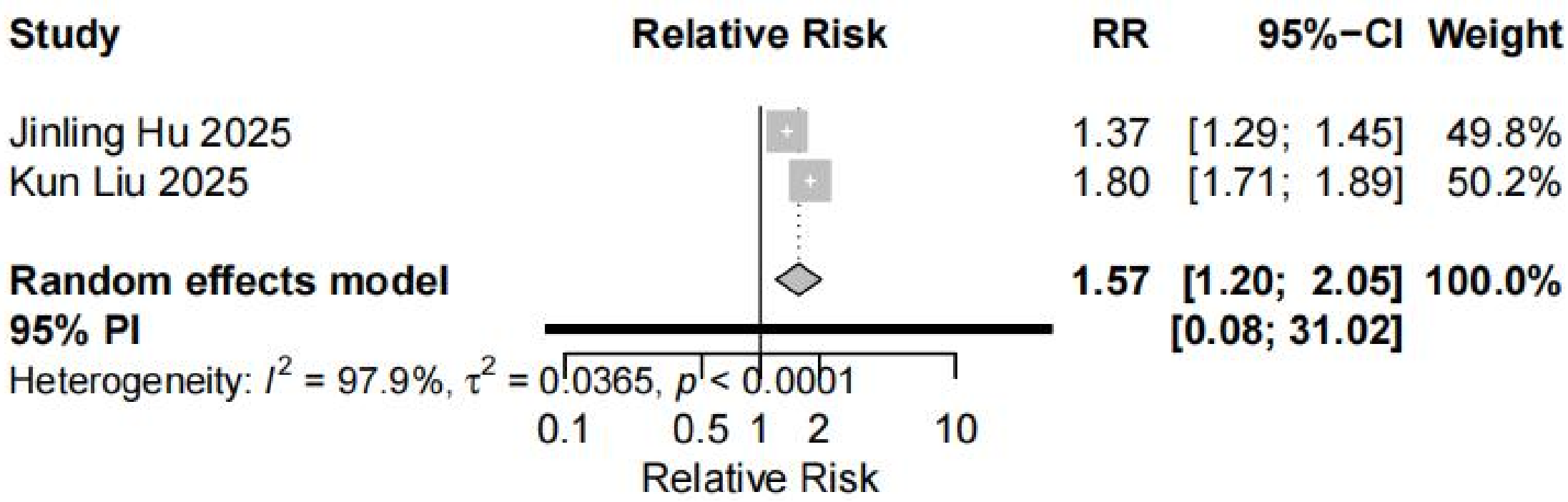

(E)Stroke incidence

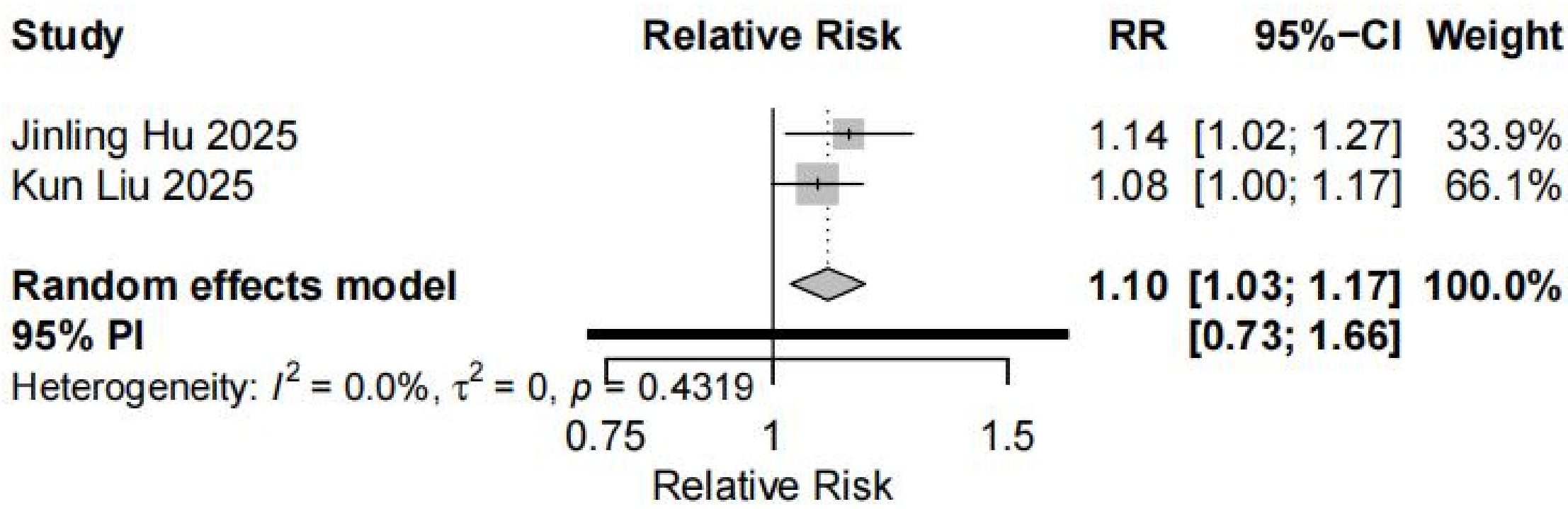

Figure S10. Forest plot of the association between categorized CTI index (highest vs. reference category) and risk of (A) CVD incidence and (B)Stroke incidence. CTI, C-Reactive Protein-Triglyceride Glucose; SD, Standard Deviation; CVD, Cardiovascular Disease.

(A)CVD incidence

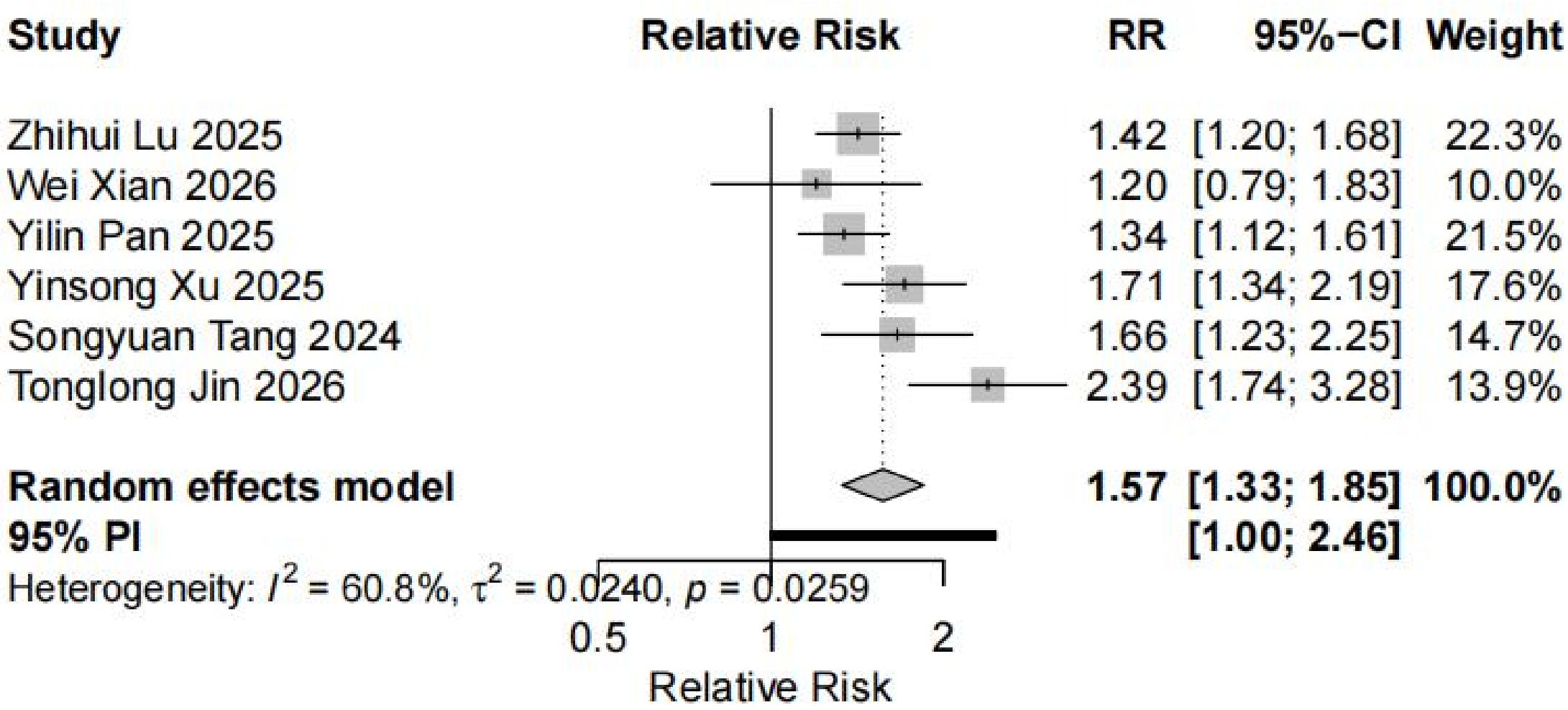

(B)Stroke incidence

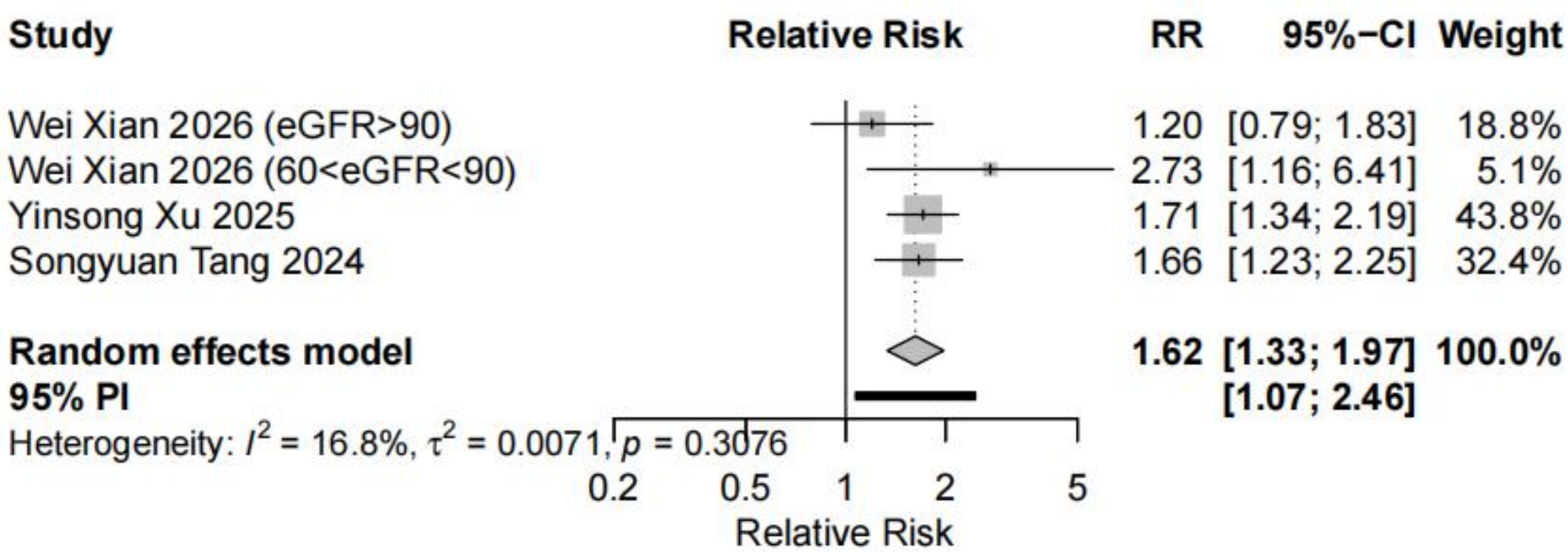

Figure S11. Leave-one-out analysis for the association between (A)TyG index (per 1-SD increase), (B) categorised TyG index (highest vs. reference category) and risks of all-cause mortality. TyG, Triglyceride-Glucose.

(A) TyG-BMI index (per 1-SD increase)

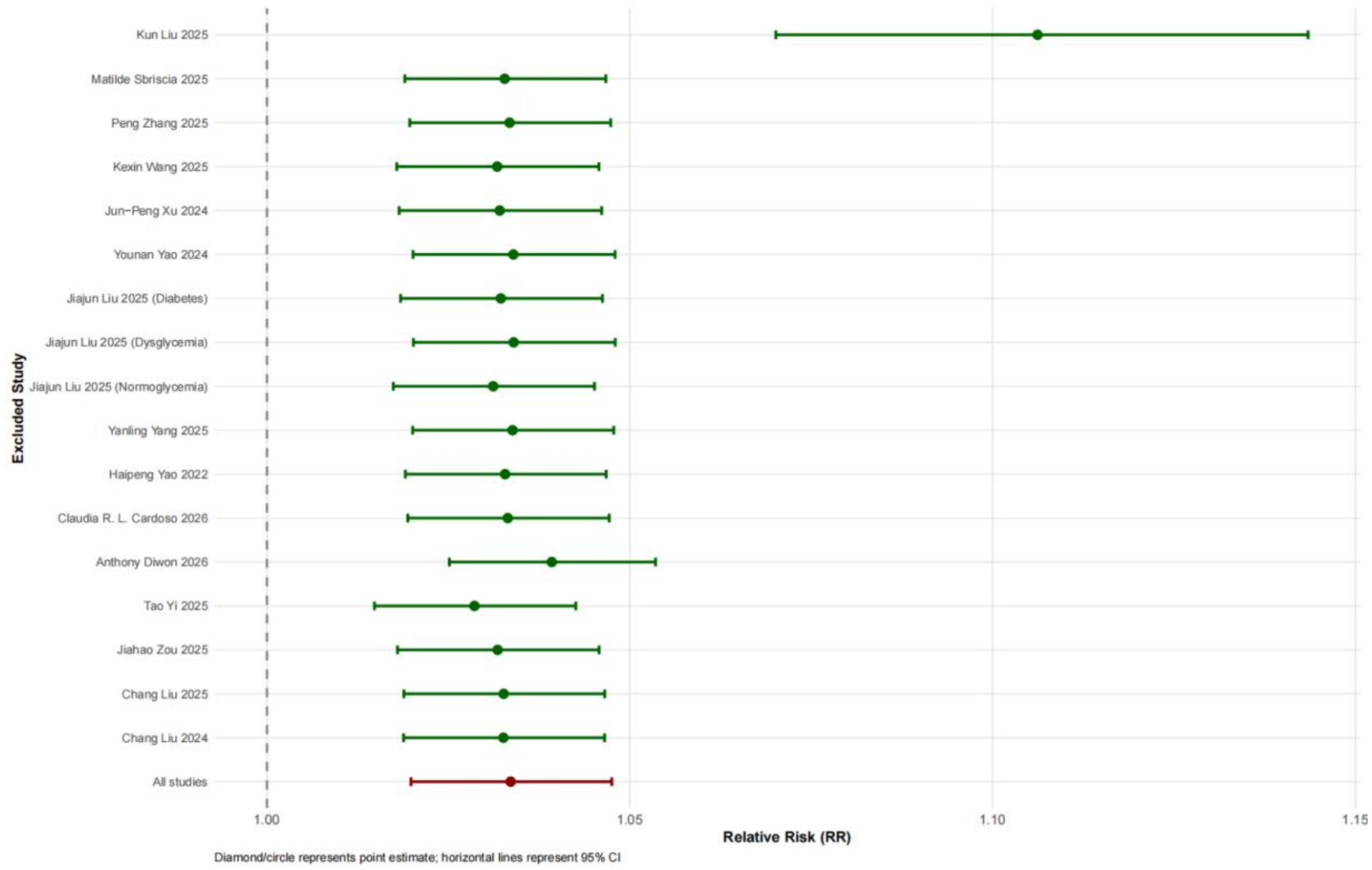

(B) Categorised TyG-BMI index

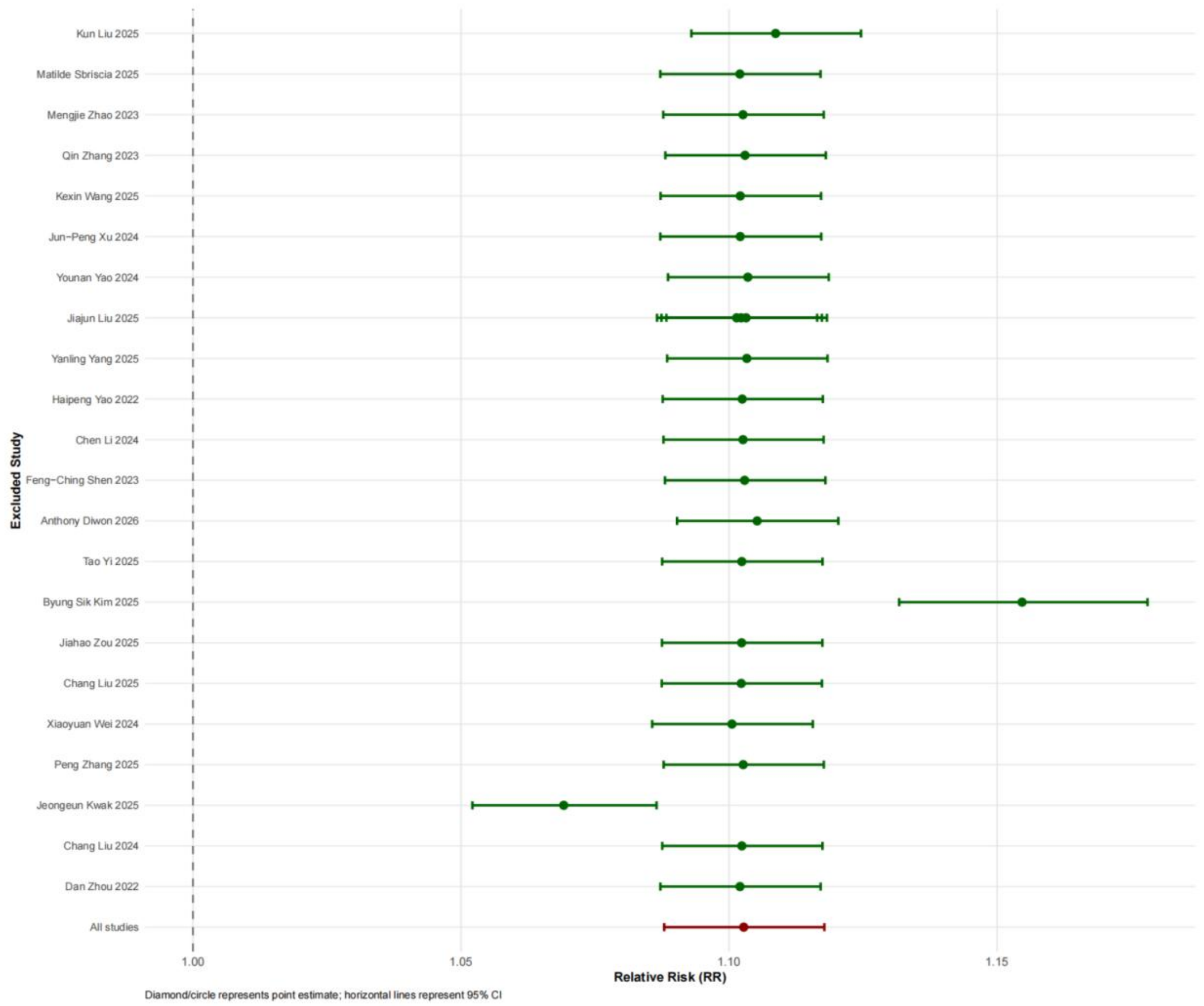

Figure S12. Leave-one-out analysis for the association between TyG index (per 1-SD increase), (B) categorised TyG index (highest vs. reference category) and risks of CVD mortality. TyG, Triglyceride-Glucose. CVD, Cardiovascular Disease.

(A)TyG index (per 1-SD increase)

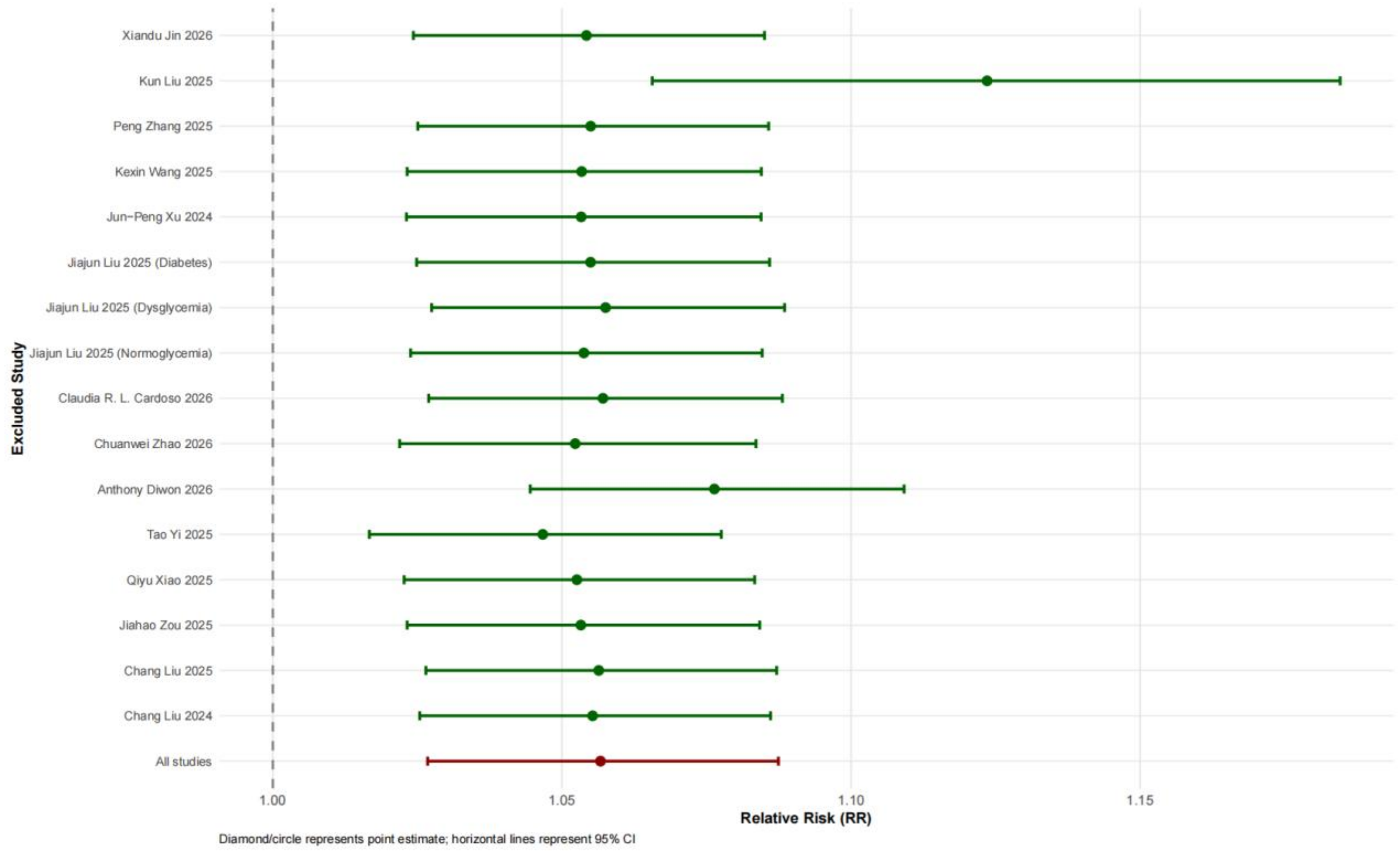

(B) Categorised TyG index

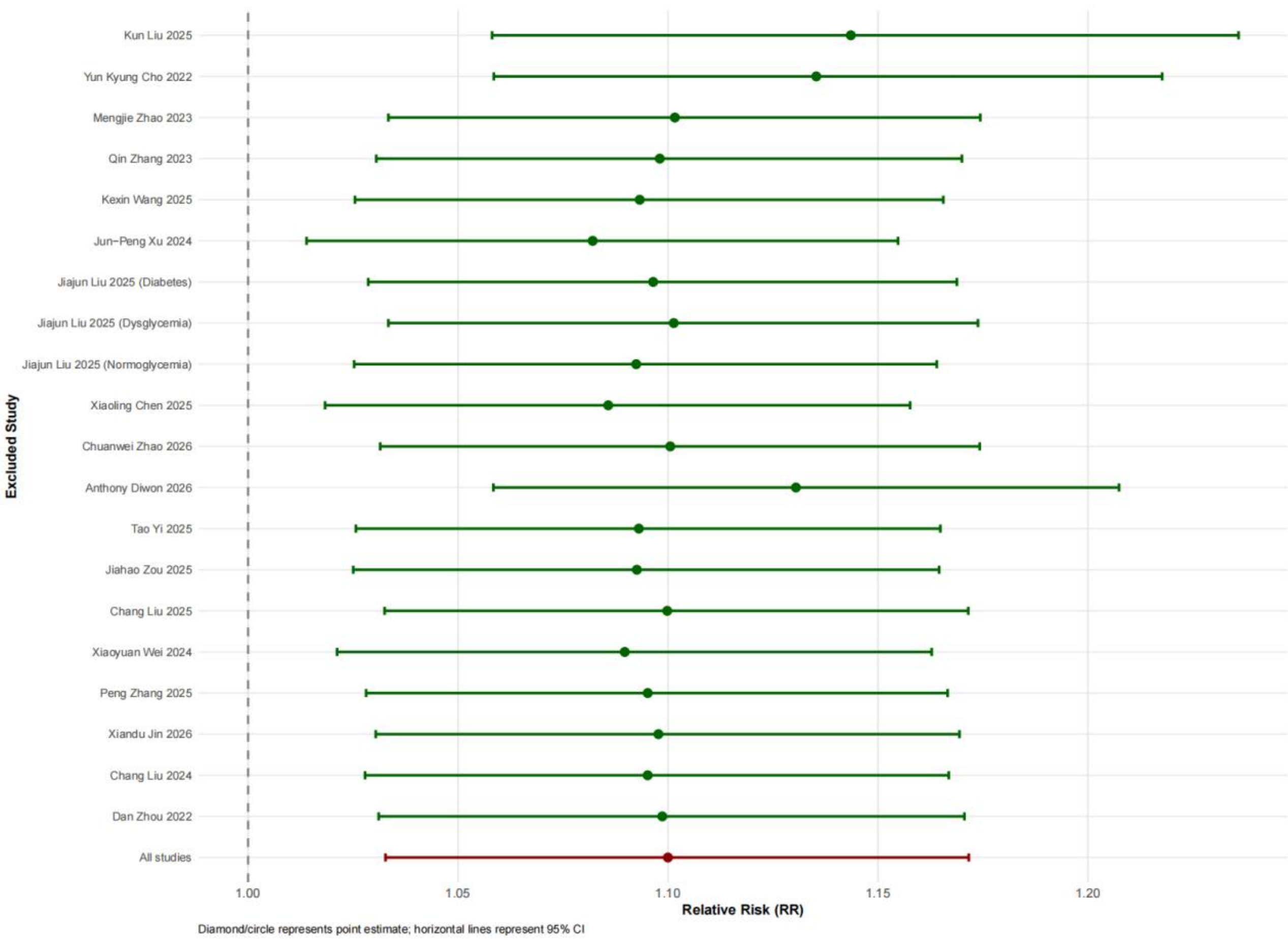

Figure S13. Leave-one-out analysis for the association between TyG index (per 1-SD increase) , (B) categorised TyG index (highest vs. reference category) and risks of CVD incidence. TyG, Triglyceride-Glucose. CVD, Cardiovascular Disease.

(A)TyG index (per 1-SD increase)

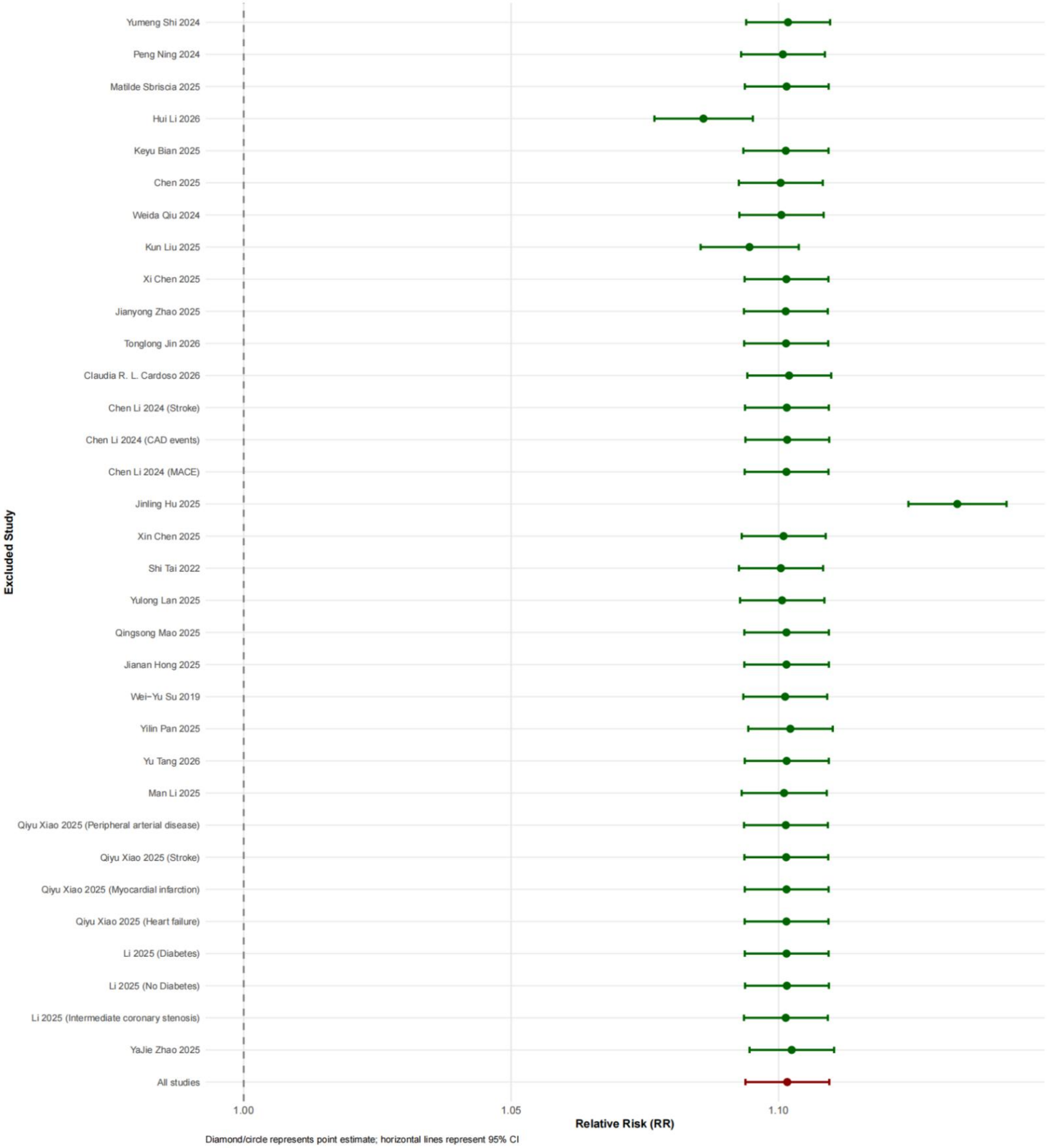

(B) Categorised TyG index

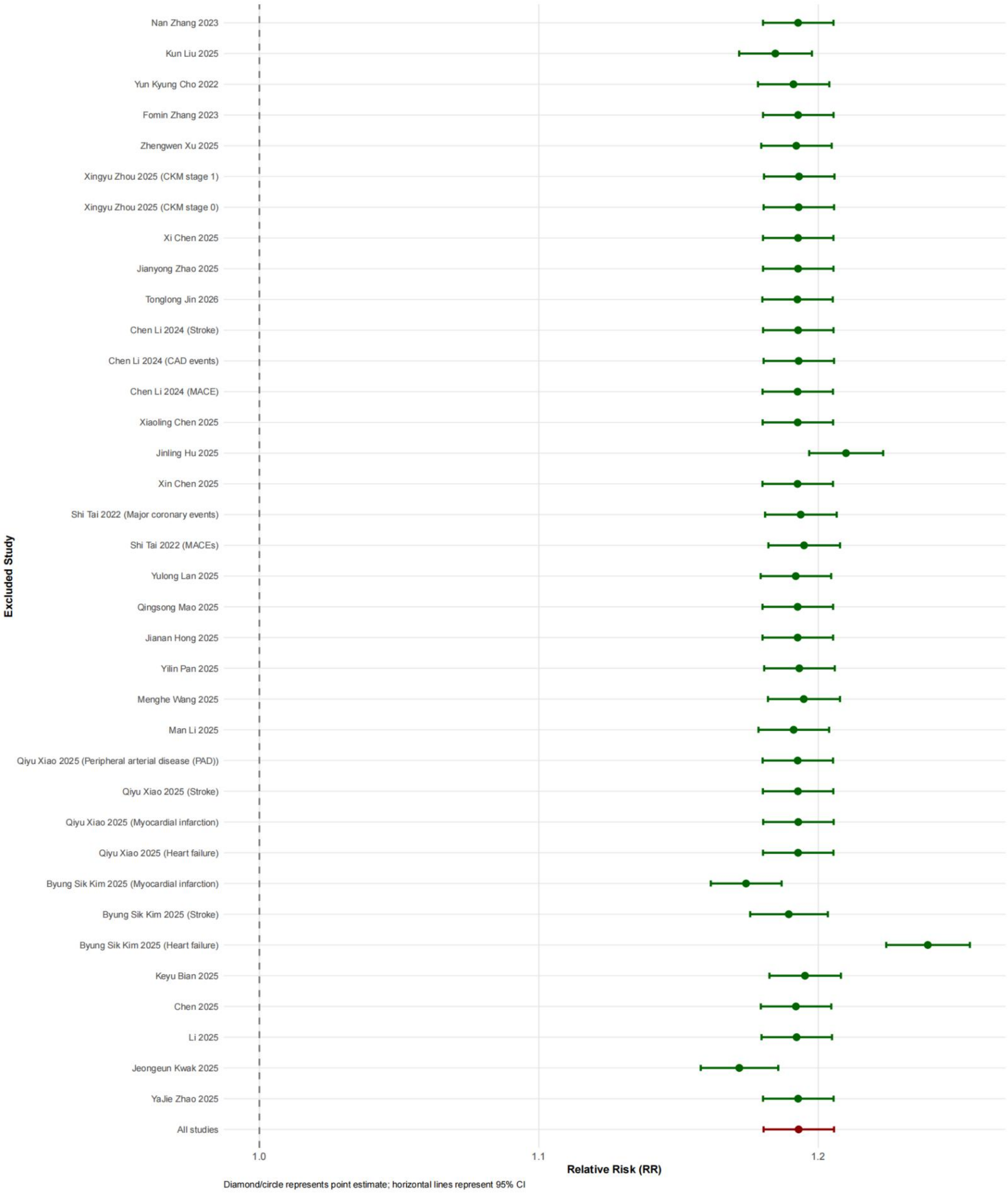

Figure S14. Leave-one-out analysis for the association between TyG index (per 1-SD increase) , (B) categorised TyG index (highest vs. reference category) and risks of CHD incidence. TyG, Triglyceride-Glucose. CHD, Coronary Heart Disease.

(A)TyG index (per 1-SD increase)

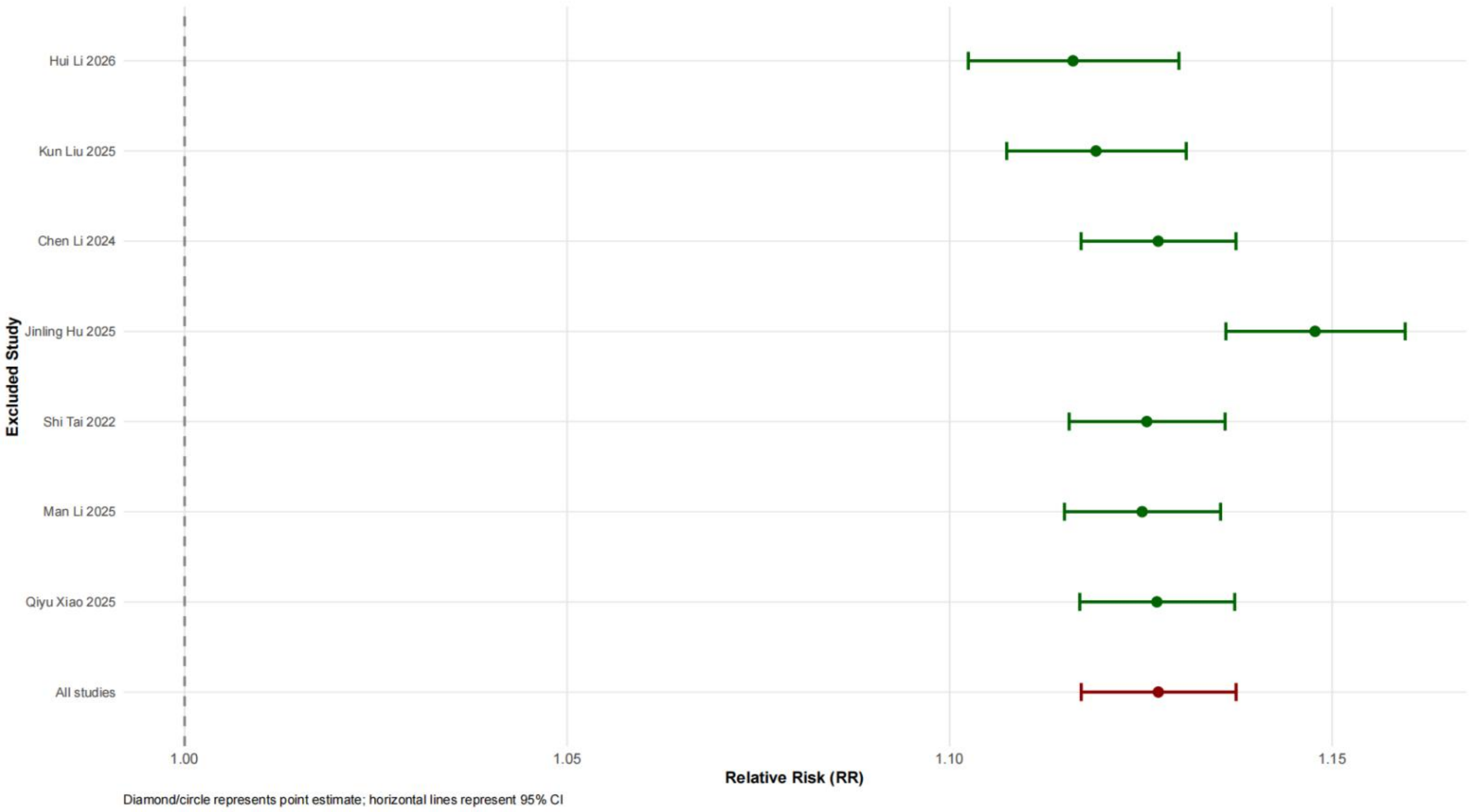

(B) Categorised TyG index

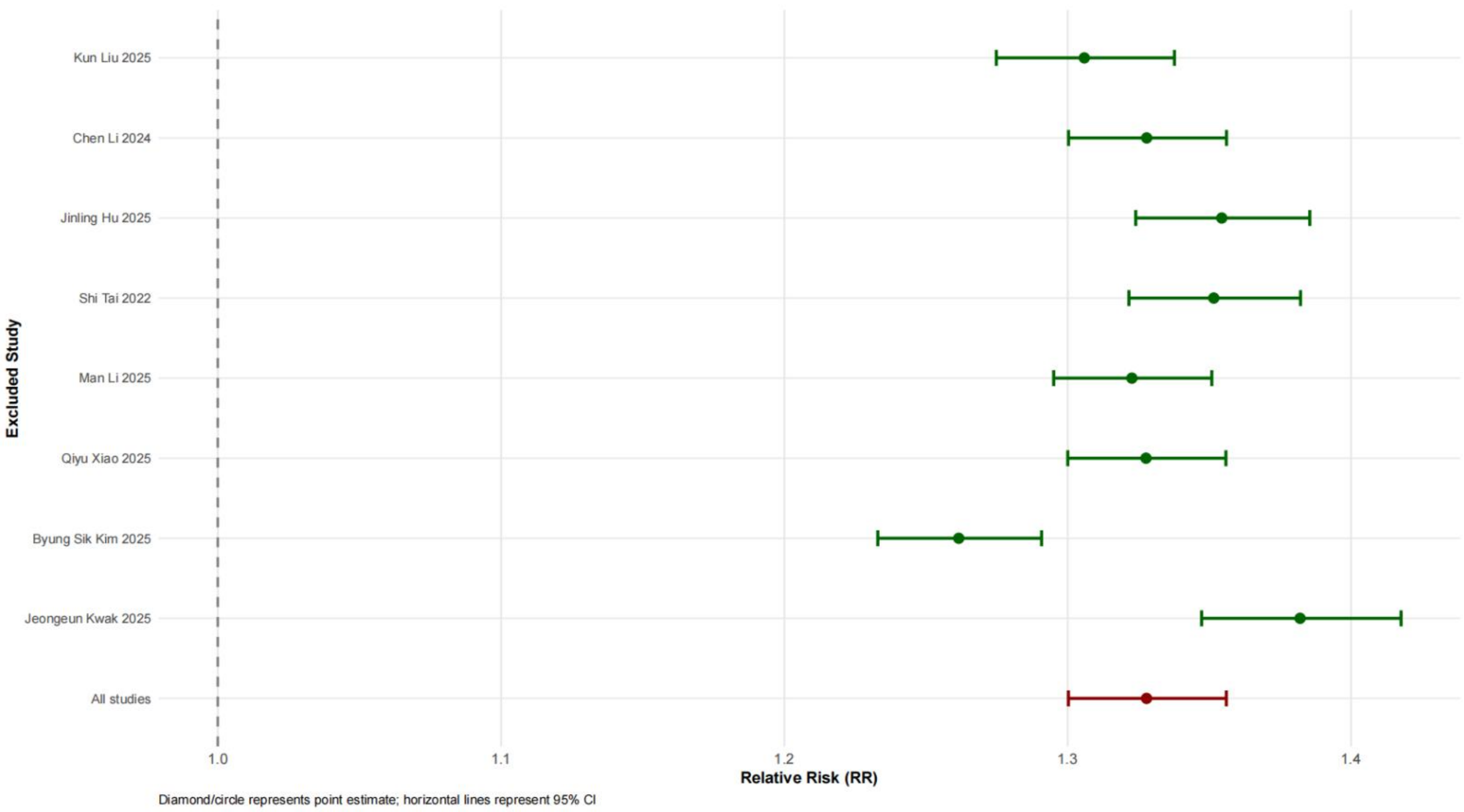

Figure S15. Leave-one-out analysis for the association between TyG index (per 1-SD increase) , (B) categorised TyG index (highest vs. reference category) and risks of stroke incidence. TyG, Triglyceride-Glucose.

(A)TyG index (per 1-SD increase)

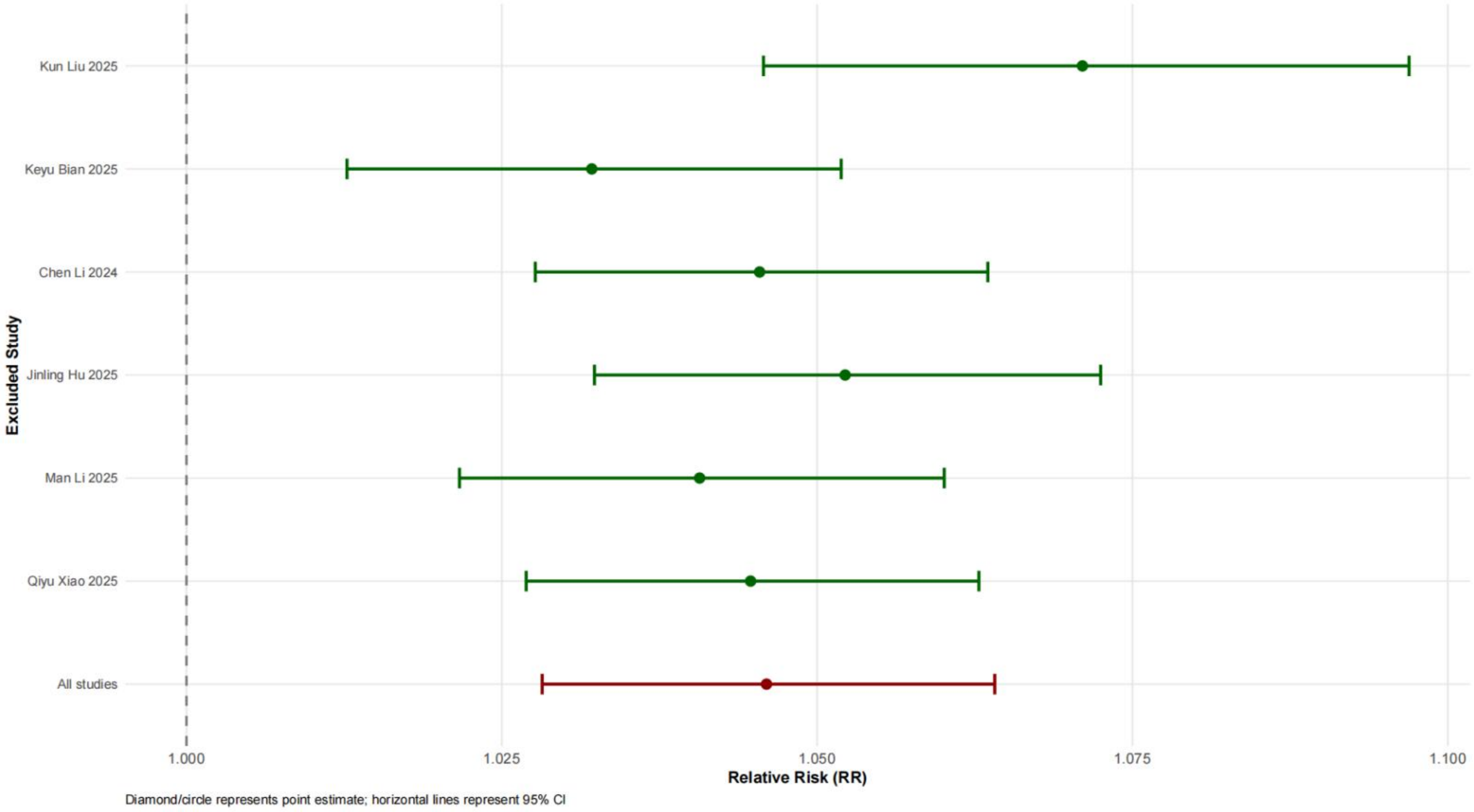

(B) Categorised TyG index

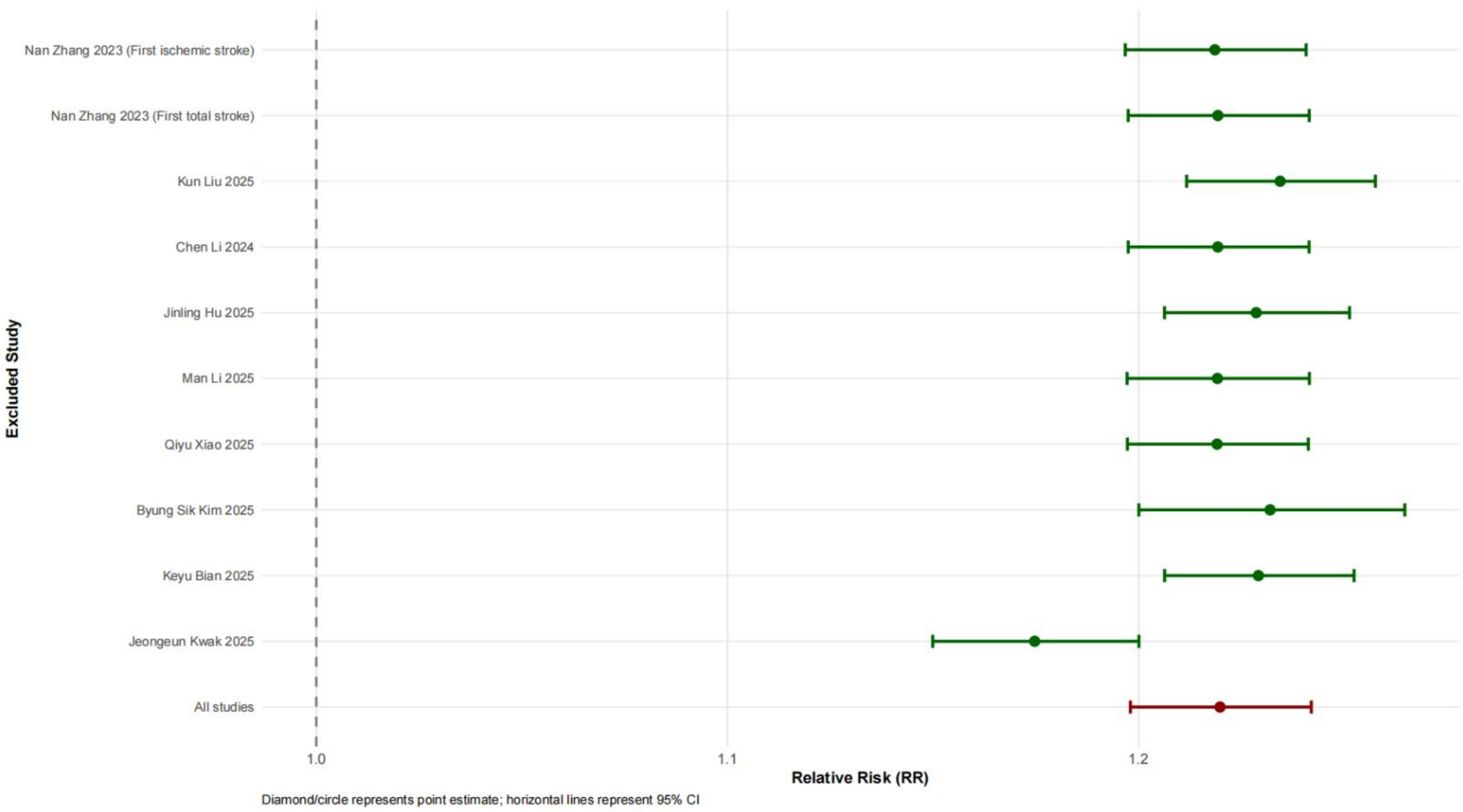

Figure S16. Leave-one-out analysis for the association between TyG index (per 1-SD increase) , (B) categorised TyG index (highest vs. reference category) and risks of heart failure incidence. TyG, Triglyceride-Glucose.

(A)TyG index (per 1-SD increase)

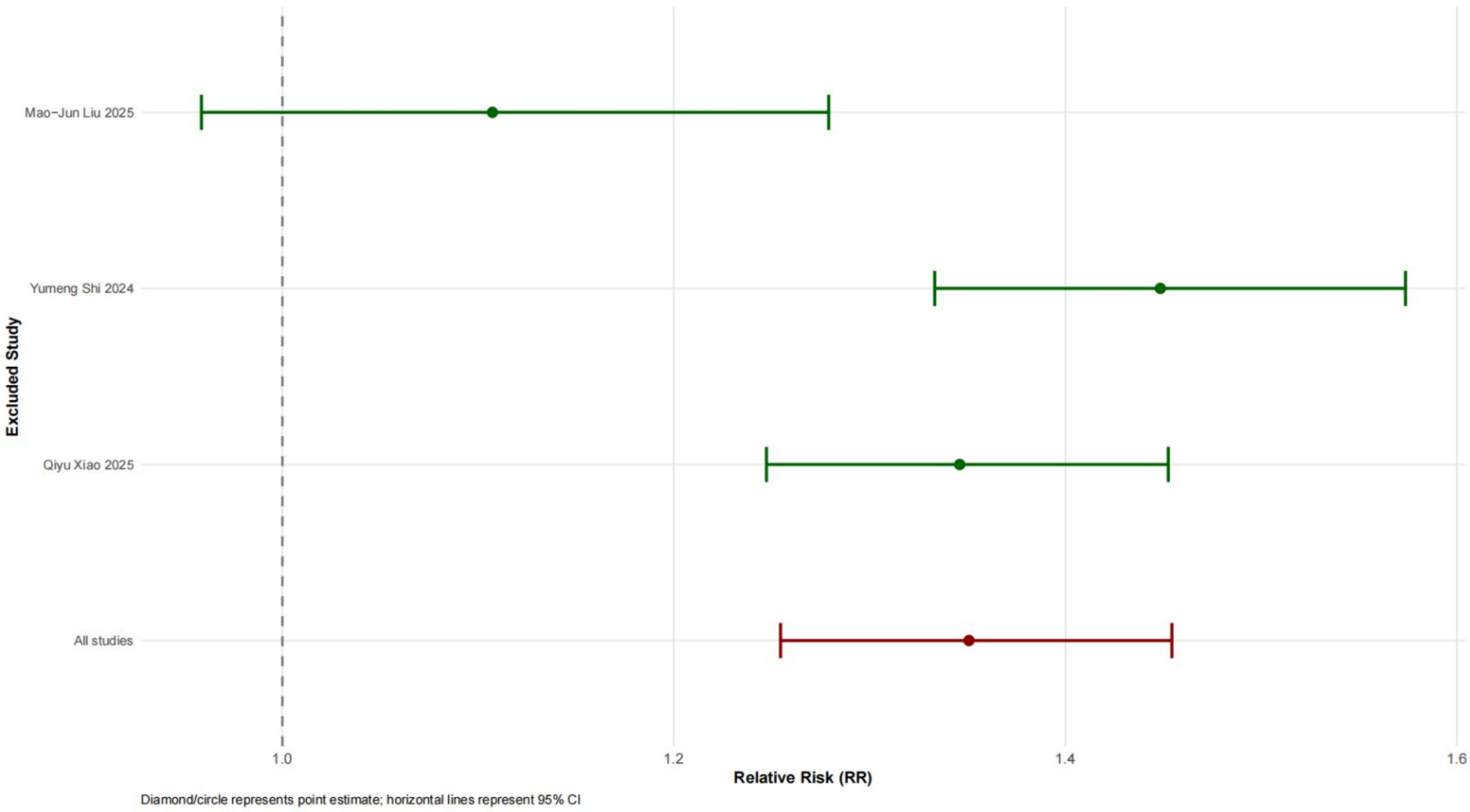

(B) Categorised TyG index

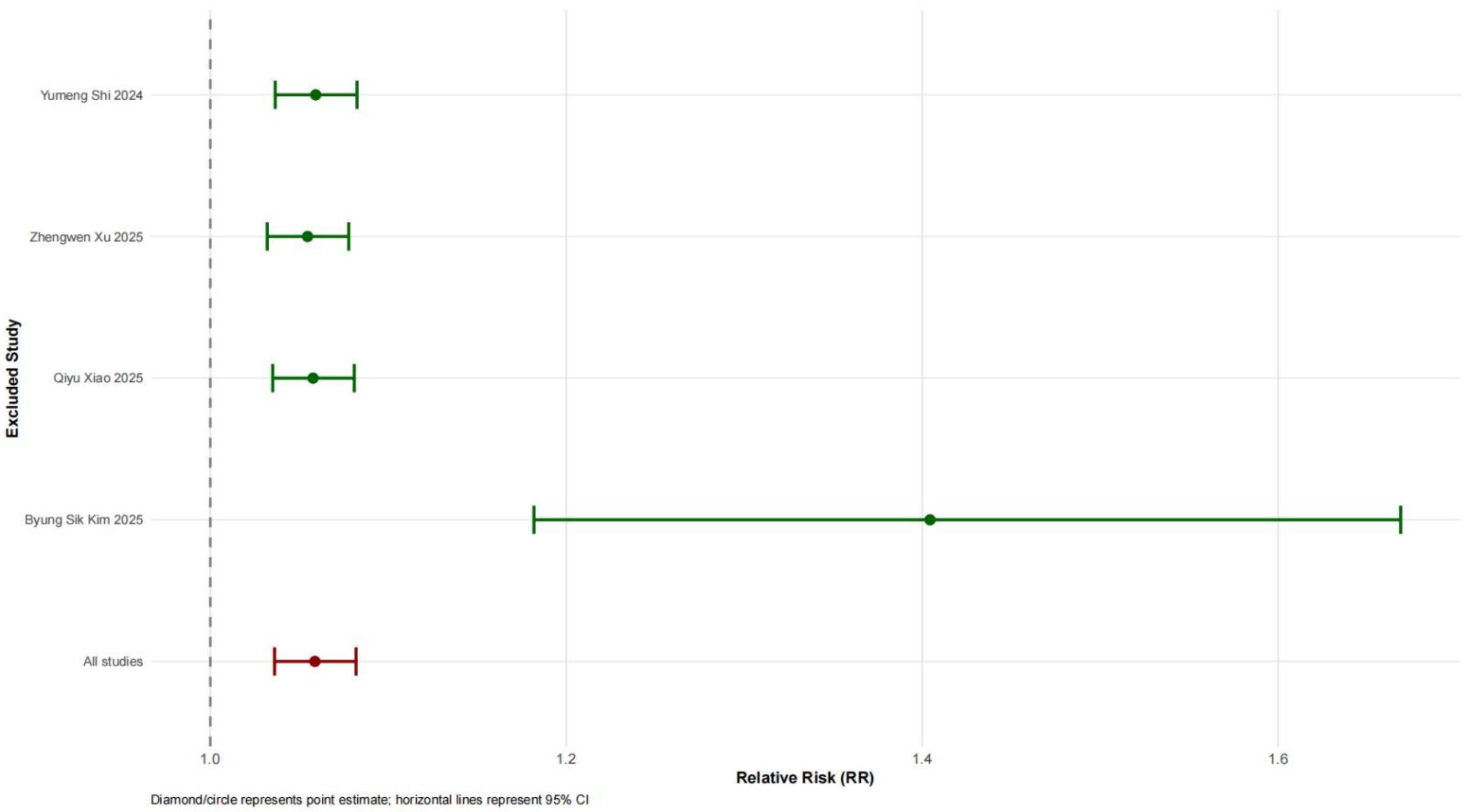

Figure S17. Leave-one-out analysis for the association between TyG index (per 1-SD increase) , (B) categorised TyG index (highest vs. reference category) and risks of MACE incidence. TyG, Triglyceride-Glucose. MACE, Major Adverse Cardiovascular Events.

(A)TyG index (per 1-SD increase)

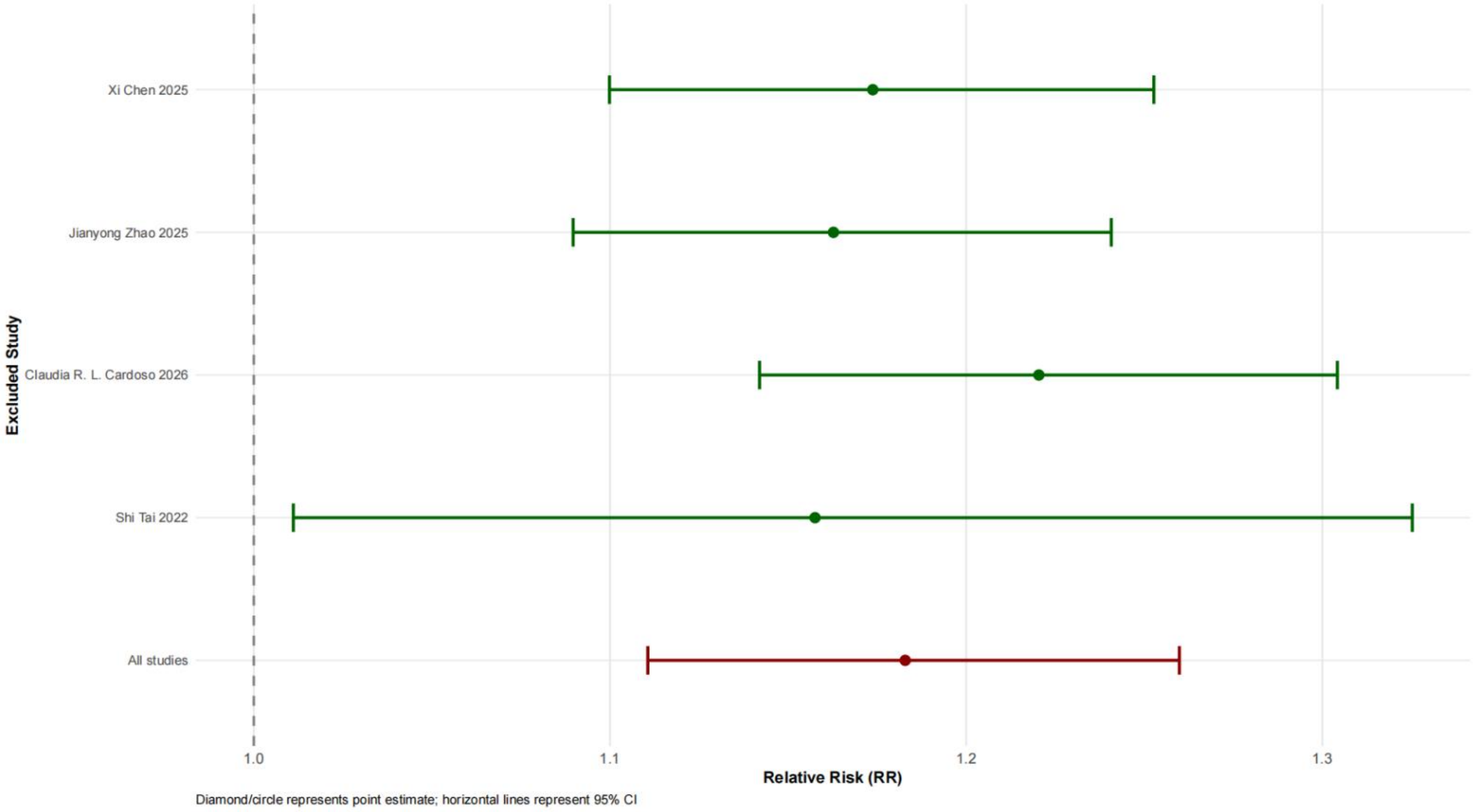

(B) Categorised TyG index

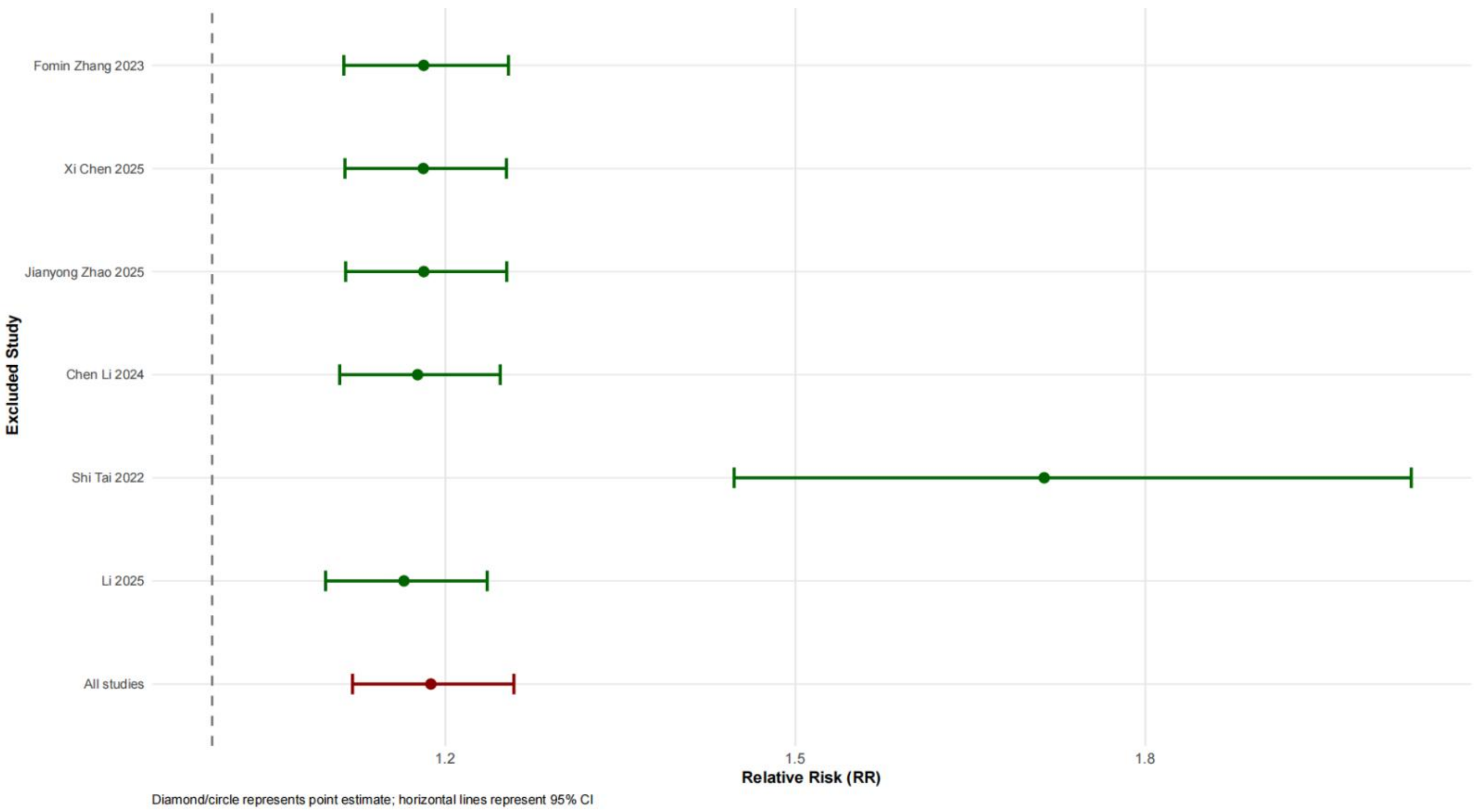

Figure S18. Leave-one-out analysis for the association between TyG-BMI index (per 1-SD increase), (B) categorised TyG-BMI index (highest vs. reference category) and risks of all-cause mortality. TyG-BMI, Triglyceride Glucose-Body Mass Index.

(A)TyG index (per 1-SD increase)

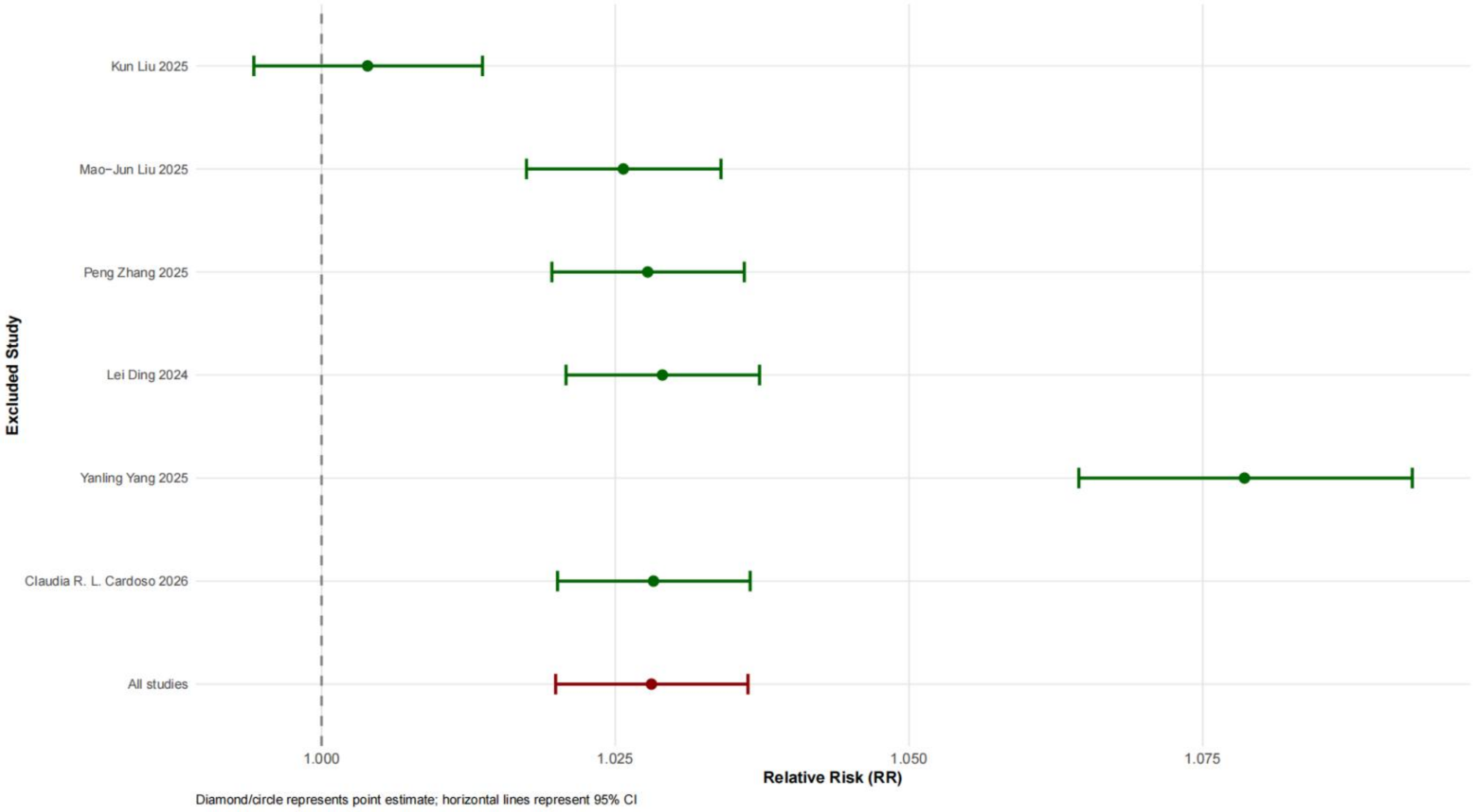

(B) Categorised TyG index

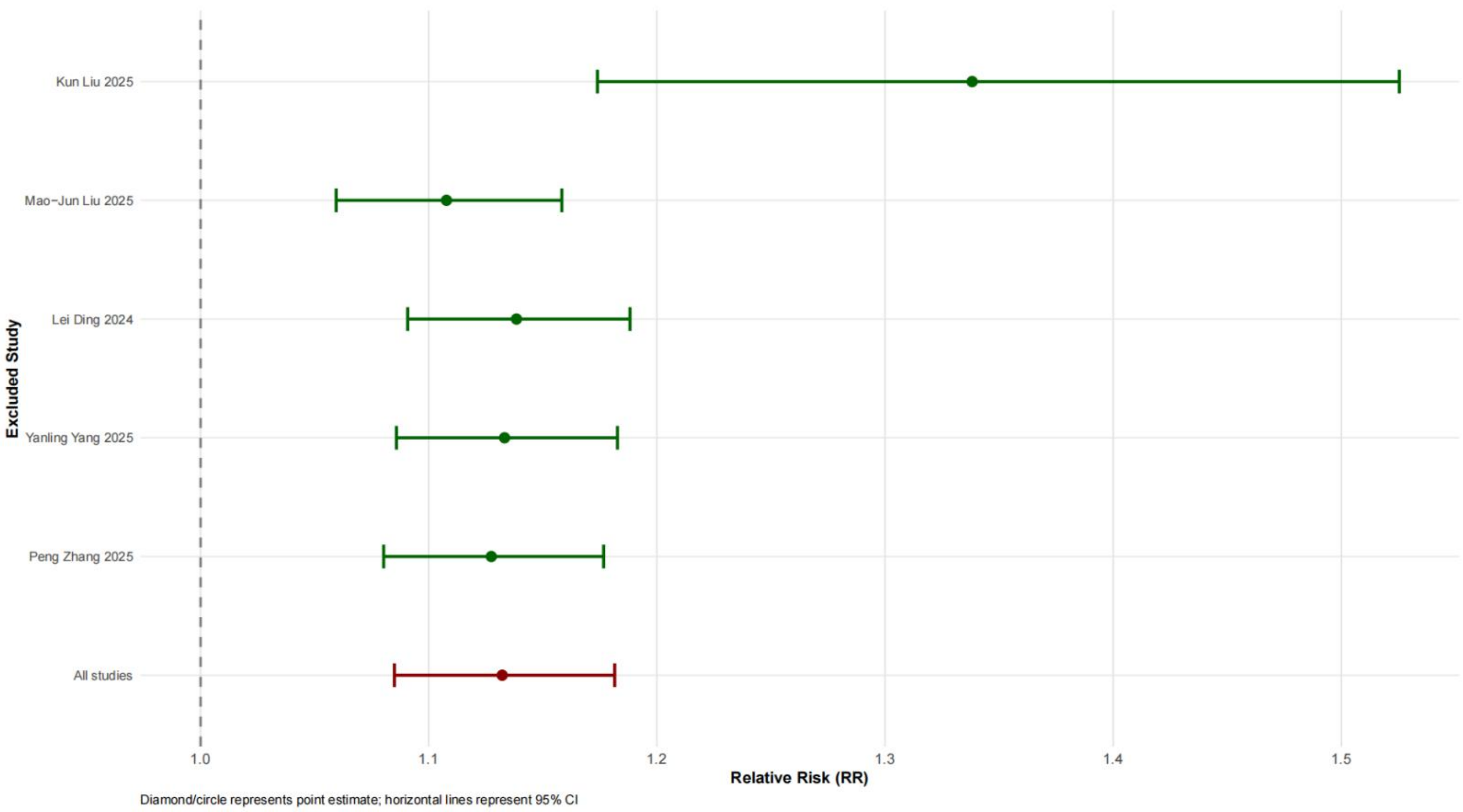

Figure S19. Leave-one-out analysis for the association between TyG-BMI index (per 1-SD increase), (B) categorised TyG index (highest vs. reference category) and risks of CVD mortality. TyG-BMI, Triglyceride Glucose-Body Mass Index. CVD, Cardiovascular Disease.

(A)TyG-BMI index (per 1-SD increase)

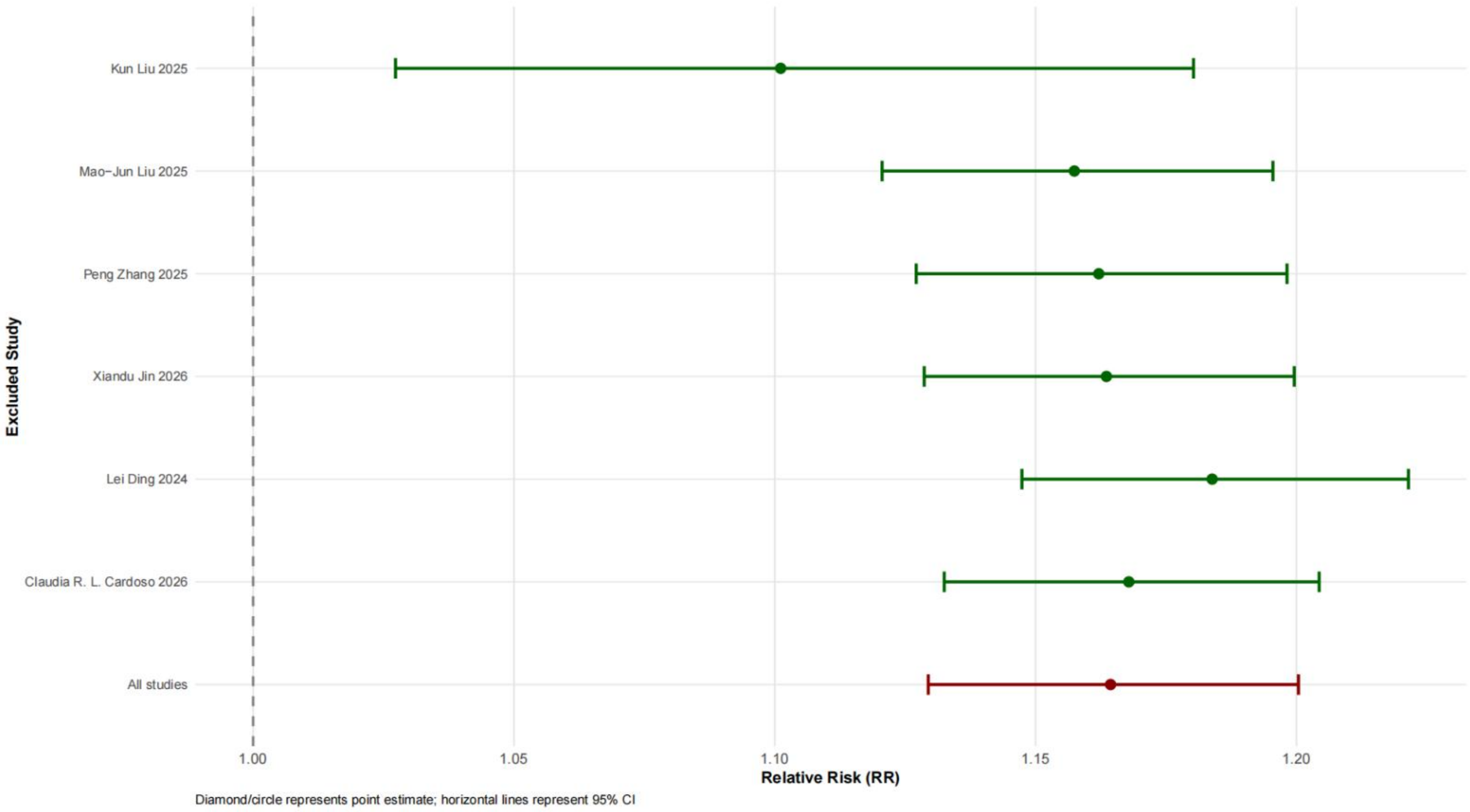

(B) Categorised TyG-BMI index

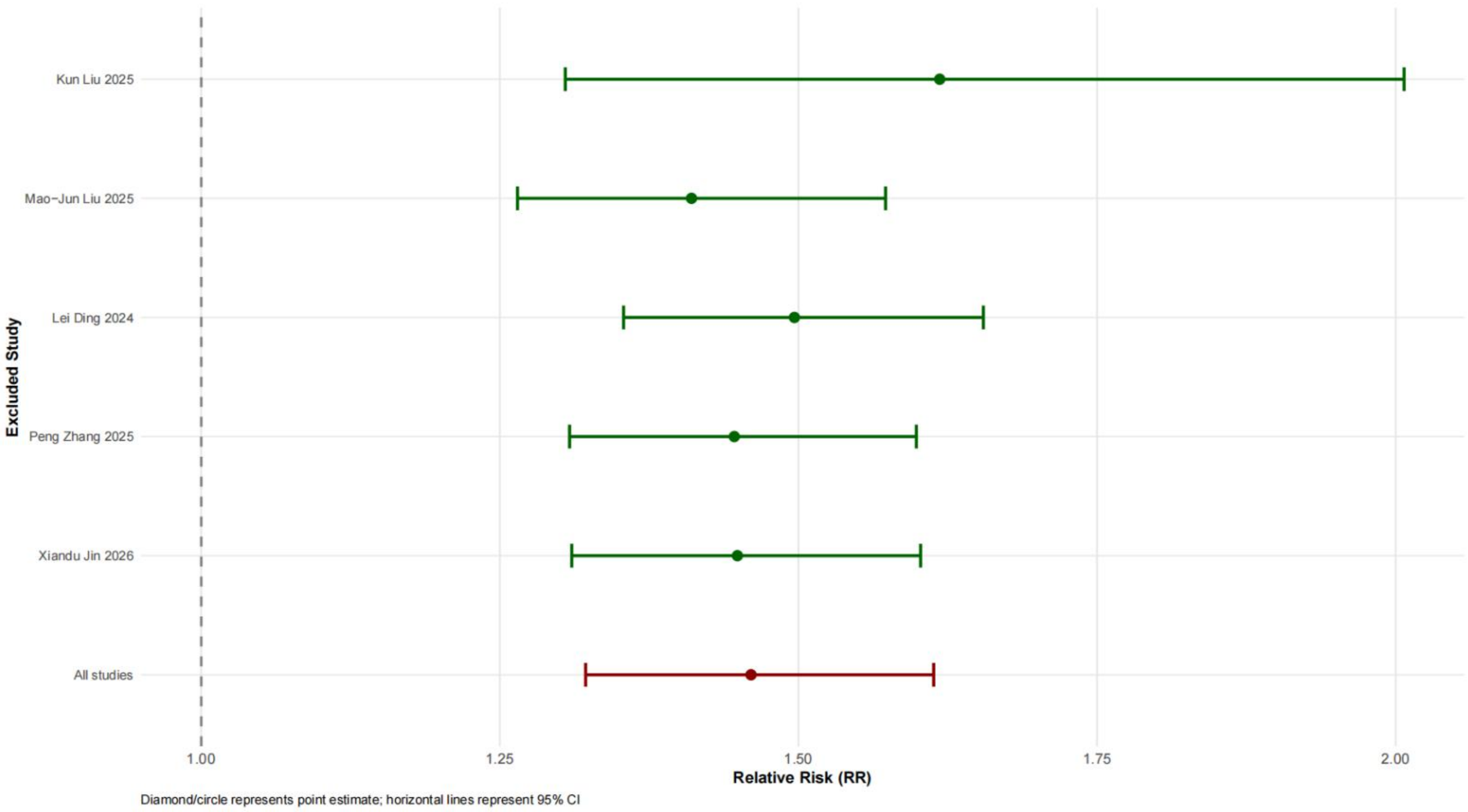

Figure S20. Leave-one-out analysis for the association between TyG-BMI index (per 1-SD increase), (B) categorised TyG index (highest vs. reference category) and risks of CVD incidence. TyG-BMI, Triglyceride Glucose-Body Mass Index. CVD, Cardiovascular Disease.

(A)TyG-BMI index (per 1-SD increase)

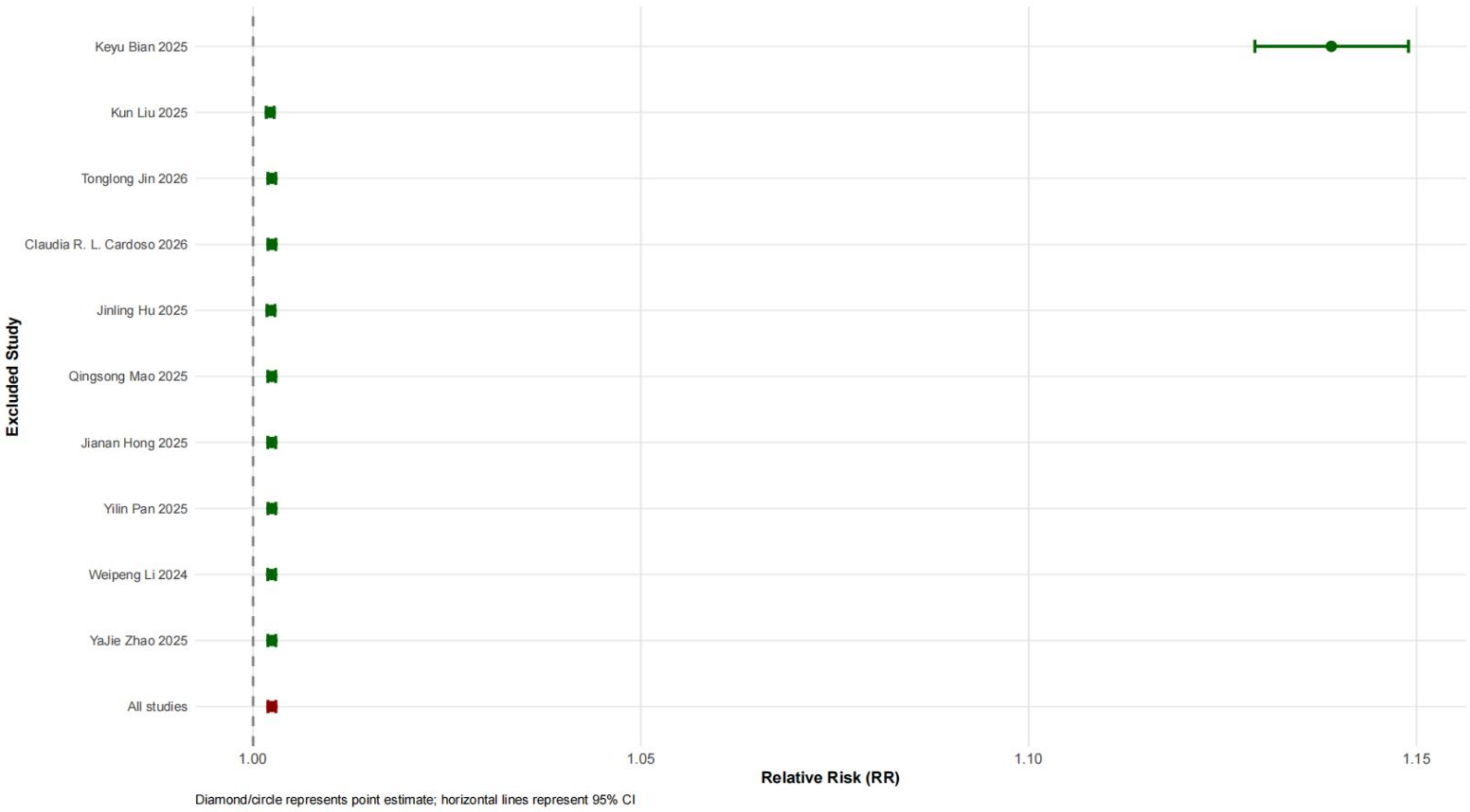

(B) Categorised TyG-BMI index

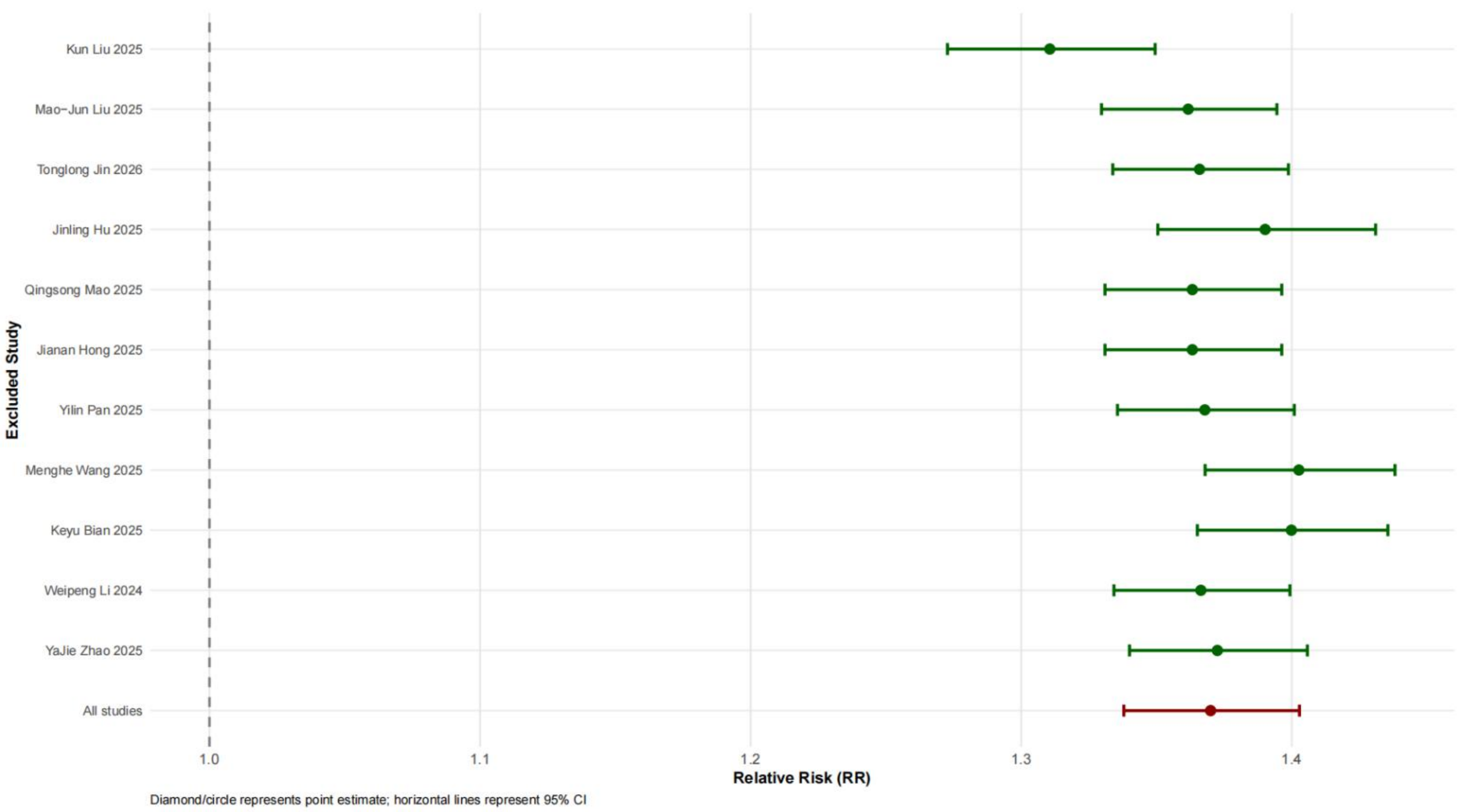

Figure S21. Leave-one-out analysis for the association between TyG-BMI index (per 1-SD increase), (B) categorised TyG index (highest vs. reference category) and risks of CHD incidence. TyG-BMI, Triglyceride Glucose-Body Mass Index. CHD, Coronary Heart Disease.

(A)TyG-BMI index (per 1-SD increase)

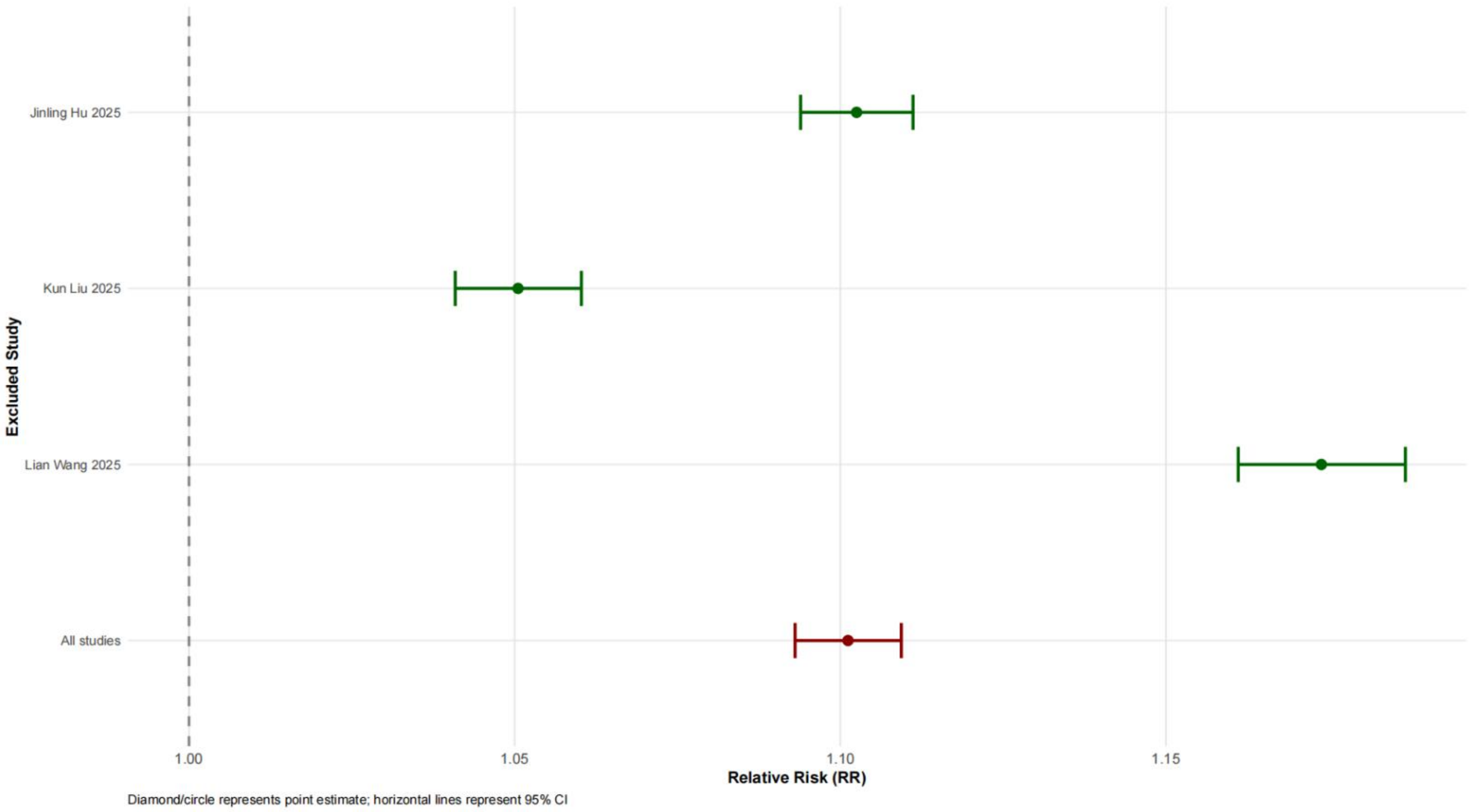

(B) Categorised TyG-BMI index

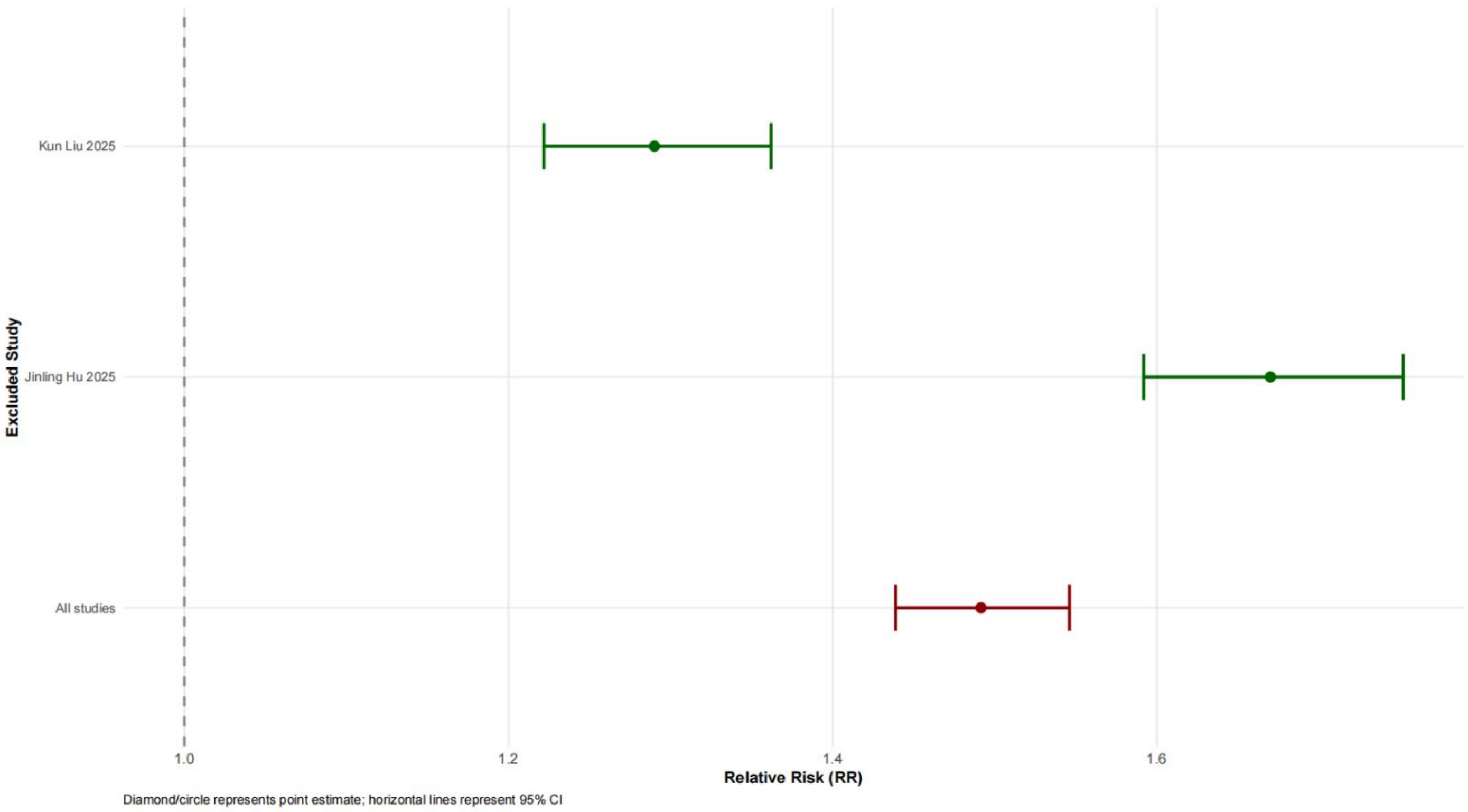

Figure S22. Leave-one-out analysis for the association between TyG-BMI index (per 1-SD increase), (B) categorised TyG index (highest vs. reference category) and risks of stroke incidence. TyG-BMI, Triglyceride Glucose-Body Mass Index.

(A)TyG-BMI index (per 1-SD increase)

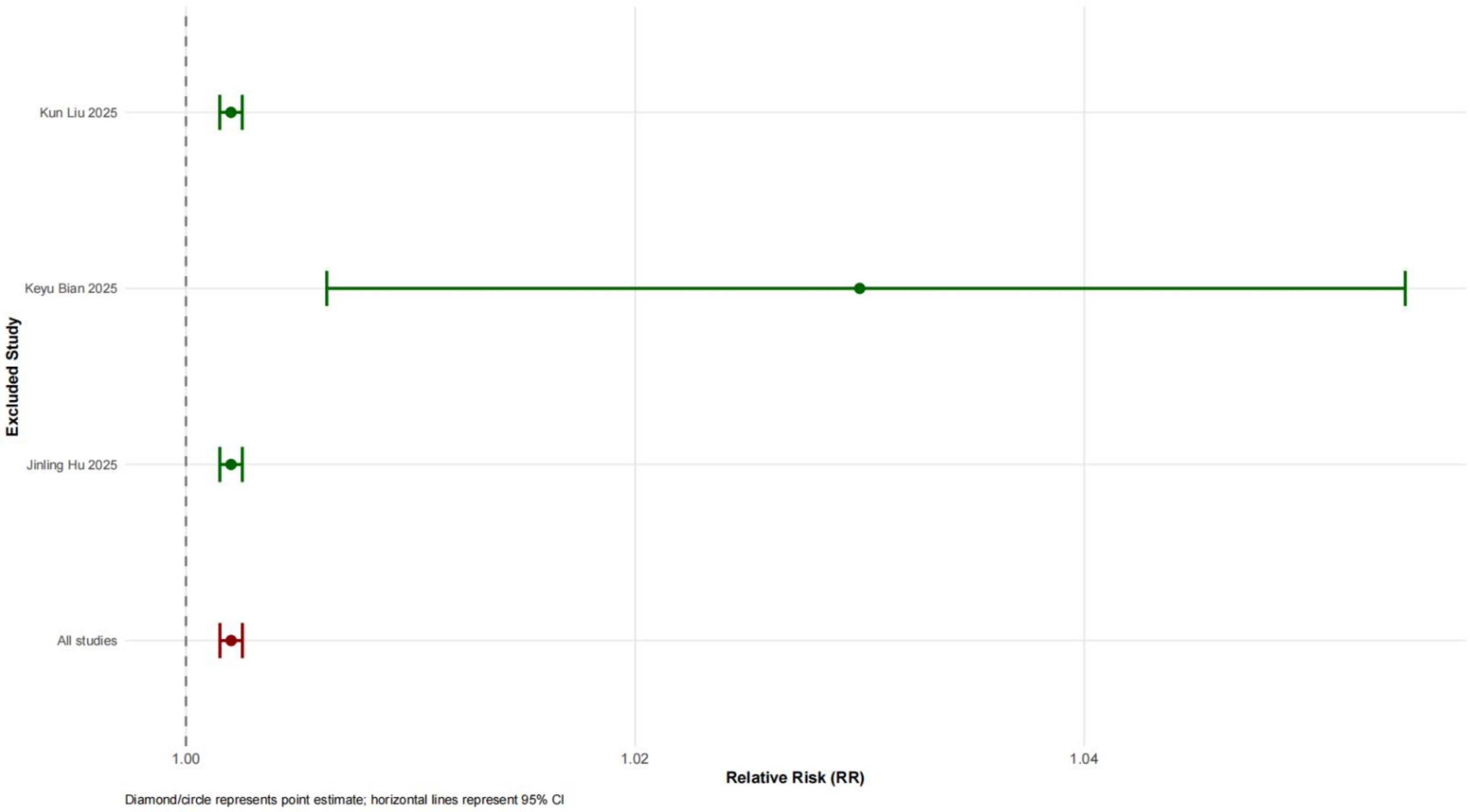

(B) Categorised TyG-BMI index

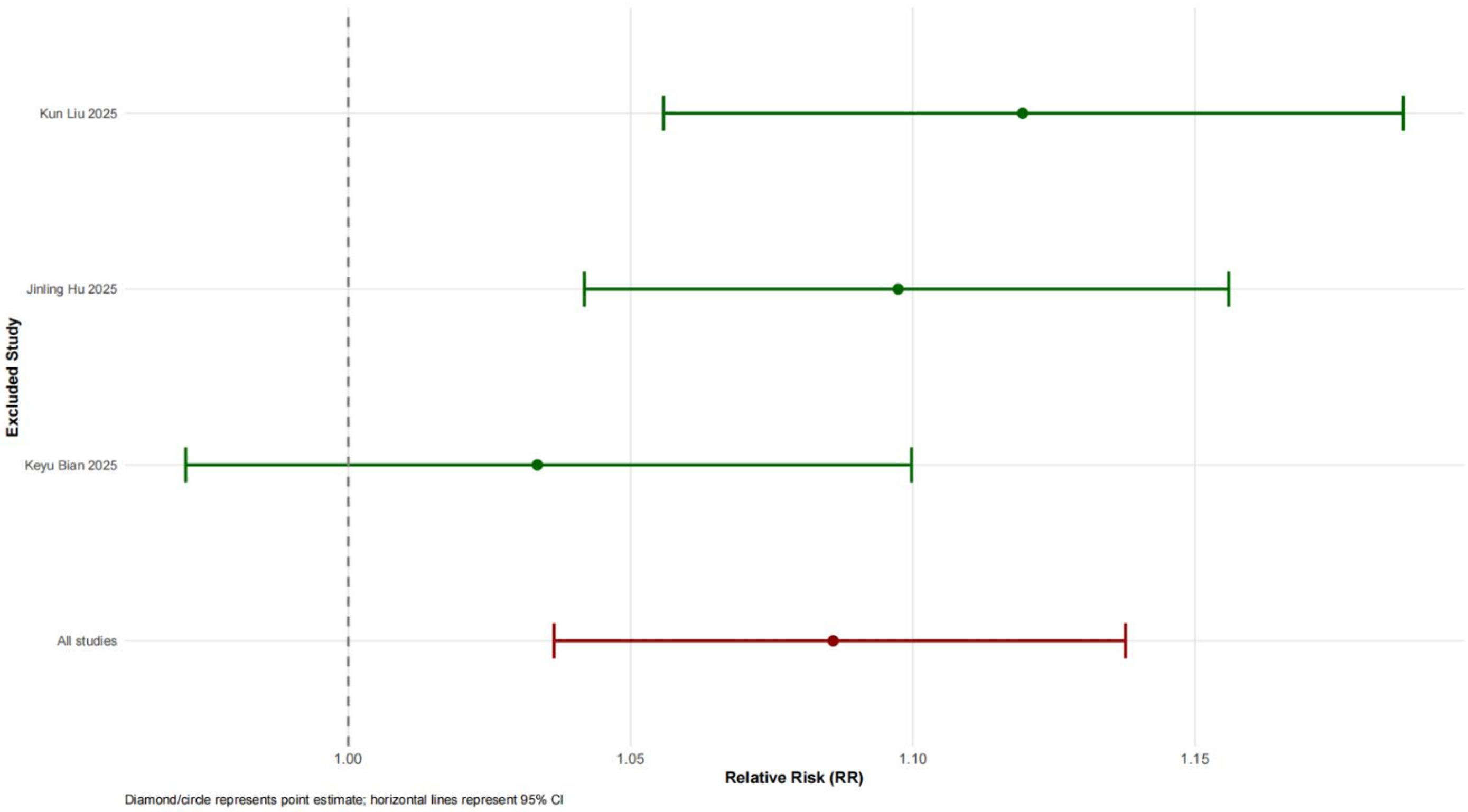

Figure S23. Leave-one-out analysis for the association between TyG-WC index (per 1-SD increase), (B) categorised TyG-WC index (highest vs. reference category) and risks of all-cause mortality. TyG-WC, Triglyceride Glucose-Waist Circumference Index.

(A)TyG-WC index (per 1-SD increase)

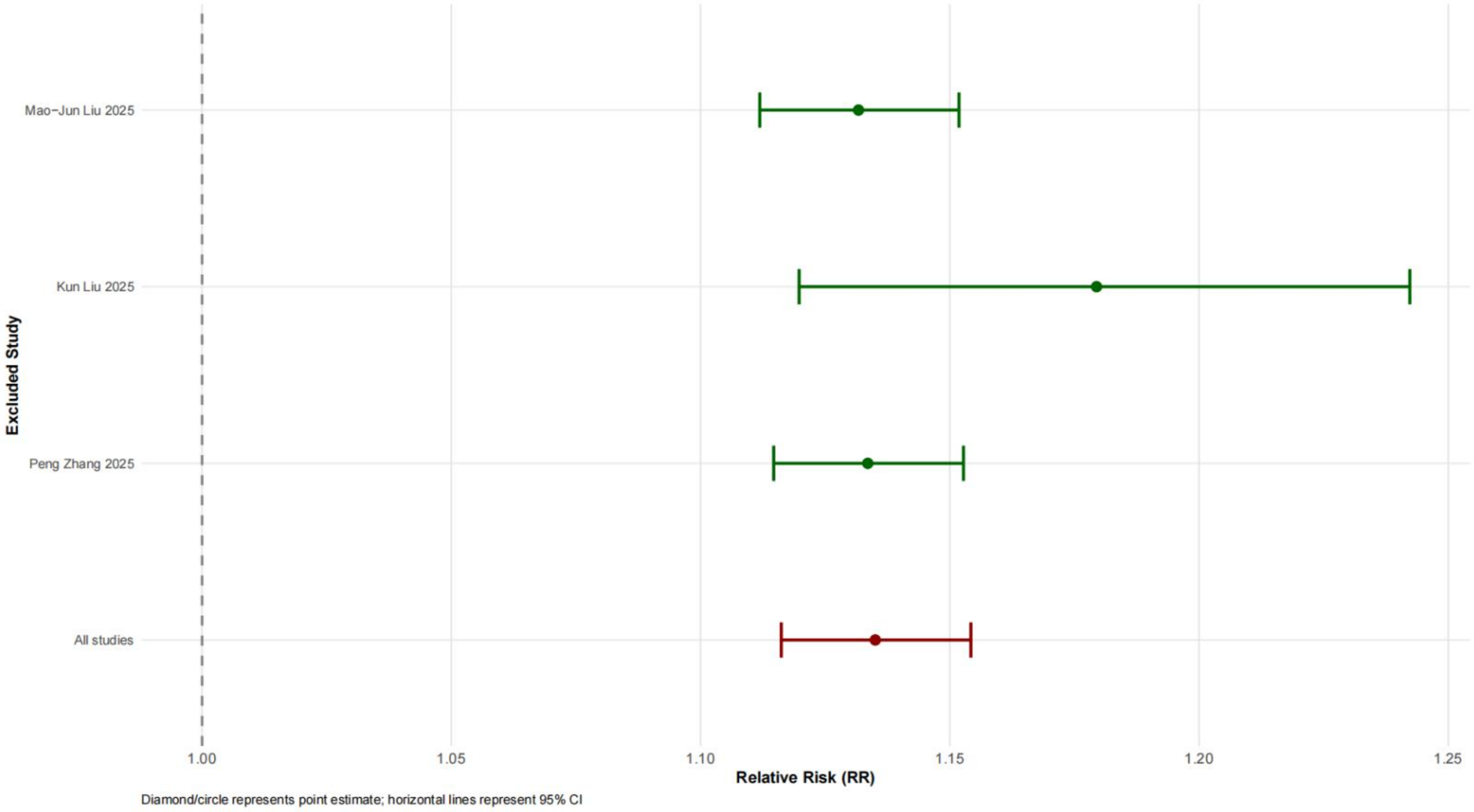

(B) Categorised TyG-WC index

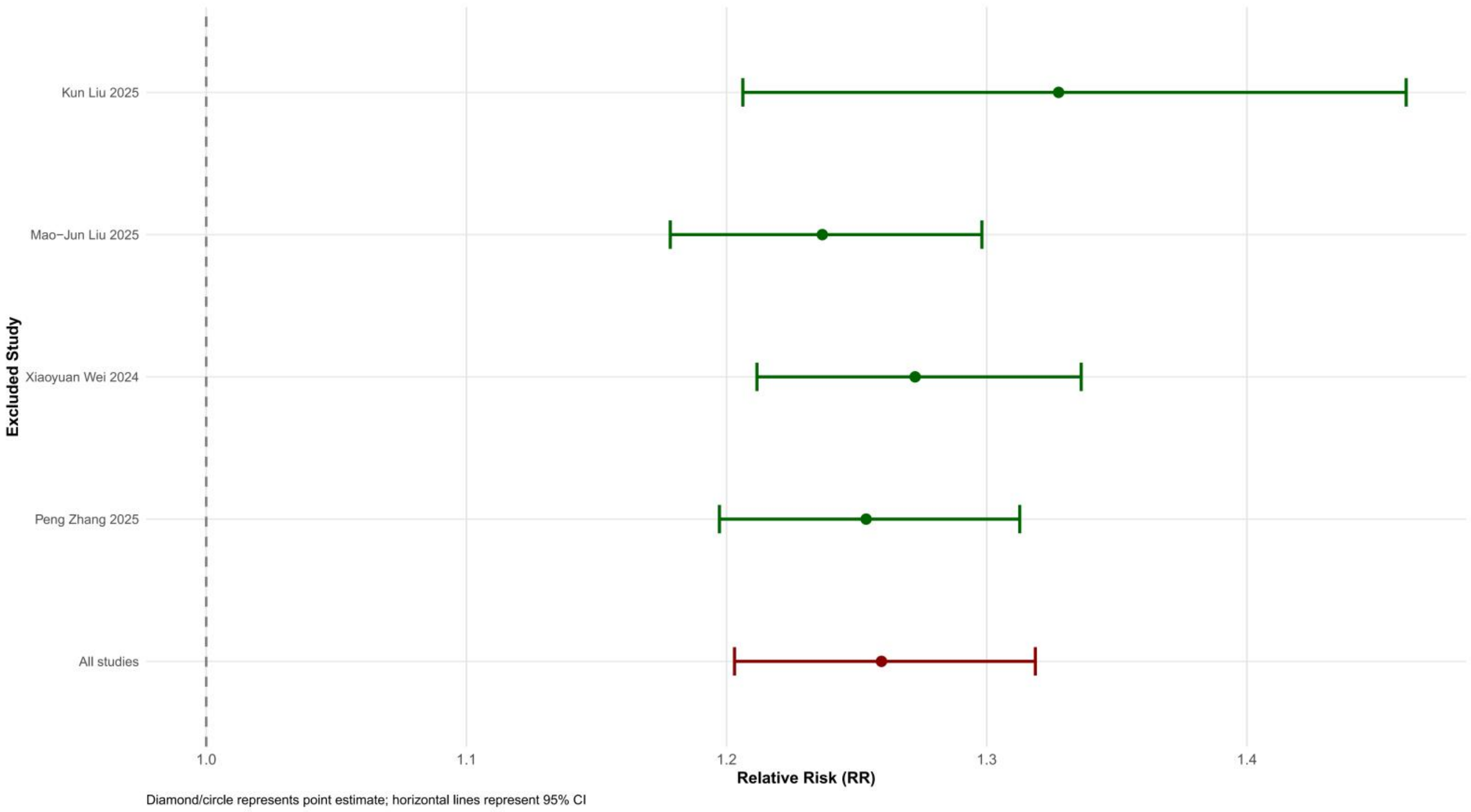

Figure S24. Leave-one-out analysis for the association between TyG-WC index (per 1-SD increase, (B) categorised TyG-WC index (highest vs. reference category) and risks of CVD mortality. TyG-WC, Triglyceride Glucose-Waist Circumference Index. CVD, Cardiovascular Disease.

(A)TyG-WC index (per 1-SD increase)

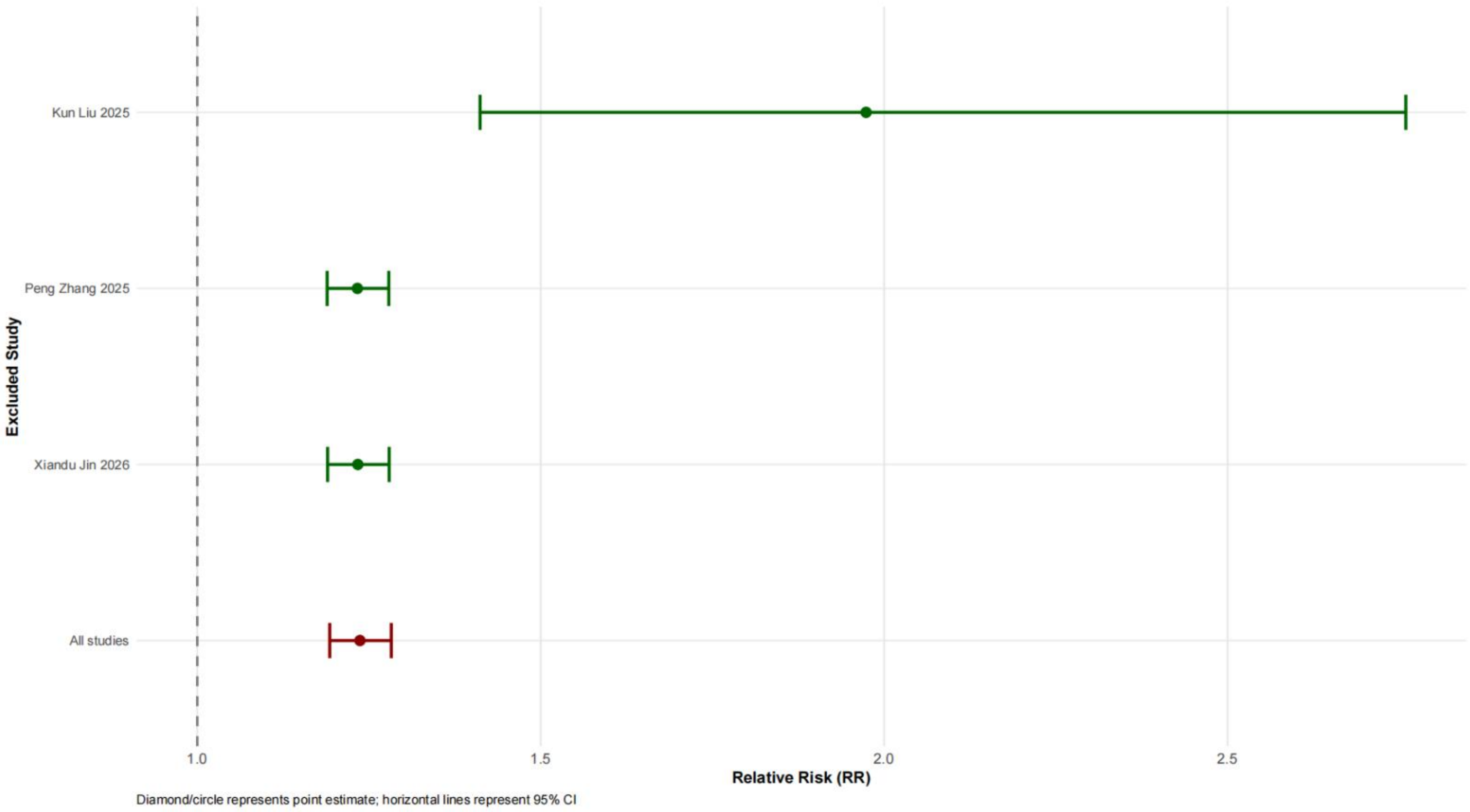

(B) Categorised TyG-WC index

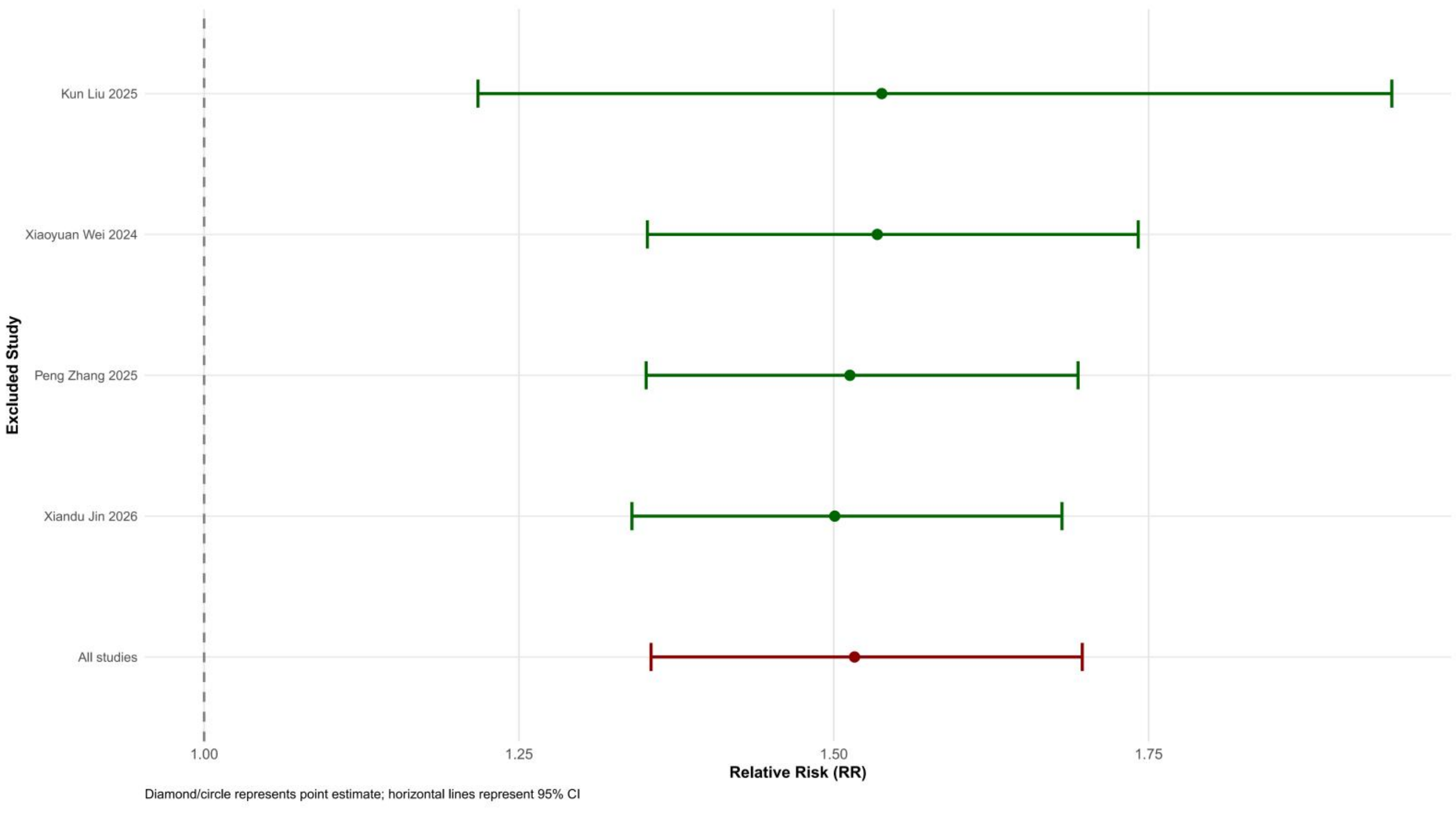

Figure S25. Leave-one-out analysis for the association between TyG-WC index (per 1-SD increase), (B) categorised TyG-WC index (highest vs. reference category) and risks of CVD incidence. TyG-WC, Triglyceride Glucose-Waist Circumference Index. CVD, Cardiovascular Disease.

(A)TyG-WC index (per 1-SD increase)

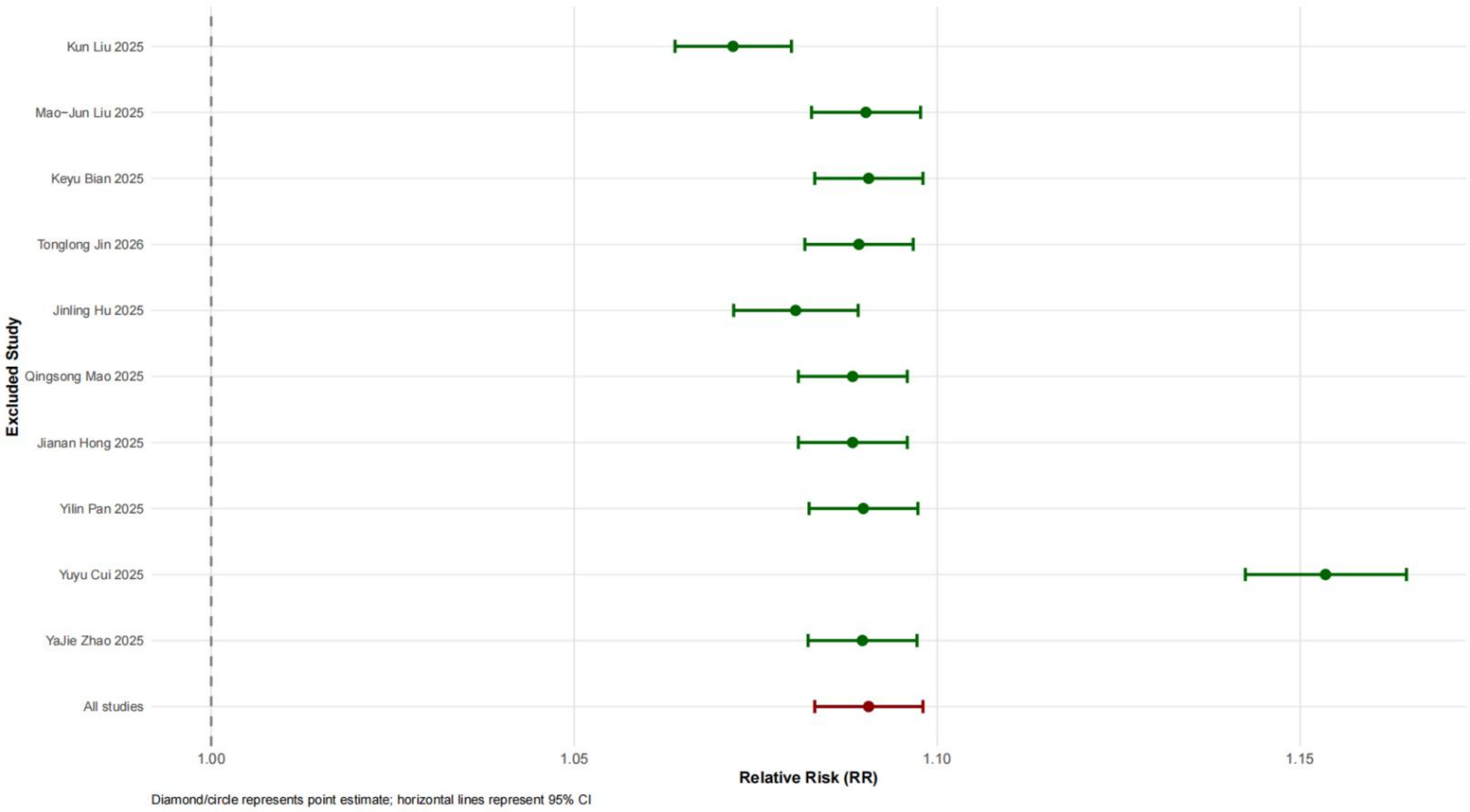

(B) Categorised TyG-WC index

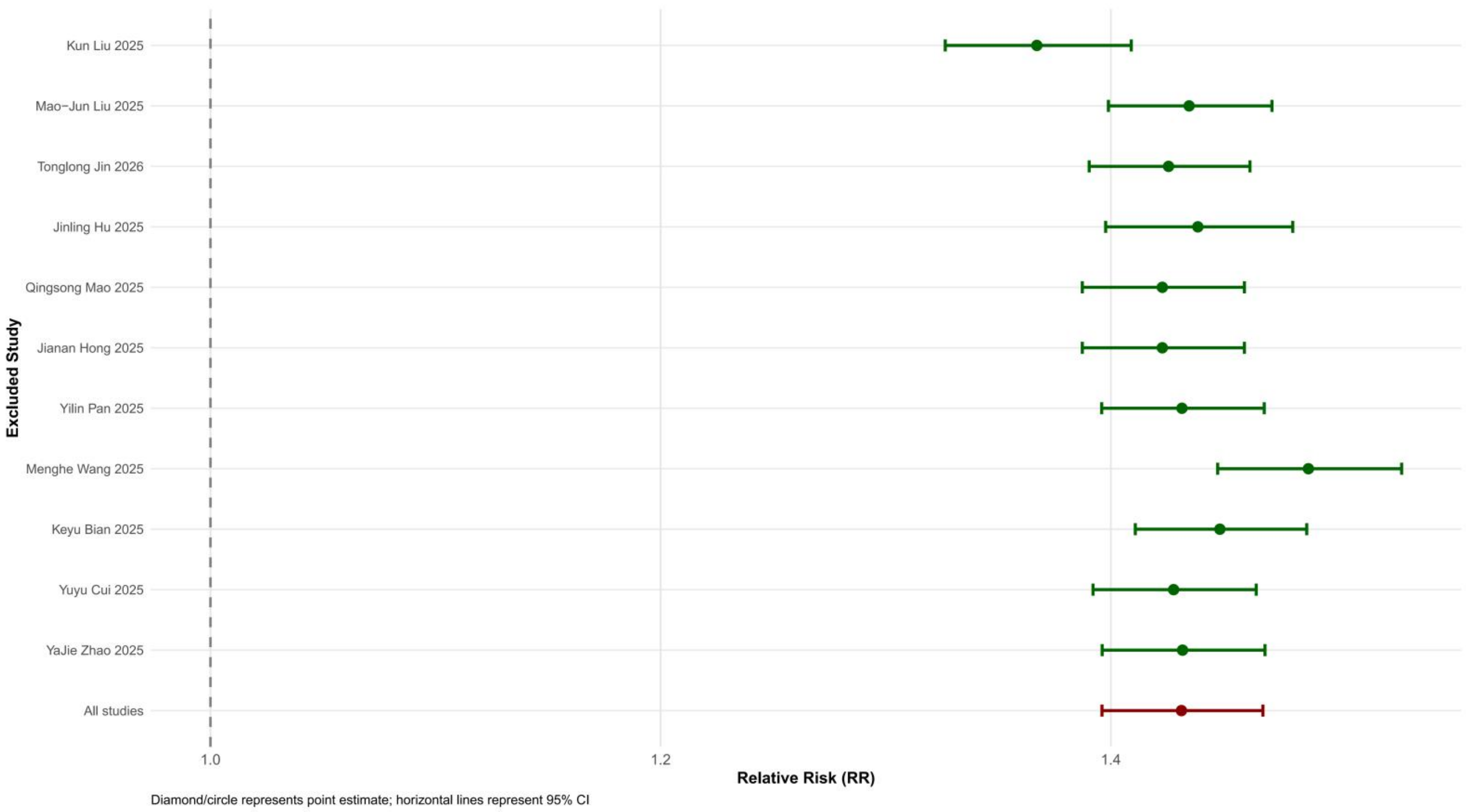

Figure S26. Leave-one-out analysis for the association between TyG-WC index (per 1-SD increase), (B) categorised TyG-WC index (highest vs. reference category) and risks of stroke incidence. TyG-WC, Triglyceride Glucose-Waist Circumference Index.

(A)TyG-WC index (per 1-SD increase)

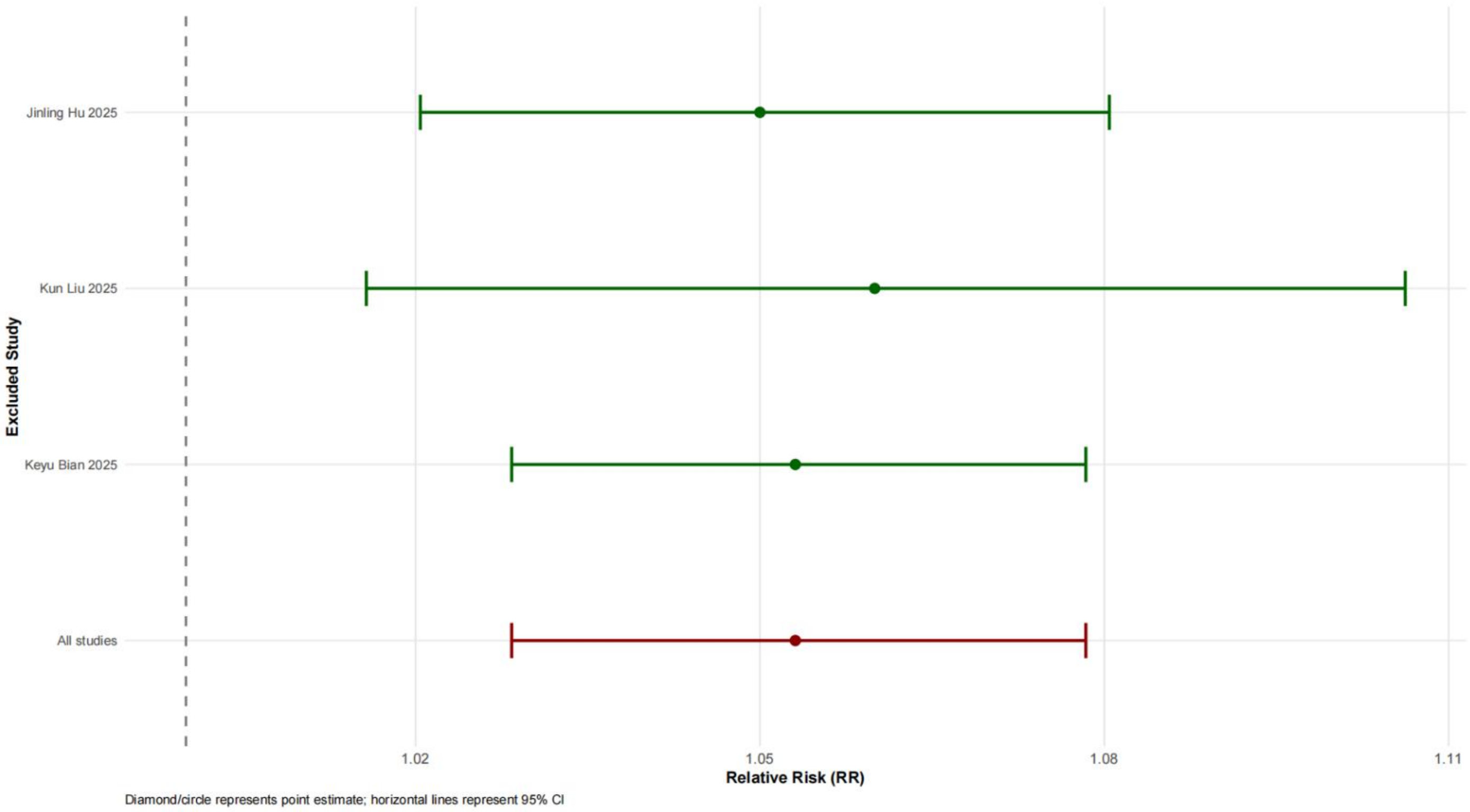

(B) Categorised TyG-WC index

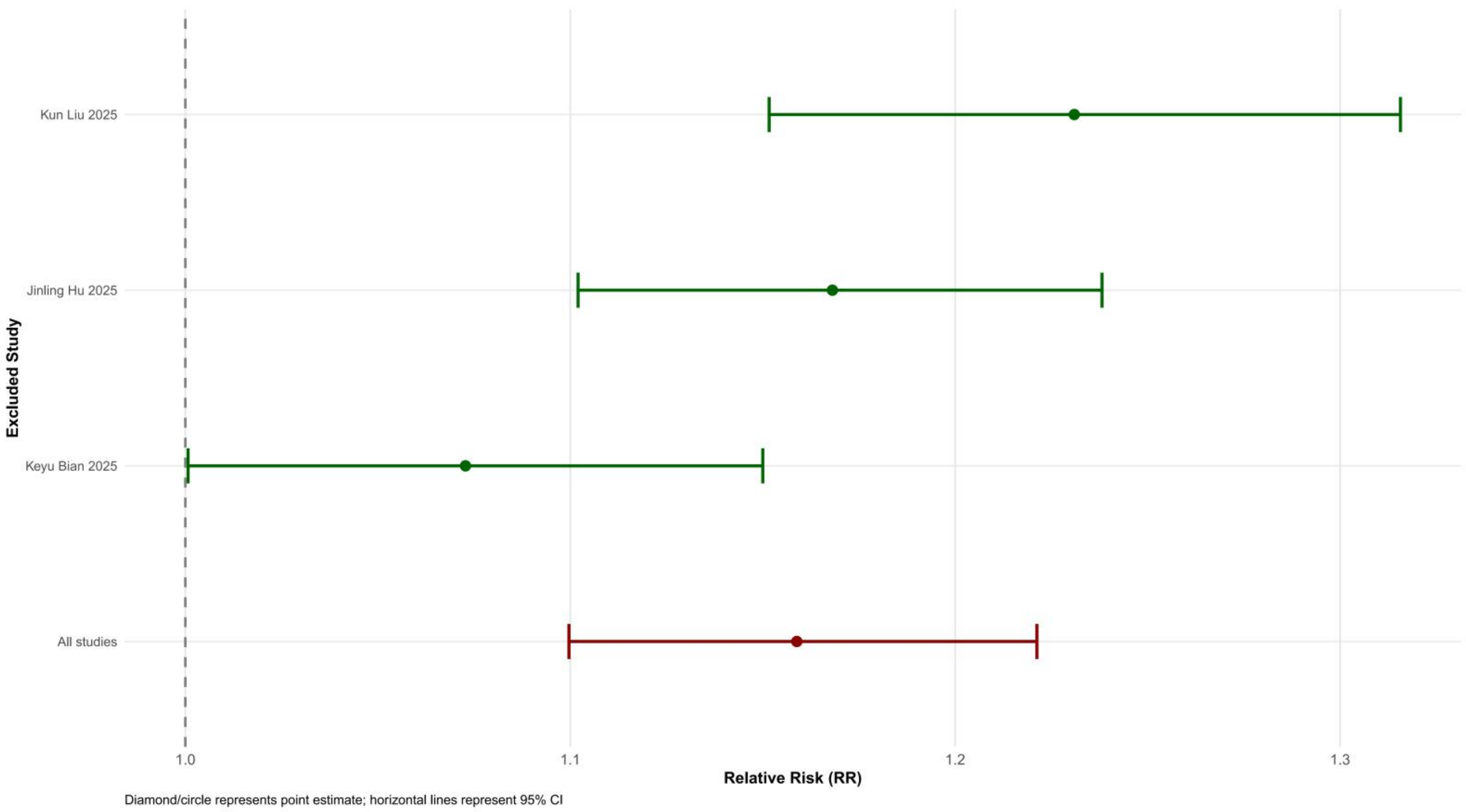

Figure S27. Leave-one-out analysis for the association between TyG-WHtR index (per 1-SD increase), (B) categorised TyG-WHtR index (highest vs. reference category) and risks of all-cause mortality. TyG-WHtR, Triglyceride–Glucose Waist-to-Height Ratio Index.

(A)TyG-WHtR index (per 1-SD increase)

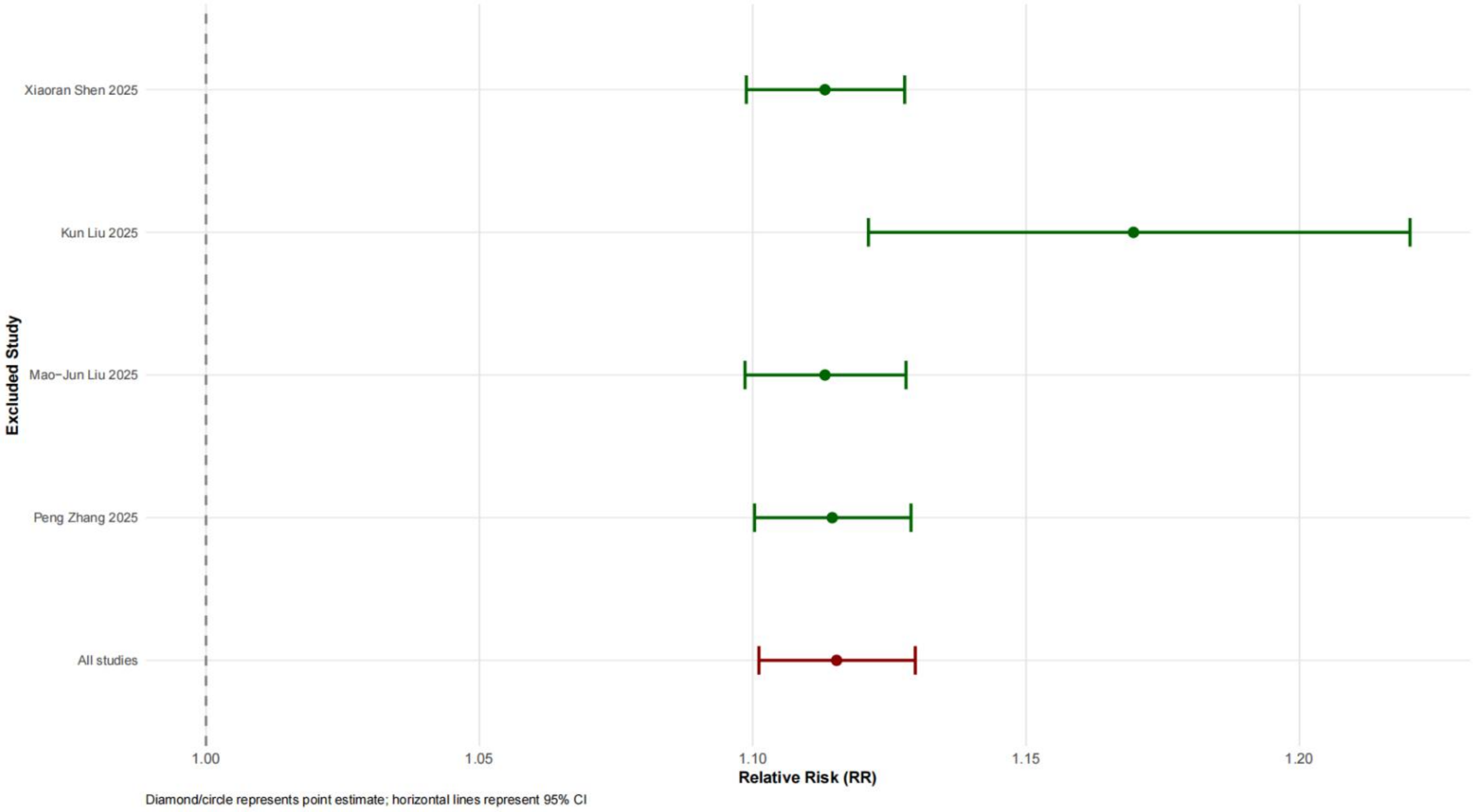

(B) Categorised TyG-WHtR index

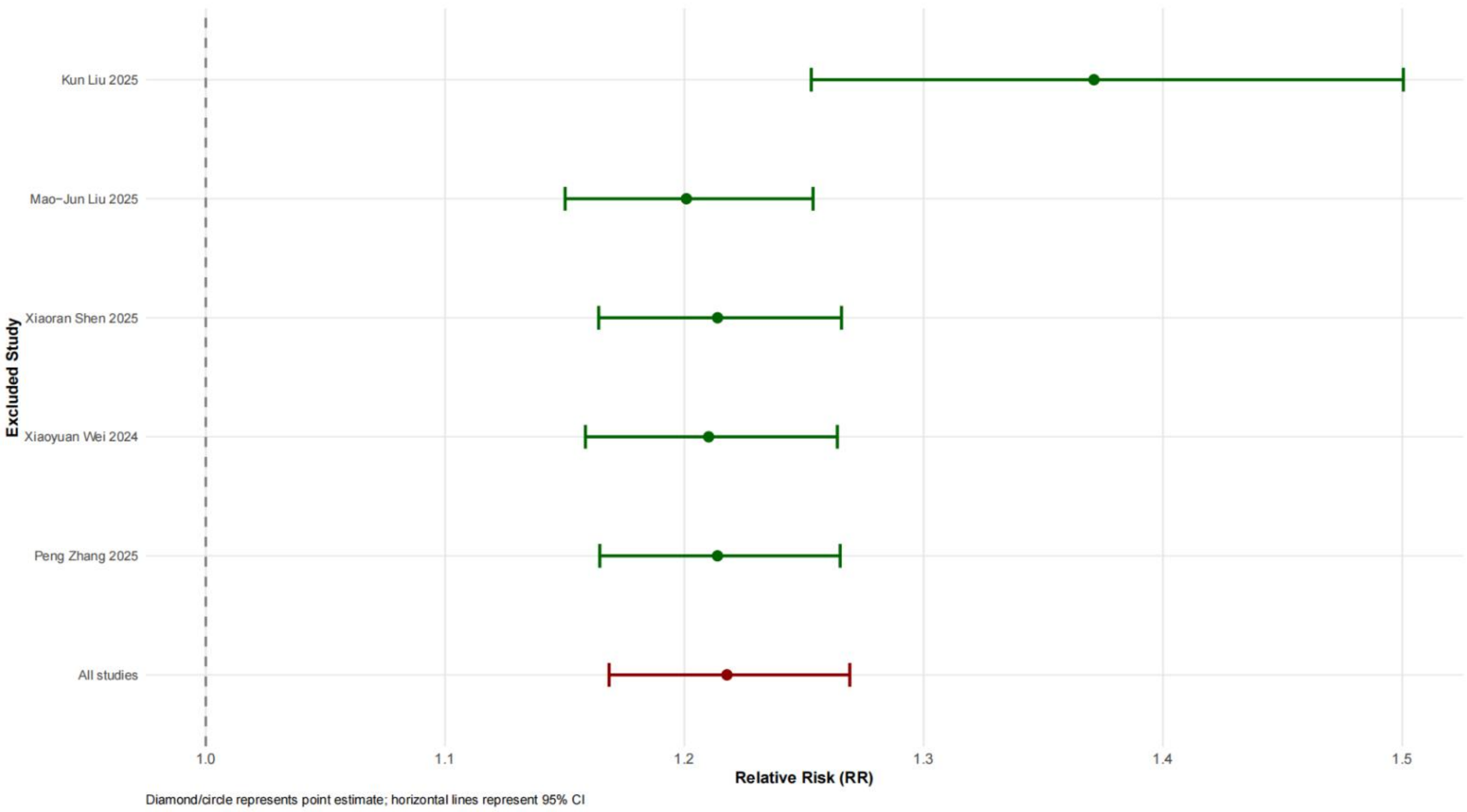

Figure S28. Leave-one-out analysis for the association between TyG-WHtR index (per 1-SD increase), (B) categorised TyG-WHtR index (highest vs. reference category) and risks of CVD mortality. TyG-WHtR, Triglyceride–Glucose Waist-to-Height Ratio Index. CVD, Cardiovascular Disease.

(A)TyG-WHtR index (per 1-SD increase)

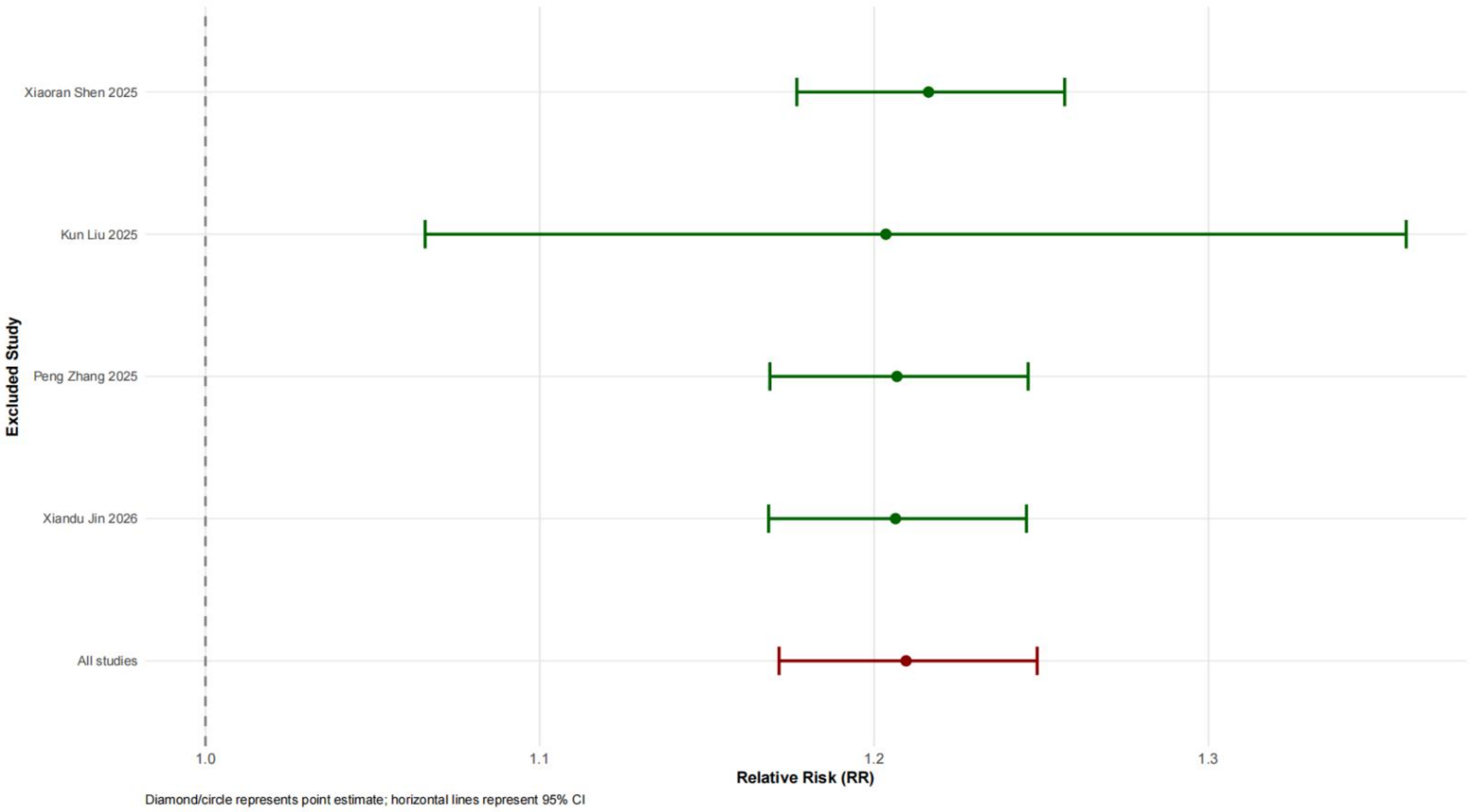

(B) Categorised TyG-WHtR index

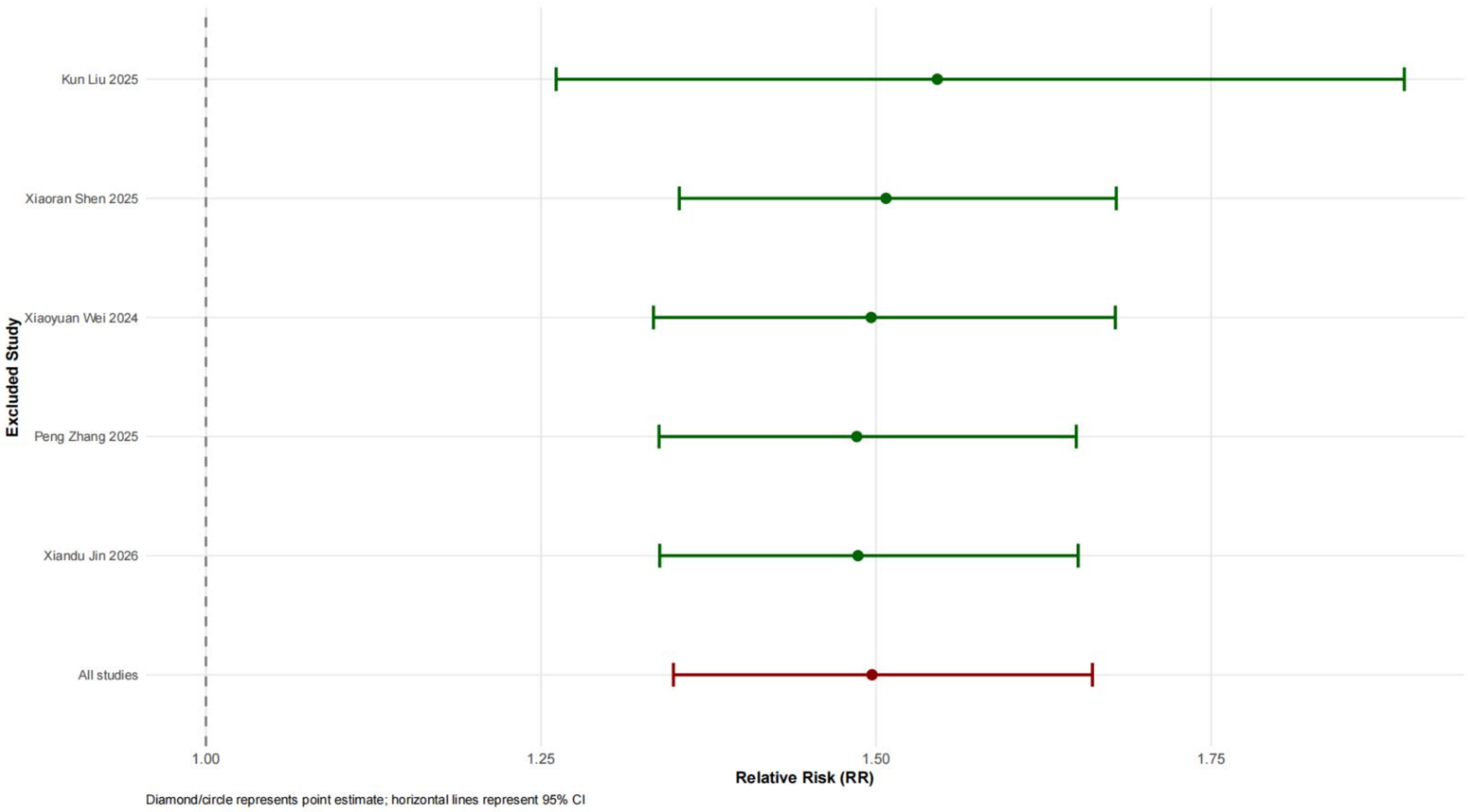

Figure S29. Leave-one-out analysis for the association between TyG-WHtR index (per 1-SD increase), (B) categorised TyG-WHtR index (highest vs. reference category) and risks of CVD incidence. TyG-WHtR, Triglyceride–Glucose Waist-to-Height Ratio Index. CVD, Cardiovascular Disease.

(A)TyG-WHtR index (per 1-SD increase)

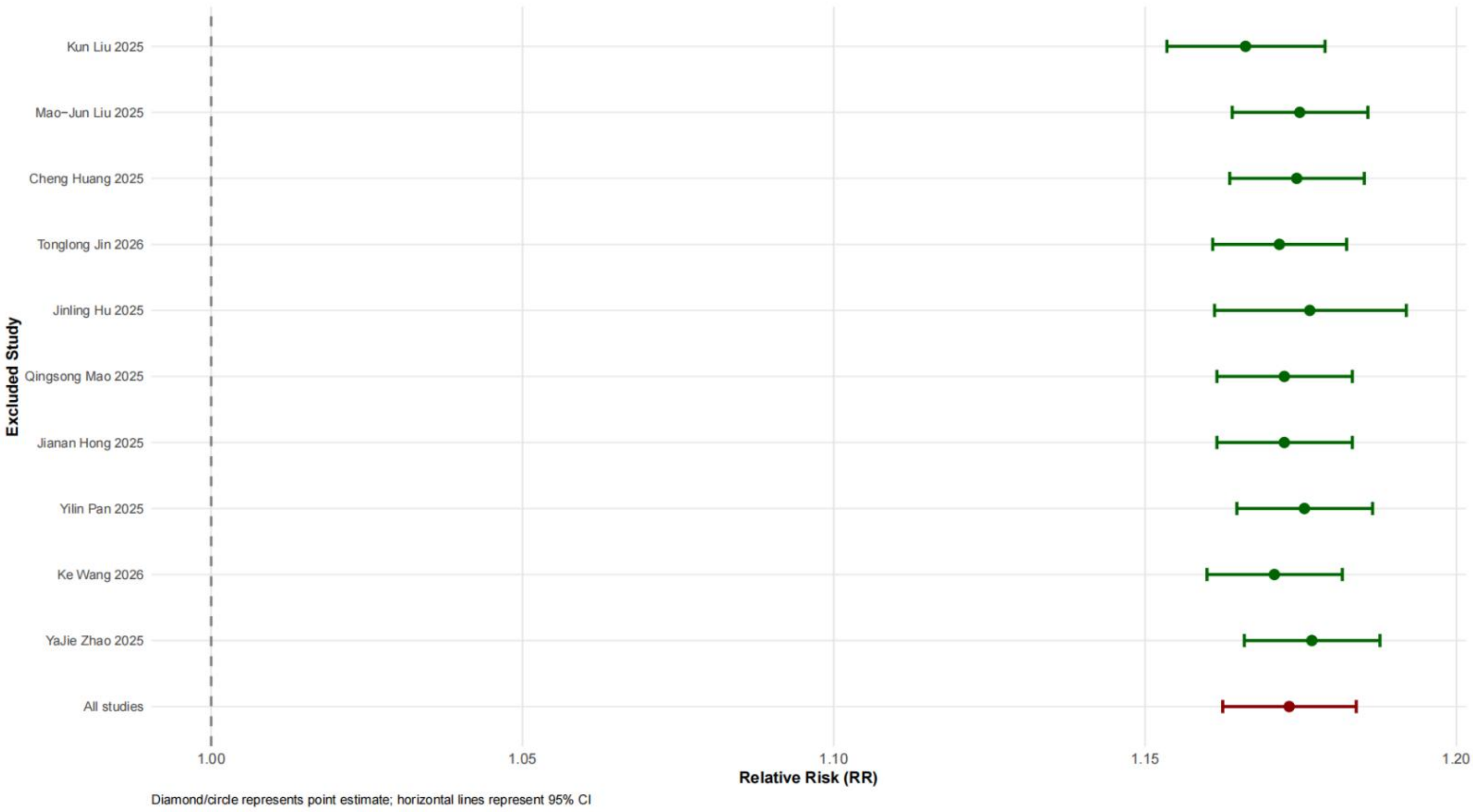

(B) Categorised TyG-WHtR index

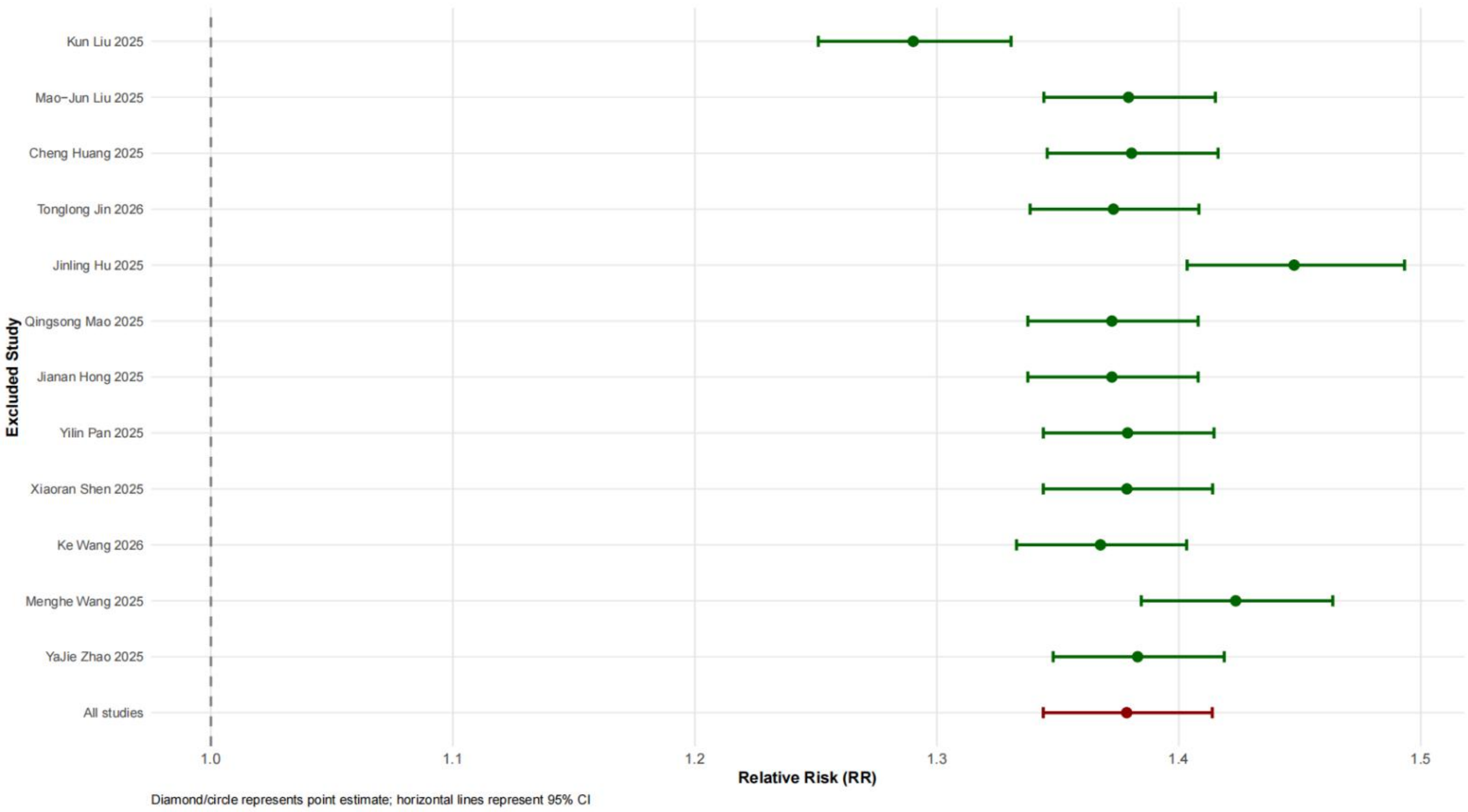

Figure S30. Leave-one-out analysis for the association between CTI index (per 1-SD increase), (B) categorised CTI index (highest vs. reference category) and risks of CVD incidence. CTI, C-Reactive Protein-Triglyceride Glucose Index. CVD, Cardiovascular Disease.

(A)CTI index (per 1-SD increase)

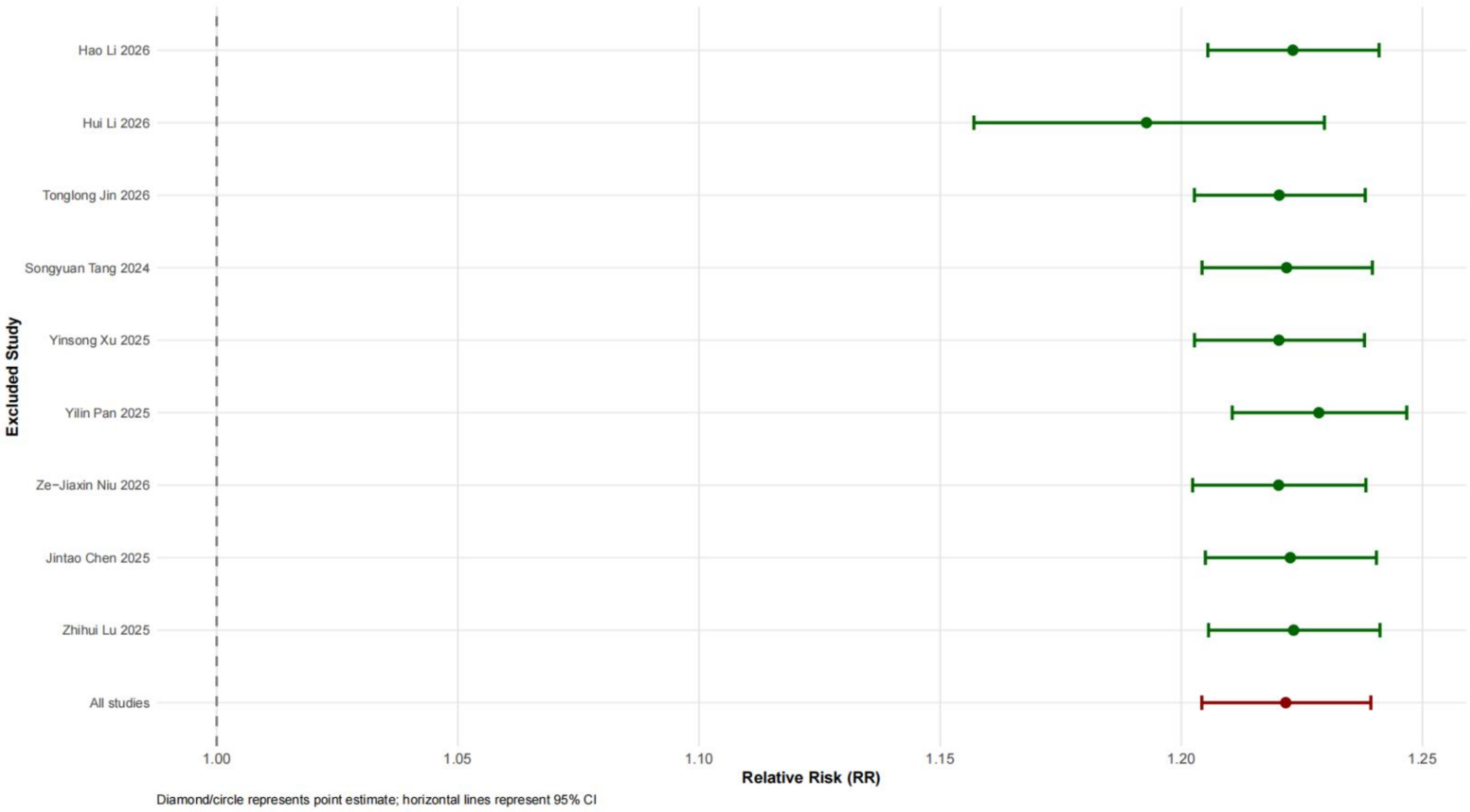

(B) Categorised CTI index

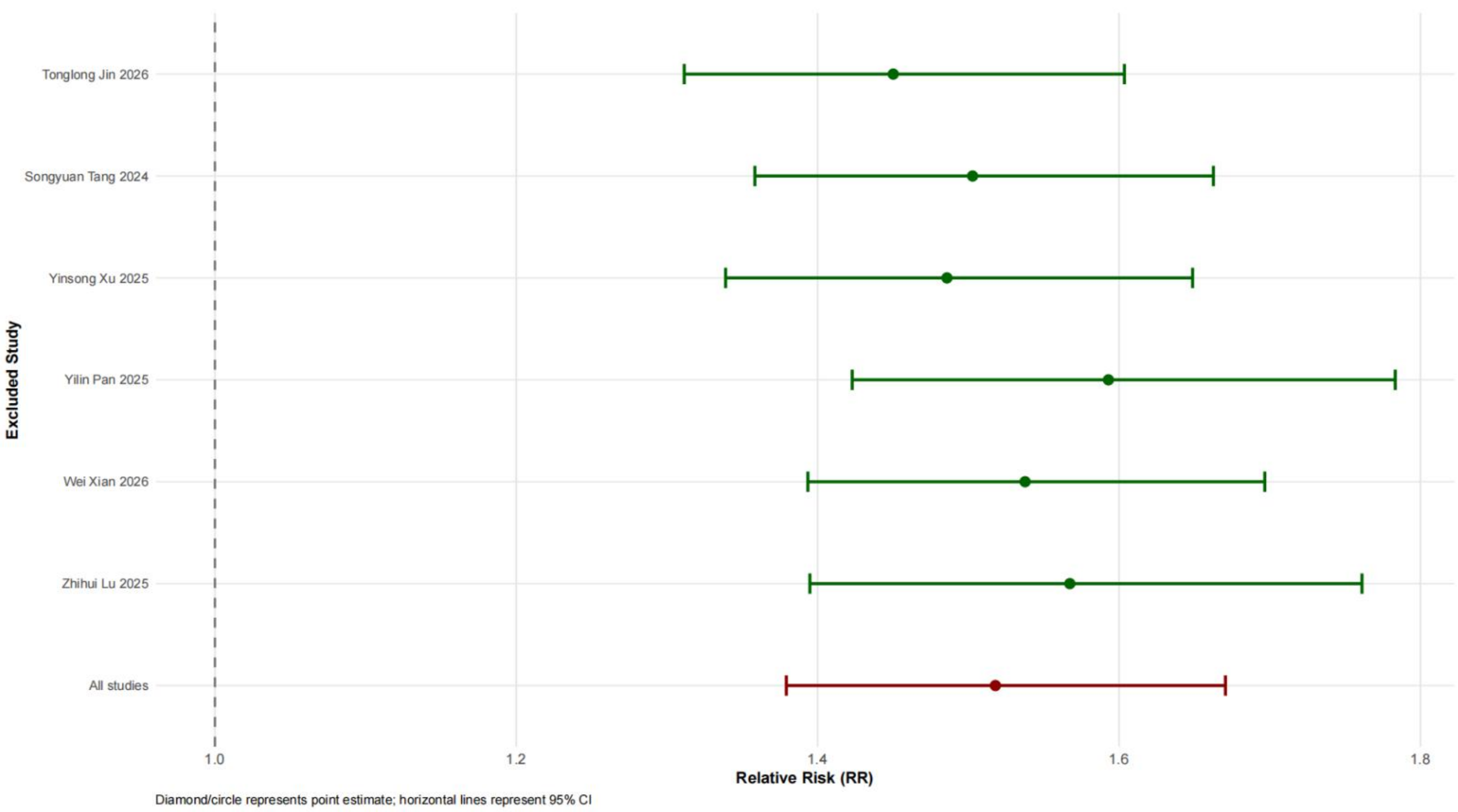

Figure S31. Leave-one-out analysis for the association between CTI index (per 1-SD increase), (B) categorised CTI index (highest vs. reference category) and risks of stroke incidence. CTI, C-Reactive Protein-Triglyceride Glucose Index.

(A)CTI index (per 1-SD increase)

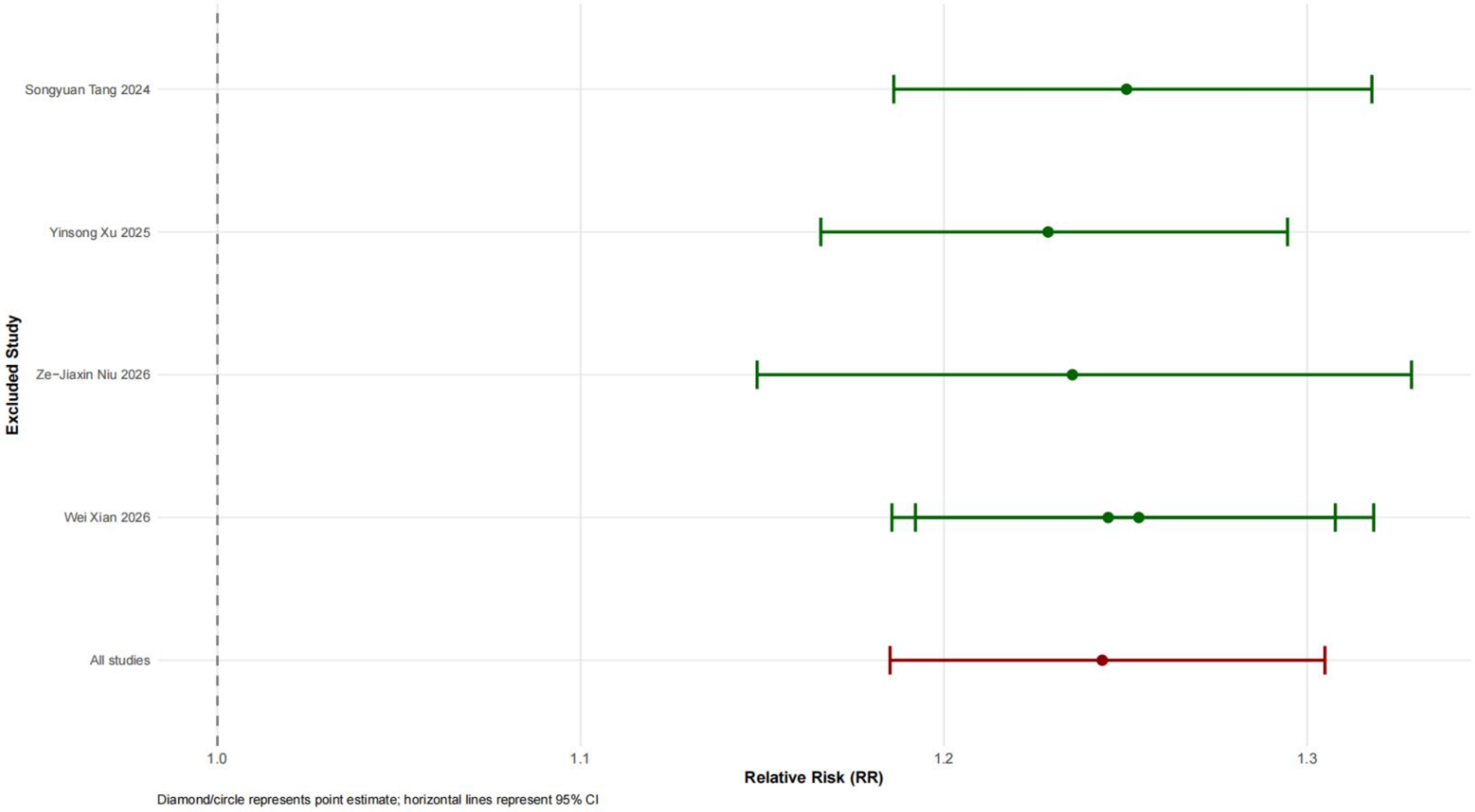

(B) Categorised CTI index

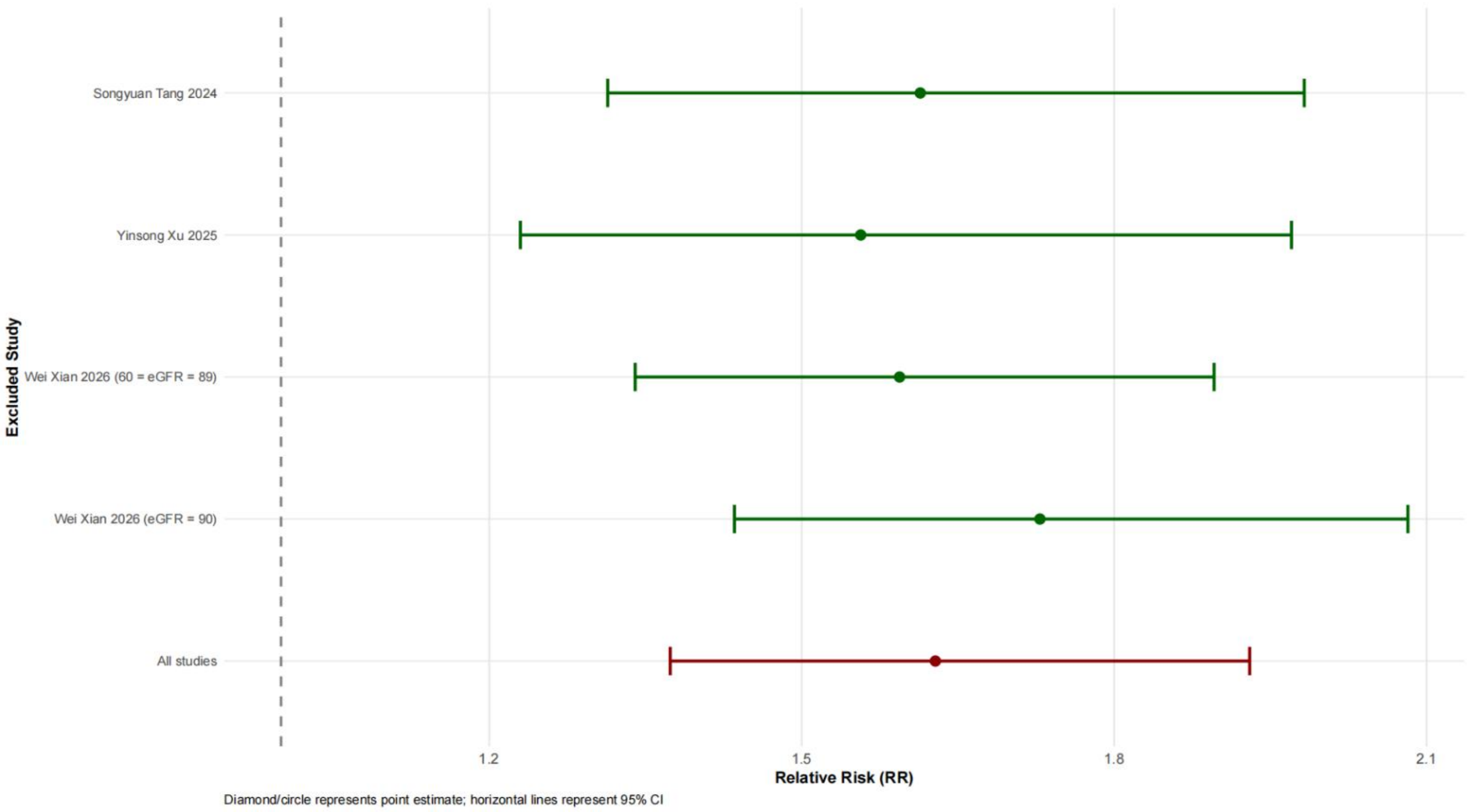

Figure 32. Funnel plot and trimfill funnel plot for the association between TyG index (per 1-SD increase), (B) categorised TyG index (highest vs. reference category) and all-cause mortality, CVD mortality and CVD incidence.

(A) TyG index (per 1-SD increase)

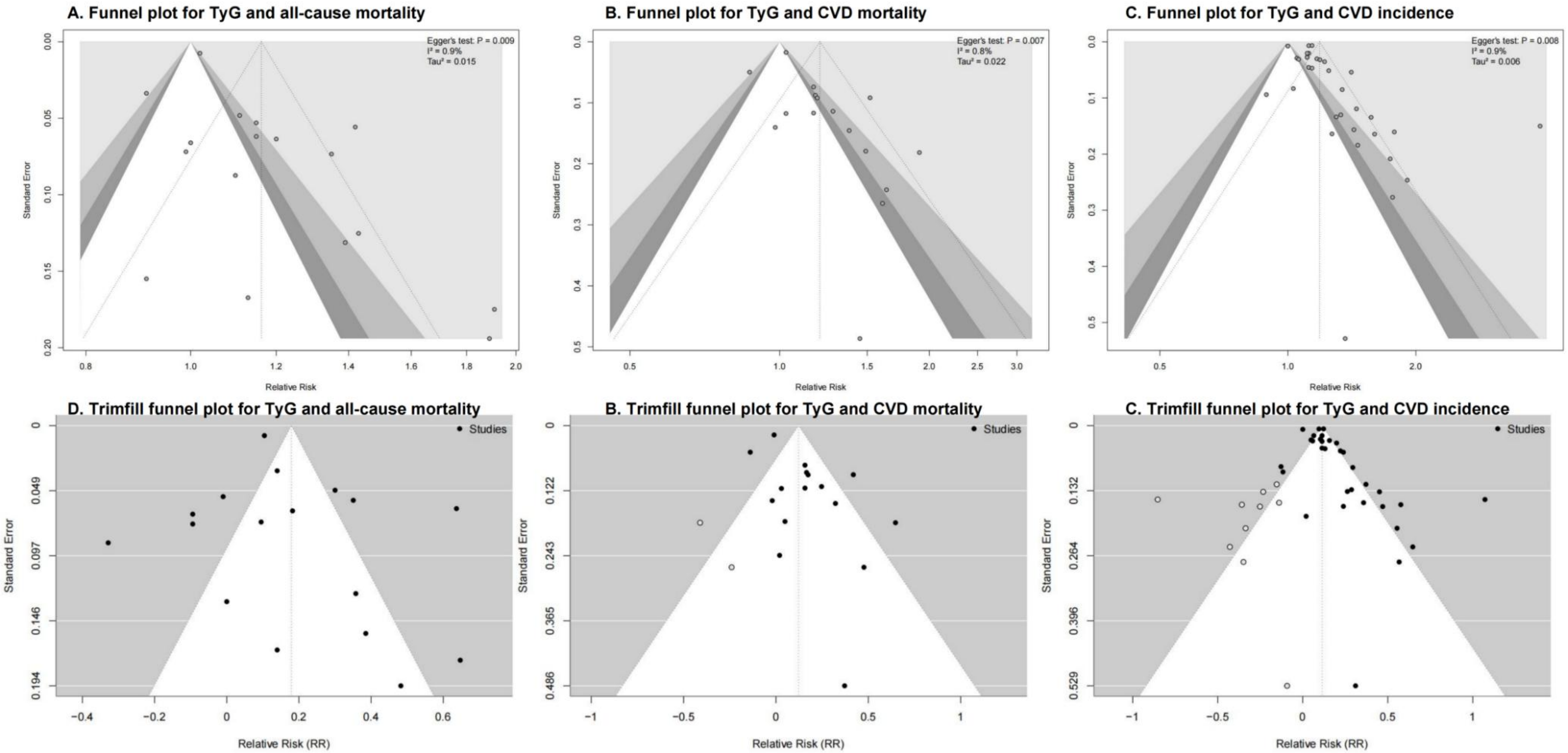

D. TyG and all-cause moratlity: Original pooled RR=1.196, Trim and fill adjusted RR=1.196, Number of missing studies=0, Total number of studies (after imputation)=21, Adjusted Z value=2.8499, Adjusted P value=0.0044, Publication bias present=No

E. Original pooled RR=1.167, Trim and fill adjusted RR=1.1298, Number of missing studies=2, Total number of studies (after imputation)=18, Adjusted Z value=2.2611, Adjusted P value=0.0238, Publication bias present=Yes

F. Original pooled RR=1.2169, Trim and fill adjusted RR=1.1212, Number of missing studies=10, Total number of studies (after imputation) =44, Adjusted Z value=2.5056, Adjusted P value=0.0122, Publication bias present=Yes

(B) Categorised TyG index

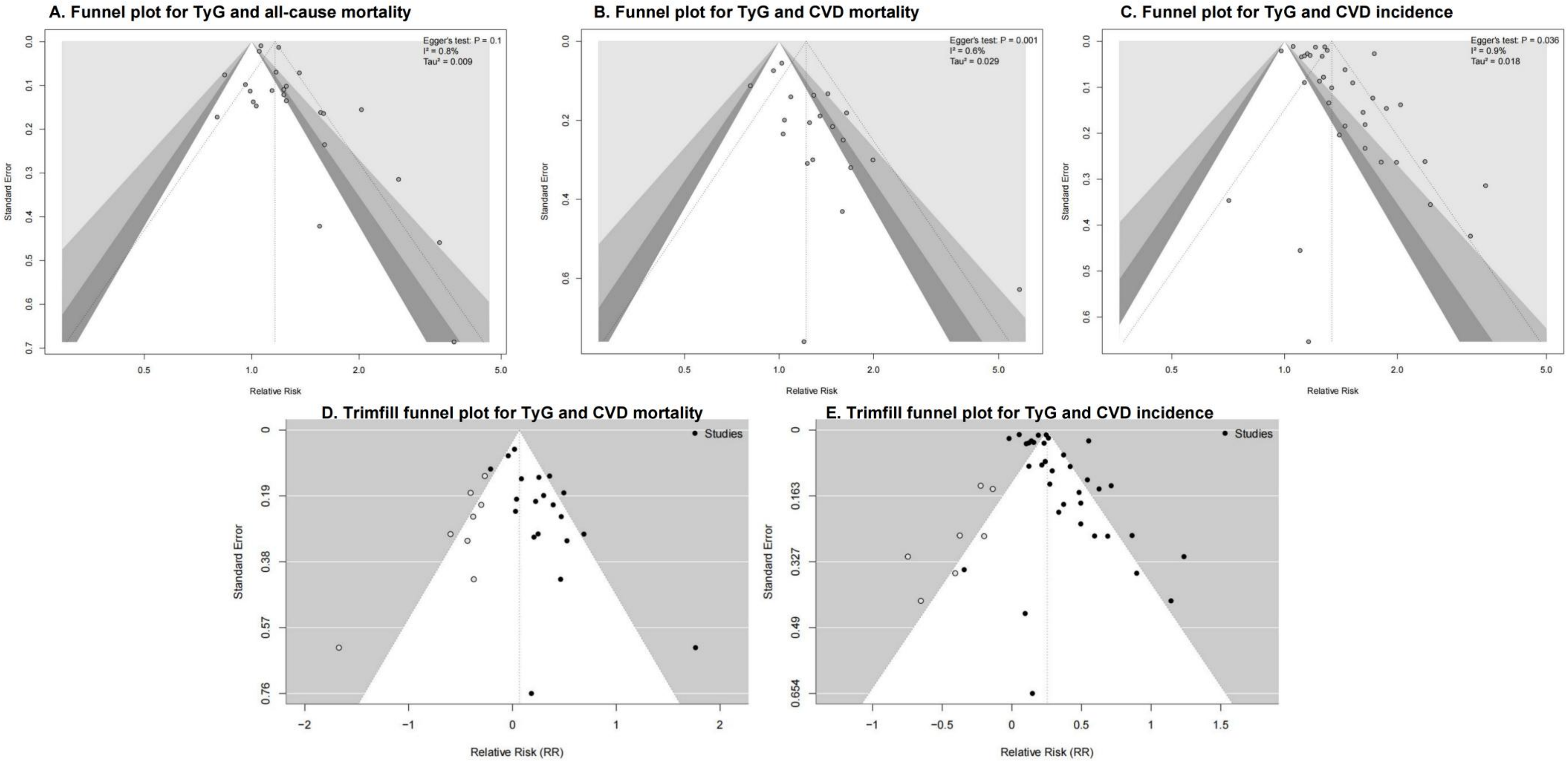

D. Original pooled RR=1.2235, Trim and fill adjusted RR=1.0678, Number of missing studies=8, Total number of studies (after imputation)=28, Adjusted Z value=0.9857, Adjusted P value=0.3243, Publication bias present=Yes

E. Original pooled RR=1.3628, Trim and fill adjusted RR=1.2891, Number of missing studies=7, Total number of studies (after imputation)=43, Adjusted Z value=5.9462, Adjusted P value=0, Publication bias present=Yes

Table S1. Search strategy

**PubMed (15,560 results)**
("Triglyceride-glucose index" OR "triglyceride glucose index" OR "triglycerides and glucose index" OR "TyG" OR "TyG index" OR "TyG-BMI" OR "TyG-body mass index" OR "TyG-waist circumference" OR "TyG-WC" OR "TyG-WHtR" OR "TyG-waist-to-height ratio" OR "Cardiometabolic index" OR "CMI" OR "C-reactive protein-triglyceride glucose index" OR "CRP-TyG" OR "CTI")
AND
(mortality OR death OR survival OR incidence OR occurrence OR prognosis OR prognostic OR predict* OR "risk stratification" OR "risk assessment" OR "major adverse cardiovascular event*" OR "major adverse cardiac event*" OR MACE OR "cardiovascular event*" OR "cardiac event*" OR "cardiovascular outcome*" OR "coronary heart disease" OR CHD OR "coronary artery disease" OR CAD OR "ischemic heart disease" OR "ischaemic heart disease" OR IHD OR stroke OR "cerebrovascular accident" OR CVA OR "cerebrovascular disease" OR "cerebral infarction" OR "brain infarction" OR "heart failure" OR "cardiac failure" OR "congestive heart failure" OR CHF OR "myocardial infarction" OR "acute myocardial infarction" OR AMI)
**Web of science(7,002 results)**
**Embase(8,279 results)**

Table S2. CKM Stage Classification of Included Studies. AHA, American Heart Association. BP, Blood Pressure. CAD, Coronary Artery Disease. CHARLS, China Health and Retirement Longitudinal Study. CKD, Chronic Kidney Disease. CKM, Cardiovascular-Kidney-Metabolic. CVD, Cardiovascular Disease. DM, Diabetes Mellitus. eGFR, estimated Glomerular Filtration Rate. IRCCS, Istituto di Ricovero e Cura a Carattere Scientifico. INRCA, Istituto Nazionale di Riposo e Cura per Anziani. KDIGO, Kidney Disease: Improving Global Outcomes. MetS, Metabolic Syndrome. MUH, Metabolically Unhealthy. NHANES, National Health and Nutrition Examination Survey. NHIS, National Health Insurance Service. NSTEMI, Non-ST-Elevation Myocardial Infarction. preDM, Prediabetes. SBP, Systolic Blood Pressure. T2D, Type 2 Diabetes.

Study ID	Assigned CKM Stage	Classification Basis	Character
Dan Zhou[1]	Stage 2	Patients with hypertension (CKM Stage 2)	Inferred
Zhihui Lu[2]	Stages 0-3	CKM syndrome stages 0-3 population	Explicit
Wei Xian[3]	Stages 0-3	General population (CHARLS) spanning CKM 0-3, stratified by eGFR (CKD=Stage 2-3)	Inferred
Jintao Chen[4]	Stages 0-3	CKM syndrome stages 0-3 population	Explicit
YaJie Zhao[5]	Stages 0-3	CKM syndrome stages 0-3 population	Explicit
Ze-Jiaxin Niu[6]	Stages 0-3	CKM syndrome stages 0-3 population	Explicit
Yafang Chen[7]	Stage 0-2	Individuals with various metabolic health phenotypes (healthy=Stage 0; obesity=Stage 1; MetS=Stage 2)	Inferred
Weipeng Li[8]	Stages 0-3	CKM syndrome stages 0-3 population	Explicit
Yuyu Cui[9]	Stages 0-3	General population (CHARLS) spanning CKM 0-3, stratified by glycemic status	Inferred
Chang Liu[10]	Stage 2	Patients with type 2 diabetes (CKM Stage 2)	Inferred
Xiandu Jin[11]	Stages 0-3	CKM syndrome stages 0-3 population	Explicit
Yanjuan Chen[12]	Stage 1-2	Metabolically healthy obese individuals (obesity=Stage 1; MUH conversion=Stage 2)	Inferred
Xinyi Shao[13]	Stages 0-3	General population (UK Biobank) spanning CKM 0-3, by glycemic status and coronary stenosis	Inferred
Keyu Bian[14]	Stages 0-3	General population (UK Biobank) spanning CKM 0-3, by glucose metabolism status	Inferred
Peng Zhang[15]	Stages 0-3	CKM syndrome stages 0-3 population	Explicit
Xiaoyuan Wei[16]	Stage 2	Population with metabolic syndrome (CKM Stage 2)	Inferred
Yong Yue[17]	Stages 0-3	CKM syndrome stages 0-3 population	Explicit
Chang Liu[18]	Stage 2	Patients with type 2 diabetes (CKM Stage 2)	Inferred
Mingjie Chen[19]	Stages 0-3	CKM syndrome stages 0-3 population	Explicit
Jiahao Zou[20]	Stage 2	Patients with comorbid hypertension and diabetes (CKM Stage 2)	Inferred
Byung Sik Kim[21]	Stages 0-3	CKM syndrome stages 0-3 population	Explicit
Qi-chao Sun[22]	Stage 2	Patients with type 2 diabetes (CKM Stage 2)	Inferred
Peixin Lan[23]	Stage 2	Adults with hyperlipidemia (CKM Stage 2)	Inferred
Qiyu Xiao[24]	Stage 2	Non-diabetic patients with hypertension (CKM Stage 2)	Inferred
Man Li[25]	Stage 2	Patients with diabetes and suspected CAD (CKM Stage 2)	Inferred
Menghe Wang[26]	Stages 0-3	CKM syndrome stages 0-3 population	Explicit
Yu Tang[27]	Stages 0-3	CKM syndrome stages 0-3 population	Explicit
Hui Li[28]	Stages 0-3	General population (UK Biobank) spanning CKM 0-3	Inferred

Hao Li[29]	Stage 2	Adults with abnormal glucose metabolism (preDM=Stage 1; DM=Stage 2)	Inferred
Ke Wang[30]	Stages 0-3	CKM syndrome stages 0-3 population	Explicit
Xiaoran Shen[31]	Stages 0-3	General population (NHANES) spanning CKM 0-3	
Yilin Pan[32]	Stages 0-3	CKM syndrome stages 0-3 population	Explicit
Wei-Yu Su[33]	Stage 2	Patients with type 2 diabetes (CKM Stage 2)	Inferred
Jianan Hong[34]	Stages 0-3	General population (CHARLS) spanning CKM 0-3	Inferred
Qingsong Mao[35]	Stages 0-3	General population (CHARLS) spanning CKM 0-3	Inferred
Yinsong Xu[36]	Stages 0-3	CKM syndrome stages 0-3 population	Explicit
Huiwen Ou[37]	Stages 0-3	CKM syndrome stages 0-3 population	Explicit
Qianliang Ying[38]	Stages 0-3	CKM syndrome stages 0-3 population	Explicit
Songyuan Tang[39]	Stage 2	Patients with hypertension (CKM Stage 2)	Inferred
Yulong Lan[40]	Stage 2	Patients with hypertension (CKM Stage 2)	Inferred
Shi Tai[41]	Stage 2	Patients with type 2 diabetes (CKM Stage 2)	Inferred
Tao Yi[42]	Stages 0-3	General population (NHANES) spanning CKM 0-3, by metabolic health and obesity	Inferred
Xin Chen[43]	Stage 2	Non-diabetic hypertension patients (CKM Stage 2)	Inferred
Anthony Diwon[44]	Stage 2	Patients with T2D and overweight/obesity (diabetes=Stage 2; obesity=Stage 1)	Inferred
Jinling Hu[45]	Stage 1-2	Individuals with preclinical/clinical obesity (obesity=Stage 1-2)	Inferred
Chuanwei Zhao[46]	Stage 2	CKM syndrome stage 2 population	Explicit
Xiaoling Chen[47]	Stage 2	Hypertensive patients with controlled BP (CKM Stage 2)	Inferred
Feng-Ching Shen[48]	Stage 3	Patients with CKD (moderate-very high risk=CKM Stage 2-3)	Inferred
Claudia R. L. Cardoso[49]	Stage 2	Patients with type 2 diabetes (CKM Stage 2)	Inferred
Tonglong Jin[50]	Stage 2	Patients with cardiometabolic multimorbidity (CKM Stage 2)	Inferred
Weida Qiu[51]	Stages 0-3	General population (CHARLS) spanning CKM 0-3, stratified by SBP	Inferred
Yanling Yang[52]	Stage 2	Patients with type 2 diabetes (CKM Stage 2)	Inferred
Jiajun Liu[53]	Stages 0-3	General population (NHANES) spanning CKM 0-3, by glucose metabolism	Inferred
Younan Yao[54]	Stage 2	Patients with type 2 diabetes (CKM Stage 2)	Inferred
Kexin Wang[55]	Stages 0-3	General population (NHANES) spanning CKM 0-3	Inferred
Lei Ding[56]	Stage 2	Elderly with diabetes/prediabetes (preDM=Stage 1; DM=Stage 2)	Inferred
Cheng Huang[57]	Stages 0-3	General population (CHARLS) spanning CKM 0-3	Inferred
Jianyong Zhao[58]	Stage 2	Patients with T2D and NSTEMI (CKM Stage 2)	Inferred
Xi Chen[59]	Stage 2	Young/middle-aged with NSTEMI (CKM Stage 2)	Inferred
Xingyu Zhou[60]	Stages 0-3	CKM syndrome stages 0-3 population	Explicit
Qin Zhang[61]	Stage 2	Patients with diabetes/prediabetes and CVD (CKM Stage 1-2)	Inferred
Shu Yang[62]	Stage 2	Patients with type 2 diabetes (CKM Stage 2)	Inferred

Zhengwen Xu[63]	Stage 2	Patients with H-type hypertension (CKM Stage 2)	Inferred
Mengjie Zhao[64]	Stage 2	Patients with type 2 diabetes (CKM Stage 2)	Inferred
Fomin Zhang[65]	Stage 2	Patients with hypertension (CKM Stage 2)	Inferred
Matilde Sbriscia[66]	Stages 0-3	General population (IRCCS INRCA, Italy) spanning CKM 0-3	Inferred
Lian Wang[67]	Stage 2	Patients with H-type hypertension (CKM Stage 2)	Inferred
Mao-Jun Liu[68]	Stage 2	Patients with type 2 diabetes (CKM Stage 2)	Inferred
Peng Ning[69]	Stage 2	Patients with type 2 diabetes (CKM Stage 2)	Inferred
Yun Kyung Cho[70]	Stage 1-2	Metabolically unhealthy obese population (CKM Stage 1-2)	Inferred
Mao-Jun Liu[71]	Stage 2	Patients with type 2 diabetes (CKM Stage 2)	Inferred
Kun Liu[72]	Stages 0-3	CKM syndrome stages 0-3 population	Explicit
Yumeng Shi[73]	Stage 2	Patients with diabetes and prediabetes (preDM=Stage 1; DM=Stage 2)	Inferred
Hui-juan Li[74]	Stage 1-2	Patients with intermediate coronary stenosis (CKM Stage 1-2)	Inferred
Shuping Yang[75]	Stage 2	Patients with DM or preDM (preDM=Stage 1; DM=Stage 2)	Inferred
Jeongeun Kwak[76]	Stages 0-3	General population (Korean NHIS) spanning CKM 0-3, by diabetes status	Inferred
Haipeng Yao[77]	Stage 2	Patients with type 2 diabetes (CKM Stage 2)	Inferred
Chen Li[78]	Stage 2	Patients with hypertension (CKM Stage 2)	Inferred
Nan Zhang[79]	Stage 2	Patients with hypertension (CKM Stage 2)	Inferred
Jiajun Liu[80]	Stage 1-2	Patients with metabolic syndrome (CKM Stage 1-2)	Inferred

Table S3. Characteristics of 80 included cohorts.

(A). Baseline characteristics

First Author	year	Country	Study name	Study design	Follow up (years)	Male (%)	Age (years)	Ethnic	Indicators	Outcome Classification
Dan Zhou[1]	2022	USA	NHANES	Retrospective	NA	49.29%	60.12±16.07	Mexican American, Black, White, others	TyG	CVD mortality, All-cause mortality
Zhihui Lu[2]	2025	China	CHARLS	Prospective	10.00	47.62%	59.01±9.37	Chinese	CTI	Incident CVD
Wei Xian[3]	2026	China	CHARLS	Prospective	7.00	46.50%	59.48±9.41	Chinese	CTI	Incident stroke
Jintao Chen[4]	2025	China	CHARLS	Prospective	3.00	45.70%	61.78±8.68	Chinese	CTI, TyG	Incident CVD
YaJie Zhao[5]	2025	China	China Multi-Ethnic Cohort (CMEC) study - Chengdu cohort	Prospective	4.30	44.80%	50.4±12.0	Chinese	TyG, TyG-BMI, TyG-WC, TyG-WHtR	Incident CVD
Ze-Jiaxin Niu[6]	2026	China	CHARLS	Prospective	9.00	52.22%	58.74±9.25	Chinese	CTI	Incident stroke
Yafang Chen[7]	2025	China	participants at Tianjin Union Medical Center	Cross-sectional	NA	48.30%	63.5	Chinese	CTI	Incident CHD
Weipeng Li[8]	2024	China	CHARLS	Prospective	7.00	47.38%	59.17±9.28	Chinese	TyG-BMI	Incident CVD
Yuyu Cui[9]	2025	China	CHARLS	Prospective	8.25	47.11%	65.91±9.67	Chinese	TyG-WC	Incident CVD
Chang Liu[10]	2024	China	NHANES	Prospective	8.20	52.92%	51.14±0.28	Mexican American, Black, White, others	TyG	All-cause mortality, CVD mortality
Xiandu Jin[11]	2026	USA	NHANES	Cross-sectional	NA	47.47%	44.01	Mexican American, Black,	TyG, TyG-BMI, TyG-WC,	CVD mortality

								White, others	and TyG-WHtR	
Yanjuan Chen[12]	2025	China	Kailuan Study	Prospective	11.00	75.00%	53.70±12.93	Chinese	TyG	Incident CHD, Incident stroke, Other
Xinyi Shao[13]	2025	UK	UK Biobank	Prospective	14.80	NA	55	White, others	TyG, TyG-BMI, TyG-WHtR,	Incident CVD
Keyu Bian[14]	2025	UK	UK Biobank	Prospective	14.68	46.00%	58	White, others	TyG, TyG-BMI, TyG-WC	Incident stroke
Peng Zhang[15]	2025	China	NHANES	Cross-sectional	NA	50.51%	61.64	Mexican American, Black, White, others	TyG, TyG-WHtR, TyG-WC, TyG-BMI	All-cause mortality, CVD mortality
Xiaoyuan Wei[16]	2024	USA	NHANES	Prospective	NA	48.11%	59	Mexican American, Black, White, others	TyG, TyG-WC, TyG-WHtR	All-cause mortality, CVD mortality, Other
Yong Yue[17]	2025	China	CHARLS	Prospective	5.20	47.30%	58	Chinese	TyG	Incident stroke
Chang Liu[18]	2025	China	NHANES	Prospective	7.92	50.70%	49.61±0.32	Mexican American, Black, White, others	TyG	All-cause mortality, CVD mortality
Mingjie Chen[19]	2025	China	NHANES; CHALRS	Cross-sectional ; Prospective	9.00	48.49%	60	American; Chinese	TyG	All-cause mortality, CVD mortality
Jiahao Zou[20]	2025	USA	NHANES	Retrospective	13.70	50.43%	64	Mexican	TyG	All-cause

								American, Black, White, others		mortality, CVD mortality, Other
Byung Sik Kim[21]	2025	Korea	Korean National Health Insurance Database (NHID)	Retrospective	12.60	53.23%	48.9	Korean	TyG	Incident MACE
Qi-chao Sun[22]	2023	China	participants at Nanjing Drum Tower Hospital	Cross-sectional	NA	NA	48.5	Chinese	TyG	Incident CVD
Peixin Lan[23]	2026	China	NHANES	Cross-sectional	NA	49.00%	51	Mexican American, Black, White, others	TyG, TyG-BMI, TyG-WC, TyG-WHtR	Incident CVD
Qiyu Xiao[24]	2025	China	Systolic Blood Pressure Intervention Trial (SPRINT)	Prospective	NA	64.53%	67.87±9.41	White, others	TyG	Incident MACE
Man Li[25]	2025	China	Kailuan Study	Prospective	14.19	NA	NA	Chinese	TyG	Incident CVD, Incident CHD, Incident stroke
Menghe Wang[26]	2025	China	Guangzhou Older Longitudinal Dynamic Health (GOLD-Health) cohort	Prospective	2.96	39.60%	70.82±6.03	Chinese	TyG, TyG-BMI, TyG-WC, TyG-WHtR	Incident CVD
Yu Tang[27]	2026	China	CHARLS	Prospective	9.00	47.09%	58	Chinese	TyG	Incident CVD
Hui Li[28]	2026	China	UK Biobank	Prospective	15.31	44.90%	55.6±8.09	White, others	TyG, CTI	Incident CHD
Hao Li[29]	2026	China	CHARLS	Prospective	5.00	NA	NA	Chinese	CTI, TyG	Incident CVD
Ke Wang[30]	2026	China	CHARLS	Prospective	9.00	47.04%	58.99±9.21	Chinese	TyG-WHtR	Incident CVD
Xiaoran Shen[31]	2025	USA	NHANES	Prospective	7.60	46.71%	50	Mexican	TyG-WHt	All-cause

								American, Black, White, others	R, TyG	mortality, CVD mortality
Yilin Pan[32]	2025	China	CHARLS	Prospective	6.00	46.32%	55	Chinese	TyG, TyG-BMI, TyG-WC, TyG-WHtR, CTI	Incident CVD
Wei-Yu Su[33]	2019	China	Kaohsiung Medical University Hospital Research Database (KMUHRD)	Retrospective	5.93	49.10%	61.68±11.90	Chinese	TyG	Incident MACE
Jianan Hong[34]	2025	China	CHARLS	Prospective	9.00	46.90%	60.53±9.33	Chinese	TyG, TyG-BMI, TyG-WC, TyG-WHtR	Incident CVD
Qingsong Mao[35]	2025	China	CHARLS	Prospective	9.00	46.90%	60.53±9.33	Chinese	TyG, TyG-BMI, TyG-WC, TyG-WHtR	Incident CVD
Yinsong Xu[36]	2025	China	CHARLS	Prospective	9.00	45.20%	57.6±8.8	Chinese	CTI	Incident stroke
Huiwen Ou[37]	2025	China	CHARLS	Prospective	9.00	45.34%	57	Chinese	CTI	Incident CVD, All-cause mortality
Qianliang Ying[38]	2025	China	NHANES	Prospective	14.00	48.73%	48.83	Mexican American, Black, White, others	CTI	All-cause mortality, CVD mortality
Songyuan	2024	China	CHARLS	Prospective	7.00	45.00%	62±10	Chinese	CTI	Incident stroke

Tang[39]										
Yulong Lan[40]	2025	China	NHANES	Retrospective	13.70	50.43%	64	Mexican American, Black, White, others	TyG	All-cause mortality, CVD mortality, Other
Shi Tai[41]	2022	USA	ACCORD/ACCORD ION trial	Retrospective	8.80	61.48%	62.77±6.63	White, others	TyG	Incident MACE
Tao Yi[42]	2025	US	NHANES	Retrospective	10.75	51.00%	44	Mexican American, Black, White, others	TyG	All-cause mortality, CVD mortality
Xin Chen[43]	2025	China	Hypertensive patients cohort from Jiangsu Province, China (baseline survey 2010, follow-up to 2020)	Prospective	8.32	41.77%	58.49±7.34	Chinese	TyG	Incident CVD
Anthony Diwon[44]	2026	China	Second Affiliated Hospital of Wenzhou Medical University and the Second Affiliated Hospital of Zhejiang University School of Medicine	Retrospective	3.80	53.40%	50	Chinese	TyG	All-cause mortality, CVD mortality
Jinling Hu[45]	2025	UK	UK Biobank	Prospective	13.45	39.30%	58	White, others	TyG, TyG-BMI, TyG-WC, TyG-WHtR	Incident CVD, Incident CHD, Incident stroke
Chuanwei Zhao[46]	2026	USA	NHANES	Cross-sectional	NA	50.59%	51.20±0.24	Mexican American,	TyG	CVD mortality

								Black, White, others		
Xiaoling Chen[47]	2025	USA	Systolic Blood Pressure Intervention Trial (SPRINT)	Prospective	3.33	64.50%	67.9	Black, others	TyG	Incident MACE
Feng-Ching Shen[48]	2023	China	Integrated CKD Care Program in Kaohsiung for Delaying Dialysis	Prospective	12.17	64.20%	62.6±14.4	Chinese	TyG, TyG-BMI	All-cause mortality, Other
Claudia R. L. Cardoso[49]	2026	Brazil	Rio de Janeiro Type 2 Diabetes Cohort (tertiary-care Clementino Fraga Filho University Hospital, 2004–2008)	Prospective	10.60	38.70%	60.2±9.5	Mixed Brazilian	TyG; TyG-BMI	Incident MACE, All-cause mortality, CVD mortality, Other
Tonglong Jin[50]	2026	China	CHARLS	Prospective	9.00	47.60%	59.2±9.0	Chinese	TyG, TyG-BMI, TyG-WC, TyG-WHtR, CTI	Incident stroke, Incident CHD, Other
Weida Qiu[51]	2024	China	CHARLS	Prospective	8.90	46.00%	58.3±8.7	Chinese	TyG	Incident CVD
Yanling Yang[52]	2025	USA	NHANES	Prospective	6.11	53.90%	72	Mexican American, Black, White, others	TyG, TyG-BMI	All-cause mortality
Jiajun Liu[53]	2025	USA	NHANES	Prospective	9.20	48.62%	58	Mexican American, Black, White, others	TyG	All-cause mortality, CVD mortality
Younan Yao[54]	2024	US	NHANES	Prospective	8.92	51.84%	62	Mexican	TyG	All-cause

								American, Black, White, others		mortality, CVD mortality, Other
Kexin Wang[55]	2025	China	NHANES	Cross-sectional	NA	52.30%	49	Mexican American, Black, White, others	TyG	All-cause mortality, CVD mortality
Lei Ding[56]	2024	USA	NHANES	Retrospective	6.44	51.40%	70.0±6.8	Mexican American, Black, White, others	TyG-BMI	All-cause mortality, CVD mortality
Cheng Huang[57]	2025	China	CHARLS	Prospective	7.77	46.48%	58.47±9.50	Chinese	TyG-WHtR	Incident CVD
Jianyong Zhao[58]	2025	China	Chaohu Hospital (Anhui Medical University)	Retrospective	3.17	61.46%	63	Chinese	TyG	Incident MACE
Xi Chen[59]	2025	China	The Second Affiliated Hospital of Harbin Medical University	Retrospective	6.08	57.70%	53.4±10.2	Chinese	TyG	Incident MACE
Xingyu Zhou[60]	2025	China	CHARLS	Prospective	4.00	46.30%	57.8±9.1	Chinese	TyG	Other
Qin Zhang[61]	2023	USA	NHANES	Prospective	NA	59.33%	66.54±0.45	Mexican American, Black, White, others	TyG	All-cause mortality, CVD mortality
Shu Yang[62]	2025	China	NHANES	Cross-sectional	NA	52.20%	70.17±6.78	Mexican American, Black, White, others	TyG	Incident CVD

Zhengwen Xu[63]	2025	China	Patients with H-type hypertension admitted to the Department of Cardiology at Wuhan Third Hospital	Retrospective	2.00	52.30%	65.4±10.7	Chinese	TyG	Incident Heart failure
Mengjie Zhao[64]	2023	USA	NHANES	Prospective	6.83	53.17%	64	Mexican American, Black, White, others	TyG	All-cause mortality, CVD mortality
Fomin Zhang[65]	2023	China	China Patient-Centered Evaluative Assessment of Cardiac Events (China PEACE) study	Prospective	3.50	44.20%	63	Chinese	TyG	Incident MACE
Matilde Sbriscia[66]	2025	Italy	IRCCS INRCA cohort	Retrospective	16.80	54.00%	67	White, others	TyG	All-cause mortality, Incident MACE, Other
Lian Wang[67]	2025	China	Inpatients from Wuhan Third Hospital & Tongren Hospital of Wuhan University	Retrospective	NA	53.40%	67.69±10.15	Chinese	TyG-BMI	Incident CHD
Mao-Jun Liu[68]	2025	USA	ACCORD/ACCORD ION trial	Retrospective	8.82	61.47%	62.81±6.64	White, others	TyG	All-cause mortality, CVD mortality
Peng Ning[69]	2024	China	Inpatients from Chengdu Fifth People's Hospital	Retrospective	NA	56.63%	59.2±12.92	Chinese	TyG	Other
Yun Kyung Cho[70]	2022	Korea	National Health Insurance Service-National	Retrospective	5.00	43.80%	59.7±8.8	Korean	TyG	Incident MACE, CVD mortality

			Health Screening Cohort (NHIS-HEALS)							
Mao-Jun Liu[71]	2025	USA	ACCORD/ACCORD ION trial	Retrospective	8.82	61.48%	62.80±6.64	White, others	TyG, TyG-WHtR, TyG-WC	Incident MACE, All-cause mortality
Kun Liu[72]	2025	China	UK Biobank	Prospective	13.63	46.80%	57	White, others	TyG index, TyG-BMI, TyG-WC, TyG-WHtR	Incident CVD, Incident CHD, Incident stroke, All-cause mortality, CVD mortality
Yumeng Shi[73]	2024	USA	NHANES	Cross-sectional	NA	53.29%	55.83±16.55	Mexican American, Black, White, others	TyG	Incident Heart failure
Hui-juan Li[74]	2025	China	Kaifeng Central Hospital	Prospective	2.35	53.90%	64.1±9.8	Chinese	TyG	Incident MACE
Shuping Yang[75]	2023	USA	NHANES	Cross-sectional	NA	46.20%	54.69±16.10	Mexican American, Black, White, others	TyG-BMI	Incident Heart failure
Jeongeun Kwak[76]	2025	Korea	Korean National Health Insurance Service (NHIS) database	Retrospective	5.64	68.55%	56.96±12.47	Korean	TyG	Incident CVD, Incident MACE, All-cause mortality
Haipeng Yao[77]	2022	China	Affiliated Hospital of Jiangsu University	Retrospective	3.42	56.00%	67.13±11.07	Chinese	TyG	Other, All-cause mortality
Chen Li[78]	2024	China	Fuwai Hospital, Chinese Academy of	Prospective	2.60	71.20%	31.9±5.8	Chinese	TyG	Incident MACE

			Medical Science							
Nan Zhang[79]	2023	China	China Stroke Primary Prevention Trial (CSPPT)	Prospective	4.50	39.50%	59.5	Chinese	TyG	Incident stroke
Jiajun Liu[80]	2025	China	NHANES	Prospective	8.30	41.62%	NA	Mexican American, Black, White, others	TyG, TyG-WHtR, and METS-IR	All-cause mortality, CVD mortality

(B).Adjustments and Baseline Cancer/Degenerative Disease

First Author	Adjustments	Baseline Cancer/Degenerative Disease
Dan Zhou[1]	Age, gender, race, smoking, marital status, education level, body mass index, systolic blood pressure, estimated glomerular filtration rate, total cholesterol, high density lipoprotein cholesterol, comorbidities (cardiovascular disease and diabetes), and medicine use (antihypertensive drugs, hypoglycemic agents, lipid-lowering drugs, and antiplatelet drugs)	Not explicitly ruled out
Zhihui Lu[2]	Gender, Age, Smoking statues, Drinking statues, Education level, BMI, Waist circumference, Hypertension, Diabetes, Sleep problems, PLT, BUN, Scr, TC, HDL-c, LDL-c, CRP, UA, HbA1c	Clearly included
Wei Xian[3]	gender, age, marital status, education level, smoking status, drinking status, BMI, eGFR, hypertension, diabetes, dyslipidemia, heart disease, hypertension medications, diabetes medications, dyslipidemia medications	Clearly excluded
Jintao Chen[4]	Age, gender, obesity, education level, marital status, residence, smoking, drinking, antihypertensive drugs, antidiabetic drugs, TC, LDL-C, lung diseases, liver diseases, cancer, digestive disease	Clearly included
YaJie Zhao[5]	Age, sex, income, education, marital status, smoking, drinking, sleep disorders, physical activity, CKM stage	Not explicitly ruled out
Ze-Jiaxin Niu[6]	Age, sex, marital status, physical activity, educational level, residence, diabetes, dyslipidemia, smoking, alcohol consumption, BMI	Not explicitly ruled out
Yafang Chen[7]	Age, sex, BMI, SBP, DBP, smoking, drinking, HDL-C, LDL-C, hypertension, diabetes, hyperlipidemia, TC, ALT, AST	Clearly excluded
Weipeng Li[8]	Age, gender, smoking status, drinking status, sleep problems, education level, marital status, BUN, Scr, TC, HDL-C, LDL-C, CRP, UA, PLT, hypertension, diabetes, depression	Clearly included
Yuyu Cui[9]	age, sex, education level, marital status, SBP, DBP, BMI, social activity, residence, HbA1c, HDL-C, LDL-C, BUN, UA, drinking history, smoking history, diabetes, dyslipidemia,kidney disease, and history of medication use	Clearly included
Chang Liu[10]	Age, gender, race, educational levels, PIR, BMI, hypertension, HDL-C, LDL-C, alcohol use, and smoking status	Not explicitly ruled out
Xiandu Jin[11]	Gender, age, educational levels, race, SBP, smoking, drinking, TC, HDL-C, diabetes, and dyslipidemia	Not explicitly ruled out
Yanjuan Chen[12]	Age, sex, smoking status, alcohol consumption habits, physical exercise habits, education, family history of CMD, BMI, SBP, FPG, HDL-C, LDL-C, UA, eGFR, and hs-CRP	Not explicitly ruled out
Xinyi Shao[13]	Age, sex, educational level, race and ethnicity, Townsend deprivation index (TDI), alcohol consumption status, smoking status	Not explicitly ruled out
Keyu Bian[14]	Age, gender, ethnicity, Townsend Deprivation Index (TDI), years of education, smoking status, drinking frequency, systolic and diastolic blood pressure, history of hypertension, use of antihypertensive and lipid-lowering drugs, physical activity, healthy diet, sleep patterns; additional adjustment for diabetes duration in the diabetes subgroup	Not explicitly ruled out
Peng Zhang[15]	Age, gender, race, poverty income ratio, eGFR, UACR, uric acid, SBP, DBP, HDL-C, LDL-C, HbA1c, smoking, drinking, statin use, antihypertensive use, antihyperglycemic agent use, insulin use, liver disease, cancer, hypertension, diabetes, MeTS, CKD, and CKM syndrome	Clearly included

Xiaoyuan Wei[16]	Age, gender, race, marital status, educational level, family income-poverty ratio, smoking status, alcohol use, history of cancer, chronic kidney disease (CKD), cardiovascular disease (CVD), BMI (only for TyG index), energy intake, serum levels of total cholesterol (TC), high-density lipoprotein cholesterol (HDL-C), low-density lipoprotein cholesterol (LDL-C), blood urea nitrogen (BUN), uric acid (UA), estimated glomerular filtration rate (eGFR), glutamic-pyruvic transaminase (ALT), aspartate transaminase (AST), albumin (ALB), total bilirubin (TBil)	Clearly included
Yong Yue[17]	Age, gender, education level, alcohol intake, smoking, hypertension, DM, antidiabetic agents, antihypertensive agents, SBP, DBP, PLT, BUN, Cr, LDL-C, CRP, HbA1c, UA, Hb, eGFR	Not explicitly ruled out
Chang Liu[18]	age, gender, race, education, PIR, BMI, hypertension, HDL-C, LDL-C, ALT, AST, HbA1c, alcohol use, and smoking status	Not explicitly ruled out
Mingjie Chen[19]	Age, sex, ethnicity, educational level, marital status, PIR, smoking status, alcohol consumption, HEI-2015, PA, HDL, LDL, HbA1c, eGFR, uACR, uric acid, CKD, liver disease, cancer, hypertension, DM, antihypertensive drug, hypoglycemic therapy, lipid-lowering drug, CKM syndrome	Clearly included
Jiahao Zou[20]	Age, sex, race, PIR, BMI, HDL-C, TC, AST, GGT, UA, alcohol user, smoker, and antihyperlipidemic drug	Clearly excluded
Byung Sik Kim[21]	Age, sex, smoking status, alcohol consumption, physical activity, household income, use of antihypertensive drugs, glucose-lowering drugs, lipid-lowering drugs, antiplatelet drugs, eGFR category, dipstick proteinuria	Not explicitly ruled out
Qi-chao Sun[22]	age, sex, body mass index, systolic blood pressure and low-density lipoprotein cholesterol	Not explicitly ruled out
Peixin Lan[23]	sex,race, age, and education level, PIR protein, total sugar, dietary fiber, total fat, cholesterol, his tory of cancer, vigorous activity, moderate activity, history of hypertension, cotinine exposure history, history of stroke, history of thyroid disease, smoking, and drinking status.	Clearly included
Qiyu Xiao[24]	The fully adjusted model included: gender, age, race, smoking status, alcohol consumption, vigorous physical activity, BMI, CKD, aspirin use, statin use, hyperlipidaemia, CVD, Framingham risk score	Not explicitly ruled out
Man Li[25]	The fully adjusted model included: age, sex, current smoking, current drinking, physical activity, education, snoring, CVD family history, BMI, TC, LDL-C, HDL-C, log-transformed hsCRP, hypertension, antihypertensive drugs, lipid-lowering drugs	Clearly excluded
Menghe Wang[26]	Age, sex, education, marriage status, occupation, smoking, drinking, physical activity, BMI, LDL-C, HDL-C, CREA (creatinine), WBC, antihypertensive medication, antidiabetic medication	Clearly included
Yu Tang[27]	Sex, age, smoking status, alcohol consumption, education level, marital status, hypertension, diabetes, cancer, lung disease, liver disease, eGFR, platelets, BUN, CRP, SUA	Clearly included
Hui Li[28]	Age, sex, BMI, race, physical activity, hypertension, cumulative dietary risk score, smoking status, alcohol consumption, lipid-lowering therapy, HbA1c, and fasting hours	Clearly excluded
Hao Li[29]	The fully adjusted model included: age, sex, education level, marital status, household registration, current smoking, current drinking, SBP, DBP, BMI, FBG, TG, TC, LDL, CRP, HbA1c, hypertension status, diabetes status	Not explicitly ruled out
Ke Wang[30]	Age, gender, education level, CESD-10 score, sleep time, smoking, alcohol consumption, hypertension, diabetes, dyslipidemia, chronic kidney diseases	Not explicitly ruled out

Xiaoran Shen[31]	The fully adjusted model included: age, sex, race, education level, family income-to-poverty ratio, smoking status, drinking status, hypertension, BMI, eGFR, HbA1c, TC, HDL-C, uric acid, and BUN	Not explicitly ruled out
Yilin Pan[32]	Age, sex, marital status, education level, residence, smoking, drinking, hypertension, diabetes, dyslipidemia, creatinine, and CRP	Not explicitly ruled out
Wei-Yu Su[33]	The fully adjusted model included: hemoglobin A1c, age, sex, hypertension, neuropathy, nephropathy, coronary artery disease, peripheral artery disease, episodes of hypoglycemia, fasting glucose, triglycerides, LDL cholesterol, eGFR	Not explicitly ruled out
Jianan Hong[34]	Age, gender, marital status, education level, smoking status, drinking status, history of hypertension and diabetes, BUN, UA, eGFR, and LDL	Not explicitly ruled out
Qingsong Mao[35]	Age, gender, marital status, education, smoking and drinking habits, prior history of hypertension and diabetes, BUN, uric acid (UA), eGFR, and LDL	Not explicitly ruled out
Yinsong Xu[36]	Age, sex, education level, alcohol consumption, smoking status, hemoglobin level, diabetes, dyslipidemia, LDL-C, fasting glucose, creatinine, place of residence, hypertension, antihyperlipidemic, antihypertensive, and antidiabetic medications	Not explicitly ruled out
Huiwen Ou[37]	Age, sex, smoking status, drinking status, marital status, educational level, BMI, eGFR, hypertension, dyslipidemia, diabetes, hypertension treatment, diabetes treatment, dyslipidemia treatment	Not explicitly ruled out
Qianliang Yi ng[38]	age, sex, and race, marital status, education level, family poverty-income ratio, smoking status, alcohol use, BMI, HEI score, HbA1c, TC, HDL, LDL, serum uric acid, eGFR, drugs for hypertension, hyperlipidemia, diabetes	Not explicitly ruled out
Songyuan Tang[39]	Gender, heart disease, hypertension treatment, SBP, LDL, uric acid (UA)	Not explicitly ruled out
Yulong Lan[40]	age, sex, race, poverty-to-income ratio (PIR), BMI, HDL–C, TC, AST, GGT, UA, alcohol use, smoking status, lipid-lowering medications use	Not explicitly ruled out
Shi Tai[41]	Baseline age, sex, previous CV event, race, BMI, education, systolic blood pressure, diastolic blood pressure, baseline eGFR, HbA1c, total plasma cholesterol, plasma LDL-C, live alone, duration of diabetes, depression, statins, biguanide, aspirin, ACEI/ARB, and insulin	Not explicitly ruled out
Tao Yi[42]	Age, gender, ethnicity, educational level, smoking status, alcohol use, poverty-to-income ratio, estimated glomerular filtration rate, total cholesterol, LDL-C, HDL-C, hypertension status, high cholesterol status, antihypercholesterolemia medications, antihypertensive medications, history of coronary heart disease, myocardial infarction, heart failure, stroke	Not explicitly ruled out
Xin Chen[43]	The fully adjusted model included: age, sex, mean arterial pressure (MAP), total cholesterol (TC), high-density lipoprotein cholesterol (HDL-C), low-density lipoprotein cholesterol (LDL-C), hypertension treatment, family history of CVD, BMI, smoking status, drinking status, physical activity, intake of vegetables and fruits	Not explicitly ruled out
Anthony Diwon[44]		Clearly excluded
Jinling Hu[45]	Age, sex, ethnicity, Townsend Deprivation Index (TDI), employment status, educational attainment, household income, smoking status, physical activity, alcohol consumption, sleep duration, diet quality score, SBP, DBP, HbA1c, history of hypertension and diabetes, antihypertensive, hypoglycemic, lipid-lowering medications, polygenic risk scores (PRS)	Not explicitly ruled out

Chuanwei Zhao[46]	Age, gender, race, education, marital status, PIR, smoking status, alcohol use, physical activity, cancer, ALT, AST, albumin	Clearly included
Xiaoling Chen[47]		Not explicitly ruled out
Feng-Ching Shen[48]	Age, sex, eGFR, Upcr (log value), cardiovascular disease, smoking, cancer, severe liver disease, hypertension, hemoglobin level, BMI, cholesterol level (log value), glycosylated hemoglobin level, albumin level, CRP (ln value), and phosphorus level	Clearly included
Claudia R. L. Cardoso[49]	Model 3 (most fully adjusted): Age, sex, BMI (except TyG-BMI analyses; body height used instead in neuropathy analyses), smoking, physical activity, diabetes duration, pre-existing macro- and microvascular complications, baseline SBP, HbA1c, number of anti-hypertensive drugs, use of insulin/statins/fibrates/aspirin, baseline eGFR and albuminuria (renal outcomes only), plus serum LDL-cholesterol (or total cholesterol)	Not explicitly ruled out
Tonglong Jin[50]	Age, gender, marital status, education level, Hukou, smoking status, drinking status, hypertension, diabetes, dyslipidemia, kidney disease, SBP, DBP, HDL-C, LDL-C, eGFR, UA, Cr, BUN, Hb, MCV, PCV, platelet count, WBC, stages of CKM syndrome	Not explicitly ruled out
Weida Qiu[51]	Age, sex, marital status, education, residence, SBP, pulse, BMI, waist circumference, hemoglobin, LDL-C, HDL-C, uric acid, eGFR, CRP, HbA1c, smoking, drinking, hypertension, antihypertensive treatment, diabetes, anti-diabetic treatment, dyslipidemia, lipid-lowering treatment, chronic lung disease	Not explicitly ruled out
Yanling Yang[52]	Age, ethnicity, educational levels, BMI, albumin, UACR, UA, creatinine, smoking status, insulin treatment, hypoglycemic drugs, antihypertensive drugs, lipid-lowering drugs, hypertension, hyperlipidemia, stroke, CAD, FBG, fasting insulin, HbA1c, and eGFR	Not explicitly ruled out
Jiajun Liu[53]		Clearly included
Younan Yao[54]	Age, gender, ethnicity, educational level, current smoking, BMI, hypertension, CAD, CHF, albumin, LDL-C, HbA1c, eGFR, UACR, UA, insulin drug, metformin, and statin	Clearly excluded
Kexin Wang[55]	Age, gender, race, body mass index, smoking status, alcohol consumption, educational level, family poverty-income ratio, antidiabetic agents, antihypertensive agents, antihyperlipidemic agents, eGFR, HDL-C, and TG	Not explicitly ruled out
Lei Ding[56]	Age, gender, race, hypertension, total cholesterol, HDL-C, LDL-C, CHD, heart failure, cancer, stroke, and COPD	Clearly included
Cheng Huang[57]	Age, sex, marital status, rural, hypertension, dyslipidemia, diabetes, drinking status, smoking status, CKM, medication history, SBP, DBP, HDL-C, LDL-C, TC, UA, BUN, HbA1c and CRP	Not explicitly ruled out
Jianyong Zhao[58]	Age, sex, Hb, Alb, C-P (C-peptide), RBC count, HbA1c%, 25(OH)D, urate-lowering drugs, and purine diet stratification	Not explicitly ruled out
Xi Chen[59]	Age, sex, BMI, current smoking, diabetes, established CVDs, CKDs, antiplatelet drugs, antidiabetic drugs, and antilipidemic drugs	Not explicitly ruled out
Xingyu Zhou[60]	Age, gender, marriage status, annuals household income, occupation status, education levels, urbanity, smoking, alcohol drinking, low-density lipoprotein cholesterol (LDL-C), eGFR, BMI, waist, HbA1c, HDL-C, SBP, and DBP	Not explicitly ruled out
Qin	Age, gender, race, BMI, tobacco use, alcohol use, education, hypertension,	Not explicitly

Zhang[61]	COPD, family income-poverty ratio	ruled out
Shu Yang[62]	age, sex, race, education level, marital status,smoking status, DBP, TC, HDL-C, LDL-C, albumin, eGFR, uric acid, Hypertension, CKD, antihypertensive drugs and Hypertension (Yes/No)	Not explicitly ruled out
Zhengwen Xu[63]	Age, sex, body mass index (BMI), smoking status, alcohol consumption, diabetes, baseline blood pressure, coronary heart disease (CHD), dyslipidemia, and renal dysfunction	Not explicitly ruled out
Mengjie Zhao[64]	Age, sex, education level, race/ethnicity, PIR, smoking status, drinking status, BMI, CHD, HF, heart attack, stroke, angina, hypertension, hyperlipidemia, glucose-lowering medication use, HbA1c, LDL-C, and TC	Not explicitly ruled out
Fomin Zhang[65]	Age, smoke, urban residence, previous MI, previous PCI, lipid-lowering drugs, anti-hypertensive drugs, anti-platelet drugs, and anti-diabetic drugs	Not explicitly ruled out
Matilde Sbriscia[66]	Sex, smoking, hypertension, age, BMI, HbA1c, statin therapy, presence of T2D complications, eGFR, total cholesterol, and hs-CRP	Not explicitly ruled out
Lian Wang[67]	Age, sex, SBP, DBP, BMI, smoking, drinking, TC, TG, LDL-C, HDL-C, CCB, β-receptor blocker, ACEI/ARB	Not explicitly ruled out
Mao-Jun Liu[68]	Age, sex, BMI, hypertension, coronary heart disease, stroke, fatty liver disease, smoking, drinking, HbA1c, HDL-C, LDL-C, and eGFR	Not explicitly ruled out
Peng Ning[69]	Sex, age, diabetes duration, smoking, drinking, TC, ALB, left SBP, right SBP	Not explicitly ruled out
Yun Kyung Cho[70]	Age, sex, smoking habits, alcohol drinking, physical activity, waist circumference, estimated glomerular filtration rate (eGFR), gamma-glutamyl transpeptidase level, low-density lipoprotein cholesterol level, and the use of anti-diabetic, anti-hypertensive, lipid-lowering, and antiplatelet agents	Not explicitly ruled out
Mao-Jun Liu[71]	Sex, race, age, education, history of cardiovascular disease, previous hypertension, previous hyperlipidemia, systolic blood pressure, diastolic blood pressure, duration of diabetes, proteinuria,	Not explicitly ruled out
Kun Liu[72]	Age, sex, ethnicity, educational attainment, Townsend deprivation index (TDI), household income, employment status, smoking status, alcohol consumption frequency, physical activity, sleep duration, diet quality, SBP, DBP, TC, and LDL-C	Not explicitly ruled out
Yumeng Shi[73]	The fully adjusted model included: age, sex, race, poverty income ratio, BMI, current smoking, HbA1c, eGFR, serum albumin, hypertension, hyperlipidemia, antihypertensive drugs, lipoprotein-lowering drugs	Not explicitly ruled out
Hui-juan Li[74]	Age, sex, BMI, history of hypertension, DM, cerebrovascular disease, chronic kidney disease, atrial fibrillation, valvular heart disease, FBG, HbA1c, HCY, UA, LDL-C, eGFR, Scr, ALT, TG, NT-proBNP, left ventricular end-diastolic diameter (LVEDD), left atrial diameter (LAD), and left ventricular ejection fraction (LVEF)	Not explicitly ruled out
Shuping Yang[75]	age, gender, race, smoking status, drinking consumption, LDL-C, eGFR, hypertension, coronary artery disease, ACE inhibitors, Beta blocker, statin, diuretics	Not explicitly ruled out
Jeongeun Kwak[76]	Age, sex, body mass index (BMI), smoking status, alcohol consumption, regular exercise, income level, hypertension, chronic kidney disease (CKD), use of three or more oral anti-diabetic agents, insulin therapy, and total cholesterol	Clearly included
Haipeng	Age, duration of diabetes, aortic calcification, hypertension, history of	Not explicitly

Yao[77]	ACEI/ARB medication, history of insulin medication, albumin, BUN, and SCr	ruled out
Chen Li[78]	Age, sex, body mass index (BMI), current smoking status, CAD, chronic renal disease, previous stroke, diabetes, hyperlipidemia, duration or the grades of hypertension, total cholesterol, low-density lipoprotein (LDL), high-density lipoprotein (HDL), and glycated hemoglobin	Not explicitly ruled out
Nan Zhang[79]	Age, sex, body mass index, systolic blood pressure, diastolic blood pressure, total homocysteine, vitamin B12, estimated glomerular filtration rate, MTHFR C677T, smoking status, alcohol drinking and treatment blood pressure	Not explicitly ruled out
Jiajun Liu[80]	age, gender, ethnicity, educational level, BMI, hypertension, hyperlipidemia, diabetes, smoking status and drinking status	Not explicitly ruled out

Reference:

[1]Zhou, D., Liu, X. C., Kenneth, L., Huang, Y. Q., & Feng, Y. Q. (2021). A Non-Linear Association of Triglyceride Glycemic Index With Cardiovascular and All-Cause Mortality Among Patients With Hypertension. *Front Cardiovasc Med, 8*, 778038. doi:10.3389/fcvm.2021.778038

[2]Lu, Z., Li, L., Wang, X., Lv, L., Rong, S., & Li, B. (2025). Association between C-reactive protein-triglyceride glucose index and Future cardiovascular disease risk in a population with cardiovascular-Kidney-metabolic syndrome stage 0-3. *Sci Rep, 15*(1), 31152. doi:10.1038/s41598-025-17173-0

[3]Xian, W., Wu, T., Zhao, X. X., Yang, Y., Li, X. Y., & Wang, Y. L. (2026). Association between C-reactive protein-triglyceride-glucose index and risk of stroke in different renal function status: a national cohort study. *Cardiovasc Diabetol, 25*(1). doi:10.1186/s12933-026-03135-x

[4]Chen, J., Yan, L., He, L., & Wang, W. (2025). Association between different dimensions of the C-reactive protein-triglyceride-glucose index and future cardiovascular disease risk in individuals with cardiovascular-kidney-metabolic syndrome stages 0-3: a nationwide cohort study. *Diabetol Metab Syndr, 17*(1), 299. doi:10.1186/s13098-025-01882-7

[5]Zhao, Y., Wang, L., Li, J., Guo, B., Zhang, J., Guo, X., . . . Zhao, X. (2025). [Association Between Surrogate Markers of Insulin Resistance and Incident Cardiovascular Disease in a Population With Stages 0-3 Cardiovascular-Kidney-Metabolic Syndrome: A Prospective Cohort Study]. *Sichuan Da Xue Xue Bao Yi Xue Ban, 56*(2), 495-505. doi:10.12182/20250360503

[6]Niu, Z. J., Liu, Z. A., Wei, T., Dou, M., Tian, P. X., & Cui, Y. (2026). Association between the combined c-reactive protein-triglyceride glucose index and hypertension with long-term stroke among cardiovascular-kidney-metabolic syndrome stages 0-3 population: a nationwide prospective cohort study. *Cardiovasc Diabetol, 25*(1), 55. doi:10.1186/s12933-026-03075-6

[7]Chen, Y., Jia, W., Guo, J., Yang, H., Sheng, X., Wei, L., & Li, J. (2025). Association between the C-reactive protein-triglyceride glucose index and new-onset coronary heart disease among metabolically heterogeneous individuals. *Cardiovasc Diabetol, 24*(1), 316. doi:10.1186/s12933-025-02876-5

[8]Li, W., Shen, C., Kong, W., Zhou, X., Fan, H., Zhang, Y., . . . Zheng, L. (2024). Association between the triglyceride glucose-body mass index and future cardiovascular disease risk in a population with Cardiovascular-Kidney-Metabolic syndrome stage 0-3: a nationwide prospective cohort study. *Cardiovasc Diabetol, 23*(1), 292. doi:10.1186/s12933-024-02352-6

[9]Cui, Y., Xu, Z., Ding, L., Li, Y., Zhou, X., & Li, L. (2025). Association between the triglyceride glucose-waist circumference index and cardiovascular disease across different glycemic statuses among middle-aged and older Chinese adults. *Front Cardiovasc Med, 12*, 1608655. doi:10.3389/fcvm.2025.1608655

[10]Liu, C., Liang, D., Xiao, K., & Xie, L. (2024). Association between the triglyceride-glucose index and all-cause and CVD mortality in the young population with diabetes. *Cardiovasc Diabetol, 23*(1), 171. doi:10.1186/s12933-024-02269-0

[11]Jin, X., Feng, Y., Liu, C., Cheng, T., Jin, Y., Shao, Z., . . . Qi, X. (2026). Association between triglyceride-glucose index and related indicators with heart failure-related mortality in individuals with early-stage cardiovascular-kidney-metabolic syndrome. *Cardiovasc Diabetol, 25*(1). doi:10.1186/s12933-026-03149-5

[12]Chen, Y., Wu, W., Cai, Z., Wu, K., Zheng, H., Fu, P., . . . Chen, Y. (2025). Association between triglyceride-glucose index and the risk of cardiometabolic diseases in metabolically healthy obese individuals: a prospective cohort study. *Front Endocrinol (Lausanne), 16*, 1524786. doi:10.3389/fendo.2025.1524786

[13]Shao, X., Tan, Z., Sun, L., Fu, Y., Chen, A., Chen, J., . . . Zhang, J. (2025). Association between triglyceride-glucose index combined with a body shape index and atherosclerotic cardiovascular disease risk varies by glycemic status: insights from the UK Biobank. *Cardiovasc Diabetol, 24*(1), 456. doi:10.1186/s12933-025-03021-y

[14]Bian, K., Hou, C., Jin, H., Feng, X., Peng, M., Zhao, X., . . . Xu, G. (2025). Association between Triglyceride-Glucose indices and ischemic stroke risk across different glucose metabolism statuses. *Diabetes Res Clin Pract, 222*, 112064. doi:10.1016/j.diabres.2025.112064

[15]Zhang, P., Mo, D., Zeng, W., & Dai, H. (2025). Association between triglyceride-glucose related indices and all-cause and cardiovascular mortality among the population with cardiovascular-kidney-metabolic syndrome stage 0-3: a cohort study. *Cardiovasc Diabetol, 24*(1), 92. doi:10.1186/s12933-025-02642-7

[16]Wei, X., Min, Y., Song, G., Ye, X., & Liu, L. (2024). Association between triglyceride-glucose related indices with the all-cause and cause-specific mortality among the population with metabolic syndrome. *Cardiovasc Diabetol, 23*(1), 134. doi:10.1186/s12933-024-02215-0

[17]Yue, Y., Li, P., Sun, Z., Murayama, R., Li, Z., Hashimoto, K., & Zhang, Y. (2025). Association of novel triglyceride-glucose-related indices with incident stroke in early-stage cardiovascular-kidney-metabolic syndrome. *Cardiovasc Diabetol, 24*(1), 301. doi:10.1186/s12933-025-02854-x

[18]Liu, C., Liang, D., Xiang, G., Zhao, X., Xiao, K., & Xie, L. (2025). Association of the triglyceride glucose index with all cause and CVD mortality in the adults with diabetes aged < 65 years without cardiovascular disease. *Sci Rep, 15*(1), 2745. doi:10.1038/s41598-025-86612-9

[19]Chen, M., Guo, J., Shangguan, Y., Sun, Z., He, X., Tu, Q., & Yan, Q. (2025). Association of the triglyceride-glucose index combined with a body shape index with all-cause and cardiovascular mortality in individuals with cardiovascular-kidney-metabolic syndrome stage 0-3: findings from two prospective cohorts. *Cardiovasc Diabetol, 24*(1), 366. doi:10.1186/s12933-025-02921-3

[20]Zou, J., Lu, S., Cao, K., He, R., Feng, X., Yang, S., . . . Peng, X. (2025). Association of the triglyceride-glucose index with all-cause and cause-specific mortality in patients with comorbid hypertension and diabetes: A population-based cohort study. *Am Heart J Plus, 60*, 100657. doi:10.1016/j.ahjo.2025.100657

[21]Kim, B. S., Kim, H. J., Moon, S., Kim, H., Lee, J., & Shin, J. H. (2025). Association of the triglyceride-glucose index with cardiovascular outcomes across cardiovascular-kidney-metabolic syndrome stages. *Korean J Intern Med, 40*(6), 961-974. doi:10.3904/kjim.2025.082

[22]Association of the triglyceride-glucose index with subclinical left ventricular dysfunction in type 2 diabetes mellitus patients: A retrospective cross-sectional study.

[23]Lan, P., & Lan, T. (2026). Association of Triglyceride-glucose Index and Obesity Metrics with Cardiovascular Disease Among U.S. Adults with Hyperlipidemia: Findings from the NHANES 2007-2016. *Metab Syndr Relat Disord, 24*(4), 139-147. doi:10.1177/15578518251413507

[24]Xiao, Q., Li, X., Zhou, X., Yao, Z., He, R., Long, Y., & Su, Y. (2025). Association of Triglyceride-Glucose Index With Different Cardiovascular Diseases in Non-Diabetic Hypertension. *J Cell Mol Med, 29*(21), e70925. doi:10.1111/jcmm.70925

[25]Li, M., Zhang, H. P., Cheng, Y. M., Sun, L. X., Chen, S. H., Duan, X. J., . . . Yin, H. Y. (2025). Association of triglyceride-glucose index with risk of cardiovascular disease among patients with prediabetes: population based prospective cohort study. *BMC Cardiovasc Disord, 25*(1), 685. doi:10.1186/s12872-025-05112-w

[26]Wang, M., Lin, W., Liu, H., Qian, Z. M., Chen, J., Rao, M., . . . Chen, G. (2025). Associations between insulin resistance indices and cardiovascular disease in older adults with cardiovascular-kidney-metabolic syndrome stage 0-3: The GOLD-Health cohort. *Diabetes Res Clin Pract, 226*, 112327. doi:10.1016/j.diabres.2025.112327

[27]Tang, Y., Xu, W., Jiang, Y., Liu, C., Zhu, W., Liu, D., . . . Wu, Q. (2026). Associations between lipid-derived indices and cardiovascular-kidney-metabolic syndrome progression among Chinese middle-aged and elderly adults: a longitudinal study. *Front Nutr, 13*, 1742974. doi:10.3389/fnut.2026.1742974

[28]Li, H., Chen, L., Wang, M., Cheng, W., Du, Z., & Huang, Y. (2026). Associations of C-reactive protein, triglyceride-glucose index, and the C-reactive protein-triglyceride glucose index with multistate trajectories in the cardiovascular-renal-diabetes cluster. *Front Endocrinol (Lausanne), 17*, 1758467. doi:10.3389/fendo.2026.1758467

[29]Associations of C-reactive protein-triglyceride-glucose index cumulative burden with incident cardiovascular disease in middle-aged and older Chinese adults with abnormal glucose metabolism.

[30]Wang, K., Luo, X., Wan, D., Su, B., & Li, Y. (2026). Associations of visceral fat accumulation with cardiovascular disease across cardiovascular-kidney-metabolic syndrome stages 0-3: mediation effects of arterial stiffness in a prospective cohort study. *BMC Cardiovasc Disord, 26*(1). doi:10.1186/s12872-026-05775-z

[31]Shen, X., Nan, J., Mou, L., Raj, V. M. S., Kosmas, C. E., Sliman, H., & Yuan, H. (2025). Combination of triglyceride-glucose index and waist-to-height ratio as a predictor of all-cause and cardiovascular mortality in adults with diabetes or prediabetes: a nationwide prospective cohort study. *Cardiovasc Diagn Ther, 15*(5), 937-954. doi:10.21037/cdt-2025-206

[32]Pan, Y., Du, B., Feng, L., & Bi, J. (2025). Comparative analysis of 16 baseline obesity and lipid-related indices for cardiovascular disease risk prediction in adults with cardiovascular-kidney-metabolic syndrome stages 0-3: a nationwide prospective cohort study. *Diabetol Metab Syndr, 17*(1), 343. doi:10.1186/s13098-025-01919-x

[33]Comparison of the Effects of Fasting Glucose, Hemoglobin A1c, and Triglyceride–Glucose Index on Cardiovascular Events in Type 2 Diabetes Mellitus.

[34]Hong, J., Zhang, R., Tang, H., Wu, S., Chen, Y., & Tan, X. (2025). Comparison of triglyceride glucose index and modified triglyceride glucose indices in predicting cardiovascular diseases incidence among populations with cardiovascular-kidney-metabolic syndrome stages 0-3: a nationwide prospective cohort study. *Cardiovasc Diabetol, 24*(1), 98. doi:10.1186/s12933-025-02662-3

[35]Mao, Q., Liu, N., Xiong, T., Wang, X., Tian, X., & Kong, Y. (2025). Comparison of triglyceride-glucose index and triglyceride-glucose related indexes in predicting cardiovascular disease incidence among populations with cardiovascular-kidney-metabolic syndrome stages 0-3: a nationwide prospective cohort study. *Front Endocrinol (Lausanne), 16*, 1562135. doi:10.3389/fendo.2025.1562135

[36]Xu, Y., Chen, S., Zhu, J., Wang, Q., Li, W., Pan, G., . . . Qu, W. (2025). C-reactive protein-triglyceride glucose index and stroke risk in early cardiovascular-kidney-metabolic syndrome: a National cohort study. *BMC Cardiovasc Disord, 25*(1), 634. doi:10.1186/s12872-025-05143-3

[37]Ou, H., Wei, M., Li, X., & Xia, X. (2025). C-reactive protein-triglyceride glucose index in evaluating cardiovascular disease and all-cause mortality incidence among individuals across stages 0-3 of

cardiovascular-kidney-metabolic syndrome: a nationwide prospective cohort study. *Cardiovasc Diabetol, 24*(1), 296. doi:10.1186/s12933-025-02848-9

[38]Ying, Q., He, F., Wu, L., Wei, Q., & Xu, J. (2025). C-reactive protein-triglyceride glucose index predicts mortality in cardiovascular-kidney-metabolic syndrome stage 0-3: a prospective cohort study. *Diabetol Metab Syndr, 17*(1), 382. doi:10.1186/s13098-025-01947-7

[39]Tang, S., Wang, H., Li, K., Chen, Y., Zheng, Q., Meng, J., & Chen, X. (2024). C-reactive protein-triglyceride glucose index predicts stroke incidence in a hypertensive population: a national cohort study. *Diabetol Metab Syndr, 16*(1), 277. doi:10.1186/s13098-024-01529-z

[40]Lan, Y., Wu, Z., Wu, D., Xu, L., Wei, H., Wu, C., . . . Wu, S. (2025). Enhancing cardiovascular risk prediction in hypertensive adults: a 3PM-based evaluation of insulin resistance and arterial stiffness. *EPMA J, 16*(4), 773-783. doi:10.1007/s13167-025-00421-8

[41]Tai, S., Fu, L., Zhang, N., Zhou, Y., Xing, Z., & Wang, Y. (2022). Impact of Baseline and Trajectory of Triglyceride-Glucose Index on Cardiovascular Outcomes in Patients With Type 2 Diabetes Mellitus. *Front Endocrinol (Lausanne), 13*, 858209. doi:10.3389/fendo.2022.858209

[42]Yi, T., Lin, Z., Hu, F., Chen, J., & Chen, L. (2025). Impact of the Triglyceride-Glucose index on all-cause and cardiovascular mortalities across different metabolic health and obesity statuses in US adults. *BMC Public Health, 25*(1), 1767. doi:10.1186/s12889-025-22901-2

[43]Chen, X., Yang, J., Wang, D., Liu, J., Jin, H., Zhang, Y., & Xiang, Q. (2025). Impact of triglyceride-glucose index on risk of cardiovascular disease among non-diabetic hypertension patients: a 10-year prospective cohort study. *BMC Public Health, 25*(1), 326. doi:10.1186/s12889-025-21522-z

[44]Diwon, A., Chen, Y., Chen, Y., Zhang, X., Wang, H., Wang, Z., . . . Mao, G. (2026). Individual and joint effects of overweight/obesity and the triglyceride-glucose index on mortality risk in type 2 diabetic patients: a retrospective cohort study in China. *Front Endocrinol (Lausanne), 17*, 1652682. doi:10.3389/fendo.2026.1652682

[45]Hu, J., Shang, C., Huang, Y., Sun, C., & Zhang, J. (2025). Insulin resistance-related indices, genetic risk, and the risk of cardiovascular disease in individuals with preclinical or clinical obesity: a large prospective cohort study in the UK biobank. *Cardiovasc Diabetol, 24*(1), 407. doi:10.1186/s12933-025-02961-9

[46]Zhao, C., Li, H., Jiang, X., Yang, Y., Yang, Y., Zhang, H., . . . Lin, M. (2026). Insulin-resistance indices and cardiovascular mortality in cardiovascular-kidney-metabolic syndrome stage 2: a comparative analysis of eGDR, TyG and METS-IR. *Diabetol Metab Syndr, 18*(1), 54. doi:10.1186/s13098-026-02089-0

[47]Chen, X., Wang, L., Jiang, Y., Tang, W., Xiao, J., Deng, W., . . . Li, C. (2025). Interplay between triglyceride-glucose index and cardiovascular events in hypertensive patients with controlled blood pressure (SBP<140 mmHg): insights from the SPRINT trial. *Front Endocrinol (Lausanne), 16*, 1508667. doi:10.3389/fendo.2025.1508667

[48]Shen, F. C., Lin, H. Y., Tsai, W. C., Kuo, I. C., Chen, Y. K., Chao, Y. L., . . . Chang, J. M. (2023). Non-insulin-based insulin resistance indices for predicting all-cause mortality and renal outcomes in patients with stage 1-4 chronic kidney disease: another paradox. *Front Nutr, 10*, 1136284. doi:10.3389/fnut.2023.1136284

[49]Cardoso, C. R. L., Castro, G. P., Leite, N. C., & Salles, G. F. (2026). Prognostic value of triglyceride-derived metabolic parameters for micro- and macrovascular complications and mortality in individuals with type 2 diabetes: The Rio de Janeiro type 2 diabetes cohort study. *Diabet Med, 43*(4), e70263. doi:10.1111/dme.70263

[50]Jin, T., Tang, X., Han, Y., Fan, H., Qin, Q., Jiang, H., . . . Wu, M. (2026). Relationship between nine triglyceride-glucose-related indices and cardiometabolic multimorbidity incidence in patients with cardiovascular-kidney-metabolic syndrome stage 0-3: a nationwide prospective cohort study. *Cardiovasc Diabetol, 25*(1), 36. doi:10.1186/s12933-026-03077-4

[51]Qiu, W., Cai, A., Li, L., & Feng, Y. (2024). Systolic blood pressure status modifies the associations between the triglyceride-glucose index and incident cardiovascular disease: a national cohort study in China. *Cardiovasc Diabetol, 23*(1), 135. doi:10.1186/s12933-024-02227-w
[52]Yang, Y., Liu, Z., Lin, B., Huang, Y., Deng, H., Yang, X., . . . Xu, W. (2025). The association between new insulin resistance indices and all-cause mortality in elderly patients with diabetes: a prospective cohort study. *Diabetol Metab Syndr, 17*(1), 181. doi:10.1186/s13098-025-01732-6
[53]Liu, J., Kang, J., Liang, P., Song, Z., Li, G., Jin, X., & Wu, H. (2025). The association between triglyceride-glucose index and all-cause/cardiovascular mortality in patients with different glucose metabolism statuses. *Cardiovasc Diabetol, 24*(1), 367. doi:10.1186/s12933-025-02826-1
[54]Yao, Y., Wang, B., Geng, T., Chen, J., Chen, W., & Li, L. (2024). The association between TyG and all-cause/non-cardiovascular mortality in general patients with type 2 diabetes mellitus is modified by age: results from the cohort study of NHANES 1999-2018. *Cardiovasc Diabetol, 23*(1), 43. doi:10.1186/s12933-024-02120-6
[55]Wang, K., Wang, W., Niu, J., Liu, Y., Wang, Z., Wang, Z., . . . Ge, H. (2025). The combined prognostic effect of the triglyceride-glucose index and estimated glucose disposal rate on long-term mortality among individuals with cardiovascular-kidney-metabolic syndrome stages 0-3. *BMC Cardiovasc Disord, 25*(1), 726. doi:10.1186/s12872-025-05161-1
[56]Ding, L., Fu, B., Zhang, H., Dai, C., Zhang, A., Yu, F., . . . Tang, M. (2024). The impact of triglyceride glucose-body mass index on all-cause and cardiovascular mortality in elderly patients with diabetes mellitus: evidence from NHANES 2007-2016. *BMC Geriatr, 24*(1), 356. doi:10.1186/s12877-024-04992-5
[57]Huang, C., Jia, J., & He, Y. (2025). The nonlinear association between the triglyceride glucose-waist-to-height ratio index and cardiovascular disease among middle-aged and older adults in China with cardiovascular-kidney-metabolic syndrome stages 0-3. *Front Cardiovasc Med, 12*, 1604270. doi:10.3389/fcvm.2025.1604270
[58]Zhao, J., Li, N., Li, S., & Dou, J. (2025). The predictive significance of the triglyceride-glucose index in forecasting adverse cardiovascular events among type 2 diabetes mellitus patients with co-existing hyperuricemia: a retrospective cohort study. *Cardiovasc Diabetol, 24*(1), 218. doi:10.1186/s12933-025-02783-9
[59]Chen, X., Meng, W., Zhu, B., Li, W., Zheng, Y., Yang, X., . . . Zhang, S. (2025). The Prognostic Value of Insulin Resistance Indices for Major Adverse Cardiovascular Events in Young and Middle-Aged Patients With Hypertension. *J Clin Hypertens (Greenwich), 27*(12), e70192. doi:10.1111/jch.70192
[60]Zhou, X., Liu, Y., Peng, J., Zhou, X., & Wei, H. (2025). The role of triglyceride-glucose index in the progression of cardiovascular-kidney-metabolic syndrome: findings from the China health and retirement longitudinal study. *Front Cardiovasc Med, 12*, 1612121. doi:10.3389/fcvm.2025.1612121
[61]Zhang, Q., Xiao, S., Jiao, X., & Shen, Y. (2023). The triglyceride-glucose index is a predictor for cardiovascular and all-cause mortality in CVD patients with diabetes or pre-diabetes: evidence from NHANES 2001-2018. *Cardiovasc Diabetol, 22*(1), 279. doi:10.1186/s12933-023-02030-z
[62]Yang, S., & Wang, Z. (2025). The triglyceride-glucose index is a promising predictor for the risk of cardiovascular disease in the diabetic population aged >/=60 years in the United States: a retrospective cohort study from NHANES (2007-2016). *Front Endocrinol (Lausanne), 16*, 1475590. doi:10.3389/fendo.2025.1475590
[63]Xu, Z., Yan, X., Li, D., & Huang, X. (2025). Triglyceride glucose index as a biomarker for heart failure risk in H-type hypertension patients. *Sci Rep, 15*(1), 4828. doi:10.1038/s41598-025-89211-w
[64]Zhao, M., Xiao, M., Tan, Q., & Lu, F. (2023). Triglyceride glucose index as a predictor of mortality in middle-aged and elderly patients with type 2 diabetes in the US. *Sci Rep, 13*(1), 16478. doi:10.1038/s41598-023-43512-0

[65]Zhang, F., Kadierding, R., Zhang, S., Yang, H., Ren, L., Ren, M., . . . Zhang, Y. (2023). Triglyceride Glucose Index for Predicting Major Adverse Cardiovascular Events in Chinese Patients with Hypertension. *Angiology, 74*(3), 259-267. doi:10.1177/00033197221104559

[66]Sbriscia, M., Colombaretti, D., Giuliani, A., Di Valerio, S., Scisciola, L., Rusanova, I., . . . Sabbatinelli, J. (2025). Triglyceride glucose index predicts long-term mortality and major adverse cardiovascular events in patients with type 2 diabetes. *Cardiovasc Diabetol, 24*(1), 115. doi:10.1186/s12933-025-02671-2

[67]Wang, L., Li, Z., Qiu, R., Luo, L., & Yan, X. (2025). Triglyceride glucose index-body mass index as a predictor of coronary artery disease severity in patients with H-type hypertension across different glucose metabolic states. *Diabetol Metab Syndr, 17*(1), 15. doi:10.1186/s13098-024-01568-6

[68]Liu, M. J., Xiang, S. M., & Hu, X. Q. (2025). Triglyceride glucose-body mass index is associated with cardiovascular outcomes and overall mortality in type-2 diabetes mellitus patients. *World J Diabetes, 16*(8), 108839. doi:10.4239/wjd.v16.i8.108839

[69]Ning, P., Zeng, J., Feng, Q., Chen, M., & Cao, H. (2024). Triglyceride-Glucose Index as a Predictor of Lower Extremity Arterial Disease in Patients with Diabetes: A Hospitalized Population Retrospective Study. *Ann Vasc Surg, 98*, 173-181. doi:10.1016/j.avsg.2023.08.013

[70]Cho, Y. K., Kim, H. S., Park, J. Y., Lee, W. J., Kim, Y. J., & Jung, C. H. (2022). Triglyceride-Glucose Index Predicts Cardiovascular Outcome in Metabolically Unhealthy Obese Population: A Nationwide Population-Based Cohort Study. *J Obes Metab Syndr, 31*(2), 178-186. doi:10.7570/jomes21086

[71]Liu, M. J., Pei, J. Y., Zeng, C., Xing, Y., Zhang, Y. F., Tang, P. Q., . . . Hu, X. Q. (2025). Triglyceride-glucose related indices as predictors for major adverse cardiovascular events and overall mortality in type-2 diabetes mellitus patients. *World J Diabetes, 16*(3), 101488. doi:10.4239/wjd.v16.i3.101488

[72]Liu, K., Hu, J., Huang, Y., He, D., & Zhang, J. (2025). Triglyceride-glucose-related indices and risk of cardiovascular disease and mortality in individuals with cardiovascular-kidney-metabolic (CKM) syndrome stages 0-3: a prospective cohort study of 282,920 participants in the UK Biobank. *Cardiovasc Diabetol, 24*(1), 277. doi:10.1186/s12933-025-02842-1

[73]Shi, Y., & Yu, C. (2024). U shape association between triglyceride glucose index and congestive heart failure in patients with diabetes and prediabetes. *Nutr Metab (Lond), 21*(1), 42. doi:10.1186/s12986-024-00819-7

[74]Li, H. J., Zhang, L. G., Xu, H., Wang, Y. X., Liu, S., & Liu, J. Y. (2025). Association between triglyceride-glucose index and major adverse cardiovascular events in patients with intermediate coronary stenosis: a prospective cohort study. *Front Endocrinol (Lausanne), 16*, 1663832. doi:10.3389/fendo.2025.1663832

[75]Yang, S., Shi, X., Liu, W., Wang, Z., Li, R., Xu, X., . . . Xu, T. (2023). Association between triglyceride glucose-body mass index and heart failure in subjects with diabetes mellitus or prediabetes mellitus: a cross-sectional study. *Front Endocrinol (Lausanne), 14*, 1294909. doi:10.3389/fendo.2023.1294909

[76]Kwak, J., Han, K. D., Lee, E. Y., Lee, S. H., Lim, D. J., Kwon, H. S., & Lee, J. (2025). Association between the Triglyceride-Glucose Index and Cardiovascular Risk and Mortality across Different Diabetes Durations: A Nationwide Cohort Study. *Endocrinol Metab (Seoul), 40*(4), 548-560. doi:10.3803/EnM.2024.2205

[77]Yao, H., Sun, Z., Yuan, W., Shao, C., Cai, H., Li, L., . . . Wang, Z. (2022). Relationship Between the Triglyceride-Glucose Index and Type 2 Diabetic Macroangiopathy: A Single-Center Retrospective Analysis. *Diabetes Metab Syndr Obes, 15*, 3483-3497. doi:10.2147/DMSO.S387040

[78]Li, C., Zhang, Y., Wu, X., Liu, K., Wang, W., Qin, Y., . . . Song, L. (2024). Prognostic value of the triglyceride-glucose index for adverse cardiovascular outcomes in young adult hypertension. *Clin Hypertens, 30*(1), 25. doi:10.1186/s40885-024-00274-9

[79]Zhang, N., Chi, X., Zhou, Z., Song, Y., Li, S., Xu, J., & Li, J. (2023). Triglyceride-glucose index is associated with a higher risk of stroke in a hypertensive population. *Cardiovasc Diabetol, 22*(1), 346. doi:10.1186/s12933-023-02082-1
[80]Liu, J., Fu, Y., Liang, P., Song, Z., Li, Y., & Wu, H. (2025). The association between novel metabolic parameters and all-cause/cardiovascular mortality in patients with metabolic syndrome is modified by age. *Cardiovasc Diabetol, 24*(1), 96. doi:10.1186/s12933-025-02587-x

Table S4. Sensitivity analysis of inferred CKM and explicit CKM cohorts: summary relative risks of target outcome for TyG and derived indicators

(A) per 1-SD increase

**Indicator**	**Outcome**	**RR (95% CI) Inferred CKM**	**I²(%)**	**RR (95% CI) Explicit CKM**	**I²(%)**
TyG	CVD incidence	1.204 (1.132-1.280)	88.8	1.066 (1.026-1.107)	0.0
TyG-BMI	CVD incidence	1.181 (1.120-1.246)	77.2	1.060 (1.019-1.102)	72.4
TyG-WC	CVD incidence	1.179 (1.100-1.263)	97.8	1.150 (1.103-1.199)	0.0
TyG-WHtR	CVD incidence	1.192 (1.141-1.245)	72.4	1.124 (1.007-1.255)	91.7
CTI	Stroke incidence	1.187 (1.085-1.300)	0.0	1.267 (1.197-1.341)	0.0

(B) Highest versus lowest category

**Indicator**	**Outcome**	**RR (95% CI) Inferred CKM**	**I²(%)**	**RR (95% CI) Explicit CKM**	**I²(%)**
TyG	CVD incidence	1.342 (1.252-1.439)	87.7	1.229 (1.094-1.381)	97.7
TyG	CHD incidence	1.431 (1.281-1.598)	87.2	1.644 (1.465-1.845)	95.4
TyG	Stroke incidence	1.207 (1.069-1.362)	90.0	1.154 (1.083-1.230)	90.7
TyG-BMI	CVD incidence	1.817 (1.523-2.169)	92.7	1.393 (1.180-1.644)	92.3
TyG-WC	CVD incidence	1.569 (1.395-1.766)	85.8	1.391 (1.162-1.666)	94.6
TyG-WHtR	CVD incidence	1.510 (1.299-1.755)	84.5	1.397 (1.171-1.668)	94.5
CTI	CVD incidence	2.408 (1.108-5.233)	95.8	1.449 (1.279-1.642)	20.3

Table S5. Sensitivity analysis excluding cross-sectional studies: summary relative risks of target outcome for TyG and derived indicators

Indicator	Outcome	RR (95% CI) Cross-sectional study	I^2(%)	RR (95% CI) Non-cross-sectional study	I^2(%)
**Per 1-SD increase**					
TyG	CVD mortality	1.178 (1.052-1.319)	0.0	1.250 (1.075-1.454)	79.9
**Highest versus lowest category**					
TyG	All-cause mortality	1.265 (1.042-1.536)	0.0	1.156 (1.085-1.233)	84.8
TyG	CVD mortality	1.457 (0.961-2.208)	59.6	1.167 (1.031-1.322)	51.4
TyG-BMI	CVD mortality	2.506 (1.434-4.380)	0.0	1.180 (0.759-1.832)	87.1
TyG-WC	CVD mortality	2.607 (1.247-5.450)	0.0	1.497 (1.336-1.678)	0.0
TyG-WHtR	CVD mortality	3.325 (1.536-7.199)	0.0	1.475 (1.328-1.639)	0.0

Table S6. Risk bias of each included cohort by the ROBINS-E tool.

Study ID	Domain 1	Domain 2	Domain 3	Domain 4	Domain 5	Domain 6	Domain 7	Overall
Dan Zhou 2022	Low	Low	Low	Moderate	Low	Low	Low	Low
Zhihui Lu 2025	Low	Low	Low	Moderate	Low	Moderate	Low	Low
Wei Xian 2026	Low	Low	Low	Moderate	Low	Moderate	Low	Low
Jintao Chen 2025	Low	Low	Low	Moderate	Low	Moderate	Low	Low
YaJie Zhao 2025	Moderate	Low	Low	Moderate	Low	Low	Low	Low
Ze-Jiaxin Niu 2026	Moderate	Low	Low	Moderate	Low	Low	Low	Low
Yafang Chen 2025	High	Moderate	High	Moderate	Low	Moderate	Low	High
Weipeng Li 2024	Low	Low	Low	Moderate	Low	Moderate	Low	Low
Yuyu Cui 2025	Low	Low	Low	Moderate	Low	Moderate	Low	Low
Chang Liu 2024	Moderate	Low	Low	Moderate	Low	Moderate	Low	Moderate
Xiandu Jin 2026	Low	Low	Moderate	Moderate	Low	Moderate	Low	Moderate
Yanjuan Chen 2025	Low	Low	Moderate	Moderate	Low	Moderate	Low	Moderate
Xinyi Shao 2025	Low	Low	Moderate	Moderate	Low	Moderate	Low	Moderate
Keyu Bian 2025	Low	Low	Low	Low	Low	Low	Low	Low
Peng Zhang 2025	Low	Low	Low	Moderate	Low	Low	Low	Low
Xiaoyuan Wei 2024	Low	Low	Low	Moderate	Low	Low	Low	Low
Yong Yue 2025	Low	Low	Low	Moderate	Low	Moderate	Low	Low
Chang Liu 2025	Low	Low	Low	Moderate	Low	Moderate	Low	Low
Mingjie Chen 2025	Low	Low	Low	Moderate	Low	Moderate	Low	Low
Jiahao Zou 2025	Low	Low	Moderate	Low	Moderate	Low	Low	Low
Byung Sik Kim 2025	Moderate	Low	Low	Low	Low	Low	Low	Low
Qi-chao Sun 2023	Moderate	Low	Low	Low	Low	Moderate	Low	Low

Peixin Lan 2026	Moderate	Low	Low	Moderate	Low	High	Low	High
Qiyu Xiao 2025	Low	Low	Low	Moderate	Low	Moderate	Low	Low
Man Li 2025	Low	Low	Low	Moderate	Low	Moderate	Low	Low
Menghe Wang 2025	Low	Low	Low	Moderate	Low	Moderate	Low	Low
Yu Tang 2026	Low	Low	Low	Moderate	Low	Moderate	Low	Low
Hui Li 2026	Low	Low	Low	Moderate	Low	Moderate	Low	Low
Hao Li 2026	Low	Low	Low	Moderate	Moderate	Moderate	Low	Moderate
Ke Wang 2026	Low	Low	Low	Moderate	Moderate	Moderate	Low	Moderate
Xiaoran Shen 2025	Low	Low	Low	Moderate	Moderate	Low	Low	Low
Yilin Pan 2025	Low	Low	Low	Moderate	Moderate	Moderate	Low	Moderate
Wei-Yu Su 2019	Low	Low	Moderate	Moderate	Moderate	Low	Low	Moderate
Jianan Hong 2025	Low	Low	Low	Moderate	Moderate	Moderate	Low	Moderate
Qingsong Mao 2025	Low	Low	Low	Moderate	Moderate	Moderate	Low	Moderate
Yinsong Xu 2025	Low	Low	Low	Moderate	Moderate	Moderate	Low	Moderate
Huiwen Ou 2025	Low	Low	Low	Moderate	Moderate	Moderate	Low	Moderate
Qianliang Ying 2025	Low	Low	Low	Moderate	Low	Low	Low	Low
Songyuan Tang 2024	Low	Low	Low	Moderate	Low	Low	Low	Low
Yulong Lan 2025	Low	Low	Low	Moderate	Low	Low	Low	Low
Shi Tai 2022	Low	Low	Low	Moderate	Low	Low	Low	Low
Tao Yi 2025	Low	Low	Low	Moderate	Low	Low	Low	Low
Xin Chen 2025	Low	Low	Low	Moderate	Low	Low	Low	Low
Anthony Diwon 2026	Moderate	Low	Moderate	Moderate	Low	Low	Low	Moderate
Jinling Hu 2025	Low	Low	Low	Moderate	Low	Low	Low	Low
Chuanwei Zhao 2026	Low	Low	Low	Moderate	Low	Low	Low	Low
Xiaoling Chen	Low	Low	Low	Low	Low	Low	Low	Low

2025								
Feng-Ching Shen 2023	Low	Low	Low	Low	Low	Low	Low	Low
Claudia R. L. Cardoso 2026	Low	Low	Moderate	Moderate	Low	Low	Low	Low
Tonglong Jin 2026	Low	Low	Low	Moderate	Low	Moderate	Low	Low
Weida Qiu 2024	Moderate	Low	Moderate	Moderate	Low	Moderate	Low	Moderate
Yanling Yang 2025	Low	Low	Low	Moderate	Low	Low	Low	Low
Jiajun Liu 2025	Low	Low	Low	Low	Low	Low	Low	Low
Younan Yao 2024	Low	Low	Low	Moderate	Low	Low	Low	Low
Kexin Wang 2025	Low	Low	Low	Moderate	Low	Low	Low	Low
Lei Ding 2024	Low	Low	Low	Moderate	Moderate	Low	Low	Low
Cheng Huang 2025	Low	Low	Low	Moderate	Low	Moderate	Low	Low
Jianyong Zhao 2025	Moderate	Low	Moderate	Moderate	Low	Low	Low	Moderate
Xi Chen 2025	Moderate	Low	Moderate	Moderate	Low	Low	Low	Moderate
Xingyu Zhou 2025	Low	Low	Low	Moderate	Low	Low	Low	Low
Qin Zhang 2023	Low	Low	Low	Moderate	Low	Low	Low	Low
Shu Yang 2025	Low	Low	Low	Moderate	Low	Low	Low	Low
Zhengwen Xu 2025	Moderate	Low	Moderate	Moderate	Low	Low	Low	Moderate
Mengjie Zhao 2023	Low	Low	Low	Moderate	Low	Low	Low	Low
Fomin Zhang 2023	Low	Low	Low	Moderate	Low	Low	Low	Low
Matilde Sbriscia 2025	Low	Low	Low	Moderate	Low	Low	Low	Low
Lian Wang 2025	Moderate	Low	Moderate	Moderate	Low	Low	Low	Moderate
Mao-Jun Liu 2025	Low	Low	Low	Moderate	Low	Low	Low	Low
Mao-Jun Liu 2025	Low	Low	Low	Moderate	Low	Low	Low	Low
Peng Ning 2024	Moderate	Low	Moderate	Moderate	Low	Moderate	Low	Moderate
Yun Kyung Cho 2022	Low	Low	Low	Moderate	Low	Low	Low	Low
Kun Liu 2025	Low	Low	Low	Moderat	Low	Low	Low	Low

				e				
Yumeng Shi 2024	Moderate	Low	Low	Moderate	Low	Moderate	Low	Moderate
Hui-juan Li 2025	Moderate	Low	High	Moderate	Moderate	Moderate	Low	High
Shuping Yang 2023	Moderate	Low	Moderate	High	Low	High	Low	High
Jeongeun Kwak 2025	Low	Low	Moderate	Moderate	Low	Moderate	Low	Moderate
Haipeng Yao 2022	Moderate	Low	Moderate	Moderate	Low	Low	Low	Moderate
Chen Li 2024	Moderate	Low	High	Low	Low	Moderate	Low	Low
Nan Zhang 2023	Moderate	Low	Low	Moderate	Low	Moderate	Low	Moderate
Jiajun Liu 2025	Low	Low	Low	Moderate	Low	Low	Low	Low

Risk Of Bias In Non-randomized Studies - of Exposures (ROBINS-E) tool was used for assessing risk of bias in each cohort. Seven separate domains (ie, domain 1: bias due to confounding; domain 2: bias arising from measurement of the exposure; domain 3: bias in selection of participants into the study; domain 4: bias due to post-exposure interventions; domain 5: bias due to missing data; domain 6: bias arising from measurement of the outcome; and domain 7: bias in selection of the reported result) were identified before reaching the overall risk of bias.

Table S7. Subgroup analysis of the association between TyG index (per 1-SD increase) and risk of all-cause mortality. TyG, Triglyceride-glucose; SD, Standard Deviation.

Category	Subgroup analysis	N	RR (95% CI)	p for RR	I^2	P for I^2	Q
Age	Age < 60	10	1.384 (1.301-1.472)	<0.0001	0.66	0.0019	26.25
	Age > 60	8	1.078 (1.014-1.146)	0.0158	0.7	0.0016	23.08
Diabetes	Diabetes	5	1.063 (1.003-1.126)	0.0405	0.34	0.1957	6.05
	No diabetes	2	1.35 (1.219-1.495)	<0.0001	0	1	0
Ethnic	American	15	1.219 (1.17-1.269)	<0.0001	0.79	<0.0001	65.68
	Others	3	1.252 (1.096-1.431)	0.001	0.73	0.0256	7.33
Follow up, years	>8.32	13	1.039 (1.024-1.053)	<0.0001	0.89	<0.0001	113.59
	≤8.32	5	0.975 (0.918-1.036)	0.4183	0.9	<0.0001	40.15
Male(%)	>50%	15	1.13 (1.088-1.174)	<0.0001	0.88	<0.0001	120.56
	≤50%	5	1.025 (1.01-1.04)	8e-04	0.79	<0.0001	18.86
Study design	Cross-sectional study	2	1.148 (1.04-1.268)	0.0062	0	0.9204	0.01
	Prospective cohort study	7	1.024 (1.01-1.039)	0.001	0.69	0.0033	19.57
Study design	Retrospective cohort study	11	1.148 (1.098-1.199)	<0.0001	0.91	<0.0001	114.59
	NHANES	15	1.219 (1.17-1.269)	<0.0001	0.79	<0.0001	65.68
	Others	5	1.017 (1.003-1.032)	0.0201	0.86	<0.0001	27.72
Weight	Normal weight	3	1.056 (0.885-1.259)	0.5459	0.26	0.258	2.71
	Obesity	2	2.475 (1.519-4.032)	3e-04	0.49	0.1629	1.95
	Overweight	3	0.957 (0.826-1.108)	0.5532	0.92	<0.0001	25.55

Table S8. Subgroup analysis of the association between TyG index (per 1-SD increase) and risk of CVD mortality; TyG, Triglyceride-glucose; CVD, Cardiovascular Disease.

Category	Subgroup analysis	N	RR (95% CI)	p for RR	I^2	P for I^2	Q
Age	Age < 60	7	1.436 (1.305-1.581)	<0.0001	0.72	0.0018	21.07
	Age > 60	7	1.263 (1.146-1.39)	<0.0001	0.6	0.0203	14.99
Diabetes	Diabetes	6	1.12 (1.009-1.243)	0.0329	0	0.755	2.64
	No diabetes	4	1.341 (1.169-1.538)	<0.0001	0	0.9848	0.15
Drinking	Ever-Drinker	2	1.357 (1.152-1.599)	3e-04	0	0.6335	0.23
	Never-Drinker	2	1.57 (1.295-1.903)	<0.0001	0.53	0.146	2.11
Ethnic	American	9	1.248 (1.158-1.345)	<0.0001	0.24	0.2341	10.46
	Others	2	1.236 (1.019-1.5)	0.0317	0.88	0.0043	8.15
Follow up, years	>8.32	6	1.246 (1.135-1.368)	<0.0001	0.55	0.0498	11.08
	≤8.32	3	0.893 (0.812-0.982)	0.0197	0.68	0.0446	6.22
Hypertension	Hypertension	2	1.354 (1.175-1.561)	<0.0001	0.54	0.1391	2.19
	Non-Hypertension	2	1.499 (1.093-2.054)	0.0119	0.78	0.0318	4.61
Male(%)	>50%	8	1.099 (1.031-1.171)	0.0037	0.86	<0.0001	49.49
	≤50%	4	1.121 (0.989-1.271)	0.0746	0.17	0.3068	3.61
Smoking	Ever-Smoker	2	1.402 (1.164-1.689)	4e-04	0	0.8087	0.06
	Never-Smoker	2	1.675 (1.367-2.053)	<0.0001	0.25	0.2492	1.33
Study design	Cross-sectional study	2	1.178 (1.052-1.319)	0.0045	0	0.8859	0.02
	Prospective cohort study	5	1.189 (1.056-1.339)	0.0041	0.65	0.0237	11.27
	Retrospective cohort study	5	1.034 (0.956-1.119)	0.4046	0.89	<0.0001	36.47
Study name	NHANES	9	1.248 (1.158-1.345)	<0.0001	0.24	0.2341	10.46
	Others	3	0.934 (0.856-1.019)	0.1249	0.89	<0.0001	18.26
Weight	Normal weight	4	1.553 (1.371-1.76)	<0.0001	0.89	<0.0001	26.45
	Obesity	2	1.556 (1.143-2.12)	0.005	0.7	0.0676	3.34
	Overweight	2	1.721 (1.34-2.209)	<0.0001	0.86	0.0082	6.99

Table S9. Subgroup analysis of the association between TyG index (per 1-SD increase) and risk of CVD incidence; TyG, Triglyceride-glucose; CVD, Cardiovascular Disease.

**Category**	**Subgroup analysis**	**N**	**RR (95% CI)**	**p for RR**	**I^2**	**P for I^2**	**Q**
Age	Age < 60	17	1.058 (1.044-1.072)	<0.0001	0.72	<0.0001	57.68
	Age > 60	24	1.039 (1.026-1.052)	<0.0001	0.88	<0.0001	184.15
CKM Stage	CKM Stage 0	2	0.89 (0.695-1.14)	0.3568	0	1	0
	CKM Stage 0-3	3	1.113 (1.102-1.124)	<0.0001	0.97	<0.0001	62.61
	CKM Stage 1	2	1.32 (1.168-1.492)	<0.0001	0	1	0
	CKM Stage 2	2	1.03 (0.968-1.096)	0.3497	0	1	0
	CKM Stage 3	2	1.09 (1.028-1.156)	0.0039	0	1	0
Diabetes	Diabetes	3	1.264 (1.132-1.412)	<0.0001	0.35	0.2158	3.07
	No diabetes	4	1.01 (0.964-1.057)	0.6844	0.58	0.067	7.16
Ethnic	Chinese	12	1.108 (1.085-1.132)	<0.0001	0.57	0.0077	25.49
	Others	11	1.011 (0.997-1.026)	0.1343	0.86	<0.0001	71.5
Follow up, years	>8.32	10	1.042 (1.03-1.055)	<0.0001	0.92	<0.0001	107.84
	≤8.32	11	1.107 (1.066-1.15)	<0.0001	0.73	<0.0001	37.51
Hypertension	Hypertension	5	1.142 (1.093-1.194)	<0.0001	0	0.5036	3.33
	Non-Hypertension	6	1.135 (1.084-1.188)	<0.0001	0.77	7e-04	21.33
Male(%)	>50%	14	1.242 (1.193-1.294)	<0.0001	0.48	0.0246	24.79
	≤50%	10	1.026 (1.013-1.04)	<0.0001	0.86	<0.0001	64.79
Smoking	Ever-Smoker	4	1.022 (1.007-1.037)	0.0031	0.81	0.0012	15.95
	Never-Smoker	4	1.032 (1.017-1.049)	<0.0001	0.81	0.0012	15.8
Study design	Prospective cohort study	20	1.038 (1.026-1.051)	<0.0001	0.85	<0.0001	123.29
	Retrospective cohort study	5	1.212 (1.162-1.265)	<0.0001	0.43	0.1333	7.05
Study name	CHARLS	5	1.103 (1.071-1.135)	<0.0001	0	0.674	2.34
	Others	19	1.035 (1.021-1.048)	<0.0001	0.88	<0.0001	147.06
Weight	Normal weight	3	1.391 (1.154-1.676)	<0.0001	0	0.6503	0.86
	Obesity	3	1.351 (1.136-1.607)	7e-04	0	0.7756	0.51

Table S10. Subgroup analysis of the association between TyG index (per 1-SD increase) and risk of MACE incidence; TyG, Triglyceride-glucose; MACE, Major Adverse Cardiovascular Event.

Category	Subgroup analysis	N	RR (95% CI)	p for RR	I^2	P for I^2	Q
Age	Age < 60	4	1.408 (1.184-1.675)	<0.0001	0	0.5311	2.2
	Age > 60	6	1.185 (1.112-1.263)	<0.0001	0.78	4e-04	22.62
Diabetes	Diabetes	2	1.514 (1.056-2.171)	0.0242	0.5	0.1564	2.01
	No diabetes	2	1.403 (1.066-1.847)	0.0159	0.27	0.2417	1.37
Follow up, years	>8.32	2	1.152 (1.077-1.232)	<0.0001	0.86	0.0074	7.16
	≤8.32	2	1.442 (1.201-1.732)	<0.0001	0	0.3798	0.77
Weight	Normal weight	2	1.449 (1.137-1.846)	0.0027	0	0.4425	0.59
	Overweight	2	1.472 (1.099-1.972)	0.0095	0	0.9854	0

Table S11. Subgroup analysis of the association between TyG-BMI index (per 1-SD increase) and risk of all-cause mortality. TyG-BMI, Triglyceride Glucose-Body Mass.

Category	Subgroup analysis	N	RR (95% CI)	p for RR	I^2	P for I^2	Q
Diabetes	Diabetes	4	1.018 (1.012-1.024)	<0.0001	0.41	0.163	5.12
	No diabetes	2	1.026 (1.018-1.035)	<0.0001	0.79	0.0296	4.73
Hypertension	Hypertension	4	1.003 (0.998-1.009)	0.2337	0.83	<0.0001	18.06
	Non-Hypertension	3	1.006 (0.997-1.016)	0.1971	0	0.5923	1.05

Table S12. Subgroup analysis of the association between TyG-BMI index (per 1-SD increase) and risk of CVD mortality. TyG-BMI, Triglyceride Glucose-Body Mass. CVD, Cardiovascular Disease.

Category	Subgroup analysis	N	RR (95% CI)	p for RR	I^2	P for I^2	Q
Age	Age < 60	2	1.009 (1.005-1.013)	<0.0001	0.67	0.0839	2.99
	Age > 60	4	0.999 (0.995-1.002)	0.4081	0.73	0.0112	11.09
Diabetes	Diabetes	6	1.005 (1.001-1.008)	0.0104	0.9	<0.0001	49.18
	No diabetes	4	1.003 (1-1.007)	0.0503	0.89	<0.0001	27.11
Hypertension	Hypertension	3	1 (0.997-1.003)	0.8039	0.95	<0.0001	38.67
	Non-Hypertension	3	1.005 (0.999-1.011)	0.0945	0.69	0.0377	6.56

Table S13. Subgroup analysis of the association between TyG-BMI index (per 1-SD increase) and risk of CVD incidence. TyG-BMI, Triglyceride Glucose-Body Mass. CVD, Cardiovascular Disease.

Category	Subgroup analysis	N	RR (95% CI)	p for RR	I^2	P for I^2	Q
Age	Age < 60	14	1.002 (1.002-1.003)	<0.0001	0.97	<0.0001	372.23
	Age > 60	13	1.001 (1.001-1.002)	<0.0001	0.97	<0.0001	408.31
CKM stage	CKM Stage 0	3	1.021 (0.901-1.158)	0.7415	0	0.6846	0.76
	CKM Stage 0-3	3	1.167 (1.157-1.177)	<0.0001	0.98	<0.0001	90.69
	CKM Stage 1	3	1.133 (1.071-1.2)	<0.0001	0.77	0.0125	8.76
	CKM Stage 2	3	1.103 (1.063-1.144)	<0.0001	0.71	0.0322	6.87
	CKM Stage 3	3	1.103 (1.063-1.144)	<0.0001	0.71	0.0311	6.94
Diabetes	Diabetes	9	1.002 (1.001-1.003)	<0.0001	0.97	<0.0001	249.21
	No diabetes	6	1.002 (1.001-1.003)	0.0024	0.96	<0.0001	119.53
	Prediabetes	4	1.004 (1.002-1.005)	<0.0001	0.95	<0.0001	59.89
Ethnic	Chinese	6	1.091 (1.074-1.109)	<0.0001	0.92	<0.0001	59.12
	Others	2	1.157 (1.137-1.177)	<0.0001	0.88	0.004	8.27
	White	2	1.002 (1.002-1.003)	<0.0001	1	<0.0001	496
Follow up, years	>8.32	7	1.002 (1.002-1.003)	<0.0001	0.99	<0.0001	896.35
	≤8.32	3	1.059 (1.04-1.078)	<0.0001	0.72	0.0268	7.24
Hypertension	Hypertension	4	1.001 (1.001-1.002)	<0.0001	0.96	<0.0001	83.44
	Non-Hypertension	4	1.001 (1.001-1.002)	<0.0001	0.95	<0.0001	64.73
Race	Non-white	2	1.003 (1-1.005)	0.0474	0.95	<0.0001	20.89
	White	2	1.002 (1.002-1.003)	<0.0001	0.97	<0.0001	34.14
Smoking	Ever-Smoker	4	1.096 (1.081-1.11)	<0.0001	0.86	<0.0001	20.79
	Never-Smoker	4	1.116 (1.1-1.132)	<0.0001	0.87	<0.0001	23.03
Study name	CHARLS	5	1.09 (1.072-1.109)	<0.0001	0.93	<0.0001	59.04
	Others	5	1.002 (1.002-1.003)	<0.0001	0.99	<0.0001	784.4

Table S14. Subgroup analysis of the association between TyG-WC index (per 1-SD increase) and risk of CVD incidence. TyG-WC, Triglyceride-Glucose-Waist Circumference. CVD, Cardiovascular Disease.

Category	Subgroup analysis	N	RR (95% CI)	p for RR	I^2	P for I^2	Q
Age	Age < 60	9	1.001 (1.001-1.002)	<0.0001	0.97	<0.0001	311.18
	Age > 60	10	1.001 (1.001-1.002)	<0.0001	0.98	<0.0001	447.67
CKM stage	CKM Stage 0	2	1.02 (0.74-1.406)	0.9037	0	1	0
	CKM Stage 0-3	3	1.186 (1.173-1.199)	<0.0001	0.98	<0.0001	89.2
	CKM Stage 1	2	1.45 (1.315-1.598)	<0.0001	0	1	0
	CKM Stage 2	2	1.2 (1.128-1.276)	<0.0001	0	1	0
	CKM Stage 3	2	1.18 (0.924-1.508)	0.1856	0	1	0
Diabetes	Diabetes	2	1 (1-1.001)	0.9715	0.93	1e-04	15.23
	No diabetes	2	1 (1-1.001)	0.0056	0.87	0.0056	7.68
Follow up, years	>8.32	4	1.136 (1.122-1.15)	<0.0001	0.92	<0.0001	37.78
	≤8.32	3	1.036 (1.026-1.046)	<0.0001	0.92	<0.0001	25.84
Smoking	Ever-Smoker	3	1.133 (1.115-1.151)	<0.0001	0.83	0.0024	12.09
	Never-Smoker	3	1.147 (1.126-1.169)	<0.0001	0.7	0.0375	6.57
Study name	CHARLS	5	1.047 (1.037-1.056)	<0.0001	0.97	<0.0001	123.51
	Others	2	1.122 (1.107-1.136)	<0.0001	0	0.3711	0.8

Table S15. Subgroup analysis of the association between TyG-WHtR index (per 1-SD increase) and risk of all-cause mortality. TyG-WHtR, Triglyceride-Glucose Waist-to-Height Ratio.

Category	Subgroup analysis	N	RR (95% CI)	p for RR	I^2	P for I^2	Q
Age	Age < 60	2	1.232 (1.095-1.385)	5e-04	0.44	0.1833	1.77
	Age > 60	4	1.16 (1.121-1.2)	<0.0001	0.19	0.2942	3.71
Ethnic	American	2	1.21 (1.126-1.301)	<0.0001	0.56	0.1331	2.26
	White	3	1.115 (1.101-1.129)	<0.0001	0.33	0.2265	2.97
Hypertension	Hypertension	2	1.272 (1.156-1.398)	<0.0001	0.53	0.1452	2.12
	Non-Hypertension	2	1.08 (0.925-1.261)	0.3313	0	1	0
Male(%)	>50%	3	1.155 (1.113-1.198)	<0.0001	0.4	0.1875	3.35
	≤50%	2	1.112 (1.098-1.127)	<0.0001	0.68	0.0763	3.14
Study design	Prospective cohort study	2	1.112 (1.098-1.127)	<0.0001	0.68	0.0763	3.14
	Retrospective cohort study	2	1.149 (1.107-1.192)	<0.0001	0	1	0
Study name	NHANES	2	1.21 (1.126-1.301)	<0.0001	0.56	0.1331	2.26
	Others	3	1.115 (1.101-1.129)	<0.0001	0.33	0.2265	2.97

Table S16. Subgroup analysis of the association between TyG-WHtR index (per 1-SD increase) and risk of CVD mortality. TyG-WHtR, Triglyceride-Glucose Waist-to-Height Ratio. CVD, Cardiovascular Disease.

Category	Subgroup analysis	N	RR (95% CI)	p for RR	I^2	P for I^2	Q
Age	Age < 60	5	1.727 (1.465-2.036)	<0.0001	0.72	0.0059	14.5
	Age > 60	3	1.121 (1.002-1.255)	0.0458	0	0.427	1.7
CKM stage	CKM Stage 1	2	3.744 (1.204-11.641)	0.0225	0	0.9267	0.01
	CKM Stage 2	2	1.504 (1.322-1.71)	<0.0001	0	0.8406	0.04
Drinking	Ever-Drinker	2	1.412 (1.203-1.657)	<0.0001	0.35	0.2149	1.54
	Never-Drinker	2	1.336 (1.126-1.586)	9e-04	0.71	0.064	3.43
Hypertension	Hypertension	3	1.143 (1.019-1.282)	0.0229	0	0.4663	1.53
	Non-Hypertension	3	1.389 (1.113-1.734)	0.0036	0.3	0.2386	2.87
Smoking	Ever-Smoker	2	1.236 (1.073-1.425)	0.0033	0	0.3802	0.77
	Never-Smoker	2	1.392 (1.207-1.605)	<0.0001	0.92	5e-04	12.28
Study design	Cross-sectional study	3	2.36 (1.745-3.192)	<0.0001	0.11	0.3238	2.26
	Prospective cohort study	2	1.204 (1.166-1.243)	<0.0001	0.37	0.2063	1.6
Weight	Normal weight	2	1.52 (1.29-1.791)	<0.0001	0.97	<0.0001	36.33
	Obesity	2	1.254 (1.092-1.439)	0.0013	0.87	0.0052	7.81

Table S17. Subgroup analysis of the association between TyG-WHtR index (per 1-SD increase) and risk of CVD incidence. TyG-WHtR, Triglyceride-Glucose Waist-to-Height Ratio. CVD, Cardiovascular Disease.

Category	Subgroup analysis	N	RR (95% CI)	p for RR	I^2	P for I^2	Q
Age	Age < 60	13	1.142 (1.126-1.159)	<0.0001	0.84	<0.0001	77.18
	Age > 60	13	1.12 (1.107-1.133)	<0.0001	0.75	<0.0001	47.44
CKM stage	CKM Stage 0	3	1.013 (0.798-1.285)	0.9185	0	0.9798	0.04
	CKM Stage 0-3	3	1.203 (1.192-1.214)	<0.0001	0.98	<0.0001	112.53
	CKM Stage 1	3	1.455 (1.338-1.582)	<0.0001	0	0.5566	1.17
	CKM Stage 2	3	1.214 (1.158-1.273)	<0.0001	0	0.7993	0.45
	CKM Stage 3	3	1.206 (1.149-1.266)	<0.0001	0.06	0.3446	2.13
Diabetes	Diabetes	4	1.16 (1.109-1.213)	<0.0001	0.61	0.0545	7.62
	No diabetes	2	1.08 (1.015-1.149)	0.0151	0	1	0
Drinking	Ever-Drinker	3	1.163 (1.089-1.241)	<0.0001	0.16	0.3038	2.38
	Never-Drinker	3	1.206 (1.155-1.259)	<0.0001	0.77	0.0122	8.82
Dyslipidemia	Dyslipidemia	2	1.09 (0.958-1.24)	0.1898	0	1	0
	Non-Dyslipidemia	2	1.08 (1.015-1.149)	0.0151	0	1	0
Ethnic	Chinese	7	1.163 (1.137-1.189)	<0.0001	0.86	<0.0001	43.02
	White	3	1.178 (1.16-1.196)	<0.0001	0.77	0.0122	8.82
Follow up, years	>8.32	8	1.179 (1.168-1.19)	<0.0001	0.72	8e-04	24.85
	≤8.32	3	1.071 (1.034-1.11)	2e-04	0	0.4536	1.58
Hypertension	Hypertension	3	1.139 (1.091-1.19)	<0.0001	0.15	0.3085	2.35
	Non-Hypertension	3	1.166 (1.096-1.241)	<0.0001	0.24	0.2696	2.62
Male(%)	>50%	2	1.117 (1.075-1.161)	<0.0001	0	1	0
	≤50%	9	1.175 (1.164-1.186)	<0.0001	0.83	<0.0001	46.46
Smoking	Ever-Smoker	6	1.108 (1.092-1.123)	<0.0001	0.72	0.0034	17.63
	Never-Smoker	6	1.12 (1.102-1.138)	<0.0001	0.21	0.2739	6.35
Study design	Prospective cohort study	9	1.175 (1.164-1.186)	<0.0001	0.83	<0.0001	46.46
	Retrospective cohort study	2	1.117 (1.075-1.161)	<0.0001	0	1	0
Study name	CHARLS	6	1.187 (1.158-1.216)	<0.0001	0.81	1e-04	26.08
	Others	5	1.169 (1.158-1.181)	<0.0001	0.84	<0.0001	25.51

Table S18. Subgroup analysis of the association between CTI index (per 1-SD increase) and risk of CVD incidence. CTI, C-Reactive Protein-Triglyceride Glucose. CVD, Cardiovascular Disease.

Category	Subgroup analysis	N	RR (95% CI)	p for RR	I^2	P for I^2	Q
Age	Age < 60	12	1.226 (1.209-1.244)	<0.0001	0.21	0.2379	13.91
	Age > 60	9	1.188 (1.144-1.234)	<0.0001	0.25	0.2218	10.66
CKM stage	CKM Stage 0-1	2	1.025 (0.915-1.149)	0.6721	0	0.3634	0.83
	CKM Stage 3	2	1.211 (1.073-1.367)	0.0019	0	0.741	0.11
Diabetes	Diabetes	3	1.081 (0.985-1.186)	0.1019	0	0.8423	0.34
	No diabetes	4	1.228 (1.177-1.282)	<0.0001	0	0.8063	0.98
Drinking	Ever-Drinker	5	1.226 (1.144-1.313)	<0.0001	0	0.5101	3.29
	Never-Drinker	4	1.25 (1.181-1.322)	<0.0001	0	0.6924	1.46
Dyslipidemia	Dyslipidemia	5	1.166 (1.095-1.242)	<0.0001	0.48	0.1019	7.73
	Non-Dyslipidemia	8	1.264 (1.217-1.314)	<0.0001	0.2	0.2721	8.74
Follow up, years	>8.32	6	1.23 (1.211-1.248)	<0.0001	0.12	0.3359	5.71
	≤8.32	5	1.148 (1.104-1.195)	<0.0001	0	0.5354	3.14
Hypertension	Hypertension	2	1.186 (1.11-1.267)	<0.0001	0	0.72	0.13
	Non-Hypertension	2	1.168 (1.075-1.268)	2e-04	0.3	0.2313	1.43
Smoking	Current-smoker	2	1.206 (1.091-1.333)	3e-04	0	0.3791	0.77
	Ever-Smoker	4	1.241 (1.112-1.385)	1e-04	0	0.493	2.4
	Never-Smoker	4	1.238 (1.17-1.309)	<0.0001	0	0.4624	2.57
Weight	Normal weight	3	1.229 (1.156-1.306)	<0.0001	0	0.6723	0.79
	Obesity	3	1.154 (1.009-1.32)	0.0362	0	0.3893	1.89
	Overweight	2	1.057 (0.957-1.167)	0.2754	0	0.8889	0.02

Table S19. Subgroup analysis of the association between CTI index (per 1-SD increase) and risk of stroke incidence. CTI, C-Reactive Protein-Triglyceride Glucose.

Category	Subgroup analysis	N	RR (95% CI)	p for RR	I^2	P for I^2	Q
Age	Age < 60	7	1.249 (1.191-1.309)	<0.0001	0	0.7941	3.12
	Age > 60	4	1.208 (1.118-1.304)	<0.0001	0	0.6583	1.6
Diabetes	Diabetes	2	1.099 (0.943-1.282)	0.2265	0	0.605	0.27
	No diabetes	2	1.191 (1.051-1.349)	0.006	0	0.5085	0.44
Drinking	Ever-Drinker	2	1.247 (1.071-1.451)	0.0045	0.68	0.0769	3.13
	Never-Drinker	2	1.271 (1.129-1.431)	<0.0001	0	0.3983	0.71
Dyslipidemia	Dyslipidemia	2	1.079 (0.97-1.201)	0.1638	0	0.6743	0.18
	Non-Dyslipidemia	2	1.58 (1.304-1.915)	<0.0001	0.37	0.2094	1.58
Follow up, years	>8.32	2	1.267 (1.197-1.341)	<0.0001	0	0.3816	0.77
	≤8.32	3	1.187 (1.085-1.3)	<0.0001	0	0.8451	0.34
Smoking	Ever-Smoker	4	1.206 (1.05-1.385)	0.0081	0	0.4093	2.89
	Never-Smoker	2	1.31 (1.158-1.481)	<0.0001	0	0.673	0.18

Table S20. Subgroup analysis of the association between categorized TyG index (highest vs. reference category) and risk of all-cause mortality. TyG, Triglyceride-glucose.

**Category**	**Subgroup analysis**	**N**	**RR (95% CI)**	**p for RR**	**I^2**	**P for I^2**	**Q**
Age	Age < 60	15	1.11 (1.094-1.126)	<0.0001	0.84	<0.0001	88.66
	Age > 60	16	1.059 (0.999-1.123)	0.0527	0.54	0.0052	32.7
Diabetes	Diabetes	9	1.101 (1.014-1.196)	0.0216	0.39	0.1067	13.15
	No diabetes	5	1.637 (1.373-1.951)	<0.0001	0.66	0.018	11.92
	Prediabetes	5	1.39 (1.215-1.591)	<0.0001	0.7	0.009	13.51
Ethnic	American	12	1.182 (1.096-1.275)	<0.0001	0.67	<0.0001	33.28
	Others	8	1.107 (1.091-1.124)	<0.0001	0.91	<0.0001	74.91
	White	2	1.058 (1.013-1.104)	0.0102	0.83	0.0154	5.87
Follow up, years	>8.32	10	1.064 (1.046-1.081)	<0.0001	0.7	<0.0001	29.91
	≤8.32	9	1.181 (1.153-1.211)	<0.0001	0.82	<0.0001	44.38
Male(%)	>50%	17	1.103 (1.087-1.119)	<0.0001	0.84	<0.0001	98.58
	≤50%	6	1.094 (1.052-1.137)	<0.0001	0.86	<0.0001	35.41
Race	Non-white	5	1.309 (1.014-1.69)	0.0388	0	0.5675	2.94
	White	2	1.16 (0.965-1.395)	0.115	0	1	0
Smoking	Ever-Smoker	3	1.351 (1.123-1.626)	0.0014	0.56	0.1012	4.58
	Never-Smoker	2	1.311 (1.025-1.676)	0.0309	0	0.356	0.85
Study design	Cross-sectional study	2	1.265 (1.042-1.536)	0.0175	0	0.6199	0.25
	Prospective cohort study	11	1.079 (1.039-1.119)	<0.0001	0.74	<0.0001	38.9
Study name	NHANES	15	1.22 (1.145-1.299)	<0.0001	0.65	<0.0001	40.46
	Others	8	1.097 (1.081-1.112)	<0.0001	0.92	<0.0001	83.33
Weight	Normal weight	6	1.379 (1.137-1.674)	0.0011	0.75	0.0013	19.92
	Overweight	9	1.33 (1.197-1.479)	<0.0001	0.46	0.0649	14.72

Table S21. Subgroup analysis of the association between categorized TyG index (highest vs. reference category) and risk of CVD mortality. TyG, Triglyceride-glucose. CVD, Cardiovascular Disease.

Category	Subgroup analysis	N	RR (95% CI)	p for RR	I^2	P for I^2	Q
Age	Age < 60	13	1.131 (1.021-1.254)	0.0186	0.3	0.1482	17.04
	Age > 60	9	1.1 (0.95-1.272)	0.2026	0.32	0.1645	11.71
Diabetes	Diabetes	13	1.211 (1.192-1.231)	<0.0001	0.71	<0.0001	42.02
	No diabetes	6	0.995 (0.923-1.073)	0.8975	0.56	0.0465	11.26
	Prediabetes	5	1.785 (1.392-2.29)	<0.0001	0.25	0.2524	5.36
Ethnic	American	13	1.304 (1.142-1.488)	<0.0001	0.13	0.3155	13.77
	Others	4	1.091 (0.97-1.227)	0.1483	0.69	0.0201	9.83
	White	2	1.026 (0.922-1.141)	0.6378	0	0.551	0.36
Follow up, years	>8.32	6	1.09 (0.99-1.199)	0.0794	0.52	0.0664	10.33
	≤8.32	7	0.999 (0.898-1.111)	0.9816	0.59	0.0245	14.51
Male(%)	>50%	11	1.159 (1.03-1.304)	0.0141	0.62	0.0037	26.03
	≤50%	9	1.054 (0.975-1.139)	0.1865	0.3	0.1811	11.38
Race	Non-white	5	2.178 (1.391-3.409)	<0.0001	0	0.7795	1.76
	White	2	1.59 (1.078-2.345)	0.0193	0	1	0
Smoking	Ever-Smoker	3	1.855 (1.33-2.588)	<0.0001	0	0.6954	0.73
	Never-Smoker	2	1.335 (0.848-2.1)	0.212	0	0.3747	0.79
Study design	Cross-sectional study	5	1.274 (1.038-1.562)	0.0204	0.48	0.1026	7.72
	Prospective cohort study	8	1.11 (1.017-1.212)	0.0192	0.45	0.0786	12.74
	Retrospective cohort study	7	0.998 (0.895-1.114)	0.9766	0.57	0.0313	13.86
Study name	NHANES	16	1.298 (1.157-1.455)	<0.0001	0	0.5399	13.81
	Others	4	0.996 (0.92-1.078)	0.9187	0.74	0.0098	11.39
Weight	Normal weight	6	0.941 (0.794-1.116)	0.4861	0	0.5564	3.95
	Overweight	6	1.564 (1.321-1.852)	<0.0001	0.5	0.0756	9.99

Table S22. Subgroup analysis of the association between categorized TyG index (highest vs. reference category) and risk of CVD incidence. TyG, Triglyceride-glucose. CVD, Cardiovascular Disease.

Category	Subgroup analysis	N	RR (95% CI)	p for RR	I^2	P for I^2	Q
Age	Age < 60	25	1.204 (1.189-1.219)	<0.0001	0.95	<0.0001	447.44
	Age > 60	18	1.202 (1.166-1.239)	<0.0001	0.74	<0.0001	65.02
CKM Stage	CKM Stage 0	2	1.1 (0.585-2.067)	0.7671	0	1	0
	CKM Stage 0-3	3	1.295 (1.261-1.33)	<0.0001	0.96	<0.0001	56.04
	CKM Stage 1	2	0.71 (0.439-1.148)	0.1621	0	1	0
Diabetes	Diabetes	9	1.313 (1.29-1.337)	<0.0001	0.87	<0.0001	59.72
	No diabetes	6	1.236 (1.206-1.265)	<0.0001	0.88	<0.0001	40.68
Dringking	Ever-Drinker	2	1.251 (1.06-1.477)	0.0082	0.7	0.0668	3.36
	Never-Drinker	2	1.418 (1.226-1.639)	<0.0001	0.82	0.0178	5.62
Ethnic	Chinese	17	1.264 (1.217-1.313)	<0.0001	0.76	<0.0001	67.88
	Others	15	1.182 (1.168-1.197)	<0.0001	0.97	<0.0001	480.36
	White	4	1.211 (1.18-1.244)	<0.0001	0.88	<0.0001	25.88
Follow up, years	>8.32	16	1.165 (1.151-1.18)	<0.0001	0.97	<0.0001	478.25
	≤8.32	17	1.265 (1.241-1.29)	<0.0001	0.7	<0.0001	54.24
Hypertension	Hypertension	7	1.298 (1.124-1.498)	4e-04	0.58	0.025	14.45
	Non-Hypertension	5	1.678 (1.397-2.016)	<0.0001	0.48	0.1007	7.76
Male(%)	>50%	21	1.203 (1.189-1.218)	<0.0001	0.96	<0.0001	453.06
	≤50%	15	1.161 (1.137-1.185)	<0.0001	0.89	<0.0001	121.88
Smoking	Ever-Smoker	4	1.361 (1.218-1.521)	<0.0001	0.25	0.2641	3.98
	Never-Smoker	3	1.546 (1.386-1.724)	<0.0001	0.47	0.1506	3.79
Study design	Prospective cohort study	26	1.177 (1.152-1.202)	<0.0001	0.88	<0.0001	206.66
	Retrospective cohort study	11	1.2 (1.185-1.214)	<0.0001	0.97	<0.0001	384.55
Study name	CHARLS	6	1.246 (1.142-1.359)	<0.0001	0.28	0.2282	6.9
	Others	30	1.192 (1.179-1.205)	<0.0001	0.95	<0.0001	578.47
Weight	Normal weight	4	1.189 (1.105-1.28)	<0.0001	0	0.4856	2.44
	Obesity	2	1.754 (1.51-2.036)	<0.0001	0.24	0.2509	1.32
	Overweight	4	1.309 (1.207-1.419)	<0.0001	0.29	0.2384	4.22

Table S23. Subgroup analysis of the association between categorized TyG index (highest vs. reference category) and risk of stroke incidence. TyG, Triglyceride-glucose.

**Category**	**Subgroup analysis**	N	**RR (95% CI)**	**p for RR**	**I^2**	**P for I^2**	**Q**
Age	Age < 60	6	1.24 (1.216-1.264)	<0.0001	0.88	<0.0001	43.44
	Age > 60	3	2.118 (1.687-2.659)	<0.0001	0	0.9658	0.07
Ethnic	Chinese	3	1.344 (1.185-1.525)	<0.0001	0.61	0.0746	5.19
	Others	5	1.243 (1.219-1.268)	<0.0001	0.93	<0.0001	53.9
	White	2	1.064 (1.013-1.118)	0.014	0.71	0.065	3.4
Follow up, years	>8.32	5	1.17 (1.145-1.196)	<0.0001	0.89	<0.0001	38.02
	≤8.32	4	1.347 (1.302-1.392)	<0.0001	0.64	0.0381	8.42
Male(%)	>50%	4	1.255 (1.23-1.28)	<0.0001	0.9	<0.0001	30.94
	≤50%	5	1.059 (1.014-1.107)	0.0103	0.79	<0.0001	19.39
Study design	Prospective cohort study	8	1.083 (1.038-1.129)	2e-04	0.81	<0.0001	36.33
	Retrospective cohort study	2	1.254 (1.229-1.279)	<0.0001	0.96	<0.0001	23.19

Table S24. Subgroup analysis of the association between categorized TyG-BMI index (highest vs. reference category) and risk of all-cause mortality. TyG-BMI, Triglyceride Glucose-Body Mass.

**Category**	**Subgroup analysis**	N	**RR (95% CI)**	**p for RR**	**I^2**	**P for I^2**	**Q**
Age	Age < 60	2	0.99 (0.669-1.466)	0.96	0	1	0
	Age > 60	5	1.135 (1.04-1.238)	0.0043	0.94	<0.0001	69.4
Ethnic	American	3	1.004 (0.732-1.378)	0.9786	0.86	<0.0001	14.74
	White	3	1.156 (1.109-1.204)	<0.0001	0.89	<0.0001	18.83
Follow up, years	>8.32	3	1.156 (1.109-1.204)	<0.0001	0.89	<0.0001	18.83
	≤8.32	2	0.674 (0.459-0.99)	0.0442	0.48	0.1661	1.92
Study design	Prospective cohort study	2	1.109 (1.06-1.16)	<0.0001	0	0.593	0.29
	Retrospective cohort study	3	1.366 (1.236-1.509)	<0.0001	0.86	<0.0001	14
Study name	NHANES	3	1.004 (0.732-1.378)	0.9786	0.86	<0.0001	14.74
	Others	3	1.156 (1.109-1.204)	<0.0001	0.89	<0.0001	18.83

Table S25. Subgroup analysis of the association between categorized TyG-BMI index (highest vs. reference category) and risk of CVD incidence. TyG-BMI, Triglyceride Glucose-Body Mass. CVD, Cardiovascular Disease.

Category	Subgroup analysis	N	RR (95% CI)	p for RR	I^2	P for I^2	Q
Age	Age < 60	4	1.237 (1.164-1.315)	<0.0001	0.89	<0.0001	26.76
	Age > 60	5	1.375 (1.297-1.457)	<0.0001	0.96	<0.0001	98.23
Diabetes	Diabetes	4	1.347 (1.079-1.681)	0.0084	0.8	0.0016	15.32
	No diabetes	7	1.138 (1.058-1.224)	5e-04	0.74	8e-04	23.06
Ethnic	Chinese	7	1.329 (1.261-1.401)	<0.0001	0.9	<0.0001	58.93
	White	4	1.434 (1.386-1.484)	<0.0001	0.97	<0.0001	86.24
Follow up, years	>8.32	8	1.41 (1.375-1.447)	<0.0001	0.94	<0.0001	113.95
	≤8.32	4	1.21 (1.138-1.286)	<0.0001	0.85	1e-04	20.49
Male(%)	>50%	2	2.65 (2.224-3.158)	<0.0001	0	1	0
	≤50%	10	1.362 (1.33-1.395)	<0.0001	0.91	<0.0001	100.75
Study design	Prospective cohort study	10	1.362 (1.33-1.395)	<0.0001	0.91	<0.0001	100.75
	Retrospective cohort study	2	2.65 (2.224-3.158)	<0.0001	0	1	0
Study name	CHARLS	5	1.7 (1.562-1.851)	<0.0001	0.15	0.3161	4.73
	Others	7	1.354 (1.321-1.388)	<0.0001	0.95	<0.0001	124.93

Table S26. Subgroup analysis of the association between categorized TyG-WC index (highest vs. reference category) and risk of CVD incidence. TyG-WC, Triglyceride-Glucose-Waist Circumference. CVD, Cardiovascular Disease.

Category	Subgroup analysis	N	RR (95% CI)	p for RR	I^2	P for I^2	Q
Age	Age < 60	5	1.345 (1.256-1.441)	<0.0001	0.83	<0.0001	23.71
	Age > 60	8	1.334 (1.278-1.393)	<0.0001	0.85	<0.0001	46.04
Diabetes	Diabetes	3	1.372 (1.192-1.579)	<0.0001	0.72	0.0286	7.11
	No diabetes	2	1.206 (1.109-1.311)	<0.0001	0.38	0.2042	1.61
Diabetes	Prediabetes	2	1.475 (1.26-1.726)	<0.0001	0	0.3388	0.91
Ethnic	Chinese	7	1.365 (1.305-1.428)	<0.0001	0.91	<0.0001	65.7
	White	4	1.481 (1.428-1.536)	<0.0001	0.88	<0.0001	25.38
Follow up, years	>8.32	8	1.483 (1.442-1.526)	<0.0001	0.87	<0.0001	55.5
	≤8.32	4	1.267 (1.205-1.333)	<0.0001	0.8	0.0019	14.9
Male(%)	>50%	2	1.314 (1.18-1.463)	<0.0001	0	1	0
	≤50%	10	1.435 (1.399-1.472)	<0.0001	0.91	<0.0001	96.57
Study design	Prospective cohort study	10	1.435 (1.399-1.472)	<0.0001	0.91	<0.0001	96.57
	Retrospective cohort study	2	1.314 (1.18-1.463)	<0.0001	0	1	0
Study name	CHARLS	5	1.683 (1.561-1.815)	<0.0001	0.75	0.0033	15.81
	Others	7	1.4 (1.364-1.437)	<0.0001	0.9	<0.0001	62.77

Table S27. Subgroup analysis of the association between categorized TyG-WHtR index (highest vs. reference category) and risk of all-cause mortality. TyG-WHtR, Triglyceride-Glucose Waist-to-Height Ratio.

Category	Subgroup analysis	N	RR (95% CI)	p for RR	I^2	P for I^2	Q
Age	Age < 60	3	1.414 (1.25-1.6)	<0.0001	0.87	<0.0001	15.65
	Age > 60	4	1.424 (1.297-1.564)	<0.0001	0	0.9991	0.02
Ethnic	American	2	1.532 (1.196-1.962)	7e-04	0.72	0.0604	3.53
	White	3	1.219 (1.168-1.271)	<0.0001	0.81	0.0049	10.66
Male(%)	>50%	3	1.442 (1.305-1.593)	<0.0001	0.47	0.1517	3.77
	≤50%	3	1.197 (1.146-1.249)	<0.0001	0.29	0.2458	2.81
Study design	Prospective cohort study	3	1.197 (1.146-1.249)	<0.0001	0.29	0.2458	2.81
	Retrospective cohort study	2	1.42 (1.284-1.571)	<0.0001	0	1	0
Study name	NHANES	3	1.34 (1.193-1.505)	<0.0001	0.6	0.0833	4.97
	Others	3	1.219 (1.168-1.271)	<0.0001	0.81	0.0049	10.66

Table S28. Meta-regression of study-level characteristics on associations of TyG Index (per 1-SD increase) with all-cause mortality. TyG, Triglyceride-Glucose.

Variable	No	Estimate_aRR	Lower_Bound	Upper_Bound	P_Value	Adjusted_R^2
n	20	1	1	1	0.355	0
Follow up, years	20	1.1764	0.7618	1.8167	0.4636	7.92
Male(%)	20	0.9273	0.516	1.6664	0.8007	0
Age	20	1.2334	0.883	1.7227	0.2186	35.9

Table S29. Meta-regression of study-level characteristics on associations of TyG Index (per 1-SD increase) with CVD mortality. TyG, Triglyceride-Glucose. CVD, Cardiovascular Disease.

Variable	No	Estimate_aRR	Lower_Bound	Upper_Bound	P_Value	Adjusted_R^2
Follow up, years	12	1.4757	0.9127	2.386	0.1124	42.33
Male(%)	12	1.1284	0.8254	1.5425	0.4489	86.88
Age	12	1.0303	0.5527	1.9203	0.9253	28.08

Table S30. Meta-regression of study-level characteristics on associations of TyG Index (per 1-SD increase) with CVD incidence. TyG, Triglyceride-Glucose. CVD, Cardiovascular Disease.

Variable	No	Estimate_aRR	Lower_Bound	Upper_Bound	P_Value	Adjusted_R^2
n	25	0.8197	0.7634	0.8801	<0.0001	100
Follow up, years	25	1.1236	0.9338	1.3519	0.2169	100
Male(%)	25	1.1236	0.9338	1.3519	0.2169	100
Age	25	0.6146	0.4346	0.8693	0.0059	74.73

Table S31. Meta-regression of study-level characteristics on associations of TyG-BMI Index (per 1-SD increase) with CVD incidence. TyG-BMI, Triglyceride Glucose-Body Mass Index. CVD, Cardiovascular Disease.

Variable	No	Estimate_aRR	Lower_Bound	Upper_Bound	P_Value	Adjusted_R^2
n	10	0.9483	0.7203	1.2485	0.7051	0

Follow up, years	10	1.2747	1.0803	1.5041	0.004	99.96
Male(%)	10	1.2747	1.0804	1.504	0.004	100
Age	10	0.9109	0.7655	1.084	0.2931	51.72

Table S32. Meta-regression of study-level characteristics on associations of TyG-WC Index (per 1-SD increase) with CVD incidence. TyG-WC, Triglyceride-Glucose-Waist Circumference Index. CVD, Cardiovascular Disease.

**Variable**	**No**	**Estimate_aRR**	**Lower_Bound**	**Upper_Bound**	**P_Value**	**Adjusted_R²**
n	12	0.8532	0.5333	1.3652	0.5081	0
Follow up, years	12	1.1206	1.0484	1.1977	<0.0001	100
Male(%)	12	0.844	0.7807	0.9124	<0.0001	100
Age	12	0.9099	0.7146	1.1586	0.4439	87.78

Table S33. Meta-regression of study-level characteristics on associations of TyG-WHtR Index (per 1-SD increase) with CVD incidence. TyG-WHtR, Triglyceride-Glucose Waist-to-Height Ratio Index. CVD, Cardiovascular Disease.

**Variable**	**No**	**Estimate_aRR**	**Lower_Bound**	**Upper_Bound**	**P_Value**	**Adjusted_R²**
n	13	1.057	0.6277	1.7801	0.8348	0
Follow up, years	13	1.264	1.1874	1.3455	<0.0001	99.99
Male(%)	13	0.9416	0.873	1.0156	0.1188	100
Age	13	1.113	0.7566	1.6375	0.5867	64.65

Table S34. Meta-regression of study-level characteristics on associations of CTI Index (per 1-SD increase) with CVD incidence. CTI, C-Reactive Protein-Triglyceride Glucose Index. CVD, Cardiovascular Disease.

**Variable**	**No**	**Estimate_aRR**	**Lower_Bound**	**Upper_Bound**	**P_Value**	**Adjusted_R²**
n	11	1.0603	0.8793	1.2787	0.5397	0
Follow up, years	11	1.0603	0.9927	1.1326	0.0816	100
Male(%)	11	0.9837	0.8725	1.1092	0.7889	100
Age	11	1.0813	0.9549	1.2245	0.218	100

Table S35. Meta-regression of study-level characteristics on associations of TyG index (highest vs. reference category) with all-cause mortality. TyG, Triglyceride-Glucose.

**Variable**	**No**	**Estimate_aRR**	**Lower_Bound**	**Upper_Bound**	**P_Value**	**Adjusted_R²**
n	23	3.8427	0.8605	17.1612	0.0779	0
Follow up, years	23	0.824	0.404	1.6807	0.5945	0
Male(%)	23	1.2952	0.5359	3.1303	0.5656	0
Age	23	0.2876	0.0648	1.2759	0.1011	0

Table S36. Meta-regression of study-level characteristics on associations of TyG index (highest vs. reference category) with CVD mortality. TyG, Triglyceride-Glucose. CVD, Cardiovascular Disease.

**Variable**	**No**	**Estimate_aRR**	**Lower_Bound**	**Upper_Bound**	**P_Value**	**Adjusted_R²**
n	20	1.2422	0.498	3.0987	0.6419	28.19
Follow	20	0.6375	0.386	1.0527	0.0786	100

up, years						
Male(%)	20	1.0625	0.8864	1.2736	0.5121	100
Age	20	1.0063	0.3799	2.6658	0.9899	0

Table S37. Meta-regression of study-level characteristics on associations of TyG index (highest vs. reference category) with CVD incidence. TyG, Triglyceride-Glucose. CVD, Cardiovascular Disease.

Variable	No	Estimate_aRR	Lower_Bound	Upper_Bound	P_Value	Adjusted_R²
n	37	0.6436	0.4033	1.027	0.0646	46.08
Follow up, years	37	1	0.5433	1.8405	1	19.06
Male(%)	37	1.4796	0.8273	2.6463	0.1866	26.17
Age	37	0.4575	0.2656	0.7882	0.0048	20.95

Table S38. Meta-regression of study-level characteristics on associations of TyG index (highest vs. reference category) with stroke incidence. TyG, Triglyceride-Glucose.

Variable	No	Estimate_aRR	Lower_Bound	Upper_Bound	P_Value	Adjusted_R²
n	10	0.5442	0.2111	1.4027	0.2079	37.89
Follow up, years	10	0.8023	0.5194	1.2394	0.3209	37.89
Male(%)	10	1.7588	1.1034	2.8036	0.0176	37.89
Age	10	0.3856	0.1325	1.1228	0.0805	0

Table S39. Meta-regression of study-level characteristics on associations of TyG-BMI index (highest vs. reference category) with CVD incidence. TyG-BMI, Triglyceride Glucose-Body Mass Index. CVD, Cardiovascular Disease.

Variable	No	Estimate_aRR	Lower_Bound	Upper_Bound	P_Value	Adjusted_R²
n	12	1.4722	0.9042	2.397	0.1199	65.25
Follow up, years	12	1.1203	1.0575	1.1868	<0.0001	100
Male(%)	12	0.8391	0.7711	0.9131	<0.0001	100
Age	12	0.9183	0.7226	1.1669	0.4855	95.39

Table S40. Meta-regression of study-level characteristics on associations of TyG-WC index (highest vs. reference category) with CVD incidence. TyG-WC, Triglyceride-Glucose-Waist Circumference Index. CVD, Cardiovascular Disease.

Variable	No	Estimate_aRR	Lower_Bound	Upper_Bound	P_Value	Adjusted_R²
n	12	1.4722	0.9042	2.397	0.1199	65.25
Follow up, years	12	1.1203	1.0575	1.1868	<0.0001	100
Male(%)	12	0.8391	0.7711	0.9131	<0.0001	100
Age	12	0.9183	0.7226	1.1669	0.4855	95.39

Table S41. Meta-regression of study-level characteristics on associations of TyG-WHtR index (highest vs. reference category) with CVD incidence. TyG-WHtR, Triglyceride-Glucose Waist-to-Height Ratio Index. CVD, Cardiovascular Disease.

Variable	No	Estimate_aRR	Lower_Bound	Upper_Bound	P_Value	Adjusted_R²
n	12	1.4722	0.9042	2.397	0.1199	65.25
Follow	12	1.1203	1.0575	1.1868	<0.0001	100

up, years						
Male(%)	12	0.8391	0.7711	0.9131	<0.0001	100
Age	12	0.9183	0.7226	1.1669	0.4855	95.39

Table S42. Leave-one-out analysis for the association between TyG index (per 1-SD increase), (B) categorised TyG index (highest vs. reference category) and risks of all-cause mortality. TyG, Triglyceride-Glucose.

(A)TyG index (per 1-SD increase)

Excluded Studies	Pooled RR After Exclusion	Lower 95% CI	Upper 95% CI	I²(%)	Heterogeneity P-value	RR Change (%)
Chang Liu 2024	1.0326	1.0188	1.0465	86	0	-0.10%
Chang Liu 2025	1.0326	1.0189	1.0465	85.2	0	-0.09%
Jiahao Zou 2025	1.0318	1.018	1.0458	86.1	0	-0.17%
Tao Yi 2025	1.0286	1.0148	1.0426	81.4	0	-0.48%
Anthony Diwon 2026	1.0392	1.0251	1.0536	84.8	0	+0.55%
Claudia R. L. Cardoso 2026	1.0332	1.0194	1.0472	86.8	0	-0.04%
Haipeng Yao 2022	1.0328	1.0191	1.0467	85.6	0	-0.07%
Yanling Yang 2025	1.0338	1.0201	1.0478	86.7	0	+0.02%
Jiajun Liu 2025 (Normoglycemia)	1.0312	1.0174	1.0451	85.1	0	-0.23%
Jiajun Liu 2025 (Dysglycemia)	1.034	1.0202	1.048	86.8	0	+0.04%
Jiajun Liu 2025 (Diabetes)	1.0322	1.0184	1.0462	86.5	0	-0.13%
Younan Yao 2024	1.034	1.0201	1.048	86.8	0	+0.04%
Jun-Peng Xu 2024	1.0321	1.0182	1.0461	86.6	0	-0.15%
Kexin Wang 2025	1.0317	1.0179	1.0457	86.3	0	-0.18%
Peng Zhang 2025	1.0334	1.0197	1.0474	86.8	0	-0.01%
Matilde Sbriscia 2025	1.0328	1.019	1.0467	86.2	0	-0.08%
Kun Liu 2025	1.1062	1.0701	1.1435	84.1	0	**+7.02%**

(B) Categorised TyG index

Excluded Studies	Pooled RR After Exclusion	Lower 95% CI	Upper 95% CI	I²(%)	Heterogeneity P-value	RR Change (%)
Dan Zhou 2022	1.1020	1.0872	1.1171	0.83	0.0000	0.06%
Chang Liu 2024	1.1024	1.0875	1.1174	0.83	0.0000	0.03%
Jeongeun Kwak 2025	1.0692	1.0521	1.0865	0.74	0.0000	3.04%
Peng Zhang 2025	1.1026	1.0878	1.1177	0.84	0.0000	0.01%
Xiaoyuan Wei 2024	1.1005	1.0857	1.1156	0.83	0.0000	0.20%
Chang Liu 2025	1.1023	1.0874	1.1173	0.83	0.0000	0.04%
Jiahao Zou 2025	1.1023	1.0875	1.1174	0.84	0.0000	0.04%
Byung Sik Kim 2025	1.1547	1.1317	1.1781	0.77	0.0000	4.71%
Tao Yi 2025	1.1024	1.0875	1.1174	0.84	0.0000	0.03%
Anthony Diwon 2026	1.1052	1.0903	1.1204	0.82	0.0000	0.23%
Feng-Ching Shen 2023	1.1029	1.0880	1.1180	0.84	0.0000	0.02%
Chen Li 2024	1.1026	1.0878	1.1176	0.83	0.0000	0.01%
Haipeng Yao 2022	1.1025	1.0876	1.1175	0.83	0.0000	0.03%
Yanling Yang 2025	1.1033	1.0884	1.1184	0.83	0.0000	0.05%
Jiajun Liu 2025	1.1014	1.0866	1.1164	0.82	0.0000	0.12%
Jiajun Liu 2025	1.1032	1.0883	1.1183	0.84	0.0000	0.04%
Jiajun Liu 2025	1.1023	1.0874	1.1173	0.84	0.0000	0.04%
Younan Yao 2024	1.1035	1.0886	1.1186	0.83	0.0000	0.07%
Jun-Peng Xu 2024	1.1021	1.0872	1.1172	0.84	0.0000	0.06%
Kexin Wang 2025	1.1021	1.0872	1.1172	0.84	0.0000	0.06%

Qin Zhang 2023	1.1030	1.0881	1.1180	0.84	0.0000	0.02%
Mengjie Zhao 2023	1.1026	1.0877	1.1177	0.84	0.0000	0.01%
Matilde Sbriscia 2025	1.1020	1.0872	1.1171	0.83	0.0000	0.06%
Kun Liu 2025	1.1087	1.0930	1.1246	0.83	0.0000	0.54%

Table S43. Leave-one-out analysis for the association between TyG index (per 1-SD increase), (B) categorised TyG index (highest vs. reference category) and risks of CVD mortality. TyG, Triglyceride-Glucose. CVD, Cardiovascular Disease.

(A)TyG index (per 1-SD increase)

Excluded Studies	Pooled RR After Exclusion	Lower 95% CI	Upper 95% CI	I²(%)	Heterogeneity P-value	RR Change (%)
Chang Liu 2024	1.0553	1.0254	1.0861	78	0	-0.13%
Chang Liu 2025	1.0564	1.0265	1.0871	78.7	0	-0.03%
Jiahao Zou 2025	1.0533	1.0232	1.0842	77.9	0	-0.32%
Qiyu Xiao 2025	1.0526	1.0227	1.0833	74.7	0	-0.39%
Tao Yi 2025	1.0467	1.0167	1.0776	72	0	-0.94%
Anthony Diwon 2026	1.0764	1.0445	1.1092	71.6	0	+1.86%
Chuanwei Zhao 2026	1.0523	1.0219	1.0836	78.2	0	-0.41%
Claudia R. L. Cardoso 2026	1.0571	1.027	1.0881	78.8	0	+0.04%
Jiajun Liu 2025 (Normoglycemia)	1.0538	1.0238	1.0846	77.7	0	-0.27%
Jiajun Liu 2025 (Dysglycemia)	1.0575	1.0275	1.0885	78.7	0	+0.08%
Jiajun Liu 2025 (Diabetes)	1.0549	1.0249	1.0859	78.6	0	-0.16%
Jun-Peng Xu 2024	1.0533	1.0231	1.0844	78.3	0	-0.32%
Kexin Wang 2025	1.0534	1.0232	1.0845	78.3	0	-0.31%
Peng Zhang 2025	1.055	1.0251	1.0857	77.7	0	-0.16%
Kun Liu 2025	1.1235	1.0656	1.1846	76.2	0	**+6.33%**
Xiandu Jin 2026	1.0542	1.0243	1.085	77.6	0	-0.23%

(B) Categorised TyG index

Excluded Studies	Pooled RR After Exclusion	Lower 95% CI	Upper 95% CI	I²(%)	Heterogeneity P-value	RR Change (%)
Dan Zhou 2022	1.0986	1.0311	1.1706	0.58	0.0010	0.12%
Chang Liu 2024	1.0952	1.0279	1.1669	0.56	0.0017	0.44%
Xiandu Jin 2026	1.0977	1.0304	1.1694	0.57	0.0012	0.21%
Peng Zhang 2025	1.0952	1.0281	1.1666	0.49	0.0083	0.44%
Xiaoyuan Wei 2024	1.0897	1.0212	1.1628	0.56	0.0015	0.94%
Chang Liu 2025	1.0998	1.0325	1.1715	0.58	0.0009	0.02%
Jiahao Zou 2025	1.0926	1.0250	1.1646	0.56	0.0017	0.67%
Tao Yi 2025	1.0931	1.0257	1.1649	0.55	0.0019	0.63%
Anthony Diwon 2026	1.1305	1.0584	1.2074	0.48	0.0111	2.77%
Chuanwei Zhao 2026	1.1005	1.0315	1.1742	0.58	0.0009	0.05%
Xiaoling Chen 2025	1.0858	1.0183	1.1577	0.52	0.0045	1.29%
Jiajun Liu 2025 (Normoglycemia)	1.0924	1.0253	1.1640	0.53	0.0032	0.69%
Jiajun Liu 2025 (Dysglycemia)	1.1014	1.0334	1.1738	0.58	0.0010	0.13%
Jiajun Liu 2025 (Diabetes)	1.0965	1.0286	1.1688	0.57	0.0011	0.32%
Jun-Peng Xu 2024	1.0821	1.0139	1.1548	0.53	0.0035	1.63%
Kexin Wang 2025	1.0933	1.0255	1.1656	0.56	0.0014	0.61%
Qin Zhang 2023	1.0980	1.0305	1.1700	0.57	0.0010	0.18%
Mengjie Zhao 2023	1.1016	1.0334	1.1744	0.58	0.0010	0.15%
Yun Kyung Cho 2022	1.1353	1.0585	1.2177	0.53	0.0035	3.21%
Kun Liu 2025	1.1435	1.0581	1.2359	0.55	0.0023	3.96%

Table S44. Leave-one-out analysis for the association between TyG index (per 1-SD increase), (B) categorised TyG index (highest vs. reference category) and risks of CVD incidence. TyG, Triglyceride-Glucose. CVD，Cardiovascular Disease.

(A)TyG index (per 1-SD increase)

Excluded Studies	Pooled RR After Exclusion	Lower 95% CI	Upper 95% CI	I²(%)	Heterogeneity P-value	RR Change (%)
YaJie Zhao 2025	1.1024	1.0946	1.1104	91.7	0	+0.08%
Li 2025 (Intermediate coronary stenosis)	1.1013	1.0935	1.1092	91.6	0	-0.03%
Li 2025 (No Diabetes)	1.1015	1.0937	1.1094	91.7	0	-0.01%
Li 2025 (Diabetes)	1.1015	1.0937	1.1093	91.6	0	-0.01%
Qiyu Xiao 2025 (Heart failure)	1.1015	1.0937	1.1093	91.7	0	-0.01%
Qiyu Xiao 2025 (Myocardial infarction)	1.1015	1.0937	1.1093	91.7	0	-0.01%
Qiyu Xiao 2025 (Stroke)	1.1014	1.0936	1.1093	91.6	0	-0.02%
Qiyu Xiao 2025 (Peripheral arterial disease)	1.1013	1.0935	1.1092	91.5	0	-0.02%
Man Li 2025	1.101	1.0931	1.109	91.7	0	-0.05%
Yu Tang 2026	1.1015	1.0937	1.1094	91.7	0	-0.01%
Yilin Pan 2025	1.1022	1.0943	1.1101	91.7	0	+0.05%
Wei-Yu Su 2019	1.1012	1.0934	1.1091	91.6	0	-0.04%
Jianan Hong 2025	1.1015	1.0936	1.1094	91.7	0	-0.01%
Qingsong Mao 2025	1.1015	1.0936	1.1094	91.7	0	-0.01%
Yulong Lan 2025	1.1007	1.0928	1.1086	91.7	0	-0.09%
Shi Tai 2022	1.1004	1.0926	1.1083	91.6	0	-0.11%
Xin Chen 2025	1.1009	1.0931	1.1088	91.6	0	-0.06%
Jinling Hu 2025	1.1334	1.1243	1.1426	81.4	0	+2.89%
Chen Li 2024 (MACE)	1.1015	1.0937	1.1093	91.6	0	-0.01%
Chen Li 2024 (CAD events)	1.1016	1.0938	1.1095	91.7	0	-0.00%
Chen Li 2024 (Stroke)	1.1015	1.0937	1.1094	91.7	0	-0.01%
Claudia R. L. Cardoso 2026	1.102	1.0941	1.1098	91.6	0	+0.03%
Tonglong Jin 2026	1.1014	1.0936	1.1093	91.7	0	-0.02%
Jianyong Zhao 2025	1.1013	1.0935	1.1092	91.6	0	-0.03%
Xi Chen 2025	1.1015	1.0936	1.1093	91.7	0	-0.01%
Kun Liu 2025	1.0946	1.0854	1.1038	91.6	0	-0.64%
Weida Qiu 2024	1.1005	1.0927	1.1084	91.6	0	-0.10%
Chen 2025	1.1004	1.0926	1.1083	91.3	0	-0.11%
Keyu Bian 2025	1.1013	1.0934	1.1093	91.7	0	-0.02%
Hui Li 2026	1.0859	1.0768	1.0952	90.8	0	-1.42%
Matilde Sbriscia 2025	1.1015	1.0937	1.1094	91.7	0	-0.01%
Peng Ning 2024	1.1008	1.093	1.1087	89.8	0	-0.07%
Yumeng Shi 2024	1.1018	1.0939	1.1096	91.7	0	+0.01%

(B) Categorised TyG index

Excluded Studies	Pooled RR After Exclusion	Lower 95% CI	Upper 95% CI	I²(%)	Heterogeneity P-value	RR Change (%)
YaJie Zhao 2025	1.1928	1.1802	1.2055	0.94	0.0000	0.02%
Jeongeun Kwak 2025	1.1717	1.1579	1.1857	0.94	0.0000	1.78%
Li 2025	1.1922	1.1797	1.2049	0.94	0.0000	0.06%
Chen 2025	1.1920	1.1794	1.2046	0.94	0.0000	0.08%
Keyu Bian 2025	1.1952	1.1825	1.2081	0.94	0.0000	0.19%
Byung Sik Kim 2025 (Heart failure)	1.2392	1.2243	1.2542	0.92	0.0000	3.87%
Byung Sik Kim 2025 (Stroke)	1.1894	1.1756	1.2034	0.94	0.0000	0.30%
Byung Sik Kim 2025 (Myocardial infarction)	1.1741	1.1615	1.1869	0.91	0.0000	1.58%
Qiyu Xiao 2025 (Heart failure)	1.1928	1.1802	1.2054	0.94	0.0000	0.02%
Qiyu Xiao 2025 (Myocardial infarction)	1.1928	1.1803	1.2055	0.94	0.0000	0.01%
Qiyu Xiao 2025 (Stroke)	1.1927	1.1802	1.2054	0.94	0.0000	0.02%
Qiyu Xiao 2025 (Peripheral arterial disease (PAD))	1.1926	1.1801	1.2053	0.94	0.0000	0.03%
Man Li 2025	1.1912	1.1786	1.2039	0.94	0.0000	0.15%
Menghe Wang 2025	1.1948	1.1820	1.2078	0.94	0.0000	0.15%
Yilin Pan 2025	1.1932	1.1806	1.2059	0.94	0.0000	0.02%
Jianan Hong 2025	1.1926	1.1800	1.2053	0.94	0.0000	0.03%
Qingsong Mao 2025	1.1926	1.1800	1.2053	0.94	0.0000	0.03%
Yulong Lan 2025	1.1919	1.1794	1.2046	0.94	0.0000	0.09%
Shi Tai 2022 (MACEs)	1.1949	1.1821	1.2078	0.94	0.0000	0.16%
Shi Tai 2022 (Major coronary events)	1.1937	1.1810	1.2066	0.94	0.0000	0.06%
Xin Chen 2025	1.1926	1.1800	1.2053	0.94	0.0000	0.03%
Jinling Hu 2025	1.2099	1.1967	1.2232	0.93	0.0000	1.42%
Xiaoling Chen 2025	1.1926	1.1801	1.2053	0.94	0.0000	0.03%
Chen Li 2024 (MACE)	1.1926	1.1800	1.2053	0.94	0.0000	0.03%
Chen Li 2024 (CAD events)	1.1930	1.1804	1.2056	0.94	0.0000	0.00%
Chen Li 2024 (Stroke)	1.1928	1.1802	1.2054	0.94	0.0000	0.02%
Tonglong Jin 2026	1.1925	1.1800	1.2052	0.94	0.0000	0.04%
Jianyong Zhao 2025	1.1928	1.1802	1.2054	0.94	0.0000	0.02%
Xi Chen 2025	1.1927	1.1802	1.2054	0.94	0.0000	0.02%
Xingyu Zhou 2025 (CKM stage 0)	1.1930	1.1804	1.2056	0.94	0.0000	0.00%
Xingyu Zhou 2025 (CKM stage 1)	1.1931	1.1806	1.2058	0.94	0.0000	0.01%
Zhengwen Xu 2025	1.1921	1.1796	1.2048	0.94	0.0000	0.07%
Fomin Zhang 2023	1.1928	1.1802	1.2055	0.94	0.0000	0.02%
Yun Kyung Cho 2022	1.1911	1.1784	1.2039	0.94	0.0000	0.16%
Kun Liu 2025	1.1846	1.1717	1.1977	0.94	0.0000	0.70%
Nan Zhang 2023	1.1928	1.1802	1.2054	0.94	0.0000	0.02%

Table S45. Leave-one-out analysis for the association between TyG index (per 1-SD increase), (B) categorised TyG index (highest vs. reference category) and risks of CHD incidence. TyG, Triglyceride-Glucose. CHD, Coronary Heart Disease

(A)TyG index (per 1-SD increase)

Excluded Studies	Pooled RR After Exclusion	Lower 95% CI	Upper 95% CI	I^2(%)	Heterogeneity P-value	RR Change (%)
Qiyu Xiao 2025	1.1271	1.117	1.1373	92	0	-0.02%
Man Li 2025	1.1252	1.115	1.1354	90.9	0	-0.19%
Shi Tai 2022	1.1258	1.1156	1.136	91.4	0	-0.13%
Jinling Hu 2025	1.1478	1.1361	1.1595	52.4	0.0619	+1.82%
Chen Li 2024	1.1273	1.1172	1.1374	92.1	0	-0.00%
Kun Liu 2025	1.1191	1.1075	1.1309	91.1	0	-0.72%
Hui Li 2026	1.1161	1.1024	1.13	91.4	0	-0.99%

(B) Categorised TyG index

Excluded Studies	Pooled RR After Exclusion	Lower 95% CI	Upper 95% CI	I^2(%)	Heterogeneity P-value	RR Change (%)
Jeongeun Kwak 2025	1.3820	1.3472	1.4176	0.96	0.0000	4.08%
Byung Sik Kim 2025	1.2615	1.2330	1.2907	0.89	0.0000	4.99%
Qiyu Xiao 2025	1.3276	1.3000	1.3558	0.97	0.0000	0.01%
Man Li 2025	1.3226	1.2950	1.3508	0.96	0.0000	0.39%
Shi Tai 2022	1.3515	1.3216	1.3821	0.96	0.0000	1.79%
Jinling Hu 2025	1.3543	1.3240	1.3854	0.96	0.0000	2.00%
Chen Li 2024	1.3278	1.3003	1.3560	0.97	0.0000	0.00%
Kun Liu 2025	1.3058	1.2748	1.3376	0.96	0.0000	1.65%

Table S46. Leave-one-out analysis for the association between TyG index (per 1-SD increase), (B) categorised TyG index (highest vs. reference category) and risks of stroke incidence. TyG, Triglyceride-Glucose.

(A)TyG index (per 1-SD increase)

Excluded Studies	Pooled RR After Exclusion	Lower 95% CI	Upper 95% CI	I²(%)	Heterogeneity P-value	RR Change (%)
Qiyu Xiao 2025	1.0447	1.0269	1.0628	79.4	0.0007	-0.12%
Man Li 2025	1.0407	1.0216	1.0601	83.3	0.0001	-0.51%
Jinling Hu 2025	1.0522	1.0323	1.0725	83.4	0.0001	+0.60%
Chen Li 2024	1.0454	1.0277	1.0635	82.3	0.0002	-0.05%
Keyu Bian 2025	1.0321	1.0127	1.0519	74.6	0.0034	-1.32%
Kun Liu 2025	1.071	1.0457	1.0969	78.2	0.0011	+2.39%

(B) Categorised TyG index

Excluded Studies	Pooled RR After Exclusion	Lower 95% CI	Upper 95% CI	I²(%)	Heterogeneity P-value	RR Change (%)
Jeongeun Kwak 2025	1.1747	1.1499	1.2000	0.86	0.0000	3.70%
Keyu Bian 2025	1.2291	1.2063	1.2524	0.91	0.0000	0.77%
Byung Sik Kim 2025	1.2319	1.2000	1.2647	0.92	0.0000	1.00%
Qiyu Xiao 2025	1.2190	1.1972	1.2412	0.92	0.0000	0.06%
Man Li 2025	1.2191	1.1972	1.2415	0.92	0.0000	0.05%
Jinling Hu 2025	1.2286	1.2063	1.2513	0.90	0.0000	0.72%
Chen Li 2024	1.2192	1.1974	1.2414	0.91	0.0000	0.04%
Kun Liu 2025	1.2344	1.2116	1.2576	0.89	0.0000	1.20%
Nan Zhang 2023 (First total stroke)	1.2193	1.1974	1.2415	0.92	0.0000	0.04%
Nan Zhang 2023 (First ischemic stroke)	1.2185	1.1967	1.2407	0.91	0.0000	0.10%

Table S47. Leave-one-out analysis for the association between TyG index (per 1-SD increase), (B) categorised TyG index (highest vs. reference category) and risks of heart failure incidence. TyG, Triglyceride-Glucose.

(A)TyG index (per 1-SD increase)

Excluded Studies	Pooled RR After Exclusion	Lower 95% CI	Upper 95% CI	I^2(%)	Heterogeneity P-value	RR Change (%)
Qiyu Xiao 2025	1.346	1.2473	1.4526	92.4	0.0003	-0.35%
Yumeng Shi 2024	1.4485	1.3333	1.5738	0	0.932	**+7.24%**
Mao-Jun Liu 2025	1.1073	0.9587	1.2791	70.8	0.0644	**-18.02%**

(B) Categorised TyG index

Excluded Studies	Pooled RR After Exclusion	Lower 95% CI	Upper 95% CI	I^2(%)	Heterogeneity P-value	RR Change (%)
Byung Sik Kim 2025	1.4044	1.1819	1.6689	0.78	0.0113	32.65%
Qiyu Xiao 2025	1.0577	1.0351	1.0809	0.87	0.0004	0.10%
Zhengwen Xu 2025	1.0546	1.0320	1.0778	0.48	0.1464	0.39%
Yumeng Shi 2024	1.0592	1.0365	1.0824	0.90	0.0001	0.04%

Table S48. Leave-one-out analysis for the association between TyG index (per 1-SD increase), (B) categorised TyG index (highest vs. reference category) and risks of MACE incidence. TyG, Triglyceride-Glucose. MACE, Major Adverse Cardiovascular Events.

(A)TyG index (per 1-SD increase)

Excluded Studies	Pooled RR After Exclusion	Lower 95% CI	Upper 95% CI	I²(%)	Heterogeneity P-value	RR Change (%)
Shi Tai 2022	1.1576	1.0111	1.3252	84.5	0.0016	-2.14%
Claudia R. L. Cardoso 2026	1.2204	1.142	1.3042	55	0.1085	+3.17%
Jianyong Zhao 2025	1.1627	1.0896	1.2407	76	0.0155	-1.70%
Xi Chen 2025	1.1738	1.0999	1.2527	83.5	0.0023	-0.77%

(B) Categorised TyG index

Excluded Studies	Pooled RR After Exclusion	Lower 95% CI	Upper 95% CI	I²(%)	Heterogeneity P-value	RR Change (%)
Li 2025	1.1644	1.0972	1.2357	0.81	0.0003	1.94%
Shi Tai 2022	1.7133	1.4474	2.0279	0.61	0.0359	44.28%
Chen Li 2024	1.1761	1.1093	1.2470	0.79	0.0007	0.96%
Jianyong Zhao 2025	1.1814	1.1144	1.2525	0.85	0.0000	0.51%
Xi Chen 2025	1.1810	1.1138	1.2523	0.86	0.0000	0.54%
Fomin Zhang 2023	1.1813	1.1129	1.2540	0.87	0.0000	0.52%

Table S49. Leave-one-out analysis for the association between TyG-BMI index (per 1-SD increase), (B) categorised TyG index (highest vs. reference category) and risks of all-cause mortality. TyG-BMI, Triglyceride Glucose-Body Mass Index.

(A)TyG-BMI index (per 1-SD increase)

Excluded Studies	Pooled RR After Exclusion	Lower 95% CI	Upper 95% CI	I²(%)	Heterogeneity P-value	RR Change (%)
Claudia R. L. Cardoso 2026	1.0282	1.0201	1.0365	84.87%	0	+0.02
Yanling Yang 2025	1.0786	1.0645	1.0928	46.85%	0	+4.91
Lei Ding 2024	1.0290	1.0208	1.0373	84.20%	0	+0.09
Peng Zhang 2025	1.0278	1.0196	1.0360	84.72%	0	-0.03
Mao-Jun Liu 2025	1.0257	1.0174	1.0340	85.28%	0	-0.23

(B) Categorised TyG-BMI index

Excluded Studies	Pooled RR After Exclusion	Lower 95% CI	Upper 95% CI	I²(%)	Heterogeneity P-value	RR Change (%)
Peng Zhang 2025	1.1274	1.0803	1.1767	84.33	0.000255	-0.42%
Yanling Yang 2025	1.1332	1.0858	1.1827	88.09	0.000014	+0.09%
Lei Ding 2024	1.1385	1.0908	1.1882	82.29	0.000727	+0.55%
Mao-Jun Liu 2025	1.1078	1.0594	1.1583	80.16	0.001717	-2.16%
Kun Liu 2025	1.3382	1.1739	1.5254	83.84	0.000337	+18.19%

Table S50. Leave-one-out analysis for the association between TyG-BMI index (per 1-SD increase), (B) categorised TyG index (highest vs. reference category) and risks of CVD mortality. TyG-BMI, Triglyceride Glucose-Body Mass Index. CVD, Cardiovascular Disease

(A)TyG-BMI index (per 1-SD increase)

Excluded Studies	Pooled RR After Exclusion	Lower 95% CI	Upper 95% CI	I²(%)	Heterogeneity P-value	RR Change (%)
Claudia R. L. Cardoso 2026	1.1679	1.1325	1.2044	84.87 %	0	+0.30
Lei Ding 2024	1.1839	1.1474	1.2215	46.85 %	0	+1.67
Xiandu Jin 2026	1.1636	1.1287	1.1996	84.20 %	0	-0.07
Peng Zhang 2025	1.1621	1.1271	1.1982	84.72 %	0	-0.19
Mao-Jun Liu 2025	1.1574	1.1206	1.1955	85.28 %	0	-0.60
Kun Liu 2025	1.1011	1.0273	1.1803	84.16 %	0	-5.43

(B) Categorised TyG-BMI index

Excluded Studies	Pooled RR After Exclusion	Lower 95% CI	Upper 95% CI	I²(%)	Heterogeneity P-value	RR Change (%)
Xiandu Jin 2026	1.4489	1.3102	1.6023	83.76	0.000351	-0.78%
Peng Zhang 2025	1.4463	1.3084	1.5987	82.02	0.000820	-0.96%
Lei Ding 2024	1.4966	1.3536	1.6548	50.40	0.109291	+2.49%
Mao-Jun Liu 2025	1.4105	1.2648	1.5730	82.65	0.000615	-3.41%
Kun Liu 2025	1.6183	1.3048	2.0070	83.81	0.000341	+10.82 %

Table S51. Leave-one-out analysis for the association between TyG-BMI index (per 1-SD increase), (B) categorised TyG index (highest vs. reference category) and risks of CVD incidence. TyG-BMI, Triglyceride Glucose-Body Mass Index. CVD, Cardiovascular Disease

(A)TyG-BMI index (per 1-SD increase)

Excluded Studies	Pooled RR After Exclusion	Lower 95% CI	Upper 95% CI	I²(%)	Heterogeneity P-value	RR Change (%)
YaJie Zhao 2025	1.0024	1.0019	1.0029	99.14 %	0	-0.00
Weipeng Li 2024	1.0024	1.0019	1.0029	99.12 %	0	-0.00
Yilin Pan 2025	1.0024	1.0019	1.0029	99.15 %	0	-0.00
Jianan Hong 2025	1.0024	1.0019	1.0029	99.10 %	0	-0.00
Qingsong Mao 2025	1.0024	1.0019	1.0029	99.10 %	0	-0.00
Jinling Hu 2025	1.0023	1.0018	1.0028	98.79 %	0	-0.01
Claudia R. L. Cardoso 2026	1.0024	1.0019	1.0029	99.15 %	0	+0.00
Tonglong Jin 2026	1.0024	1.0019	1.0029	99.12 %	0	-0.00
Kun Liu 2025	1.0022	1.0017	1.0027	98.20 %	0	-0.02
Keyu Bian 2025	1.1390	1.1292	1.1490	92.39 %	0	**+13.63**

(B) Categorised TyG-BMI index

Excluded Studies	Pooled RR After Exclusion	Lower 95% CI	Upper 95% CI	I²(%)	Heterogeneity P-value	RR Change (%)
YaJie Zhao 2025	1.3725	1.3400	1.4058	92.92	0.000000	+0.18%
Weipeng Li 2024	1.3664	1.3343	1.3993	92.70	0.000000	-0.26%
Keyu Bian 2025	1.3999	1.3651	1.4355	91.28	0.000000	+2.18%
Menghe Wang 2025	1.4026	1.3680	1.4381	90.54	0.000000	+2.38%
Yilin Pan 2025	1.3679	1.3356	1.4009	92.92	0.000000	-0.16%
Jianan Hong 2025	1.3632	1.3310	1.3963	92.55	0.000000	-0.49%
Qingsong Mao 2025	1.3632	1.3310	1.3963	92.55	0.000000	-0.49%
Jinling Hu 2025	1.3902	1.3505	1.4310	92.81	0.000000	+1.47%
Tonglong Jin 2026	1.3659	1.3338	1.3988	92.42	0.000000	-0.30%
Mao-Jun Liu 2025	1.3617	1.3297	1.3945	91.07	0.000000	-0.61%
Kun Liu 2025	1.3106	1.2727	1.3495	91.23	0.000000	-4.34%

Table S52. Leave-one-out analysis for the association between TyG-BMI index (per 1-SD increase) and risks of CHD incidence. TyG-BMI, Triglyceride Glucose-Body Mass Index. CVD, Cardiovascular Disease. CHD, Coronary Heart Disease.

Excluded Studies	Pooled RR After Exclusion	Lower 95% CI	Upper 95% CI	I^2(%)	Heterogeneity P-value	RR Change (%)
Lian Wang 2025	1.1739	1.1611	1.1868	98.10 %	0	+6.60
Kun Liu 2025	1.0505	1.0409	1.0602	91.65 %	0	-4.60
Jinling Hu 2025	1.1025	1.0939	1.1112	99.66 %	0	+0.12

Table S53. Leave-one-out analysis for the association between TyG-BMI index (per 1-SD increase), (B) categorised TyG index (highest vs. reference category) and risks of stroke incidence. TyG-BMI, Triglyceride Glucose-Body Mass Index. CVD, Cardiovascular Disease. CHD, Coronary Heart Disease.

(A)TyG-BMI index (per 1-SD increase)

Excluded Studies	Pooled RR After Exclusion	Lower 95% CI	Upper 95% CI	I²(%)	Heterogeneity P-value	RR Change (%)
Jinling Hu 2025	1.0020	1.0015	1.0025	62.74 %	0.0638	-0.00
Keyu Bian 2025	1.0300	1.0063	1.0543	70.90 %	1	+2.79
Kun Liu 2025	1.0020	1.0015	1.0025	0.00%	0.1645	-0.00

(B) Categorised TyG-BMI index

Excluded Studies	Pooled RR After Exclusion	Lower 95% CI	Upper 95% CI	I²(%)	Heterogeneity P-value	RR Change (%)
Keyu Bian 2025	1.0335	0.9712	1.0998	0.00	0.884508	-4.83%
Jinling Hu 2025	1.0974	1.0418	1.1560	79.02	0.029031	+1.06%
Kun Liu 2025	1.1194	1.0558	1.1869	63.33	0.098684	+3.09%

Table S54. Leave-one-out analysis for the association between TyG-WC index (per 1-SD increase), (B) categorised TyG-WC index (highest vs. reference category) and risks of CVD mortality. TyG-WC, Triglyceride Glucose-Waist Circumference Index. CVD, Cardiovascular Disease.

(A)TyG-WC index (per 1-SD increase)

Excluded Studies	Pooled RR After Exclusion	Lower 95% CI	Upper 95% CI	I²(%)	Heterogeneity P-value	RR Change (%)
Xiandu Jin 2026	1.2337	1.1897	1.2794	71.25 %	0.0622	-0.25
Peng Zhang 2025	1.2331	1.1891	1.2788	77.71 %	0.0342	-0.30
Kun Liu 2025	1.9737	1.4117	2.7594	0.00%	0.5476	**+59.58**

(B) Categorised TyG-WC index

Excluded Studies	Pooled RR After Exclusion	Lower 95% CI	Upper 95% CI	I²(%)	Heterogeneity P-value	RR Change (%)
Xiandu Jin 2026	1.5008	1.3397	1.6812	0.00	0.854416	-1.04%
Peng Zhang 2025	1.5128	1.3510	1.6940	9.69	0.330468	-0.24%
Xiaoyuan Wei 2024	1.5345	1.3520	1.7417	11.94	0.321215	+1.19%
Kun Liu 2025	1.5381	1.2175	1.9431	17.14	0.299158	+1.42%

Table S55. Leave-one-out analysis for the association between TyG-WC index (per 1-SD increase), (B) categorised TyG-WC index (highest vs. reference category) and risks of CVD incidence. TyG-WC, Triglyceride Glucose-Waist Circumference Index. CVD, Cardiovascular Disease.

(A)TyG-WC index (per 1-SD increase)

Excluded Studies	Pooled RR After Exclusion	Lower 95% CI	Upper 95% CI	I²(%)	Heterogeneity P-value	RR Change (%)
YaJie Zhao 2025	1.0897	1.0822	1.0972	97.80 %	0	-0.08
Yuyu Cui 2025	1.1535	1.1424	1.1646	87.97 %	0	+5.77
Yilin Pan 2025	1.0898	1.0824	1.0973	97.80 %	0	-0.07
Jianan Hong 2025	1.0884	1.0809	1.0959	97.68 %	0	-0.20
Qingsong Mao 2025	1.0884	1.0809	1.0959	97.68 %	0	-0.20
Jinling Hu 2025	1.0805	1.0720	1.0891	97.68 %	0	-0.92
Tonglong Jin 2026	1.0892	1.0818	1.0967	97.65 %	0	-0.12
Keyu Bian 2025	1.0906	1.0831	1.0980	97.51 %	0	+0.00
Mao-Jun Liu 2025	1.0902	1.0827	1.0977	97.82 %	0	-0.04
Kun Liu 2025	1.0719	1.0639	1.0799	96.46 %	0	-1.71

(B) Categorised TyG-WC index

Excluded Studies	Pooled RR After Exclusion	Lower 95% CI	Upper 95% CI	I²(%)	Heterogeneity P-value	RR Change (%)
YaJie Zhao 2025	1.4319	1.3961	1.4685	90.80	0.000000	+0.03%
Yuyu Cui 2025	1.4279	1.3921	1.4646	90.70	0.000000	-0.24%
Keyu Bian 2025	1.4485	1.4109	1.4870	89.98	0.000000	+1.19%
Menghe Wang 2025	1.4878	1.4475	1.5292	83.29	0.000000	+3.94%
Yilin Pan 2025	1.4316	1.3959	1.4682	90.80	0.000000	+0.02%
Jianan Hong 2025	1.4229	1.3873	1.4593	89.96	0.000000	-0.59%
Qingsong Mao 2025	1.4229	1.3873	1.4593	89.96	0.000000	-0.59%
Jinling Hu 2025	1.4387	1.3977	1.4808	90.76	0.000000	+0.51%
Tonglong Jin 2026	1.4256	1.3904	1.4618	88.92	0.000000	-0.40%
Mao-Jun Liu 2025	1.4348	1.3989	1.4716	90.68	0.000000	+0.24%
Kun Liu 2025	1.3671	1.3264	1.4091	87.12	0.000000	-4.49%

Table S56. Leave-one-out analysis for the association between TyG-WC index (per 1-SD increase), (B) categorised TyG-WC index (highest vs. reference category) and risks of stroke incidence. TyG-WC, Triglyceride Glucose-Waist Circumference Index.

(A)TyG-WC index (per 1-SD increase)

Excluded Studies	Pooled RR After Exclusion	Lower 95% CI	Upper 95% CI	I^2(%)	Heterogeneity P-value	RR Change (%)
Keyu Bian 2025	1.0531	1.0284	1.0784	0	0	-0.08
Kun Liu 2025	1.0600	1.0157	1.1062	0	0	+5.77
Jinling Hu 2025	1.0500	1.0204	1.0804	0	0	-0.07

(B) Categorised TyG-WC index

Excluded Studies	Pooled RR After Exclusion	Lower 95% CI	Upper 95% CI	I^2(%)	Heterogeneity P-value	RR Change (%)
Keyu Bian 2025	1.0335	0.9712	1.0998	0.00	0.884508	-4.83%
Jinling Hu 2025	1.0974	1.0418	1.1560	79.02	0.029031	+1.06%
Kun Liu 2025	1.1194	1.0558	1.1869	63.33	0.098684	+3.09%

Table S57. Leave-one-out analysis for the association between TyG-WHtR index (per 1-SD increase), (B) categorised TyG-WHtR index (highest vs. reference category) and risks of all-cause mortality. TyG-WHtR, Triglyceride–Glucose Waist-to-Height Ratio Index.

(A)TyG-WHtR index (per 1-SD increase)

Excluded Studies	Pooled RR After Exclusion	Lower 95% CI	Upper 95% CI	I^2(%)	Heterogeneity P-value	RR Change (%)
Peng Zhang 2025	1.1145	1.1003	1.1290	55.88 %	0.1038	-0.07
Mao-Jun Liu 2025	1.1132	1.0986	1.1280	73.59 %	0.0227	-0.19
Kun Liu 2025	1.1696	1.1212	1.2202	43.91 %	0.1681	+4.87
Xiaoran Shen 2025	1.1132	1.0988	1.1278	66.71 %	0.0496	-0.19

(B) Categorised TyG-WHtR index

Excluded Studies	Pooled RR After Exclusion	Lower 95% CI	Upper 95% CI	I^2(%)	Heterogeneity P-value	RR Change (%)
Peng Zhang 2025	1.2138	1.1646	1.2651	61.86 %	0.0489	-32.46%
Xiaoyuan Wei 2024	1.2101	1.1586	1.2639	76.86 %	0.0047	-62.77%
Xiaoran Shen 2025	1.2139	1.1642	1.2657	76.60 %	0.005	-32.22%
Mao-Jun Liu 2025	1.2008	1.1502	1.2538	66.41 %	0.0302	-139.09 %
Kun Liu 2025	1.3712	1.253	1.5004	43.97 %	0.1476	1259.46 %

Table S58. Leave-one-out analysis for the association between TyG-WHtR index (per 1-SD increase), (B) categorised TyG-WHtR index (highest vs. reference category) and risks of CVD mortality. TyG-WHtR, Triglyceride–Glucose Waist-to-Height Ratio Index. CVD, Cardiovascular Disease

(A)TyG-WHtR index (per 1-SD increase)

Excluded Studies	Pooled RR After Exclusion	Lower 95% CI	Upper 95% CI	I²(%)	Heterogeneity P-value	RR Change (%)
Xiandu Jin 2026	1.2064	1.1684	1.1877	62.05 %	0.0717	-0.26
Peng Zhang 2025	1.2068	1.1688	1.1817	80.80 %	0.0055	-0.23
Kun Liu 2025	1.2035	1.0657	1.1865	85.75 %	0.0009	-0.50
Xiaoran Shen 2025	1.2163	1.1769	1.1833	83.67 %	0.0022	+0.56

(B) Categorised TyG-WHtR index

Excluded Studies	Pooled RR After Exclusion	Lower 95% CI	Upper 95% CI	I²(%)	Heterogeneity P-value	RR Change (%)
Xiandu Jin 2026	1.4865	1.3386	1.6508	7.30%	0.3566	-70.06%
Peng Zhang 2025	1.4856	1.3381	1.6494	0.00%	0.6307	-76.09%
Xiaoyuan Wei 2024	1.4963	1.3339	1.6785	36.81 %	0.1912	-4.33%
Xiaoran Shen 2025	1.5074	1.3532	1.6792	33.52 %	0.2112	69.71%
Kun Liu 2025	1.5457	1.2613	1.8941	35.05 %	0.2019	325.28%

Table S59. Leave-one-out analysis for the association between TyG-WHtR index (per 1-SD increase), (B) categorised TyG-WHtR index (highest vs. reference category) and risks of CVD incidence. TyG-WHtR, Triglyceride–Glucose Waist-to-Height Ratio Index. CVD, Cardiovascular Disease

(A)TyG-WHtR index (per 1-SD increase)

Excluded Studies	Pooled RR After Exclusion	Lower 95% CI	Upper 95% CI	I²(%)	Heterogeneity P-value	RR Change (%)
YaJie Zhao 2025	1.1768	1.1659	1.1877	0.82	0	+0.31
Ke Wang 2026	1.1708	1.1599	1.1817	0.75	0	-0.20
Yilin Pan 2025	1.1756	1.1647	1.1865	0.82	0	+0.21
Jianan Hong 2025	1.1724	1.1615	1.1833	0.81	0	-0.07
Qingsong Mao 2025	1.1724	1.1615	1.1833	0.84	0	-0.07
Jinling Hu 2025	1.1764	1.1611	1.1919	0.84	0	+0.28
Tonglong Jin 2026	1.1716	1.1609	1.1824	0.84	0	-0.13
Cheng Huang 2025	1.1743	1.1636	1.1852	0.80	0	+0.10
Mao-Jun Liu 2025	1.1748	1.1640	1.1858	0.83	0	+0.14
Kun Liu 2025	1.1661	1.1535	1.1789	0.83	0	-0.60

(B) Categorised TyG-WHtR index

Excluded Studies	Pooled RR After Exclusion	Lower 95% CI	Upper 95% CI	I²(%)	Heterogeneity P-value	RR Change (%)
YaJie Zhao 2025	1.383	1.3481	1.4188	91.64 %	0	32.74%
Menghe Wang 2025	1.4235	1.3845	1.4636	89.21 %	0	326.80%
Ke Wang 2026	1.3676	1.3329	1.4032	90.93 %	0	-78.55%
Xiaoran Shen 2025	1.3785	1.344	1.414	91.88 %	0	0.54%
Yilin Pan 2025	1.3788	1.344	1.4146	91.88 %	0	2.67%
Jianan Hong 2025	1.3724	1.3376	1.408	91.55 %	0	-44.34%
Qingsong Mao 2025	1.3724	1.3376	1.408	91.55 %	0	-44.34%
Jinling Hu 2025	1.4477	1.4034	1.4933	89.41 %	0	502.00%
Tonglong Jin 2026	1.373	1.3385	1.4083	90.63 %	0	-39.75%
Cheng Huang 2025	1.3805	1.3456	1.4162	91.83 %	0	14.64%
Mao-Jun Liu 2025	1.3792	1.3442	1.4151	91.87 %	0	5.41%
Kun Liu 2025	1.2902	1.251	1.3307	85.52 %	0	-639.99 %

Table S60. Leave-one-out analysis for the association between TyG-WC index (per 1-SD increase), (B) categorised TyG-WC index (highest vs. reference category) and risks of all-cause mortality. TyG-WC, Triglyceride Glucose-Waist Circumference Index.

(A)TyG-WC index (per 1-SD increase)

Excluded Studies	Pooled RR After Exclusion	Lower 95% CI	Upper 95% CI	I²(%)	Heterogeneity P-value	RR Change (%)
Peng Zhang 2025	1.1335	1.1147	1.1527	16.99 %	0.2724	-0.13
Kun Liu 2025	1.1794	1.1198	1.2423	74.53 %	0.0475	+3.91
Mao-Jun Liu 2025	1.1317	1.1119	1.1518	80.68 %	0.0229	-0.30

(B) Categorised TyG-WC index

Excluded Studies	Pooled RR After Exclusion	Lower 95% CI	Upper 95% CI	I²(%)	Heterogeneity P-value	RR Change (%)
Peng Zhang 2025	1.2537	1.1973	1.3127	68.40	0.042244	-0.47%
Xiaoyuan Wei 2024	1.2725	1.2117	1.3363	82.94	0.002842	+1.03%
Mao-Jun Liu 2025	1.2368	1.1784	1.2981	74.16	0.020864	-1.81%
Kun Liu 2025	1.3276	1.2063	1.4612	82.76	0.003030	+5.41%

Table S61. Leave-one-out analysis for the association between CTI index (per 1-SD increase), (B) categorised CTI index (highest vs. reference category) and risks of CVD incidence. CTI, C-Reactive Protein-Triglyceride Glucose Index. CVD, Cardiovascular Disease

(A)CTI index (per 1-SD increase)

Excluded Studies	Pooled RR After Exclusion	Lower 95% CI	Upper 95% CI	I²(%)	Heterogeneity P-value	RR Change (%)
Zhihui Lu 2025	1.2233	1.2056	1.2412	56.2	0.0254	+0.13%
Jintao Chen 2025	1.2226	1.205	1.2404	58.2	0.019	+0.08%
Ze-Jiaxin Niu 2026	1.2202	1.2023	1.2383	58.2	0.0192	-0.12%
Yilin Pan 2025	1.2285	1.2105	1.2467	0	0.5058	+0.56%
Yinsong Xu 2025	1.2202	1.2027	1.238	54.6	0.0311	-0.12%
Songyuan Tang 2024	1.2218	1.2043	1.2396	59.4	0.016	+0.01%
Tonglong Jin 2026	1.2203	1.2027	1.2381	57	0.0226	-0.11%
Hui Li 2026	1.1928	1.157	1.2297	50.8	0.0475	-2.36%
Hao Li 2026	1.2231	1.2055	1.241	56.5	0.0243	+0.12%
Zhihui Lu 2025	1.2233	1.2056	1.2412	56.2	0.0254	+0.13%

(B) Categorised CTI index

Excluded Studies	Pooled RR After Exclusion	Lower 95% CI	Upper 95% CI	I²(%)	Heterogeneity P-value	RR Change (%)
Zhihui Lu 2025	1.5673	1.3948	1.7612	66.24%	0.0185	325.56%
Wei Xian 2026	1.5376	1.3935	1.6967	65.13%	0.0217	129.92%
Yilin Pan 2025	1.5929	1.4229	1.7833	60.88%	0.0368	494.19%
Yinsong Xu 2025	1.4858	1.3389	1.6488	65.74%	0.0199	-211.74%
Songyuan Tang 2024	1.5028	1.3583	1.6626	67.65%	0.0148	-99.74%
Tonglong Jin 2026	1.4501	1.3114	1.6035	1.68%	0.3968	-446.48%

Table S62. Leave-one-out analysis for the association between CTI index (per 1-SD increase), (B) categorised CTI index (highest vs. reference category) and risks of stroke incidence. CTI, C-Reactive Protein-Triglyceride Glucose Index. CVD, Cardiovascular Disease

(A)CTI index (per 1-SD increase)

Excluded Studies	Pooled RR After Exclusion	Lower 95% CI	Upper 95% CI	I²(%)	Heterogeneity P-value	RR Change (%)
Wei Xian 2026	1.2537	1.1922	1.3183	0	0.7185	+0.81%
Wei Xian 2026	1.2452	1.1857	1.3078	0	0.4881	+0.13%
Ze-Jiaxin Niu 2026	1.2354	1.1486	1.3287	0	0.4842	-0.66%
Yinsong Xu 2025	1.2287	1.1661	1.2946	0	0.762	-1.20%
Songyuan Tang 2024	1.2503	1.1862	1.3178	0	0.5195	+0.54%

(B) Categorised CTI index

Excluded Studies	Pooled RR After Exclusion	Lower 95% CI	Upper 95% CI	I²(%)	Heterogeneity P-value	RR Change (%)
Wei Xian 2026 (eGFR ≥ 90)	1.7288	1.4354	2.0821	0.00%	0.5552	616.69%
Wei Xian 2026 (60 ≤ eGFR ≤ 89)	1.594	1.3401	1.896	6.50%	0.3432	-211.10%
Yinsong Xu 2025	1.5567	1.2299	1.9703	39.60%	0.191	-440.26%
Songyuan Tang 2024	1.6139	1.3137	1.9826	44.15%	0.1669	-88.94%
